# Catalytic system-controlled divergent reactions of pyrazolidinones with 3-alkynyl-3-hydroxyisoindolinones to construct diversified nitrogen-containing heterocyclic scaffolds†

**DOI:** 10.1039/d5ra01992c

**Published:** 2025-06-26

**Authors:** Zhenwei Zhang, Xiaoxue Zhang, Hao Wang, Geyao Xie, Yu Zhou

**Affiliations:** a School of Pharmaceutical Science and Technology, Hangzhou Institute for Advanced Study, University of Chinese Academy of Sciences Hangzhou 310024 China zhouyu@simm.ac.cn zhangzhenwei@ucas.ac.cn; b Drug Discovery & Development Center, Shanghai Institute of Materia Medica, Chinese Academy of Sciences Shanghai 201203 China; c University of Chinese Academy of Sciences Beijing 100049 China; d School of Chinese Materia Medica, Nanjing University of Chinese Medicine Nanjing 210023 China

## Abstract

A catalytic system-controlled divergent reaction was reported to construct three distinct nitrogen-containing heterocycles from readily available starting materials *via* a precise chemical bond activation and annulation cascade. Notably, this is the first capture of pyrazolidinones and propargyl alcohols to construct tetrahydropyrimidinones *via* selective N–N bond activation and to generate previously unreported 3-acylindoles. This protocol demonstrates a broad substrate scope, moderate to good yields, and valuable transformations, laying a robust foundation for drug discovery applications.

## Abstract 2

A catalytic system-controlled divergent reaction was reported to construct three distinct nitrogen-containing heterocycles from readily available starting materials *via* a precise chemical bond activation and annulation cascade.

## Graphical abstract

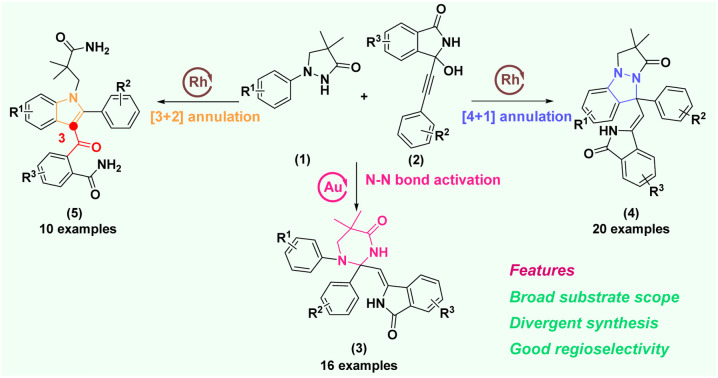

## Introduction

**Scheme 1:**
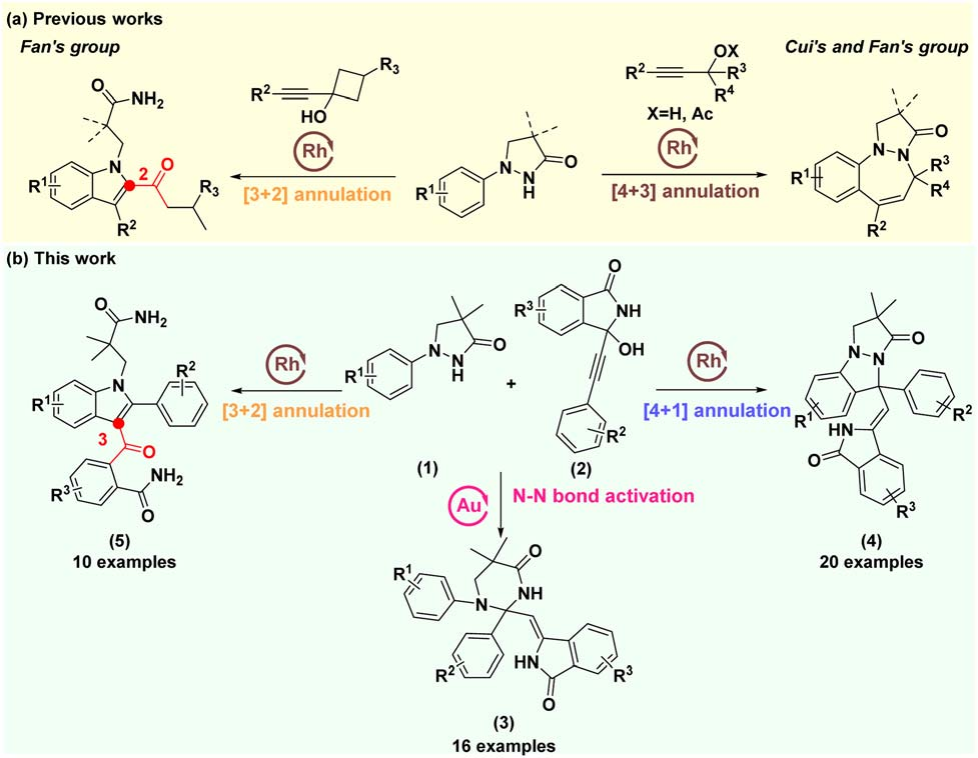
Previous works and this work.

Nitrogen-containing heterocyclic structures are highly valued patterns present in a wide range of natural products, pharmaceuticals, agrochemicals, and organic materials.^1–3^ Recently, pyrazolidinones with a free NH moiety have been employed as a traceless directing group for constructing diverse privileged nitrogen-containing heterocyclic frameworks, including N-substituted indoles,^4–6^ pyrazolo[1,2-*a*]pyrazolones,^7–9^*N*,*N*-bicyclic pyrazolidinones,^10,11^ diazepines^12–14^ and CF_3_-tethered indazoles.^15^ The propargylic alcohols have also been successfully involved in constructing diverse nitrogen-containing heterocyclic compounds.^16,17^ In 2020, Fan's group developed a pioneering Rh(iii)-catalyzed redox-neutral coupling of 1-phenylpyrazolidinones with alkynyl cyclobutanols, enabling selective construction of 2-acylindoles and pyrazolo[1,2-*a*]pyrazolones through [3 + 2] and [4 + 1] annulations, respectively, by adjusting reaction conditions (Scheme 1a, left).^18^ In 2018, Ji's group reported three examples where Rh(iii)-catalyzed [4 + 1] annulation of substituted 1-phenylpyrazolidinones with 1-phenylbut-2-yn-1-ol was used to construct desired pyrazolo[1,2-*a*]indazolones.^19^ In 2020, the groups of Cui and Fan independently accomplished the Rh(iii)-catalyzed [4 + 3] annulations of pyrazolidinones with propargyl alcohols or propargylic acetates to construct benzodiazepines (Scheme 1a, right).^20,21^ Despite these elegant works, they mainly focused on C–H bond activation/annulation cascades, with limited exploration of N–N bond activation for heterocyclic framework construction from these substrates.

In our continuous efforts to pursue rapid construction of diverse drug-like heterocyclic skeletons *via* transition-metal catalysed chemical bond activation/annulation cascades,^22–27^ we sought to develop more distinct and structurally complex nitrogen-containing heterocycles by employing the polycyclic propargyl alcohols. Interestingly, we observed an interesting phenomenon when the propargylic alcohols (2) with a large sterically hindered group were introduced as the coupling partner, 1-phenylpyrazolidinones (1) looked like a generalist that could be precisely and independently assembled into three distinct types of nitrogen-containing heterocyclic skeletons at different reaction sites of itself under different catalytic conditions (Scheme 1b). Of note, except for passing through a C(sp^2^)–H/N–H activation to generate pyrazolo[1,2-*a*]pyrazolones (4), we have captured a new N–N bond activation product tetrahydropyrimidinones (3) and an unusual product 3-acylindoles (5). These findings provide a versatile strategy for synthesizing diverse and biologically active nitrogen-containing heterocycles.

## Results and discussion

We initially investigated this transformation by employing the 4,4-dimethyl-1-phenylpyrazolidin-3-one (1a, 0.24 mmol) and 3-hydroxy-3-(phenylethynyl)isoindolin-1-one (2a, 0.2 mmol) as model substrates, and the results showed that the desired N–N bond activation product 3aa could be constructed with a 20% yield under [Cp*RhCl_2_]_2_ (5 mol%) as the catalyst and 1,2-dichloroethane (DCE) as the solvent at 90 °C for 4 h (Table 1, entry 1). Surprisingly, adding silver acetate and acetic acid into the catalytic system would result in a new [4 + 1] annulation product 4aa with 8% yield (Table 1, entry 2), while the addition of cesium pivalate and acetic acid into the catalytic system simultaneously formed 4aa (28% yield) and a [3 + 2] annulation product 5aa (10% yield) (Table 1, entry 3). Interestingly, the yield of 5aa could be increased to 50% when we used dioxane as the solvent (Table 1, entry 4). Encouraged by these results, we further screened different catalysts, including Rh_2_(esp)_2_, CyJohnPhos AuCl, (Ph_3_P)AuCl, Ph_3_PAuNTf_2_, CyJohnPhos AuNTf_2_ and AuCl(IPr) (Table 1, entries 5–10). The results showed that the AuCl(IPr) offered the best catalytic activity (Table 1, entry 10) for the formation of 3aa, the yield of which could be increased to 54%. Subsequent solvent screening indicated that DCE was the most conducive to this transformation (Table 1, entries 11–13). Next, lowering the reaction temperature to 70 °C could further increase the yield to 65% (Table 1, entry 14), while increasing the reaction temperature to 100 °C resulted in a slight decrease in yield (Table 1, entry 15). In brief, the optimal reaction conditions for 3aa were as follows: 1a (0.24 mmol), 2a (0.2 mmol), and AuCl(IPr) (5 mol%) in DCE at 70 °C for 4 h (condition A). Likewise, the optimal reaction conditions for 4aa and 5aa were also separately investigated (see Tables S1 and S2†). The results displayed that when using 1a (0.24 mmol), 2a (0.2 mmol), [Cp*Rh(CH_3_CN)_3_](SbF_6_)_2_ (5 mol%), LiOAc (0.2 mmol), BzOH (0.1 mmol) at 100 °C for 8 h (Table 1, entry 16, condition B) and 1a (0.2 mmol), 2a (0.24 mmol), [Cp*RhCl_2_]_2_ (5 mol%), CsOPiv (0.2 mmol) and AcOH (0.2 mmol) in diglyme at 80 °C for 2 h (Table 1, entry 17, condition C) as their respective catalytic systems, the products 4aa and 5aa were effectively prepared with 60% and 80%, respectively. Their structures were confirmed by ^1^H and ^13^C NMR, MS and X-ray spectra.

**Table 1:** Optimization of reaction conditions^a^

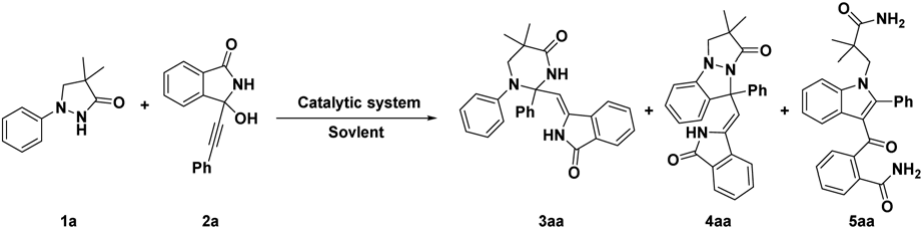					
Entry	Catalytic system	Solvent	Yield (%)		
			3aa	4aa	5aa
1	[Cp*RhCl_2_]_2_	DCE	20	—	—
2^b^	[Cp*RhCl_2_]_2_/AgOAc/AcOH	DCE	—	8	—
3^b^	[Cp*RhCl_2_]_2_/CsOPiv/AcOH	DCE	—	28	10
4^b^	[Cp*RhCl_2_]_2_/CsOPiv/AcOH	Dioxane	—	—	50
5	Rh_2_(esp)_2_	DCE	44	—	—
6	CyJohnPhos AuCl	DCE	31	—	—
7	CyJohnPhos AuNTf_2_	DCE	44	—	—
8	(Ph_3_P)AuCl	DCE	44	—	—
9	Ph_3_PAuNTf_2_	DCE	36	—	—
10	AuCl(IPr)	DCE	54	—	—
11	AuCl(IPr)	MeCN	15	—	—
12	AuCl(IPr)	CHCl_3_	41	—	—
13	AuCl(IPr)	HFIP	Trace	—	—
14^c^	AuCl(IPr)	DCE	65	—	—
15^d^	AuCl(IPr)	DCE	50	—	—
16^e^	[Cp*Rh(CH_3_CN)_3_](SbF_6_)_2_/LiOAc/BzOH	CH_3_CN	—	60	Trace
17^f^	[Cp*RhCl_2_]_2_/CsOPiv/AcOH	Diglyme	—	—	80

a Reaction conditions: 1a (0.24 mmol), 2a (0.2 mmol), catalyst (5.0 mol%) in solvent (4 mL) at 90 °C for 4 h, NMR yield (1,3,5-trimethoxybenzene as the internal standard).

b 1a (0.2 mmol), 2a (0.24 mmol), inorganic salts (0.4 mmol), AcOH (0.2 mmol) at 80 °C for 8 h, isolated yield.

c 70 °C.

d 100 °C.

e 1a (0.24 mmol), 2a (0.2 mmol), [Cp*Rh(CH_3_CN)_3_](SbF_6_)_2_ (5 mol%), LiOAc (0.2 mmol), BzOH (0.1 mmol) at 100 °C for 8 h, isolated yield.

f 1a (0.2 mmol), 2a (0.24 mmol), [Cp*RhCl_2_]_2_ (5 mol%), CsOPiv (0.2 mmol), AcOH (0.2 mmol) at 80 °C for 2 h, isolated yield.

**Scheme 2:**
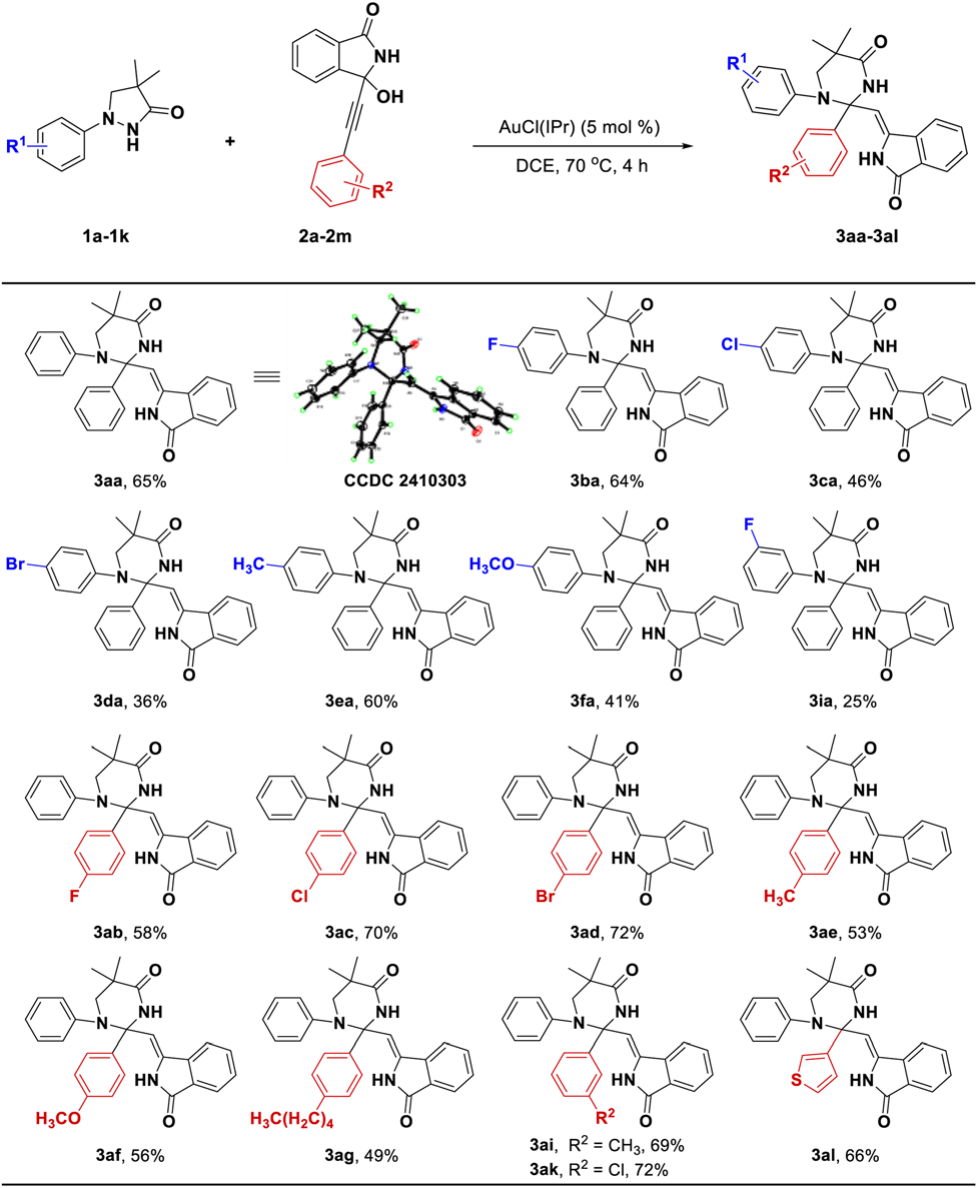
The investigation of substrate scope for the synthesis of 3. General reaction conditions (condition A): 1 (0.24 mmol), 2 (0.2 mmol), AuCl(IPr) (5 mol%) in DCE (4.0 mL) at 70 °C under air for 4 h, isolated yields.

With the optimized reaction conditions in hand, we firstly conducted a comprehensive investigation into the scope of pyrazolidinones 1 and coupling partners 2 under the optimal reaction condition to build diversified tetrahydropyrimidinones 3. As shown in Scheme 2, whether introducing halogen atoms (F, Cl and Br) or electron-donating groups (CH_3_ and OCH_3_) into 4-position of benzene ring of substrate 1, the reaction efficiencies were maintained at a moderate yield (Scheme 2, 3ba–3fa, 36–64%). However, introducing an F atom into 3-position of phenyl ring resulted in a decrease in yield (3ia, 25%). Subsequently, we investigated the effect of various substituents on the coupling partners 2. Notably, substrates 2 bearing halogen atoms (F, Cl and Br) or electron-donating groups (CH_3_, OCH_3_ and (CH_2_)_4_CH_3_) at the 4-position of the phenyl ring afforded corresponding products in moderate to good yields (3ab–3ag, 49–72%). Introducing CH_3_ and Cl groups into the 3-position of the phenyl ring also gave satisfactory results (3ai–3ak, 69–72%). Replacing the phenyl ring of 2 with the thiophene ring also provided a good yield (3al, 66%).

**Scheme 3:**
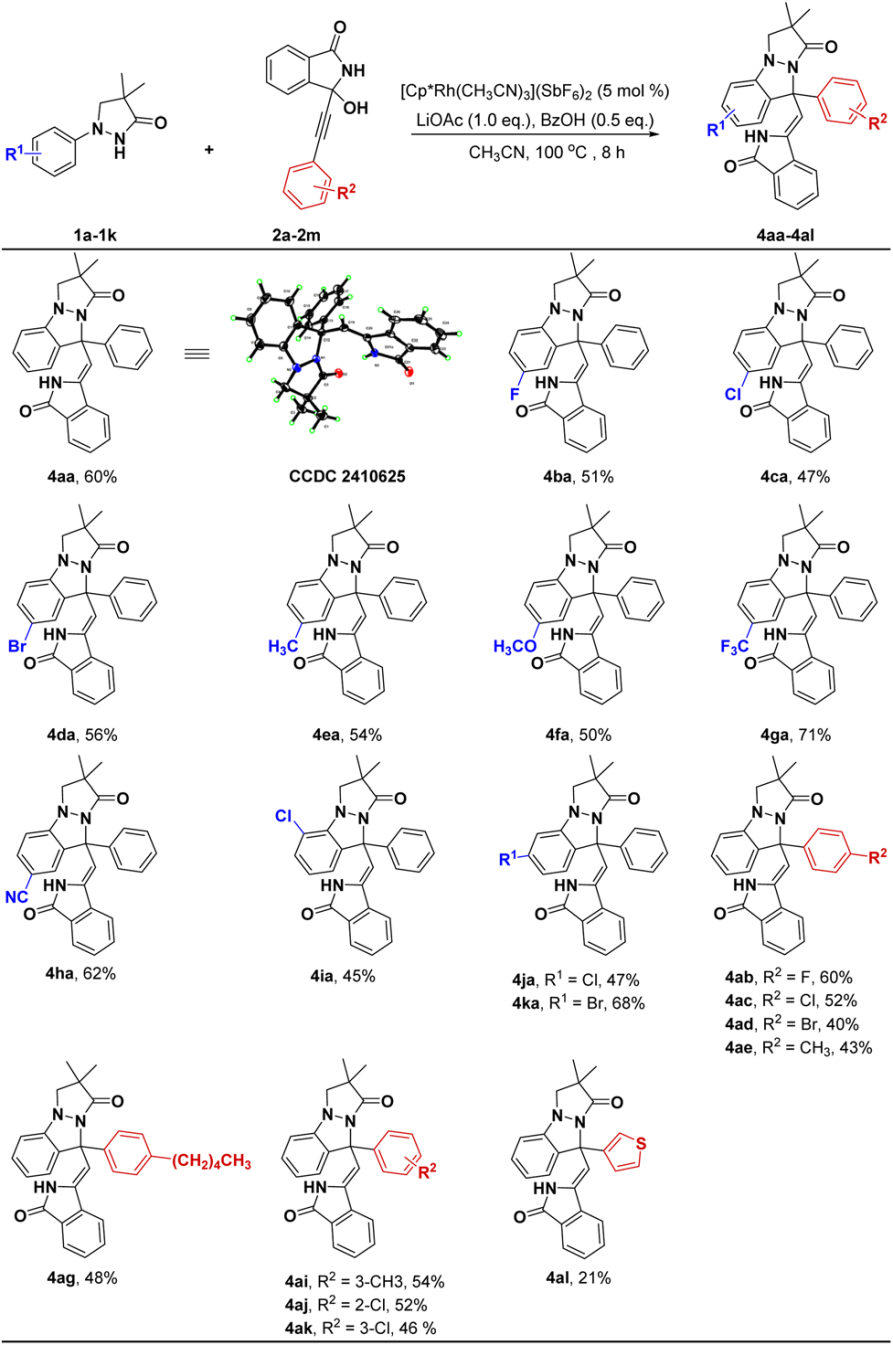
The investigation of substrate scope for the synthesis of 4. General reaction conditions (condition B): 1 (0.24 mmol), 2 (0.2 mmol), [Cp*Rh(CH_3_CN)_3_](SbF_6_)_2_ (5 mol%), LiOAc (0.2 mmol), BzOH (0.1 mmol) in MeCN (4.0 mL) at 100 °C under air for 8 h, isolated yields.

During the reaction condition screening, we observed the pyrazolo[1,2-*a*]pyrazolone derivative 4aa could be selectively constructed *via* a C(sp^2^)–H bond activation/[4 + 1] annulation cascade. As shown in Scheme 3, the reaction worked well with electron-withdrawing and electron-donating groups at the 4-position of the phenyl ring of substrates 1, affording 4ba–4ha with 47–71% yields (Scheme 3). Translocating Cl or Br to the 3-position of the phenyl ring could also successfully generate corresponding products with 45–68% yields (4ia–4ka). Next, various substituted groups at the 4-position of the phenyl ring of substrate 2 were explored, and the results indicated that all substrates could react smoothly with 1a to provide the corresponding products 4ab–4ag in moderate to good yields (40–60%). Likewise, introducing CH_3_ or Cl into the *meta* or *ortho* position of the benzene ring was also satisfactory (4ai–4ak). However, replacing benzene ring of 2 with thiophene ring gave a relatively low yield (4al, 21%).

**Scheme 4:**
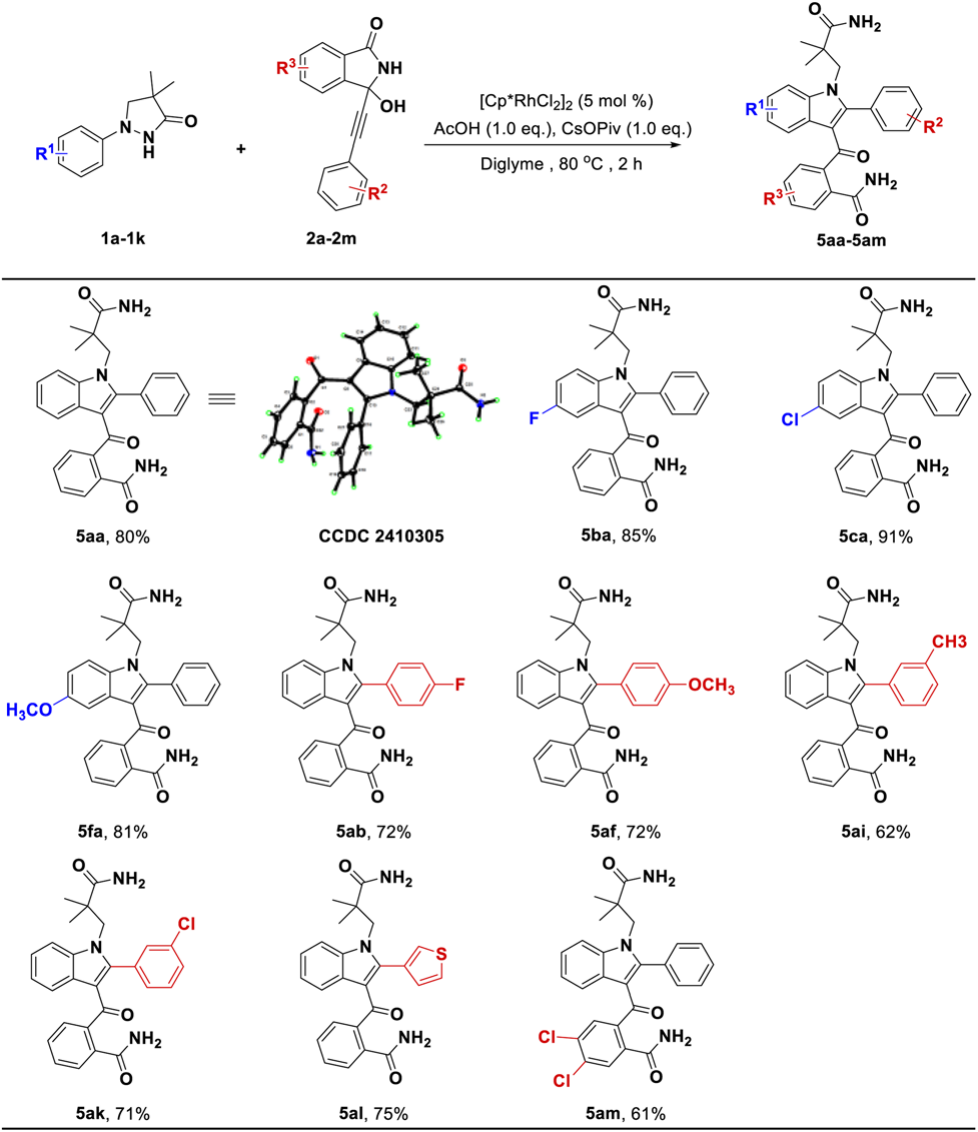
The investigation of substrate scope for the synthesis of 5. General reaction conditions (condition C): 1 (0.2 mmol), 2 (0.24 mmol), [Cp*RhCl_2_]_2_ (5 mol%), CsOPiv (0.2 mmol), AcOH (0.2 mmol) in diglyme (4.0 mL) at 80 °C under air for 2 h, isolated yields.

Another discovery was that we successfully obtained previously unreported 3-acylindoles *via* a C(sp^2^)–H bond activation and [3 + 2] annulation cascade from pyrazolidinone 1 and its coupling partner 2. As shown in Scheme 4, whether introducing electron-donating or electron-withdrawing groups into pyrazolidinones 1 or 3-alkynyl-3-hydroxyisoindolinones 2 could generate desired products in good yields (5ba–5fa, 81–91%; 5ab–5ak, 62–72%). In particular, in this transformation, replacing the benzene ring of 2 with the thiophene ring could provide a good yield (Scheme 4, 5al, 75%). Additionally, we also attempted to introduce the group into R^3^ position of substrate 2, the corresponding product 5am was obtained with good yield.

**Scheme 5:**
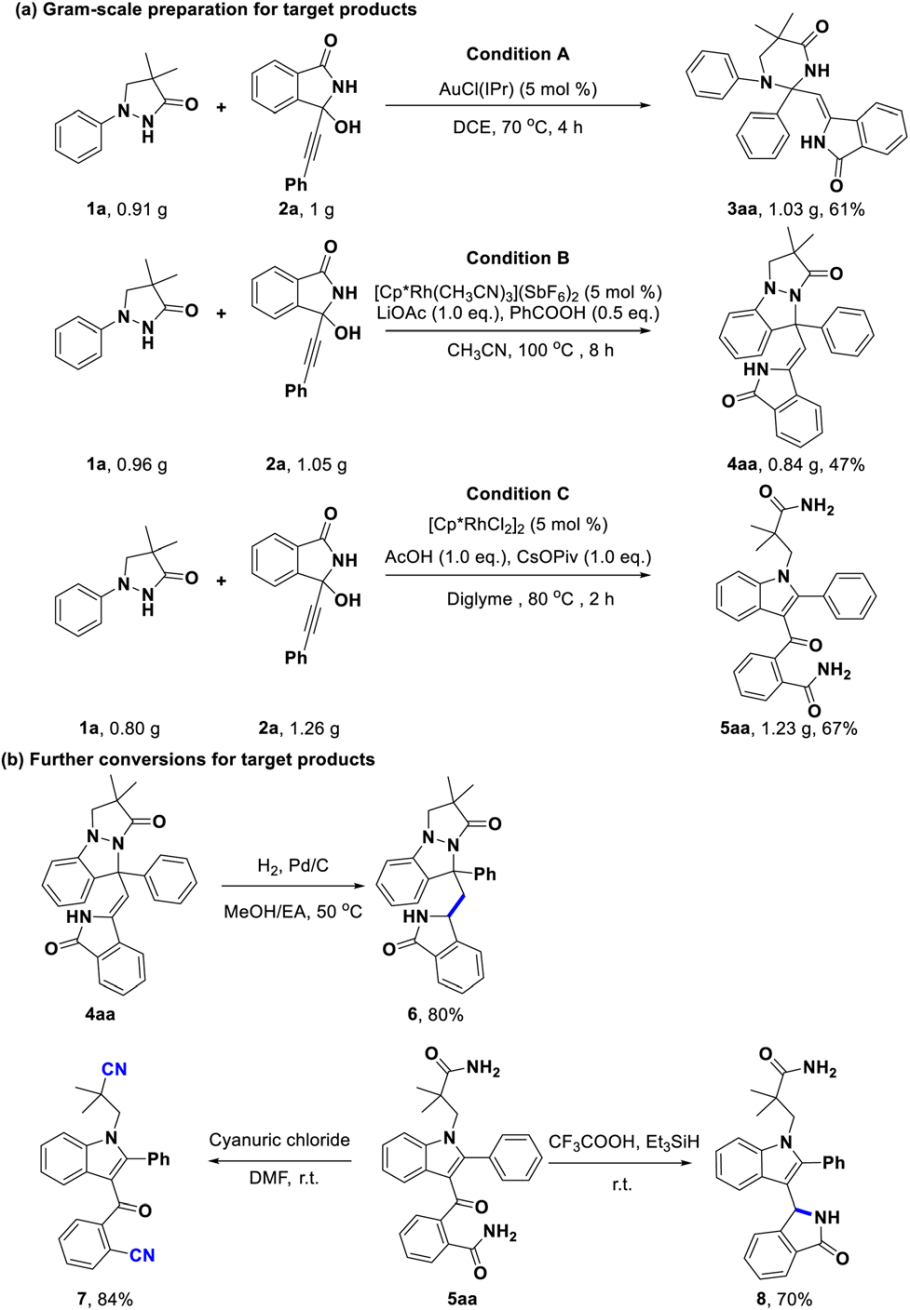
Gram-scale preparations and conversions.

Given the potential application of these intriguing heterocycles, the gram-scale preparations for the products 3aa, 4aa, and 5aa were performed with 61%, 47%, and 67% isolated yields, respectively (Scheme 5a). Then, the synthetic transformations of compounds 4aa and 5aa were also explored. For example, the C–C double bond of 4aa could be selectively reduced using hydrogen gas and Pd/C, generating the desired product 6 with 80% yield (Scheme 5b). Interestingly, we successfully fulfilled a simultaneous transformation of two amide groups in 5aa, in which compound 5aa could be converted into nitrile product 7 with 84% yield in the presence of cyanuric chloride (Scheme 5b). Additionally, treatment of 5aa with triethylsilane and trifluoroacetic acid afforded a ring-closed product 8 (Scheme 5b).

**Scheme 6:**
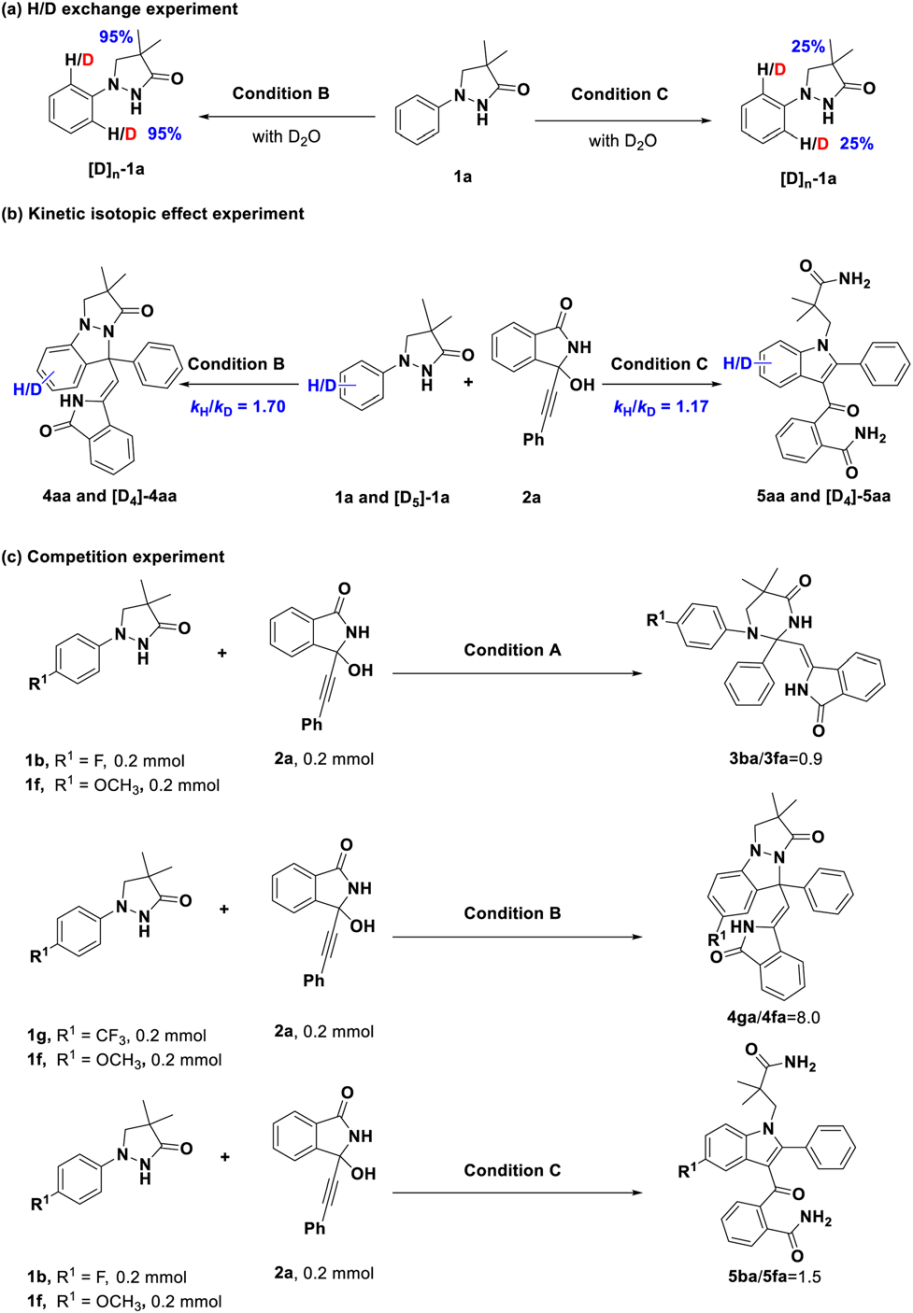
Preliminary mechanism studies.

Having established the substrate scope and utility of the product, we then conducted a series of experiments to explore the preliminary mechanism (Scheme 6). Firstly, 95% of *ortho* H could be deuterated by treating 1a with D_2_O under standard condition B, 25% of the *ortho* H could be deuterated by treating 1a with D_2_O under standard condition C, suggesting the reversibility of the C(aryl)–H bond cleavage during the formation of products 4 and 5 (Scheme 6a). Next, the kinetic isotope effect (KIE) values of 1.70 for 4aa and 1.17 for 5aa indicated that the C–H bond cleavage was likely not the turnover-limiting step (Scheme 6b). Moreover, three competitive experiments within three reaction conditions by introducing different substrates with electron-withdrawing and electron-donating groups were carried out. Treating a 1 : 1 mixture of fluorine/methoxy substituted pyrazolidinone (1b/1f) with 2a under conditions A and C, respectively, showed that there is no apparent difference in the reaction rate between two substrates 1 with electron-withdrawing and electron-donating groups (Scheme 6c). These results indicated that these different groups are well tolerated in these transformations. However, we observed a high ratio (8.0) between 4ga and 4fa when treating a 1 : 1 mixture of trifluoromethyl/methoxy substituted pyrazolidinone (1g/1f) with 2a under condition B (Scheme 6c), suggesting that the electron-withdrawing substrates reacted faster in this transformation.

**Scheme 7:**
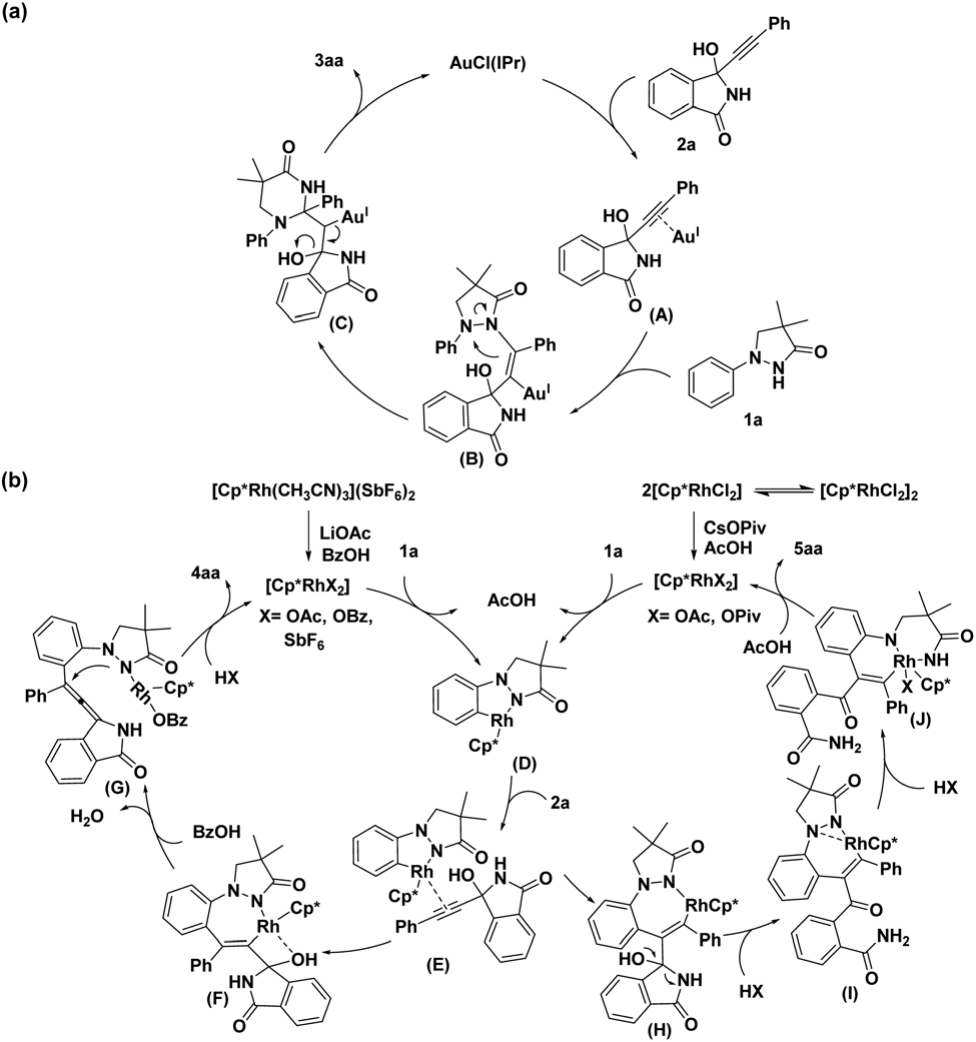
Plausible mechanisms.

Based on the above observations and literature precedents,^18,21,24,28–32^ three plausible catalytic mechanisms are proposed for N–N bond activations and C–H functionalizations. As shown in Scheme 7a, the propargylic alcohol 2a initially forms the intermediate A by coordinating with the Au(i) catalyst. Upon the entry of the pyrazolone 1a into the system, it undergoes alkyne hydroamination with Au(i) to yield complex B, which further forms the intermediate C after the intramolecular electrophilic addition. Finally, elimination of the Au(i) catalyst and the hydroxy group from C gives the desired product 3aa. The speculated mechanisms of the desired products 4aa and 5aa are shown in Scheme 7b. The NH directing group of 1a assists the activated Rh catalyst with the cleavage of the *ortho* C(sp^2^)–H bond, enabling the formation of a five-membered rhodacycle D. Next, the complexation of Rh(iii) with the triple bond of 2a gives intermediate E. The following regioselective insertion of the alkynyl unit of 2a into the C–Rh bond of E generates intermediate F, which is most likely driven by additional coordination of the OH group with Rh(iii) to overcome the unfavorable steric interactions between the isoindolin-1-one group and the Cp ligand. Further, the cleavage of the C–Rh bond and elimination of water under the assistance of benzoic acid offers the allenyl intermediate G. Finally, G undergoes nucleophilic addition and followed by protodemetalation to form target product 4aa. Specially, using [Cp*RhCl_2_]_2_ as the catalyst in diglyme results in the opposite regioselective insertion of the alkyne unit of 2a into the C–Rh bond of E to form intermediate H, probably because the solvent prevents the additional coordination of the OH group of propargylic alcohol with Rh(iii) complex. Then protonation of intermediate H leads to the generation of intermediate I, which then undergoes an oxidative addition with N–N bond to form intermediate J. Finally, intermediate J undergoes a reductive elimination and a following *N*-demethylation/protonation to produce the desired product 5aa.

## Conclusions

In summary, we have successfully fulfilled a catalytic system-controlled divergent reaction strategy to construct three types of nitrogen-containing heterocyclic skeletons through chemical bond activation/annulation routes of pyrazolidinones with 3-alkynyl-3-hydroxyisoindolinones. More importantly, this is the first capture of pyrazolidinones and propargyl alcohols to construct tetrahydropyrimidinones and unusual 3-acylindoles. These strategies exhibit a broad range of substrates, moderate to good yields, and valuable transformations, offering structural and biological potentials for further drug discovery.

## Conflicts of interest

The authors declare no competing interests.

## Supplementary Material

RA-015-D5RA01992C-s001

RA-015-D5RA01992C-s002

RA-015-D5RA01992C-s003

RA-015-D5RA01992C-s004

## Data Availability

The data supporting this article have been included as part of the ESI,† which includes experimental details, characterization data, NMR spectra, and HPLC spectra, along with single-crystal X-ray data for compounds 3aa (CCDC 2410303), 4aa (CCDC 2410625), and 5aa (CCDC 2410305).
