# Supplementary material for: Catalytic system-controlled divergent reactions of pyrazolidinones with 3-alkynyl-3-hydroxyisoindolinones to construct diversified nitrogen-containing heterocyclic scaffolds
Source: RSC Adv. 2025 Jun 26;15(27):21872–8. doi: 10.1039/d5ra01992c (PMC12198952; doi:10.1039/d5ra01992c)
Supplement: RA-015-D5RA01992C-s001 [file RA-015-D5RA01992C-s001.pdf]

Supplementary Information (SI) for RSC Advances.
This journal is © The Royal Society of Chemistry 2025

Supplementary information

Catalytic system-controlled divergent reactions of pyrazolidinones with 3-alkynyl-3-hydroxyisoindolinones to construct diversified nitrogen-containing heterocyclic scaffolds

Zhenwei Zhang,*‡[abc] Xiaoxue Zhang,‡[bcd] Hao Wang,[bc] Geyao Xie[abc] and Yu Zhou*[abcd]

[a]School of Pharmaceutical Science and Technology, Hangzhou Institute for Advanced Study, University of Chinese Academy of Sciences, Hangzhou 310024, China.

[b]Drug Discovery & Development Center, Shanghai Institute of Materia Medica, Chinese Academy of Sciences, Shanghai 201203, China.

[c]University of Chinese Academy of Sciences, Beijing 100049, China.

[d]School of Chinese Materia Medica, Nanjing University of Chinese Medicine, Nanjing 210023, China.

‡These authors contributed equally: Zhenwei Zhang, Xiaoxue Zhang

*E-mail: zhangzhenwei@ucas.ac.cn (Zhenwei Zhang); zhouyu@simm.ac.cn (Yu Zhou).

Contents

I. General experimental information.........3

II. Synthesis of substrates.........3

III. Optimization of Reaction Conditions.........6

IV. General procedure for the products.........7

V. Gram-scale preparations and conversions.........10

VI. Mechanistic studies.........14

VII. Characterization of the products.........21

VIII. X-ray Crystallographic Data.........45

IX. NMR Spectra of Products.........47

X. HPLC Data of Products.........96

I. General experimental information

Unless otherwise specified, the reagents were purchased from commercial sources, and used without further purification. All products were characterized by their NMR and MS spectra. ^{1}H and $^{13}C\{^{1}H\}$ NMR spectra were recorded on a 400, 500 or 600 MHz instrument. The chemical shifts were reported in parts per million (ppm, δ) downfield from tetramethylsilane (TMS). Proton coupling patterns were described as singlet (s), doublet (d), triplet (t), quartet (q), multiplet (m), doublet of doublets (dd), doublet of triplets (dt), triplet of doublets (td), doublet of doublet of doublets (ddd), doublet of doublet of triplets (ddt) and broad (br). High resolution mass spectra (HRMS) were measured on a Micromass Ultra Q-TOF spectrometer. Analytical thin-layer chromatography (TLC) was performed on HSGF 254 (0.2-0.3 mm thickness). Column chromatography was performed on silica gel (300-400 mesh) using ethyl acetate (EA)/petroleum ether (PE) or dichloromethane (DCM)/methanol (MeOH). HPLC analysis of target compounds was performed on an Agilent 1260 HPLC instrument with a binary pump and a photodiode array detector (DAD), using an Agilent Extend-C18 column (4.6 mm × 150 mm, 5 μm) at 1.0 mL/min flow monitored by UV absorption at 254 nm. Mobile phase A: H_2O, mobile phase B: MeOH.

Time/min	%A	%B
0	20	80
10	20	80

II. Synthesis of substrates

The pyrazolidinones **1a**-**1k**, 3-alkynyl-3-hydroxyisoindolinones **2a**-**2m**, and deuterated pyrazolidinone **[D$_5$]-1a** were prepared based on literature procedures,[1-4] if needed, which were recrystallized before use. The detailed synthesis procedures of **1a**, **2a**, **[D$_5$]-1a** are as follows:

(1) Synthesis of pyrazolidinone (1a)

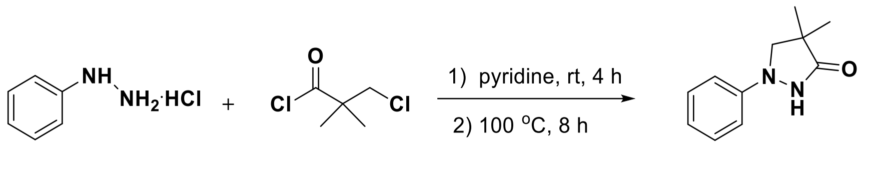

1a

The 3-chloro-2,2-dimethylpropionyl chloride (1.06 g, 8.87 mmol, 1.0 eq.) was added dropwise to a solution of phenylhydrazine hydrochloride (1g, 6.87 mmol, 1.0 eq.) in pyridine (40 mL). The resulting mixture was stirred at room temperature for 4 h and then stirred at 100 °C for 8 h. The reaction mixture was poured into 200 mL aqueous HCl (2 M) and extracted with ethyl acetate (50 mL × 3). After then, the organic layer was washed with saturated sodium carbonate solution (100 mL × 3), dried over anhydrous Na_2SO_4, filtrated and concentrated in vacuo. The residue was purified by flash chromatography (PE/EA=3/1, v/v) to afford the corresponding pyrazolidinone **1a**.

(2) Synthesis of 3-hydroxy-3-(phenylethynyl) isoindolin-1-one (2a)

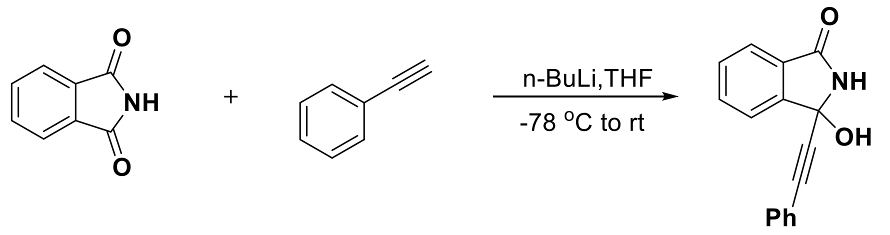

2a

A solution of *N*-butyl lithium (2.61g, 40.78 mmol, 4.0 eq., 2.5 M in petroleum ether solution) was added dropwise to a solution of phenylacetylene (4.17g, 40.78 mmol, 4.0 eq.) in THF (30 mL) at -78 °C and then the reaction mixture was stirred for 30 min. After then, a solution of phthalimide (1.5 g, 10.19 mmol,1.0 eq.) in THF (15 mL) was added to the reaction mixture. The resulting mixture was stirred at -78 °C for 30 min and stirred at room temperature for 4 h. Upon completion as indicated by TLC, the reaction mixture was quenched with saturated ammonium chloride and added 1N HCl to adjust pH=5.0. The aqueous solution was extracted with ethyl acetate (50 mL×3), washed with saturated sodium chloride solution (100 mL × 3), dried over anhydrous

Na_2SO_4, filtrated and concentrated in vacuo. The residue was purified by flash chromatography over silica gel using PE/EA (1:1) to afford the corresponding pyrazolidinone **2a**.

(3) Synthesis of [D$_5$]-1a

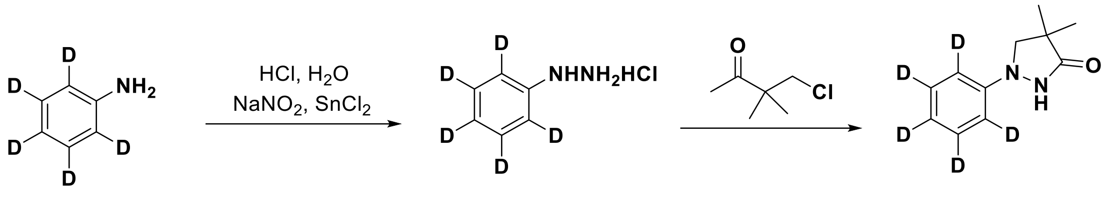

Synthesis of phenylhydrazine hydrochloride D$_5$ (B): A solution of $NaNO_2$ (718 mg, 10.4 mmol) in water (3 mL) was added dropwise to a solution of **A** (1.0 g, 10 mmol) in 37% HCl (5 mL). The resulting mixture was stirred at 0 °C for 20 min and added a saturated solution of tin(II) chloride dihydrate (2.67 g, 14 mmol) in 37% HCl. Then the mixture was stirred at 0 °C for 20 min and filtered to obtain **B** (1.5 g).

Synthesis of [D$_5$]-1a: The pyrazolidinone **[D$_5$]-1a** was prepared from deuterated phenylhydrazine hydrochloride according to the synthesis procedure of **1a**. **^{1}H NMR (400 MHz, DMSO-*d*$_6$)** δ 10.20 (s, 1H), 3.68 (s, 2H), 1.01 (s, 6H).

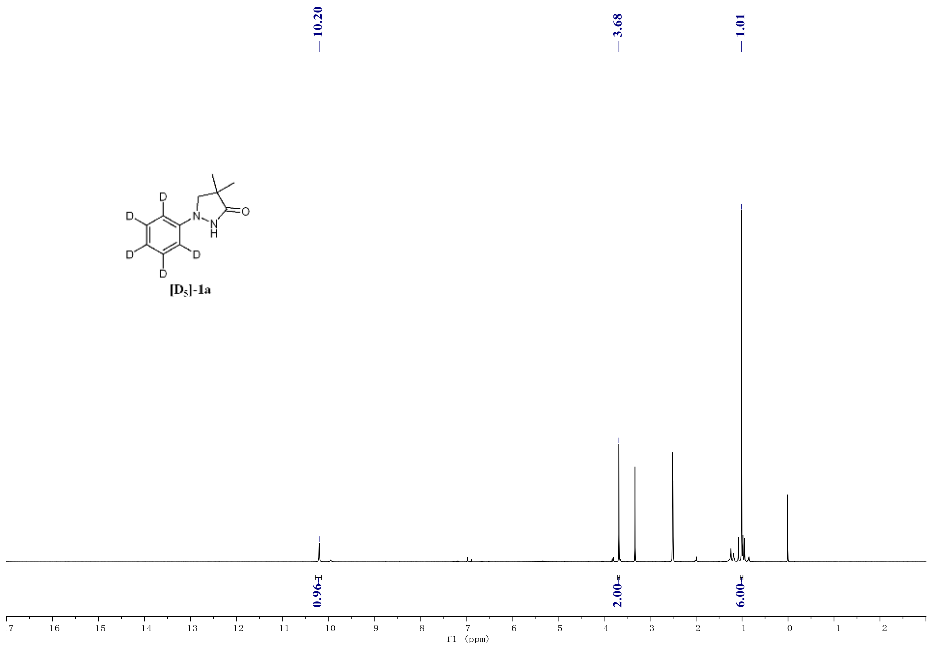

III. Optimization of Reaction Conditions

Table S1. Optimization of reaction conditions for 4aa[a]

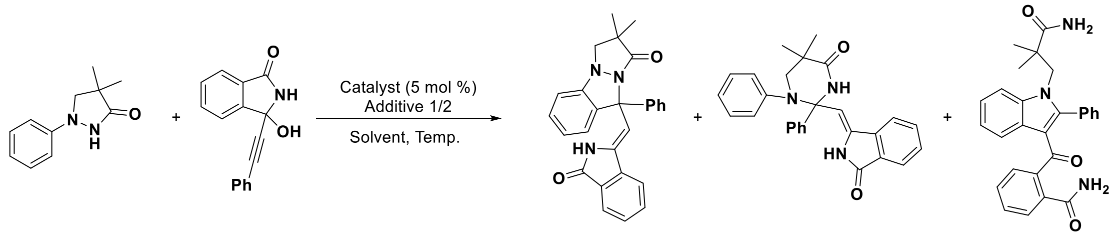

Entry	Catalyst	Additive 1	Additive 2	Solvent	Yield(%)[b] **4aa (3aa/5aa)**
1	$[Cp^{*}RhCl_2]_2$	AgOAc	AcOH	DCE	8(nd/nd)
2	$[Cp^{*}RhCl_2]_2$	NaOAc	AcOH	DCE	24(nd/10)
3	$[Cp^{*}RhCl_2]_2$	LiOAc	AcOH	DCE	5(48/nd)
4	$[Cp^{*}RhCl_2]_2$	CsOPiv	AcOH	DCE	28(nd/10)
5	$[Cp^{*}RhCl_2]_2$	CsOPiv	AcOH	Dioxane	nd(nd/50)
6	$[Cp^{*}RhCl_2]_2$	LiOAc	AcOH	CH_3CN	25(36/trace)
7	$[Cp^{*}Rh(CH_3CN)_3](SbF_6)_2$	LiOAc	AcOH	CH_3CN	46(0/trace)
8	$[Cp^{*}Rh(CH_3CN)_3](SbF_6)_2$	LiOAc	AcOH	Acetone	40(nd/12)
9	$[Cp^{*}Rh(CH_3CN)_3](SbF_6)_2$	LiOAc	AcOH	MeOH	nd(nd/10)
10	$[Cp^{*}Rh(CH_3CN)_3](SbF_6)_2$	LiOAc	AcOH	$CHCl_3$	37(nd/nd)
11	$[Cp^{*}Rh(CH_3CN)_3](SbF_6)_2$	LiOAc	PivOH	CH_3CN	26(5/5)
12	$[Cp^{*}Rh(CH_3CN)_3](SbF_6)_2$	LiOAc	Benzoic acid	CH_3CN	52(nd/trace)
13[c]	$[Cp^{*}Rh(CH_3CN)_3](SbF_6)_2$	LiOAc	Benzoic acid	CH_3CN	nd(nd/nd)
14[d]	$[Cp^{*}Rh(CH_3CN)_3](SbF_6)_2$	LiOAc	Benzoic acid	CH_3CN	55(nd/trace)
15[e]	$[Cp^{*}Rh(CH_3CN)_3](SbF_6)_2$	LiOAc	Benzoic acid	CH_3CN	22(nd/nd)
16[f]	$[Cp^{*}Rh(CH_3CN)_3](SbF_6)_2$	LiOAc	Benzoic acid	CH_3CN	57(nd/trace)
17[g]	$[Cp^{*}Rh(CH_3CN)_3](SbF_6)_2$	LiOAc	Benzoic acid	CH_3CN	60(nd/trace)

[a]Reaction conditions: **1a** (0.2 mmol), **2a** (0.24 mmol), catalyst (5 mol%), additive 1 (0.4 mmol), additive 2 (0.2 mmol) in solvent (4 mL), at 80 °C for 8 h. [b]Isolated yield. [c]rt. [d]100 °C. [e]120 °C. [f] **1a**:**2a**=1.2:1 [g]**1a**:**2a**=1.2:1, additive 1 (0.2 mmol), additive 2 (0.1 mmol), 100 °C. nd = not detected

Table S2. Optimization of reaction conditions for 5aa[a]

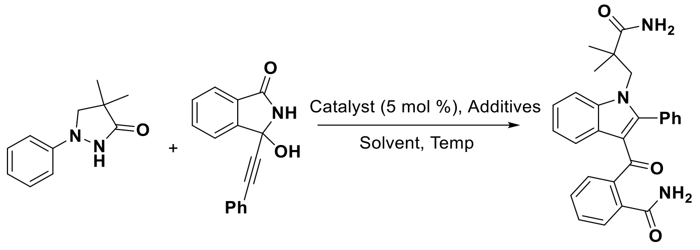

1a 2a 5aa

Entry	Catalyst	Additive 1	Additive 2	Solvent	Yield(%)[b] **5aa**
1	$[Cp^*RhCl_2]_2$	CsOPiv	AcOH	Dioxane	50
2	$[Cp^*RhCl_2]_2$	Cs_2CO_3	AcOH	Dioxane	45
3	$[Cp^*RhCl_2]_2$	CsF	AcOH	Dioxane	48
4	$[Cp^*RhCl_2]_2$	CsOAc	AcOH	Dioxane	40
5	$[Cp^*RhCl_2]_2$	Na_2CO_3	AcOH	Dioxane	31
6	$[Cp^*RhCl_2]_2$	NaOPiv	AcOH	Dioxane	25
7	$[Cp^*RhCl_2]_2$	CsOPiv	AcOH	THF	41
8	$[Cp^*RhCl_2]_2$	CsOPiv	AcOH	Diglyme	75
9[c]	$[Cp^*RhCl_2]_2$	CsOPiv	AcOH	Diglyme	80

[a]Reaction conditions: **1a** (0.2 mmol), **2a** (0.24 mmol), $[Cp^*RhCl_2]_2$ (5 mol%), additive 1 (0.4 mmol), additive 2 (0.2 mmol) in solvent (4 mL), at 80 °C for 8 h. [b]Isolated yield. [c]additive 1 (0.2 mmol), additive 2 (0.2 mmol), 2 h.

IV. General procedure for the products

(1) General procedure for the synthesis of 3 (Condition A)

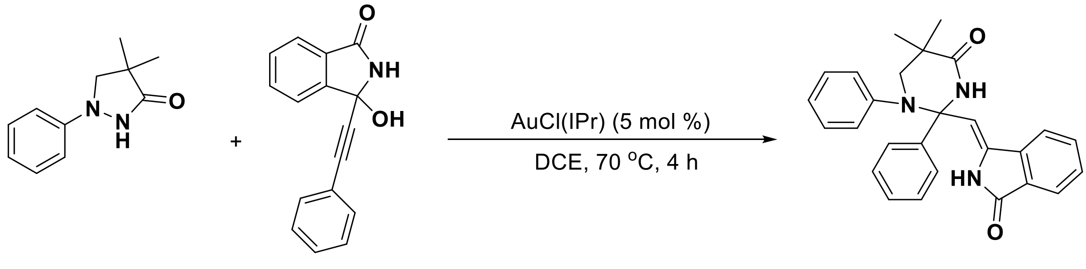

1a 2a 3aa

The AuCl(IPr) (0.01 mmol, 0.05 eq.) was added to a solution of **1a** (0.24 mmol, 1.2 eq.) and **2a** (0.2 mmol, 1.0 eq.) in DCE (4.0 mL) and the reaction mixture was stirred at 70 °C for 4 h. Upon completion as indicated by TLC, the mixture was poured into water (30 mL) and extracted with ethyl acetate (10 mL × 3). The combined organic layer was washed with brine (10 mL × 3), dried over anhydrous Na_2SO_4, filtrated and

concentrated under vacuum. The residue was purified by column chromatography (PE/EA=1/1, v/v) to afford **3aa**.

(2) General procedure for the synthesis of 4 (Condition B)

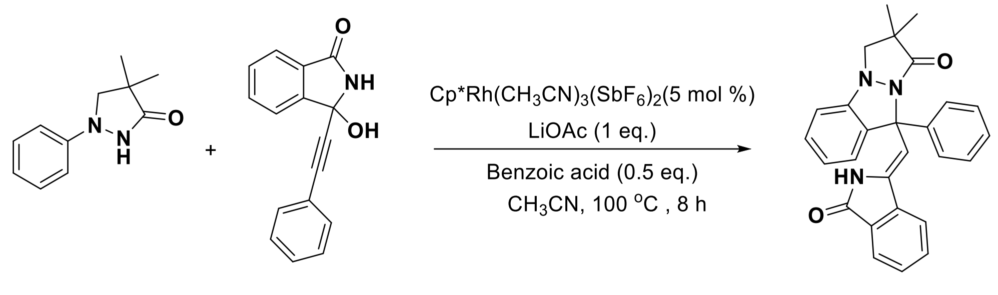

1a **2a** **4aa**

A suspension of **1a** (0.24 mmol, 1.2 eq.), **2a** (0.2 mmol, 1.0 eq.), $[Cp^*Rh(CH_3CN)_3](SbF_6)_2$ (0.01 mmol, 0.05 eq.) , LiOAc (0.2 mmol, 1.0 eq.), benzoic acid (0.1 mmol, 0.5 eq.) in MeCN (4.0 mL) was stirred at 100 °C for 8 h. Upon completion as indicated by TLC, the reaction mixture was poured into water (30 mL) and extracted with ethyl acetate (10 mL × 3). The combined organic layer was washed with brine (10 mL × 3), dried over anhydrous Na_2SO_4, filtrated and concentrated under vacuum. The residue was purified by column chromatography (PE/EA=1/1, v/v) to afford **4aa**.

(3) General procedure for the synthesis of 5 (Condition C)

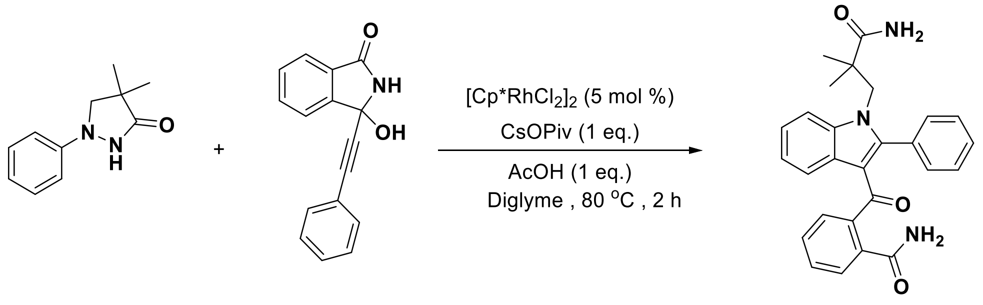

1a **2a** **5aa**

A suspension of **1a** (0.2 mmol, 1.0 eq.), **2a** (0.24 mmol, 1.2 eq.), $[Cp^*RhCl_2]_2$ (0.01 mmol, 0.05 eq.), CsOPiv (0.2 mmol, 1.0 eq.), AcOH (0.2 mmol, 1.0 eq.) in diethylene glycol dimethyl ether (4.0 mL) was stirred at 80 °C for 2 h. Upon completion as indicated by TLC, the reaction mixture was poured into water (30 mL) and extracted

with ethyl acetate (10 mL × 3). The combined organic layer was washed with brine (10 mL × 3), dried over anhydrous Na_2SO_4, filtrated and concentrated under vacuum. The residue was purified by column chromatography (PE/EA=1/1, v/v) to afford **5aa**.

V. Gram-scale preparations and conversions

(1) Gram-scale preparation

(a) Gram-scale preparation for 3aa

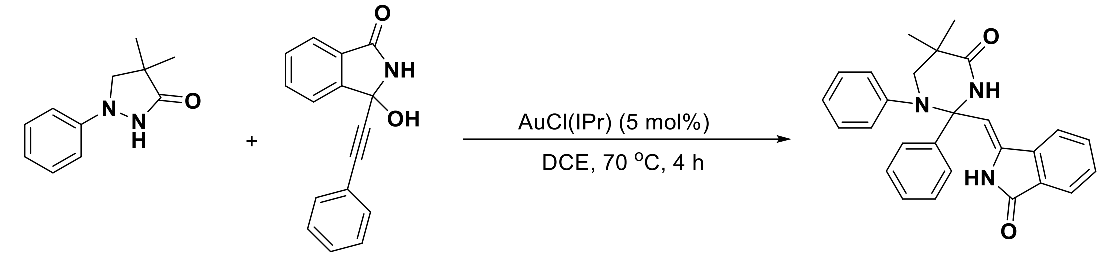

1a (0.91g, 4.8 mmol) **2a** (1 g, 4.0 mmol) **3aa** (1.03 g, 61%)

A suspension of **1a** (910 mg, 4.8 mmol), **2a** (1 g, 4.0 mmol), AuCl(IPr) (124.2 mg, 5 mol %) was combined in DCE (10 mL) was stirred at 70 °C for 4 h. Upon completion as indicated by TLC, the reaction mixture was poured into water (30 mL) and extracted with ethyl acetate (10 mL × 3). The combined organic layer was washed with brine (10 mL × 3), dried over anhydrous Na_2SO_4,filtrated and concentrated under vacuum. The residue was purified by column chromatography (PE/EA=1/1, v/v) to afford **3aa**.as white solid (1.03 g, 61% yield).

(b) Gram-scale preparation for 4aa

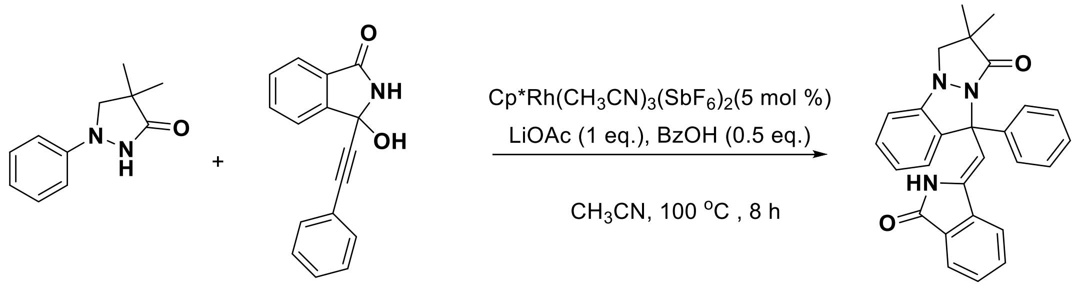

1a (0.96 g, 5.05 mmol) **2a** (1.05 g, 4.21 mmol) **4aa** (0.84 g, 47%)

A suspension of **1a** (960 mg, 5.05 mmol), **2a** (1.05 g, 4.21 mmol), $Cp^*Rh(CH_3CN)_3(SbF_6)_2$ (176.2 mg, 5 mol %) , LiOAc (277.2 mg, 4.21 mmol), benzoic acid (256.2 mg, 2.1 mmol) in MeCN (10 mL) was stirred at 100 °C for 8 h. Upon completion as indicated by TLC, the reaction mixture was poured into water (50 mL) and extracted with ethyl acetate (30 mL × 3). The combined organic layer was washed with brine (20 mL × 3), dried over anhydrous Na_2SO_4,filtrated and concentrated under

vacuum. The residue was purified by column chromatography (PE/EA=1/1, v/v) to afford **4aa** as yellow oil (0.84 g, 47% yield).

(c) Gram-scale preparation for 5aa

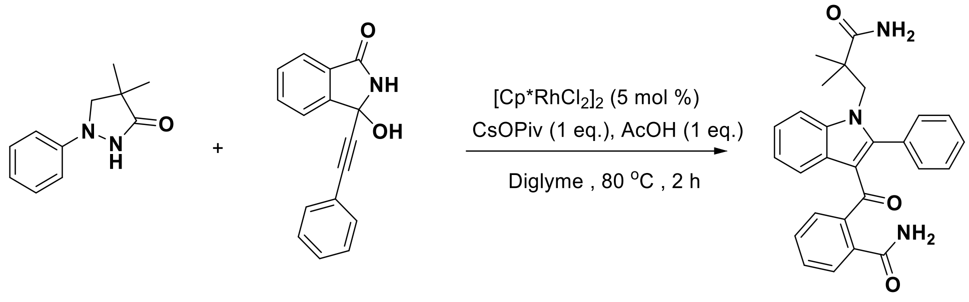

1a (0.8 g, 4.21mmol) **2a** (1.26 g, 5.05 mmol) **5aa** (1.23 g, 67%)

A suspension of **1a** (800 mg, 4.21 mmol), **2a** (1.26 g, 5.05 mmol), $[Cp^*RhCl_2]_2$ (131.6 mg, 5 mol %), CsOPiv (982.8 mg, 4.21 mmol), HOAc (152 mg, 4.21 mmol) in diethylene glycol dimethyl ether (10 mL) was stirred at 80 °C for 2 h. Upon completion as indicated by TLC, the reaction was poured into water (50 mL) and extracted with ethyl acetate (30 mL × 3). The combined organic layer was washed with brine (20 mL × 3), dried over anhydrous Na_2SO_4, filtrated and concentrated under vacuum. The residue was purified by column chromatography (PE/EA = 1:1, v/v) to afford **5aa** as yellowish solid (1.23 g, 67% yield).

(2) Conversions of 4aa and 5aa

(a) Procedure and Characterization of Compound 6:

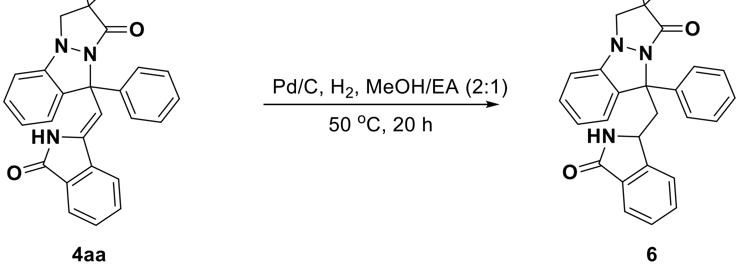

4aa **6**

The 10 mol % Pd/C (10 mg) was added to a solution of **4aa** (100 mg, 0.24 mmol) in MeOH/EtOAc (v/v=2/1, 20 mL) under the hydrogen environment. The resulting

mixture was stirred at 50°C for 20 h. Upon completion as indicated by TLC, the mixture was filtered by suction and the filtrate was extracted with ethyl acetate (30 mL×3) and concentrated in vacuo. The residue was purified by column chromatography (PE/EA=1/1, v/v) to afford **6** as white solid (80 mg, 80% yield).

(6) 2,2-dimethyl-9-((3-oxoisoindolin-1-yl)methyl)-9-phenyl-2,3-dihydro-1*H*,9*H*-pyrazolo[1,2-*a*]indazol-1-one

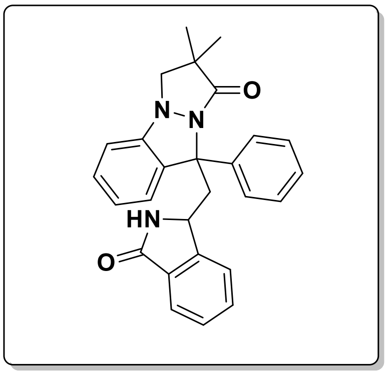

^{1}H NMR (400 MHz, Chloroform-*d*) δ 7.83 (dd, *J* = 7.2, 1.8 Hz, 1H), 7.60 – 7.51 (m, 1H), 7.49 – 7.42 (m, 4H), 7.42 – 7.30 (m, 5H), 7.29 (d, *J* = 7.5 Hz, 1H), 7.20 – 7.14 (m, 1H), 6.89 (d, *J* = 7.9 Hz, 1H), 6.72 (s, 1H), 4.71 – 4.63 (m, 1H), 3.69 (d, *J* = 8.5 Hz, 1H), 3.54 (d, *J* = 8.5 Hz, 1H), 3.12 – 2.98 (m, 2H), 1.42 (d, *J* = 7.6 Hz, 6H). **^{13}C{^{1}H} NMR (101 MHz, Chloroform-*d*)** δ 170.80, 170.05, 147.59, 147.16, 140.55, 133.40, 131.87, 129.57, 128.79, 128.34, 128.27, 126.47, 123.82, 123.61, 123.31, 122.42, 110.50, 70.40, 63.91, 54.25, 45.89, 43.69, 22.94, 22.90. **HRMS (ESI) m/z**: calculated for $C_{27}H_{26}N_3O_2$ $[M + H]^+$:424.2022, found: 424.2020.

(b) Procedure and Characterization of Compound 7:

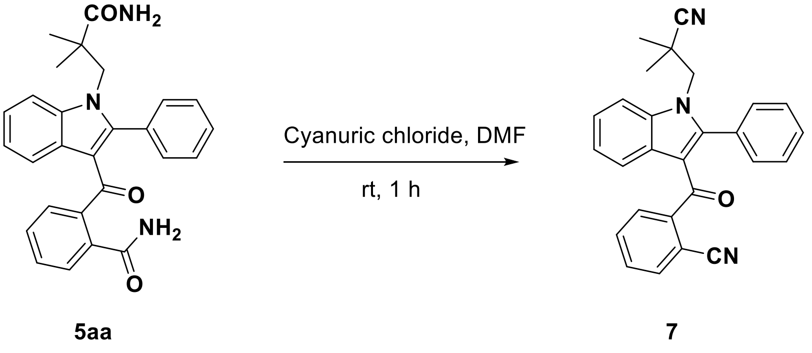

The solution of **5aa** (88 mg, 0.2 mmol) and cyanuric chloride (74 mg, 0.4 mmol) in DMF (3 mL) was stirred at room temperature for 1 h. Upon completion as indicated by TLC, the reaction mixture was quenched with water (30 mL), extracted with ethyl

acetate (15 mL×3), washed with brine (15 mL×3), dried over anhydrous Na_2SO_4, filtered, and concentrated under vacuum. The crude product was purified by column chromatography (PE/EA=2/1, v/v) to afford **7** as yellow oil (68 mg, 84% yield).

(7) 2-(1-(2-cyano-2-methylpropyl)-2-phenyl-1*H*-indole-3-carbonyl)benzonitrile

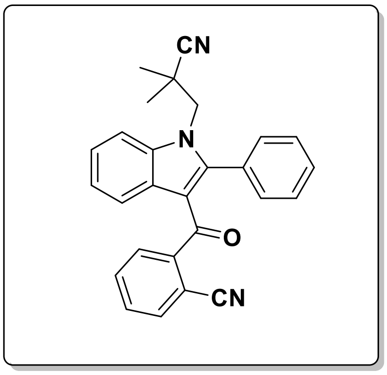

^{1}H NMR (400 MHz, Chloroform-*d*) δ 8.27 – 8.19 (m, 1H), 7.71 – 7.64 (m, 1H), 7.47 – 7.21 (m, 11H), 4.49 (s, 2H), 1.12 (s, 6H). **^{13}C NMR (126 MHz, Chloroform-*d*)** δ 188.86, 146.07, 143.19, 136.06, 131.94, 130.74, 128.85, 128.54, 128.37, 128.30, 127.28, 123.14, 122.64, 122.41, 121.09, 116.31, 115.27, 110.52, 109.95, 49.42, 33.23, 24.68. **HRMS (ESI) m/z**: calculated for $C_{27}H_{22}N_3O$ $[M + H]^+$:404.1759, found: 404.1757.

(c) Procedure and Characterization of Compound 8:

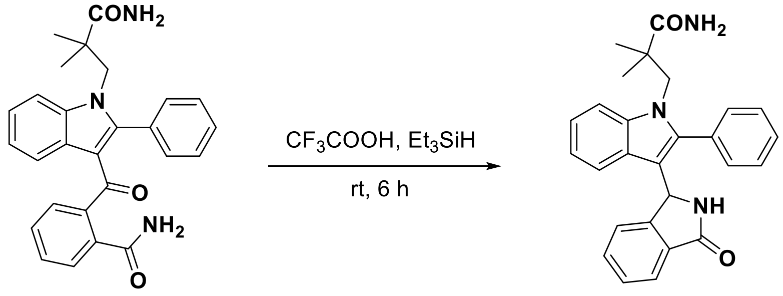

5aa **8**

The mixture of **5aa** (88 mg, 0.2 mmol), trifluoroacetic acid (2 mL) and triethylsilane (3 mL) was stirred at room temperature for 6 h. Upon completion as indicated by TLC, the mixture was quenched with 10 mL of saturated sodium carbonate solution, extracted with ethyl acetate (15 mL×3), washed with brine (15 mL×3), dried over anhydrous Na_2SO_4, filtered, and concentrated under vacuum. The residue was purified by column chromatography (PE/EA=1/1, v/v) to afford **8** as yellow oil (59 mg, 70% yield).

(8) 2,2-dimethyl-3-(3-(3-oxoisoindolin-1-yl)-2-phenyl-1*H*-indol-1yl)propanamide

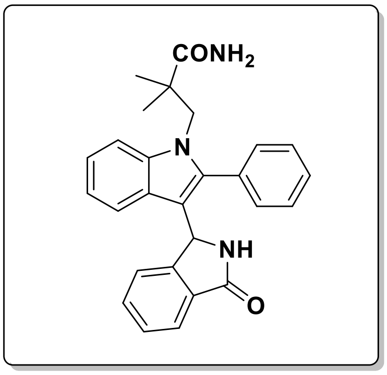

^{1}H NMR (400 MHz, Methanol-*d*$_4$) δ 7.91 – 7.82 (m, 1H), 7.64 – 7.49 (m, 8H), 7.29 – 7.20 (m, 1H), 7.12 – 7.03 (m, 1H), 6.78 (t, *J* = 7.5 Hz, 1H), 6.61 (s, 1H), 5.72 (s, 1H), 4.50 (d, *J* = 2.9 Hz, 2H), 0.90 (d, *J* = 2.1 Hz, 6H). **^{13}C NMR (126 MHz, Methanol-*d*$_4$)** δ 180.15, 148.12, 140.59, 137.43, 131.57, 131.29, 130.92, 130.61, 128.08, 128.00, 127.46, 122.76, 122.28, 121.19, 118.95, 117.80, 110.86, 108.42, 54.18, 50.20, 43.77, 22.94, 22.81. **HRMS (EI) m/z**: calculated for $C_{27}H_{25}N_2O_3$: 423.1941, found: 423.1939.

VI. Mechanistic studies

(1) H/D exchange experiment

(a) H/D exchange experiment of 4aa

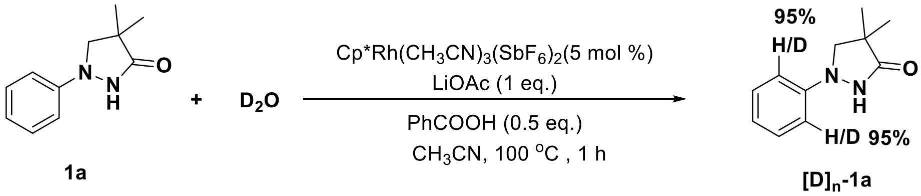

A pressure tube was charged with 4,4-Dimethyl-1-phenylpyrazolidine-3-one (**1a**, 57 mg, 0.3 mmol) and D_2O (0.3 ml), $Cp^*Rh(CH_3CN)_3(SbF_6)_2$ (12.4 mg, 5 mol %), LiOAc (19.8 mg, 0.3mmol), benzoic acid (18.3mg, 0.15 mmol) and MeCN (3 mL). The reaction mixture was stirred at 100 °C for 1 h. After that, the solvent was removed under vacuum and the resulting residue was purified by column chromatography (PE/EA=3/1, v/v) to afford the deuterium product **[Dn]-1a.** The H/D exchange as follows figure.

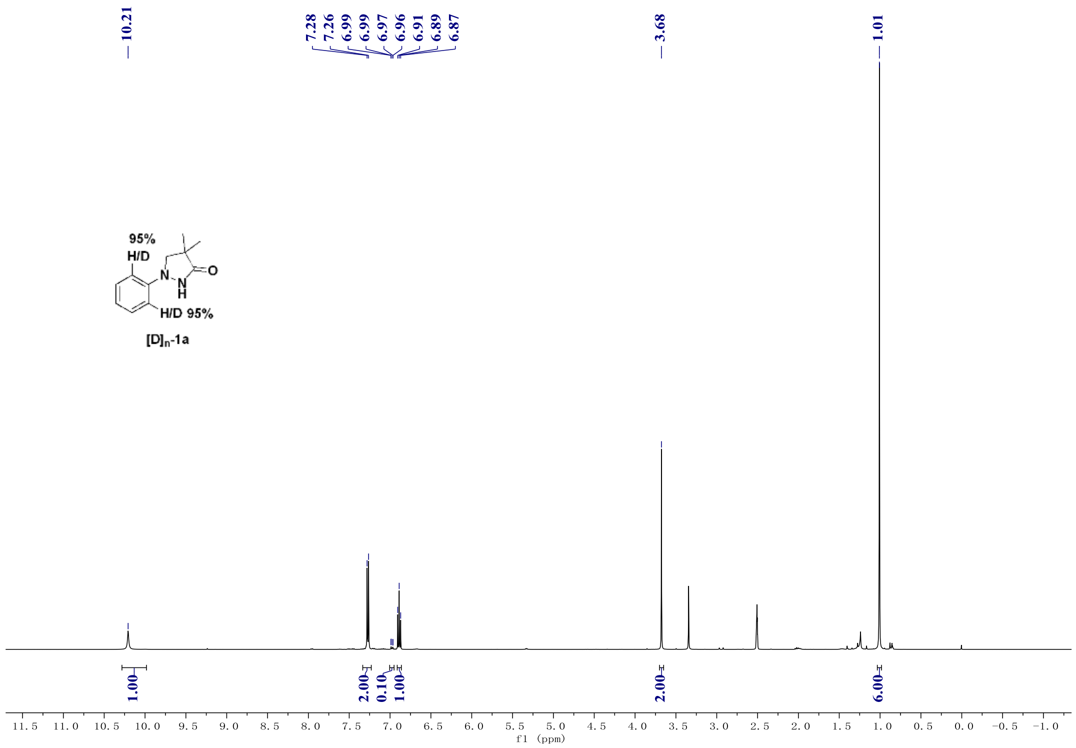

(b) H/D exchange experiment of 5aa

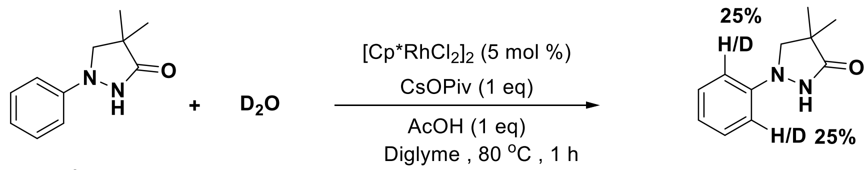

1a

[D]n-1a

A pressure tube was charged with 4,4-dimethyl-1-phenylpyrazolidine-3-one (**1a**, 57 mg, 0.3 mmol) and D_2O (0.3 ml), $[Cp^*RhCl_2]_2$ (9.3 mg, 5 mol %), CsOPiv (70.2 mg, 0.3 mmol), HOAc (7.5 mg, 0.15 mmol) and diethylene glycol dimethyl ether (3 mL). The reaction mixture was stirred at 80 °C for 1h. After that, the solvent was removed under vacuum and the residue was purified by column chromatography (PE/EA=3/1, v/v) to afford the deuterium product **[Dn]-1a.** The H/D exchange as follows figure.

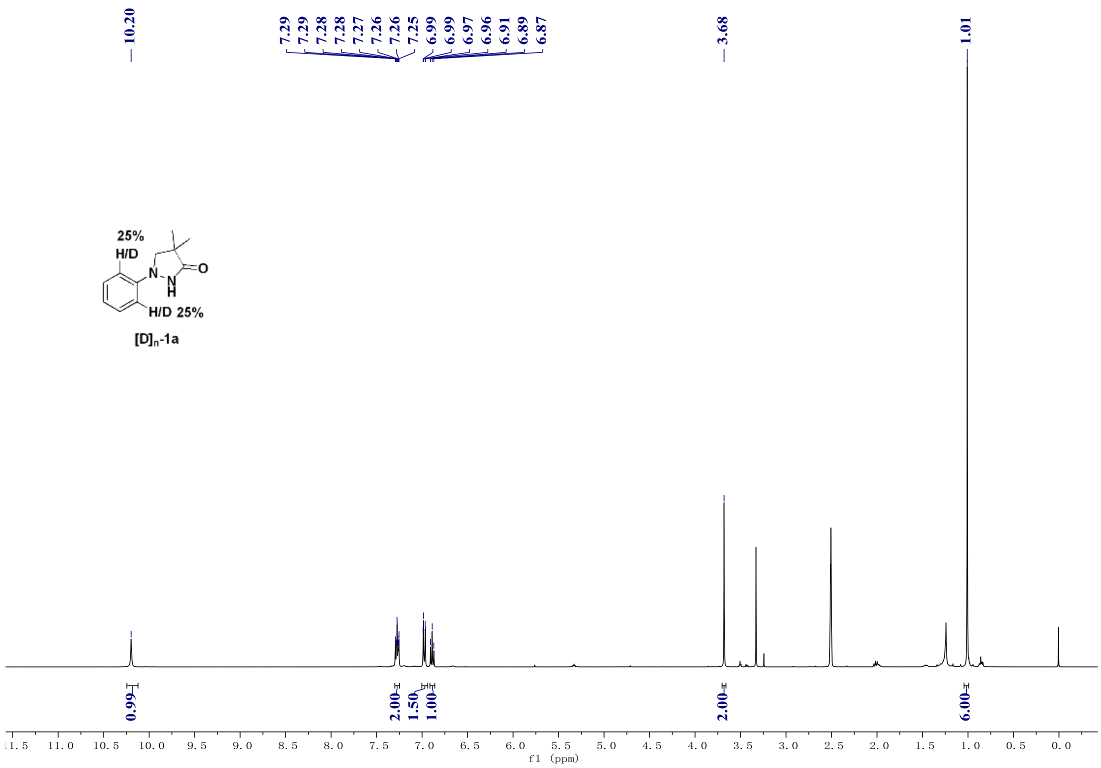

(2) Kinetic isotope effect

(a) KIE Experiments of 4aa

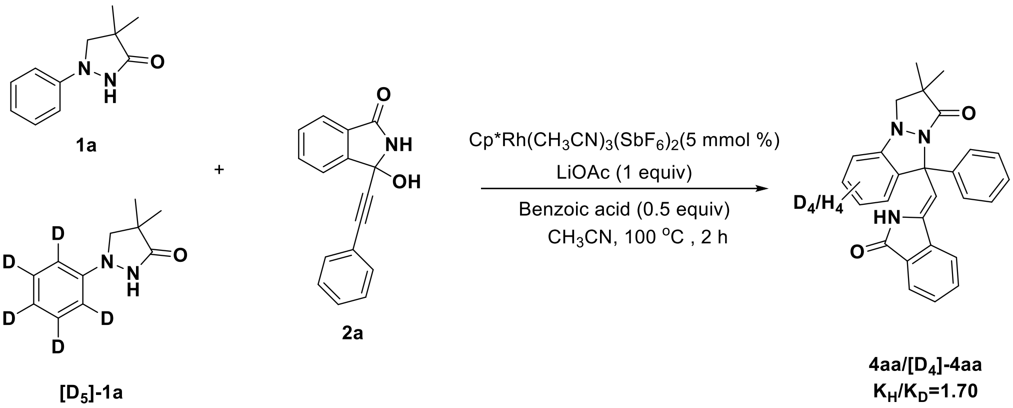

To a reaction tube were added **1a** (38 mg, 0.2 mmol), **[D$_5$]-1a** (37 mg, 0.2 mmol), **2a** (24.9 mg, 0.2 mmol), Cp*Rh(CH_3CN)$_3$(SbF_6)$_2$ (8.4 mg, 5 mol %), LiOAc (23.2 mg, 0.2 mmol) , benzoic acid (12.2 mg, 0.1 mmol) and MeCN (4 mL). The resulting mixture was stirred at 100 °C for 2 h. Upon completion as indicated by TLC, the solvent was removed under vacuum and the residue was purified by column chromatography (PE/EA=1/1, v/v) to obtain a mixture of **4aa** and **[D$_4$]-4aa**. According to the integral

value of characteristic peak in ^{1}H NMR and the ratio of products **4aa** and **[D$_4$]-4aa** was calculated. The ratio of **4aa**/**[D$_4$]-4aa** was 1.70.

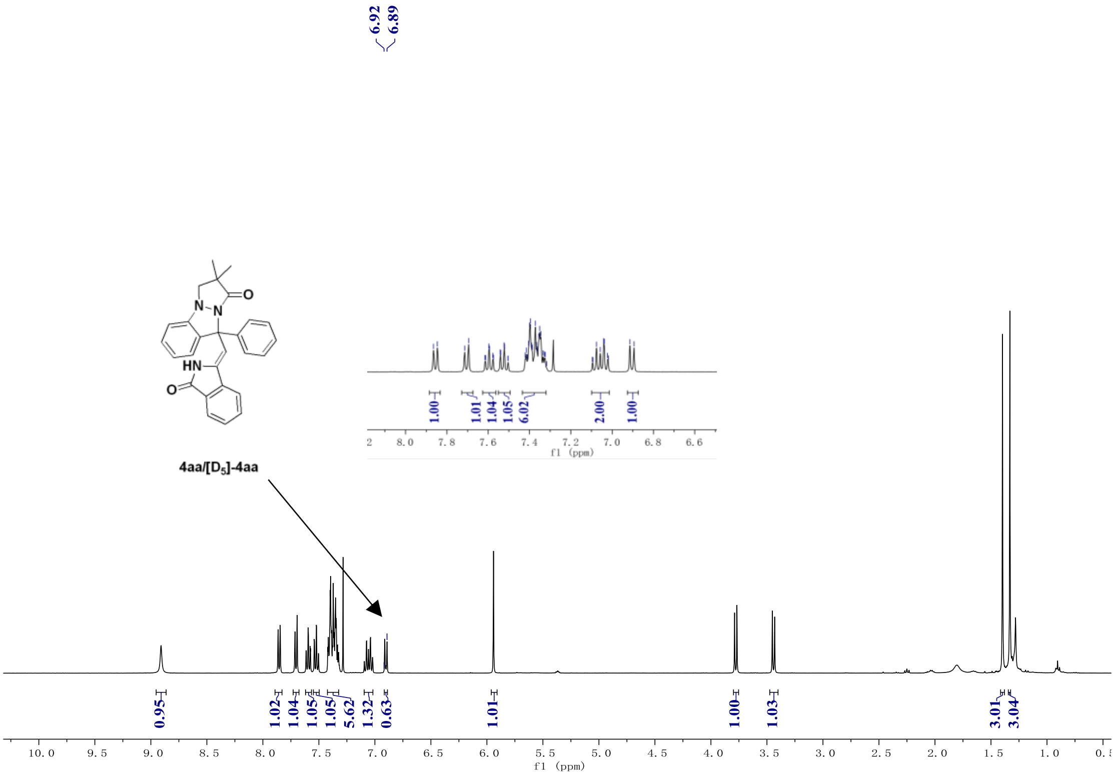

(b) KIE Experiments of 5aa

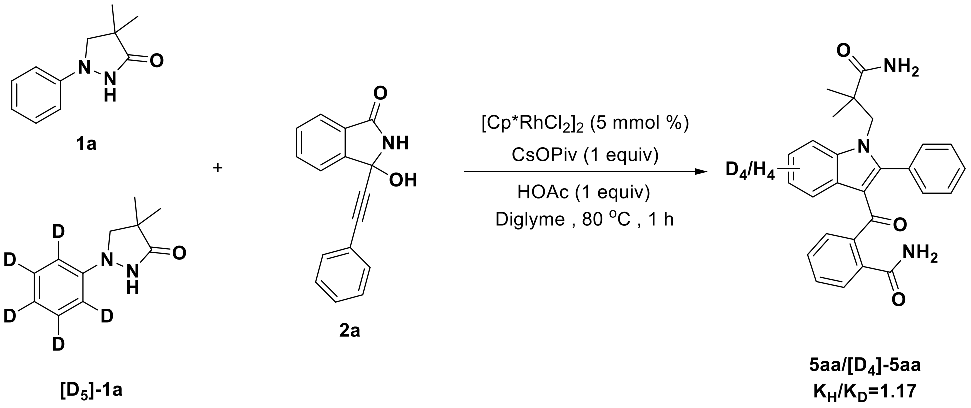

To a reaction tube were added **1a** (38 mg, 0.2 mmol), **[D$_5$]-1a** (37 mg, 0.2 mmol), **2a** (24.9 mg, 0.1 mmol), [Cp*RhCl$_2$]$_2$ (6.28 mg, 5 mol %), CsOPiv (46.8 mg, 0.2 mmol), AcOH (12.0 mg, 0.2 mmol) and diglyme (4 mL). The resulting mixture was stirred at 80 °C for 1 h. Upon completion as indicated by TLC, the solvent was removed under vacuum and the residue was purified by column chromatography (PE/EA = 1/1, v/v) to obtain a mixture of **5aa** and **[D$_4$]-5aa**. According to the integral value of characteristic

peak in ^{1}H NMR and the ratio of products **5aa** and **[D4]-5aa** was calculated. The ratio of **5aa**/ **[D4]-5aa** was 1.17.

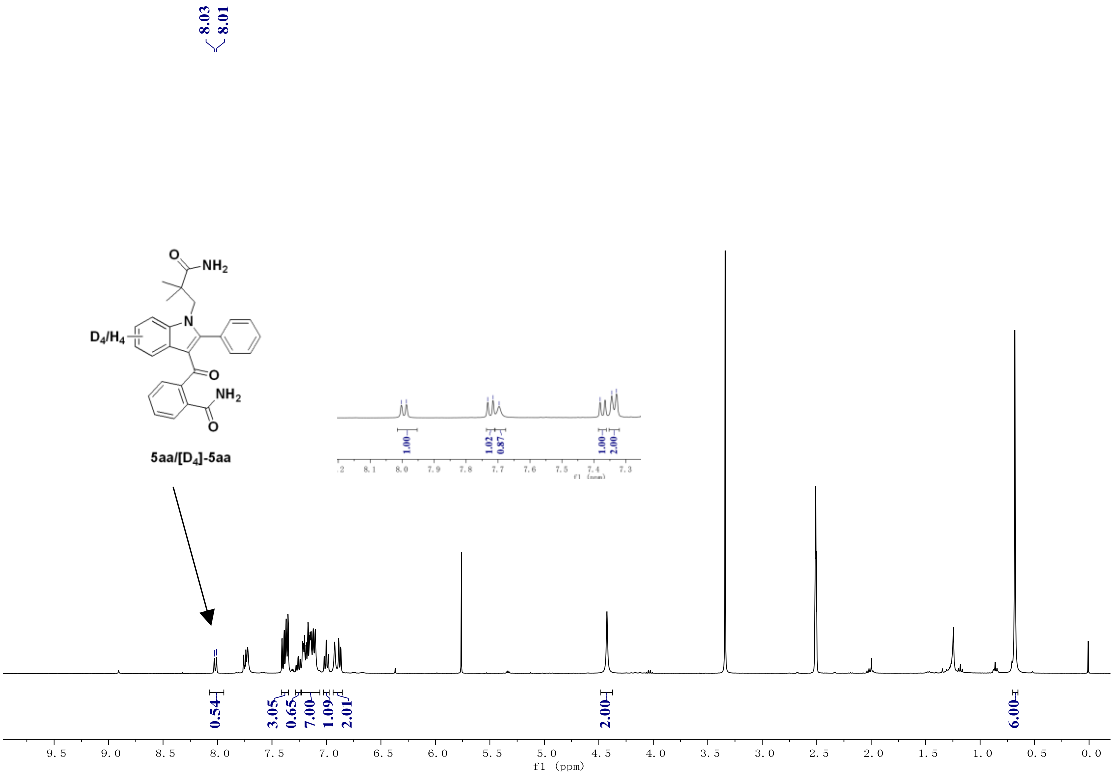

(3) Competition experiment

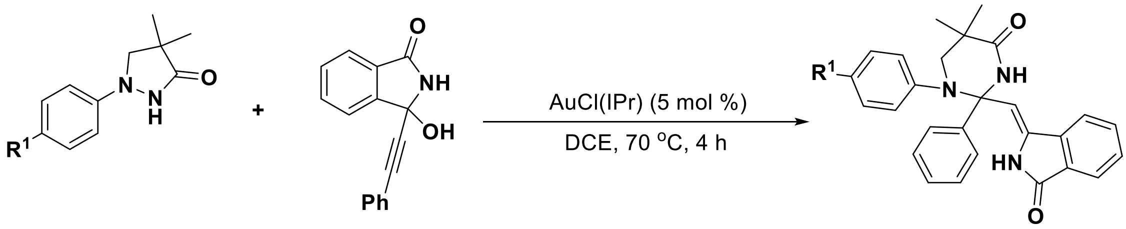

1b, R^1 = F, 0.2 mmol
1f, R^1 = OCH_3, 0.2 mmol

2a, 0.2 mmol

3ba/**3fa**=0.9

To a reaction tube were added **1b** (42 mg, 0.2 mmol), **1f** (44 mg, 0.2 mmol), **2a** (49.8 mg, 0.2 mmol), AuCl(IPr) (6.2 mg, 5 mol%) and DCE (4 mL). The mixture was stirred at 70 °C for 4 h. Upon completion as indicated by TLC,, the solvent was removed under vacuum and the resulting residue was purified by column chromatography (PE/EA=1/1, v/v) to obtain a mixture of **3ba** and **3fa**. The ratio of **3ba**/**3fa** was 0.90 based on ^{1}H NMR.

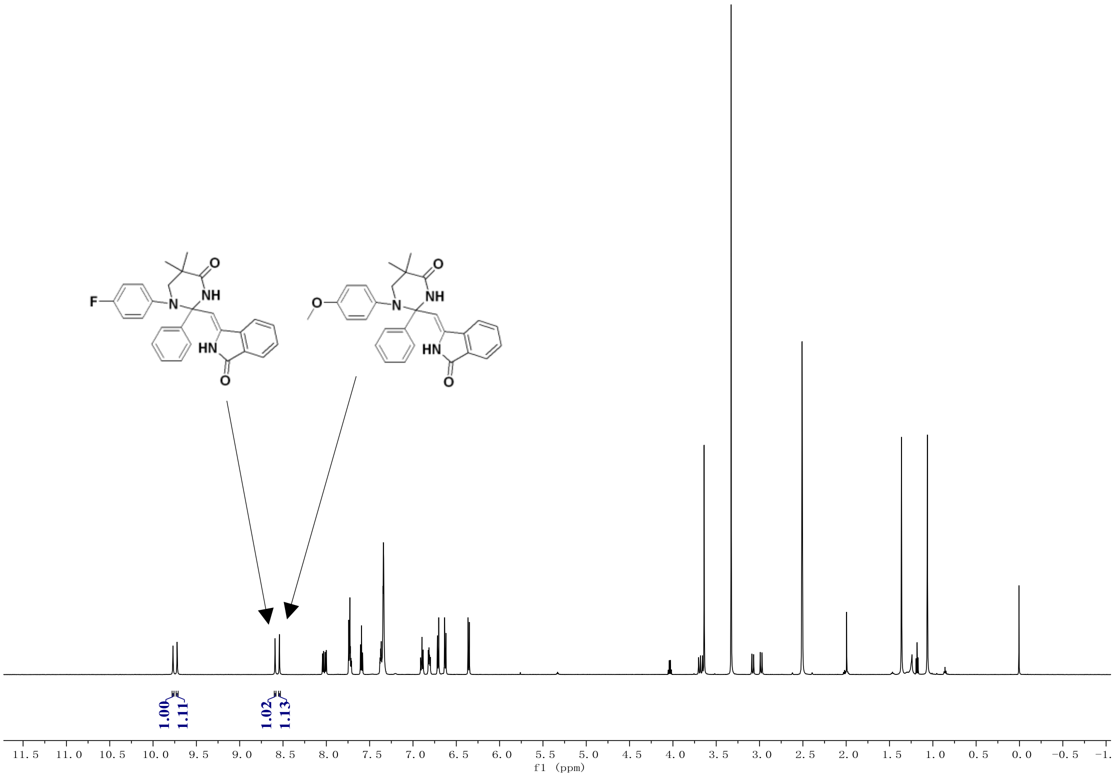

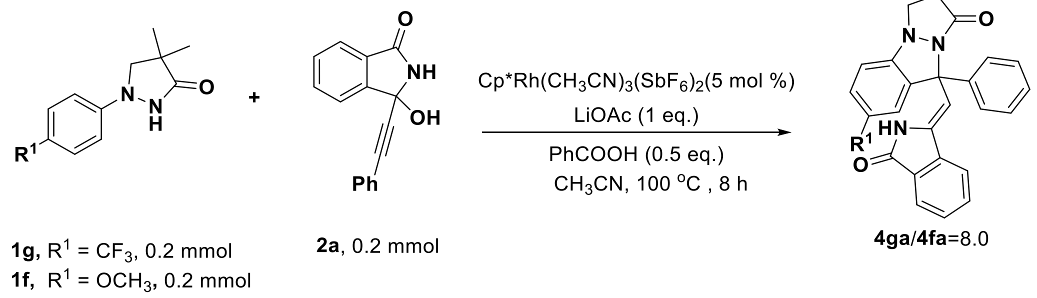

To a reaction tube equipped were added **1g** (52 mg, 0.2 mmol), **1f** (44 mg, 0.2 mmol), **2a** (49.8 mg, 0.2 mmol), $Cp^*Rh(CH_3CN)_3(SbF_6)_2$ (8.39 mg, 5 mol %), LiOAc(13.2 mg, 0.2 mmol), benzoic acid (12.2 mg, 0.1 mmol) and MeCN (4 mL). The reaction mixture was stirred at 100 °C for 8 h. Upon completion as indicated by TLC,, the solvent was removed under vacuum and the residue was purified by column chromatography (PE/EA=1/1, v/v) to obtain a mixture of **4ga** and **4fa**. The ratio of **4ga**/**4fa** was 8.0 based on ^{1}H NMR.

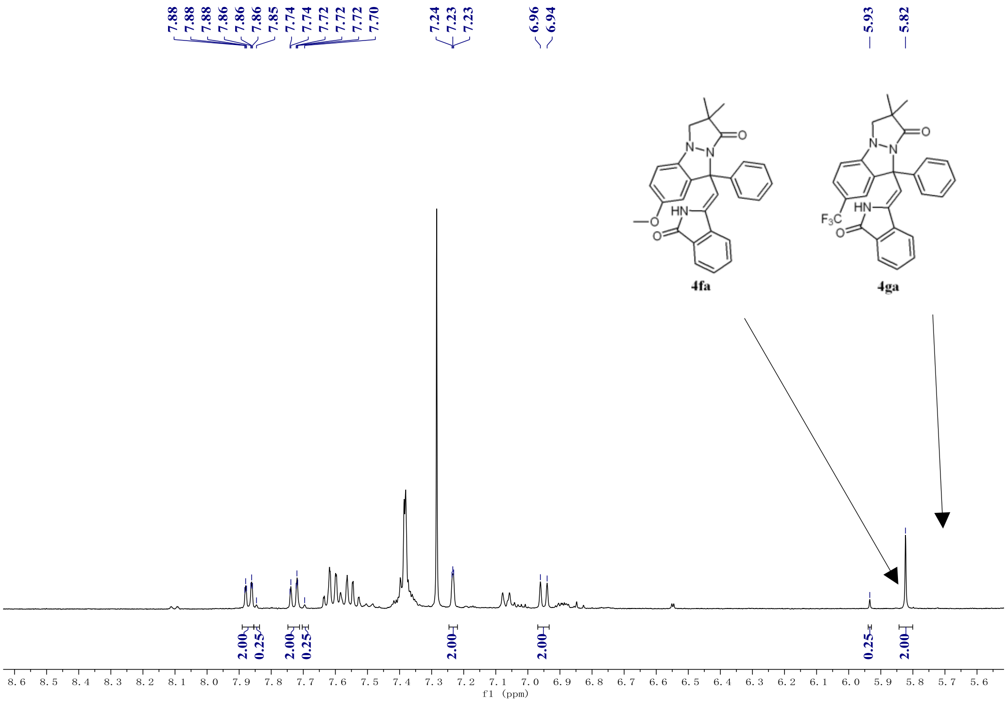

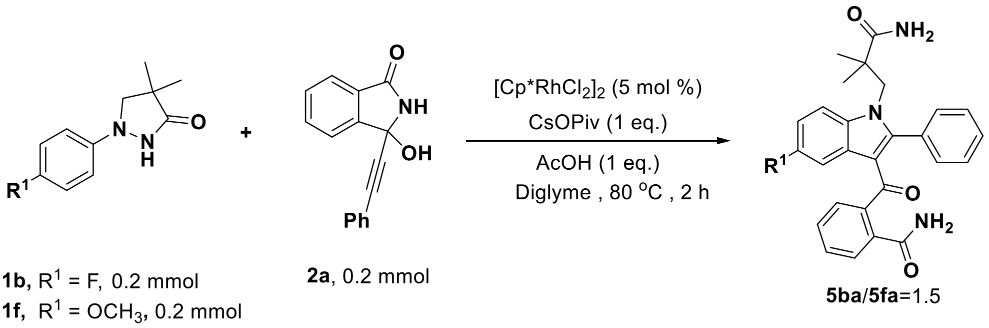

To a reaction tube were added **1b** (42 mg, 0.2 mmol), **1f** (44 mg, 0.2 mmol), **2a** (49.8 mg, 0.2 mmol), Cy-[Cp*RhCl$_2$]$_2$ (6.28 mg, 5 mol %), CsOPiv (46.8 mg, 0.2 mmol), HOAc (12 mg, 0.2 mmol) and diglyme (4 mL). The reaction mixture was stirred at 80 °C (oil bath) for 2 h. Upon completion as indicated by TLC, the solvent was removed under vacuum and the residue was purified by column chromatography (PE/EA=1/1, v/v) to obtain a mixture of **5ba** and **5fa**. The ratio of **5ba**/**5fa** was 1.5 based on ^{1}H NMR.

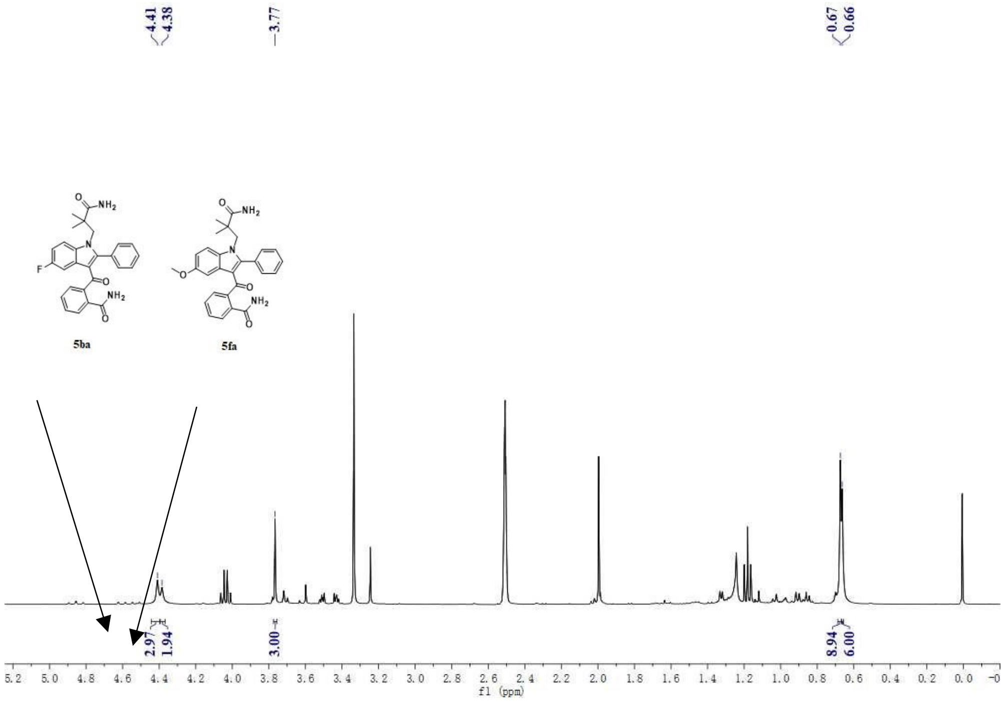

VII. Characterization of the products

(3aa) (*Z/E*)-3-((5,5-dimethyl-4-oxo-1,2-diphenylhexahydropyrimidin-2-yl)methylene)isoindolin-1-one

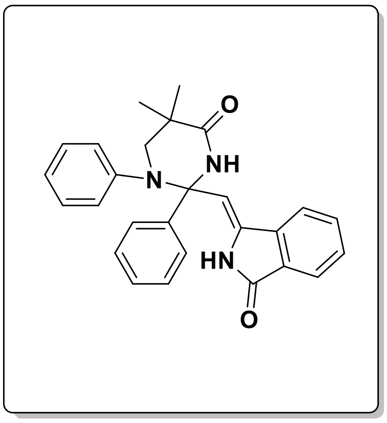

Following by general procedure for the synthesis of **3aa**. White solid, 61 mg, yield: 65%. **^{1}H NMR (500 MHz, DMSO-*d*$_6$)** δ 9.85 (s, 1H), 8.56 (s, 1H), 8.02 – 7.92 (m, 1H), 7.78 – 7.67 (m, 2H), 7.58 (td, *J* = 7.4, 0.7 Hz, 1H), 7.44 – 7.37 (m, 2H), 7.32 (dd, *J* = 5.0, 1.9 Hz, 3H), 7.05 (dd, *J* = 8.5, 7.2 Hz, 2H), 6.89 (t, *J* = 7.3 Hz, 1H), 6.82 – 6.71 (m, 2H), 6.35 (s, 1H), 3.71 (d, *J* = 13.0 Hz, 1H), 3.19 (d, *J* = 13.0 Hz, 1H), 1.38 (s, 3H), 1.05 (s, 3H). **^{13}C{^{1}H} NMR (126 MHz, DMSO-*d*$_6$)** δ 174.9, 168.2, 147.5, 142.0, 136.1, 132.5, 129.7, 128.6, 128.2, 128.2, 128.0, 128.0, 124.8, 123.0, 122.7, 120.6, 106.2, 77.6, 58.9, 25.9, 23.4. **HRMS (ESI) m/z**: calculated for $C_{27}H_{24}N_3O_2$ [M- H]$^-$: 422.1874, found:422.1879.

(3ba) **(*Z/E*)-3-((1-(4-fluorophenyl)-5,5-dimethyl-4-oxo-2-phenylhexahydropyrimidin-2-yl) methylene)isoindolin-1-one**

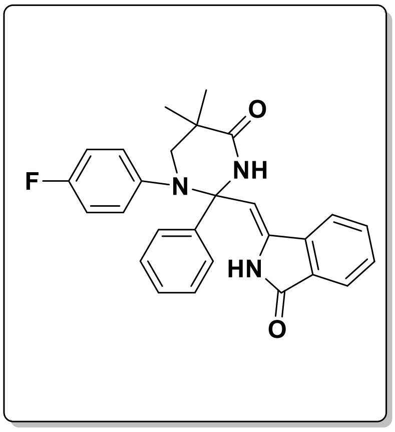

Following by general procedure for the synthesis of **3aa**. White solid, 56 mg, yield: 64%. **^{1}H NMR (400 MHz, DMSO-*d*$_6$)** δ 9.80 (s, 1H), 8.60 (s, 1H), 8.04 (d, J = 7.8 Hz, 1H), 7.72 (t, J = 7.7 Hz, 2H), 7.59 (t, J = 7.4 Hz, 1H), 7.40 – 7.30 (m, 5H), 6.94 – 6.85 (m, 2H), 6.85 – 6.77 (m, 2H), 6.36 (s, 1H), 3.70 (d, J = 12.8 Hz, 1H), 3.08 (d, J = 12.9 Hz, 1H), 1.36 (s, 3H), 1.06 (s, 3H). **^{13}C{^{1}H} NMR (126 MHz, DMSO-*d*$_6$)** δ 175.0, 175.0, 168.2, 168.0, 158.5 (d, J = 240.9 Hz), 143.8, 141.8, 141.8, 137.7, 137.6, 136.0, 135.9, 132.5, 129.7, 128.8, 128.3, 128.2, 128.0, 127.4 (d, J = 8.1 Hz), 122.7, 120.7, 114.6 (d, J = 22.1 Hz), 105.9, 105.8, 77.6, 77.5, 59.0, 26.0, 23.4, 23.4. **HRMS (ESI) m/z**: calculated for $C_{27}H_{23}FN_3O_2$ $[M- H]^-$:440.1780, found: 440.1783.

(3ca) **(*Z/E*)-3-((1-(4-chlorophenyl)-5,5-dimethyl-4-oxo-2-phenylhexahydropyrimidin-2-yl)methylene)isoindolin-1-one**

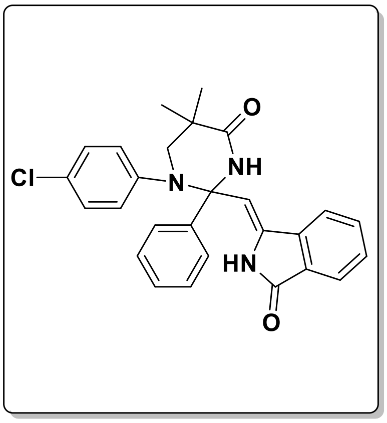

Following by general procedure for the synthesis of **3aa**. White solid, 42 mg, yield: 46%.**^{1}H NMR (400 MHz, DMSO-*d*$_6$)** δ 9.88 (s, 1H), 8.60 (s, 1H), 8.03 (d, J = 7.7 Hz, 1H), 7.77 – 7.67 (m, 2H), 7.59 (t, J = 7.4 Hz, 1H), 7.45 – 7.37 (m, 2H), 7.38 – 7.31 (m, 3H), 7.14 – 7.07 (m, 2H), 6.86 – 6.75 (m, 2H), 6.32 (s, 1H), 3.71 (d, J = 13.1 Hz, 1H), 3.20 (d, J = 13.1 Hz, 1H), 1.37 (s, 3H), 1.05 (s, 3H). **^{13}C{^{1}H} NMR (151 MHz, DMSO-*d*$_6$)** δ 175.3, 168.7, 146.8, 142.2, 138.1, 136.6, 133.0, 130.2, 129.2, 128.8, 128.5, 128.4, 128.3, 127.4, 126.8, 123.1, 121.2, 106.4, 78.0, 59.3, 26.3, 23.8.**HRMS (ESI) m/z**: calculated for $C_{27}H_{23}ClN_3O_2$ $[M- H]^-$:456.1484, found: 456.1487.

(3da) (*Z/E*)-3-((1-(4-bromophenyl)-5,5-dimethyl-4-oxo-2-phenylhexahydropyrimidin-2-yl)methylene)isoindolin-1-one

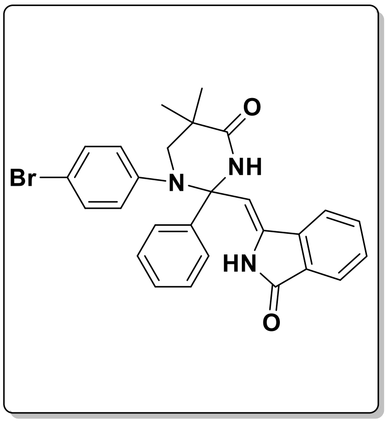

Following by general procedure for the synthesis of **3aa**. White solid, 36 mg, yield: 36%. **^{1}H NMR (400 MHz, DMSO-*d*$_6$)** δ 9.89 (s, 1H), 8.60 (s, 1H), 8.03 (d, *J* = 7.7 Hz, 1H), 7.78 – 7.67 (m, 2H), 7.59 (t, *J* = 7.4 Hz, 1H), 7.46 – 7.29 (m, 5H), 7.25 – 7.17 (m, 2H), 6.79 – 6.71 (m, 2H), 6.32 (s, 1H), 3.71 (d, *J* = 13.1 Hz, 1H), 3.21 (d, *J* = 13.1 Hz, 1H), 1.37 (s, 3H), 1.05 (s, 3H). **^{13}C{^{1}H} NMR (151 MHz, DMSO-*d*$_6$)** δ 175.2, 168.7, 147.3, 142.2, 138.1, 136.7, 133.0, 131.2, 130.2, 129.2, 128.8, 128.5, 128.4, 127.0, 123.1, 121.3, 115.4, 106.4, 77.9, 59.3, 26.3, 23.8. **HRMS (ESI) m/z**: calculated for $C_{27}H_{23}BrN_3O_2$ [M- H]$^-$:500.0979, found:500.0981.

(3ea) (*Z/E*)-3-((5,5-dimethyl-4-oxo-2-phenyl-1-(*p*-tolyl)hexahydropyrimidin-2-yl)methylene)isoindolin-1-one

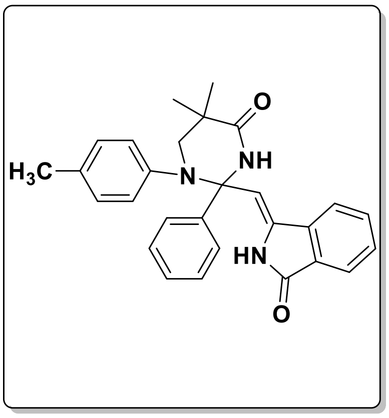

Following by general procedure for the synthesis of **3aa**. White solid, 52 mg, yield: 60%. **^{1}H NMR (400 MHz, DMSO-*d*$_6$)** δ 9.83 (s, 1H), 8.56 (s, 1H), 7.98 (d, *J* = 7.7 Hz, 1H), 7.77 – 7.68 (m, 2H), 7.59 (t, *J* = 7.4 Hz, 1H), 7.42 – 7.29 (m, 5H), 6.86 (d, *J* = 8.1 Hz, 2H), 6.66 (d, *J* = 8.1 Hz, 2H), 6.34 (s, 1H), 3.68 (d, *J* = 12.9 Hz, 1H), 3.09 (d, *J* = 12.8 Hz, 1H), 2.14 (s, 3H), 1.37 (s, 3H), 1.05 (s, 3H). **^{13}C{^{1}H} NMR (126 MHz, DMSO-*d*$_6$)** δ 175.0, 168.2, 144.9, 142.11, 137.7, 136.0, 136.0, 132.5, 132.3, 129.7, 128.6, 128.5, 128.3, 128.1, 128.0, 125.2, 122.7, 120.6, 106.1, 77.6, 77.5, 59.0, 25.9, 23.4, 23.4, 20.2. **HRMS (ESI) m/z**: calculated for $C_{28}H_{26}N_3O_2$ [M- H]$^-$:436.2031, found:436.2035.

(3fa) (*Z/E*)-3-((1-(4-methoxyphenyl)-5,5-dimethyl-4-oxo-2-phenylhexahydropyrimidin-2-yl)methylene)isoindolin-1-one

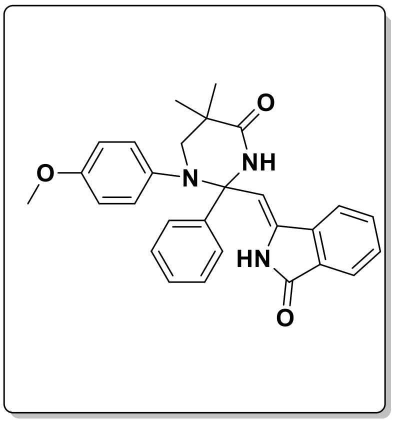

Following by general procedure for the synthesis of **3aa**. White solid, 36 mg, yield: 41%. **^{1}H NMR (600 MHz, DMSO-*d*$_6$)** δ 9.71 (s, 1H), 8.54 (s, 1H), 8.00 (d, *J* = 7.7 Hz, 1H), 7.73 (t, *J* = 7.8 Hz, 2H), 7.59 (t, *J* = 7.4 Hz, 1H), 7.34 (d, *J* = 2.1 Hz, 5H), 6.71 (d, *J* = 8.9 Hz, 2H), 6.63 (d, *J* = 8.9 Hz, 2H), 6.36 (s, 1H), 3.66 (s, 1H), 3.64 (s, 3H), 2.98 (d, *J* = 12.6 Hz, 1H), 1.36 (s, 3H), 1.06 (s, 3H). **^{13}C NMR (151 MHz, DMSO-*d*$_6$)** δ 175.0, 168.1, 155.7, 142.0, 140.3, 137.7, 135.8, 132.5, 129.6, 128.6, 128.3, 128.0, 127.2, 122.7, 120.6, 113.1, 106.0, 77.7, 59.0, 55.0, 26.0, 23.5. **HRMS (ESI) m/z**: calculated for $C_{28}H_{26}N_3O_3$ [M- H]$^-$:452.1980, found:452.1979. HPLC purity: 92.4%; t_R = 2.728 min, MeOH-H_2O (80:20).

(3ia) (*Z/E*)-3-((1-(3-fluorophenyl)-5,5-dimethyl-4-oxo-2-phenylhexahydropyrimidin-2-yl)methylene)isoindolin-1-one

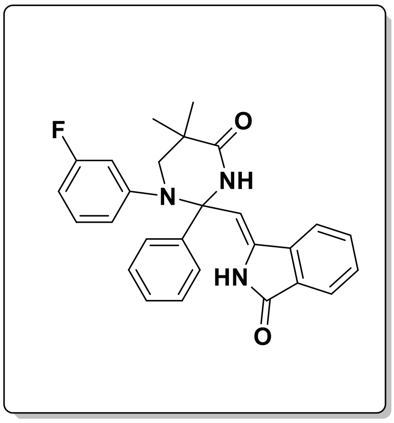

Following by general procedure for the synthesis of **3aa**. White solid, 22 mg, yield: 25%. **^{1}H NMR (400 MHz, DMSO-*d*$_6$)** δ 9.95 (s, 1H), 8.60 (s, 1H), 8.04 (d, *J* = 7.7 Hz, 1H), 7.78 – 7.68 (m, 2H), 7.60 (t, *J* = 7.4 Hz, 1H), 7.48 – 7.40 (m, 2H), 7.40 – 7.31 (m, 3H), 7.09 (q, *J* = 7.9 Hz, 1H), 6.74 – 6.62 (m, 2H), 6.51 (dt, *J* = 12.1, 2.4 Hz, 1H), 6.33 (s, 1H), 3.74 (d, *J* = 13.3 Hz, 1H), 3.31 (d, *J* = 16.2 Hz, 3H), 1.38 (s, 3H), 1.06 (s, 3H). **^{13}C{^{1}H} NMR (126 MHz, DMSO-*d*$_6$)** δ 174.7, 174.7, 168.3, 161.7 (d, *J* = 241.9 Hz), 149.4 (d, *J* = 9.6 Hz), 141.7, 137.7, 136.3, 132.5, 129.8, 129.3 (d, *J* = 9.8 Hz), 128.8, 128.4, 128.0, 122.7, 120.8, 119.9, 111.0 (d, *J* = 23.6 Hz), 109.1 (d, *J* = 20.9 Hz),

106.0, 77.5, 77.5, 58.7, 25.7, 23.2, 23.2. **HRMS (ESI) m/z**: calculated for $C_{27}H_{23}FN_3O_2$ $[M- H]^-$:440.1780, found:440.1783.

(3ab) (*Z/E*)-3-((2-(4-fluorophenyl)-5,5-dimethyl-4-oxo-1-phenylhexahydropyrimidin-2-yl)methylene)isoindolin-1-one

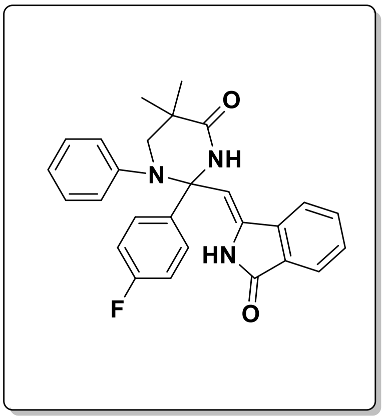

Following by general procedure for the synthesis of **3aa**. White solid, 51 mg, yield: 58%. **^{1}H NMR (400 MHz, DMSO-*d*$_6$)** δ 9.89 (s, 1H), 8.58 (s, 1H), 8.02 (d, *J* = 7.7 Hz, 1H), 7.80 – 7.68 (m, 2H), 7.59 (t, *J* = 7.5 Hz, 1H), 7.42 (t, *J* = 6.9 Hz, 2H), 7.12 (dt, *J* = 22.8, 8.1 Hz, 4H), 6.92 (t, *J* = 7.1 Hz, 1H), 6.80 (d, *J* = 7.9 Hz, 2H), 6.39 (s, 1H), 3.74 (d, *J* = 13.0 Hz, 1H), 3.18 (d, *J* = 13.3 Hz, 1H), 1.40 (s, 3H), 1.06 (s, 3H). **^{13}C{^{1}H} NMR (126 MHz, DMSO-*d*$_6$)** δ 175.3, 175.3, 168.7, f168.6, 162.4 (d, *J* = 245.3 Hz), 147.8, 138.6, 138.1, 136.8, 133.0, 131.0 (d, *J* = 8.6 Hz), 130.2, 128.6, 128.5, 125.5, 123.6, 123.2, 121.2, 115.4 (d, *J* = 21.5 Hz), 106.3, 77.5, 77.5, 59.4, 26.3, 23.7. **HRMS (ESI) m/z**: calculated for $C_{27}H_{23}FN_3O_2$ $[M- H]^-$:440.1780, found:440.1782.

(3ac) (*Z/E*)-3-((2-(4-chlorophenyl)-5,5-dimethyl-4-oxo-1-phenylhexahydropyrimidin-2-yl)methylene)isoindolin-1-one

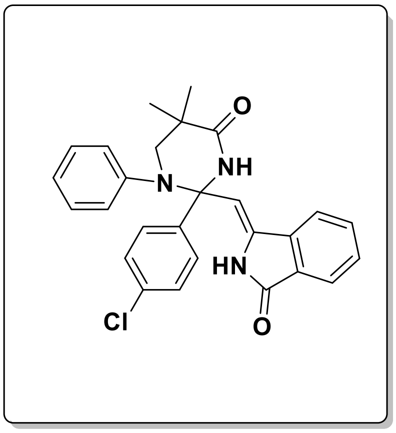

Following by general procedure for the synthesis of **3aa**. White solid, 63 mg, yield: 70%. **^{1}H NMR (400 MHz, DMSO-*d*$_6$)** δ 9.88 (s, 1H), 8.58 (s, 1H), 8.03 (d, *J* = 7.7 Hz, 1H), 7.80 – 7.69 (m, 2H), 7.60 (t, *J* = 7.4 Hz, 1H), 7.40 (s, 4H), 7.17 – 7.05 (m, 2H), 6.92 (t, *J* = 7.3 Hz, 1H), 6.87 – 6.78 (m, 2H), 6.38 (s, 1H), 3.73 (d, *J* = 13.1 Hz, 1H), 3.20 (d, *J* = 13.1 Hz, 1H), 1.39 (s, 3H), 1.06 (s, 3H). **^{13}C{^{1}H} NMR (126 MHz, DMSO-*d*$_6$)** δ 175.3, 168.7, 147.7, 141.4, 141.3, 138.0, 136.8, 133.6, 133.0, 130.6, 130.2,

128.7, 128.6, 128.4, 125.4, 123.6, 123.2, 121.2, 106.2, 77.5, 77.4, 59.4, 26.3, 23.7, 23.7. **HRMS (ESI) m/z**: calculated for $C_{27}H_{23}ClN_3O_2$ [M- H]$^-$:456.1484, found:456.1489.

(3ad) (*Z/E*)-3-((2-(4-bromophenyl)-5,5-dimethyl-4-oxo-1-phenylhexahydropyrimidin-2-yl)methylene)isoindolin-1-one

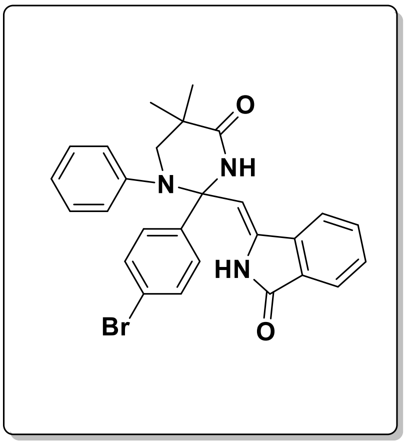

Following by general procedure for the synthesis of **3aa**. White solid, 72 mg, yield: 72%. **^{1}H NMR (400 MHz, DMSO-*d*$_6$)** δ 9.88 (s, 1H), 8.57 (s, 1H), 8.03 (d, J = 7.8 Hz, 1H), 7.77 – 7.67 (m, 2H), 7.64 – 7.49 (m, 3H), 7.34 (d, J = 8.4 Hz, 2H), 7.10 (t, J = 7.7 Hz, 2H), 6.96 – 6.76 (m, 3H), 6.38 (s, 1H), 3.73 (d, J = 13.1 Hz, 1H), 3.20 (d, J = 13.0 Hz, 1H), 1.39 (s, 3H), 1.06 (s, 3H). **^{13}C{^{1}H} NMR (151 MHz, DMSO-*d*$_6$)** δ 175.3, 175.2, 168.7, 147.7, 141.8, 141.8, 138.1, 133.0, 131.6, 131.0, 130.3, 128.7, 128.4, 125.3, 123.6, 123.2, 122.3, 121.2, 106.1, 77.6, 77.5, 59.5, 26.3, 26.3, 23.7, 23.7.**HRMS (ESI) m/z**: calculated for $C_{27}H_{23}BrN_3O_2$ [M- H]$^-$:500.0979, found:500.0984.

(3ae) (*Z/E*)-3-((5,5-dimethyl-4-oxo-1-phenyl-2-(p-tolyl)hexahydropyrimidin-2-yl)methylene)isoindolin-1-one

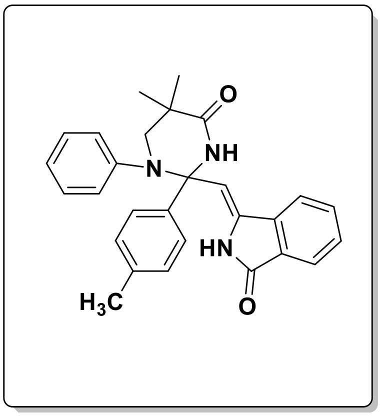

Following by general procedure for the synthesis of **3aa**. White solid, 46 mg, yield: 53%. **^{1}H NMR (400 MHz, DMSO-*d*$_6$)** δ 9.85 (s, 1H), 8.53 (s, 1H), 7.99 (d, J = 7.8 Hz, 1H), 7.77 – 7.67 (m, 2H), 7.59 (t, J = 7.4 Hz, 1H), 7.27 (d, J = 8.1 Hz, 2H), 7.13 (d, J = 8.0 Hz, 2H), 7.07 (t, J = 7.7 Hz, 2H), 6.89 (t, J = 7.3 Hz, 1H), 6.81 (d, J = 8.0 Hz, 2H), 6.35 (s, 1H), 3.70 (d, J = 13.0 Hz, 1H), 3.19 (d, J = 13.0 Hz, 1H), 2.29 (s, 3H), 1.37 (s, 3H), 1.05 (s, 3H). **^{13}C{^{1}H} NMR (126 MHz, DMSO-*d*$_6$)** δ 175.3, 148.0, 138.3, 133.0,

130.1, 129.2, 128.5, 128.4, 125.2, 123.3, 123.2, 121.1, 107.0, 77.8, 77.8, 59.4, 26.3, 26.3, 23.8, 23.8, 21.2. **HRMS (ESI) m/z**: calculated for $C_{28}H_{26}N_3O_2$ [M- H]$^-$:436.2031, found:436.2034.

(3af) (*Z/E*)-3-((2-(4-methoxyphenyl)-5,5-dimethyl-4-oxo-1-phenylhexahydropyrimidin-2-yl)methylene)isoindolin-1-one

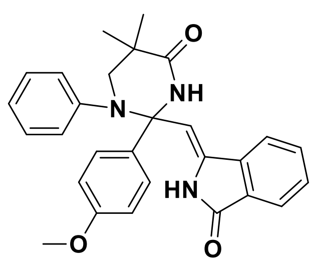

Following by general procedure for the synthesis of **3aa**. White solid, 51 mg, yield: 56%. **^{1}H NMR (600 MHz, Acetic acid -*d*$_4$)** δ 7.89 (d, *J* = 7.6 Hz, 1H), 7.85 (d, *J* = 7.8 Hz, 1H), 7.73 – 7.68 (m, 1H), 7.60 (t, *J* = 7.4 Hz, 1H), 7.48 – 7.43 (m, 2H), 7.20 (dd, *J* = 8.7, 7.1 Hz, 2H), 7.07 (d, *J* = 7.8 Hz, 3H), 6.99 – 6.95 (m, 2H), 6.28 (s, 1H), 3.85 (s, 3H), 3.57 (d, *J* = 13.3 Hz, 1H), 3.36 (d, *J* = 13.2 Hz, 1H), 1.39 (s, 3H), 1.28 (s, 3H).**^{13}C{^{1}H} NMR (151 MHz, Acetic acid -*d*$_4$)** δ 179.9, 170.2, 160.1, 147.5, 137.8, 134.7, 133.0, 132.9, 129.8, 128.9, 128.3, 128.0, 125.8, 124.3, 123.6, 120.0, 113.7, 107.9, 78.4, 58.6, 54.6, 39.2, 24.5, 24.1.**HRMS (ESI) m/z**: calculated for $C_{28}H_{26}N_3O_3$ [M-H]$^-$:452.1980, found:452.1981.

(3ag) (*Z/E*)-3-((5,5-dimethyl-4-oxo-2-(4-pentylphenyl)-1-phenylhexahydropyrimidin-2-yl)methylene)isoindolin-1-one

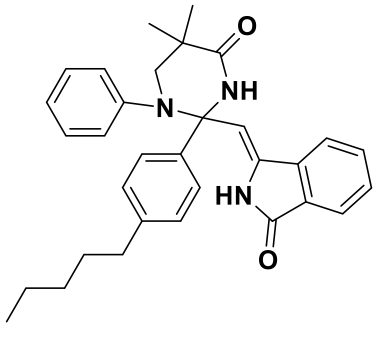

Following by general procedure for the synthesis of **3aa**. White solid, 48 mg, yield: 49%. **^{1}H NMR (400 MHz, DMSO-*d*$_6$)** δ 9.86 (s, 1H), 8.54 (s, 1H), 7.98 (d, *J* = 7.8 Hz, 1H), 7.76 – 7.69 (m, 2H), 7.59 (t, *J* = 7.4 Hz, 1H), 7.28 (d, *J* = 8.1 Hz, 2H), 7.14 (d, *J* = 8.1 Hz, 2H), 7.04 (t, *J* = 7.8 Hz, 2H), 6.89 (t, *J* = 7.3 Hz, 1H), 6.76 (d, *J* = 8.0 Hz, 2H), 6.34 (s, 1H), 3.71 (d, *J* = 13.0 Hz, 1H), 3.17 (d, *J* = 12.9 Hz, 1H), 2.59

– 2.52 (m, 2H), 1.55 (q, J = 7.4 Hz, 2H), 1.37 (s, 3H), 1.29 – 1.21 (m, 4H), 1.05 (s, 3H), 0.85 (t, J = 7.1 Hz, 3H). **$^{13}C\{^1H\}$ NMR (151 MHz, DMSO-*d*6)** δ 175.3, 168.6, 148.0, 143.2, 138.2, 133.0, 130.1, 128.6, 128.5, 128.5, 128.4, 125.3, 123.4, 123.2, 121.1, 106.8, 106.8, 77.9, 59.3, 35.1, 31.2, 31.0, 26.3, 23.8, 23.8, 22.4, 14.4. **HRMS (ESI) m/z**: calculated for $C_{32}H_{34}N_3O_2$ $[M-H]^-$:492.2657, found:492.2657.

(3ai) (*Z/E*)-3-((5,5-dimethyl-4-oxo-1-phenyl-2-(m-tolyl)hexahydropyrimidin-2-yl)methylene)isoindolin-1-one

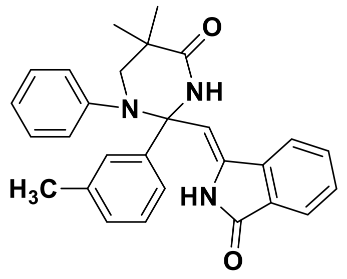

Following by general procedure for the synthesis of **3aa**. White solid, 60 mg, yield: 69%. **^1H NMR (400 MHz, DMSO-*d*6)** δ 9.81 (s, 1H), 8.55 (s, 1H), 7.98 (d, J = 7.7 Hz, 1H), 7.76 – 7.67 (m, 2H), 7.59 (t, J = 7.4 Hz, 1H), 7.26 – 7.12 (m, 4H), 7.07 (t, J = 7.7 Hz, 2H), 6.90 (t, J = 7.3 Hz, 1H), 6.81 (d, J = 8.0 Hz, 2H), 6.32 (s, 1H), 3.68 (d, J = 13.0 Hz, 1H), 3.18 (d, J = 13.0 Hz, 1H), 2.26 (s, 3H), 1.36 (s, 3H), 1.05 (s, 3H). **$^{13}C\{^1H\}$ NMR (126 MHz, DMSO-*d*6)** δ 168.1, 147.5, 142.1, 137.2, 132.5, 129.7, 129.2, 128.8, 128.5, 128.0, 125.4, 124.9, 123.0, 122.7, 120.6, 112.3, 106.4, 106.3, 77.6, 77.5, 58.9, 25.9, 23.5, 23.5, 21.2. **HRMS (ESI) m/z**: calculated for $C_{28}H_{26}N_3O_2$ $[M-H]^-$:436.2031, found:436.2032. HPLC purity: 95.1%; t_R = 2.568 min, MeOH-H_2O (80:20).

(3ak) (*Z/E*)-3-((2-(3-chlorophenyl)-5,5-dimethyl-4-oxo-1-phenylhexahydropyrimidin-2-yl)methylene)isoindolin-1-one

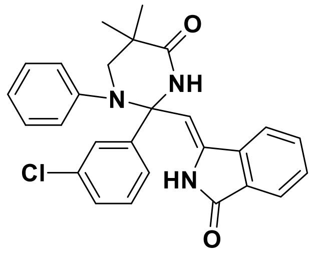

Following by general procedure for the synthesis of **3aa**. White solid, 65 mg, yield: 72%. **^1H NMR (600 MHz, DMSO-*d*6)** δ 9.80 (s, 1H), 8.63 (s, 1H), 8.02 (d, J = 7.7 Hz, 1H), 7.77 – 7.68 (m, 2H), 7.59 (t, J = 7.4 Hz, 1H), 7.45 – 7.32 (m, 4H), 7.11 (t, J = 7.7 Hz, 2H), 6.94 (t, J = 7.3 Hz, 1H), 6.87 (d, J = 8.0 Hz, 2H), 6.36 (s, 1H), 3.70 (d, J = 13.1 Hz, 1H), 3.20 (d, J = 13.1 Hz,

1H), 1.35 (s, 3H), 1.05 (s, 3H). **$^{13}C\{^1H\}$ NMR (151 MHz, DMSO-*d_6*)** δ 175.43, 168.63, 147.63, 144.97, 138.09, 136.56, 133.29, 133.04, 130.55, 130.27, 129.03, 128.65, 128.41, 128.21, 127.37, 125.43, 123.79, 123.19, 121.24, 106.07, 77.60, 59.44, 26.28, 23.96. **HRMS (ESI) m/z**: calculated for $C_{27}H_{25}ClN_3O_2$ $[M+H]^+$:458.1630, found: 458.1634.

(3al) (*Z/E*)-3-((5,5-dimethyl-4-oxo-1-phenyl-2-(thiophen-3-yl)hexahydropyrimidin-2-yl)methylene)isoindolin-1-one

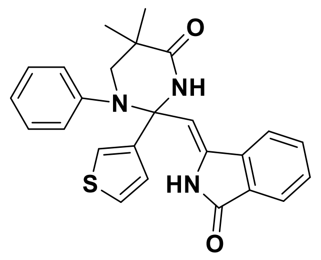

Following by general procedure for the synthesis of **3aa**. White solid, 56 mg, yield: 66%. **^1H NMR (400 MHz, DMSO-*d_6*)** δ 9.79 (s, 1H), 8.62 (s, 1H), 7.94 (d, *J* = 7.7 Hz, 1H), 7.77 – 7.68 (m, 2H), 7.61 – 7.51 (m, 2H), 7.25 (d, *J* = 2.8 Hz, 1H), 7.11 (t, *J* = 7.3 Hz, 3H), 6.94 (t, *J* = 7.3 Hz, 1H), 6.86 (d, *J* = 8.0 Hz, 2H), 6.30 (s, 1H), 3.64 (d, *J* = 12.9 Hz, 1H), 3.16 (d, *J* = 12.9 Hz, 1H), 1.33 (s, 3H), 1.05 (s, 3H). **$^{13}C\{^1H\}$ NMR (151 MHz, DMSO-*d_6*)** δ 175.3, 175.3, 168.5, 148.1, 144.1, 144.0, 138.3, 132.9, 130.1, 128.5, 128.1, 127.2, 126.4, 126.3, 125.2, 123.7, 123.1, 121.1, 107.2, 107.2, 75.3, 75.2, 59.0, 26.2, 26.2, 24.0, 23.9. **HRMS (ESI) m/z**: calculated for $C_{25}H_{22}N_3O_2S$ $[M-H]^-$:428.1438, found:428.1441. HPLC purity: 92.8%; t_R = 2.484 min, MeOH-H_2O (80:20).

(4aa) (*Z/E*)-2,2-dimethyl-9-((3-oxoisoindolin-1-ylidene)methyl)-9-phenyl-2,3-dihydro-1*H*,9*H*-pyrazolo[1,2-*a*]indazol-1-one

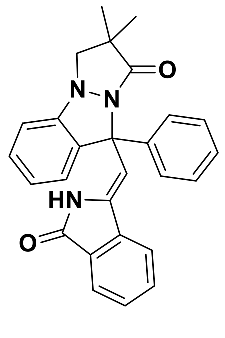

Following by general procedure for the synthesis of **4aa**. Yellow oil, 25 mg, yield: 60%. **^1H NMR (400 MHz, Chloroform-*d*)** δ 8.93 (s, 1H), 7.86 (d, *J* = 7.4 Hz, 1H), 7.70 (d, *J* = 7.6 Hz, 1H), 7.60 (td, *J* = 7.6, 1.2 Hz, 1H), 7.55 – 7.49 (m, 1H), 7.43 – 7.32 (m, 6H), 7.11 – 7.01 (m, 2H), 6.90 (d, *J* = 7.9 Hz, 1H), 5.94 (s, 1H), 3.78 (d, *J* = 8.3 Hz, 1H), 3.44 (d, *J* = 8.3 Hz, 1H),

1.40 (s, 3H), 1.33 (s, 3H). **^{13}C{^{1}H} NMR (151 MHz, Chloroform-*d*)** δ 169.8, 168.4, 138.9, 137.7, 137.0, 135.9, 132.0, 129.6, 129.5, 129.4, 128.8, 128.5, 127.8, 123.8, 123.5, 120.1, 110.3, 104.9, 69.1, 64.3, 46.0, 22.8, 22.7. **HRMS (ESI) m/z**: calculated for $C_{27}H_{22}N_3O_2$ [M - H]$^-$: 420.1718, found: 420.1716.

(4ba) (*Z/E*)-7-fluoro-2,2-dimethyl-9-((3-oxoisoindolin-1-ylidene)methyl)-9-phenyl-2,3-dihydro-1*H*,9*H*-pyrazolo[1,2-*a*]indazol-1-one

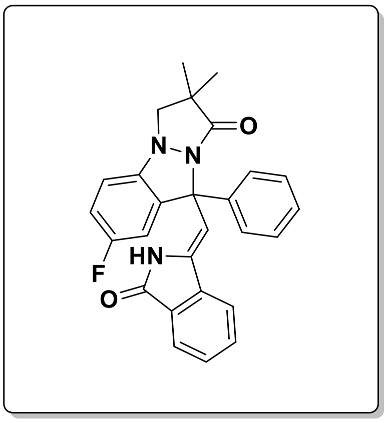

Following by general procedure for the synthesis of **4aa**. Yellow oil, 44 mg, yield: 51%. **^{1}H NMR (500 MHz, Chloroform-*d*)** δ 9.05 (s, 1H), 7.83 (d, *J* = 7.5 Hz, 1H), 7.68 (d, *J* = 7.7 Hz, 1H), 7.58 (td, *J* = 7.5, 1.2 Hz, 1H), 7.53 – 7.47 (m, 1H), 7.37 – 7.31 (m, 5H), 7.02 (td, *J* = 8.6, 2.5 Hz, 1H), 6.82 (dd, *J* = 8.7, 4.1 Hz, 1H), 6.70 (dd, *J* = 7.9, 2.5 Hz, 1H), 5.85 (s, 1H), 3.76 (d, *J* = 8.2 Hz, 1H), 3.35 (d, *J* = 8.2 Hz, 1H), 1.37 (s, 3H), 1.29 (s, 3H). **^{13}C{^{1}H} NMR** (126 MHz, Chloroform-*d*) δ 169.6, 167.9, 159.2 (d, *J* = 242.6 Hz), 141.8, 137.6, 137.1, 137.0, 131.7, 129.3, 129.1, 128.4, 128.3, 127.3, 123.1, 119.6, 115.9 (d, *J* = 24.0 Hz), 110.8 (d, *J* = 8.5 Hz), 110.6 (d, *J* = 25.0 Hz), 103.5, 68.8, 64.3, 45.7, 22.3, 22.1. **HRMS (ESI) m/z**: calculated for $C_{27}H_{21}FN_3O_2$ [M - H]$^-$: 438.1623, found: 438.1628.

(4ca) (*Z/E*)-7-chloro-2,2-dimethyl-9-((3-oxoisoindolin-1-ylidene)methyl)-9-phenyl-2,3-dihydro-1*H*,9*H*-pyrazolo[1,2-*a*]indazol-1-one

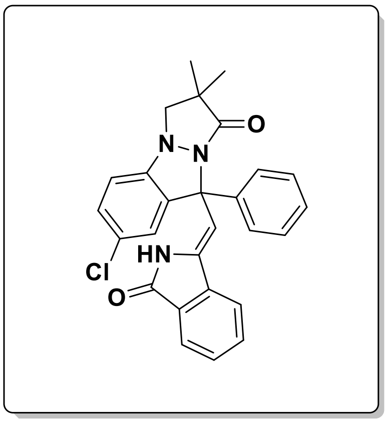

Following by general procedure for the synthesis of **4aa**. Yellow oil, 42 mg, yield: 47%. **^{1}H NMR (400 MHz, Chloroform-*d*)** δ 9.00 (s, 1H), 7.86 (dt, *J* = 7.5, 1.1 Hz, 1H), 7.72 (dt, *J* = 7.7, 1.0 Hz, 1H), 7.61 (td, *J* = 7.4, 1.1 Hz, 1H), 7.54 (td, *J* = 7.3, 0.8 Hz, 1H), 7.43 – 7.32 (m, 5H), 7.30 (dd, *J* = 8.4, 2.0 Hz, 1H), 6.97 (d, *J* = 2.0 Hz, 1H), 6.83 (d, *J* = 8.4 Hz, 1H), 5.85 (s, 1H),

3.79 (d, *J* = 8.3 Hz, 1H), 3.39 (d, *J* = 8.3 Hz, 1H), 1.39 (s, 3H), 1.32 (s, 3H). **^{13}C{^{1}H} NMR (126 MHz, Chloroform-*d*)** δ 169.4, 167.9, 144.2, 137.5, 137.3, 137.0, 131.65, 129.31, 129.02, 128.48, 128.33, 127.31, 123.40, 123.12, 119.6, 110.8, 103.5, 68.7, 63.8, 45.7, 22.4, 22.2. **HRMS (ESI) m/z**: calculated for $C_{27}H_{21}ClN_3O_2$ [M - H]$^-$: 454.1328, found: 454.1326.

(4da) (*Z/E*)-7-bromo-2,2-dimethyl-9-((3-oxoisoindolin-1-ylidene)methyl)-9-phenyl-2,3-dihydro-1*H*,9*H*-pyrazolo[1,2-*a*]indazol-1-one

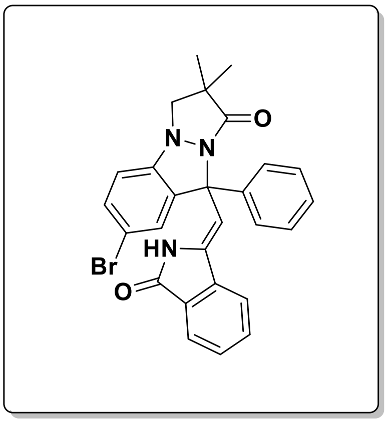

Following by general procedure for the synthesis of **4aa**. Yellow oil, 57 mg, yield: 56%. **^{1}H NMR (400 MHz, Chloroform-*d*)** δ 8.99 (s, 1H), 7.86 (dt, *J* = 7.5, 1.1 Hz, 1H), 7.72 (dt, *J* = 7.8, 1.0 Hz, 1H), 7.61 (td, *J* = 7.5, 1.2 Hz, 1H), 7.54 (td, *J* = 7.4, 1.0 Hz, 1H), 7.45 (dd, *J* = 8.4, 1.9 Hz, 1H), 7.42 – 7.32 (m, 5H), 7.11 (d, *J* = 1.8 Hz, 1H), 6.78 (d, *J* = 8.4 Hz, 1H), 5.85 (s, 1H), 3.78 (d, *J* = 8.3 Hz, 1H), 3.39 (d, *J* = 8.3 Hz, 1H), 1.39 (s, 3H), 1.32 (s, 3H). **^{13}C{^{1}H} NMR (126 MHz, Chloroform-*d*)** δ 169.3, 167.9, 144.6, 137.7, 137.5, 137.1, 137.0, 131.8, 131.7, 129.3, 128.5, 128.3, 127.3, 126.2, 123.1, 119.6, 115.4, 111.3, 103.6, 68.6, 63.7, 45.7, 22.4, 22.2. **HRMS (ESI) m/z**: calculated for $C_{27}H_{22}BrN_3NaO_2$ [M + Na]$^+$: 522.0788, found: 522.0790.

(4ea) (*Z/E*)-2,2,7-trimethyl-9-((3-oxoisoindolin-1-ylidene)methyl)-9-phenyl-2,3-dihydro-1*H*,9*H*-pyrazolo[1,2-*a*]indazol-1-one

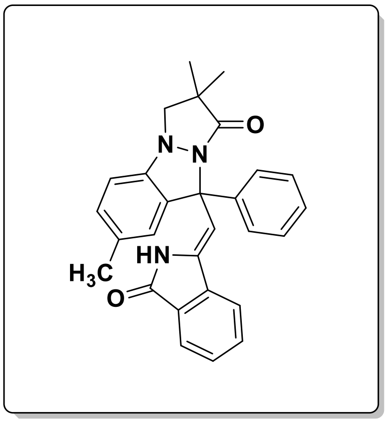

Following by general procedure for the synthesis of **4aa**. Yellow oil, 47 mg, yield: 54%. **^{1}H NMR (400 MHz, Chloroform-*d*)** δ 8.79 (s, 1H), 7.59 (td, *J* = 7.5, 1.2 Hz, 1H), 7.52 (td, *J* = 7.4, 1.0 Hz, 1H), 7.38 – 7.30 (m, 1H), 7.28 – 7.23 (m, 1H), 7.21 – 7.12 (m, 3H), 7.10 – 7.03 (m, 2H), 6.90 (d, *J* = 7.9 Hz, 1H), 5.95 (s, 1H), 3.74 (d, *J* = 8.3 Hz, 1H), 3.47 (d, *J* = 8.3 Hz, 1H), 2.34 (s, 3H), 1.39 (s, 3H), 1.34 (s, 3H). **^{13}C{^{1}H} NMR (126

MHz, Chloroform-*d*) δ 169.4, 167.9, 143.5, 138.5, 137.2, 136.5, 135.5, 133.2, 131.6, 129.6, 129.1, 128.3, 128.0, 127.3, 123.6, 123.0, 119.6, 109.7, 104.7, 68.6, 64.3, 45.6, 22.3, 22.2, 20.6. **HRMS (ESI) m/z**: calculated for $C_{28}H_{25}N_3NaO_2$ $[M + Na]^+$: 458.1839, found: 458.1841.

(4fa) (*Z/E*)-7-methoxy-2,2-dimethyl-9-((3-oxoisoindolin-1-ylidene)methyl)-9-phenyl-2,3-dihydro-1*H*,9*H*-pyrazolo[1,2-*a*]indazol-1-one

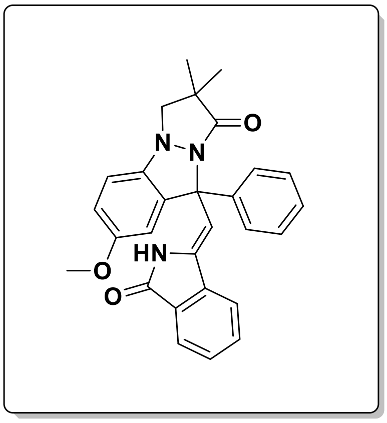

Following by general procedure for the synthesis of **4aa**. Yellow oil, 45 mg, yield: 50%. **^{1}H NMR (500 MHz, Chloroform-*d*) δ** 9.01 (s, 1H), 7.83 (d, *J* = 7.5 Hz, 1H), 7.68 (d, *J* = 7.6 Hz, 1H), 7.57 (t, *J* = 7.3 Hz, 1H), 7.50 (t, *J* = 7.4 Hz, 1H), 7.40 – 7.31 (m, 5H), 6.87 (dd, *J* = 8.7, 2.4 Hz, 1H), 6.81 (d, *J* = 8.7 Hz, 1H), 6.52 (d, *J* = 2.4 Hz, 1H), 5.91 (s, 1H), 3.73 (d, *J* = 6.4 Hz, 4H), 3.33 (d, *J* = 8.2 Hz, 1H), 1.36 (s, 3H), 1.28 (s, 3H). **^{13}C{^{1}H} NMR (126 MHz, Chloroform-*d*)** δ 169.6, 167.9, 156.5, 139.5, 138.1, 136.7, 131.6, 129.2, 128.3, 128.1, 127.4, 123.1, 119.6, 114.6, 110.7, 109.0, 104.2, 68.8, 64.8, 55.4, 45.6, 22.3, 22.2. **HRMS (ESI) m/z**: calculated for $C_{28}H_{25}N_3NaO_3$ $[M + Na]^+$: 474.1788, found: 474.1790.

(4ga) (*Z/E*)-2,2-dimethyl-9-((3-oxoisoindolin-1-ylidene)methyl)-9-phenyl-7-(trifluoromethyl)-2,3-dihydro-1*H*,9*H*-pyrazolo[1,2-*a*]indazol-1-one

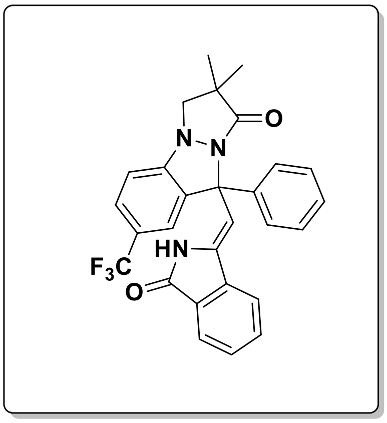

Following by general procedure for the synthesis of **4aa**. Yellow oil, 65 mg, yield: 71%. **^{1}H NMR (400 MHz, Chloroform-*d*)** δ 9.00 (s, 1H), 7.87 (dt, *J* = 7.5, 1.1 Hz, 1H), 7.73 (dt, *J* = 7.7, 1.0 Hz, 1H), 7.62 (td, *J* = 7.6, 1.3 Hz, 2H), 7.55 (td, *J* = 7.3, 1.2 Hz, 1H), 7.43 – 7.34 (m, 5H), 7.23 (s, 1H), 6.95 (d, *J* = 8.3 Hz, 1H), 5.83 (s, 1H), 3.85 (d, *J* = 8.4 Hz, 1H), 3.46 (d, *J* = 8.4 Hz, 1H), 1.41 (s, 3H), 1.34 (s, 3H). **^{13}C{^{1}H} NMR (126**

MHz, Chloroform-*d*) δ 169.3, 167.9, 148.0, 137.3, 137.2, 137.0, 136.4, 131.7, 129.4, 129.1, 128.6, 128.4, 127.3, 126.6, 126.6, 123.1, 120.5, 120.4, 119.7, 68.7, 63.0, 45.6, 22.5, 22.2. **HRMS (ESI) m/z**: calculated for $C_{28}H_{21}F_3N_3O_2$ [M - H]$^-$: 488.1591, found:488.1595.

(4ha) (*Z/E*)-2,2-dimethyl-1-oxo-9-((3-oxoisoindolin-1-ylidene)methyl)-9-phenyl-2,3-dihydro-1*H*,9*H*-pyrazolo[1,2-*a*]indazole-7-carbonitrile

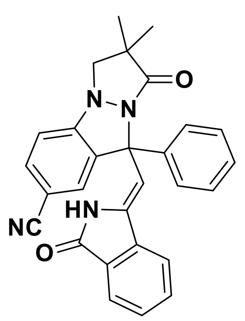

Following by general procedure for the synthesis of **4aa**. Yellow solid, 55 mg, yield: 62%. **^{1}H NMR (500 MHz, Chloroform-*d*)** δ 8.93 (s, 1H), 7.84 (d, *J* = 7.5 Hz, 1H), 7.69 (d, *J* = 7.5 Hz, 1H), 7.63 – 7.58 (m, 2H), 7.54 (dd, *J* = 7.5, 0.9 Hz, 1H), 7.40 – 7.32 (m, 5H), 7.24 (d, *J* = 1.5 Hz, 1H), 6.89 (d, *J* = 8.2 Hz, 1H), 5.75 (s, 1H), 3.83 (d, *J* = 8.5 Hz, 1H), 3.47 (d, *J* = 8.5 Hz, 1H), 1.38 (s, 3H), 1.33 (s, 3H). **^{13}C{^{1}H} NMR (126 MHz, Chloroform-*d*)** δ 169.1, 167.8, 148.3, 137.5, 137.0, 137.0, 136.9, 133.6, 131.8, 129.5, 129.1, 128.7, 128.6, 127.2, 127.1, 123.2, 119.6, 109.7, 105.9, 102.8, 68.6, 62.4, 45.7, 22.6, 22.2. **HRMS (ESI) m/z**: calculated for $C_{28}H_{22}N_4NaO_2$ [M + Na]$^+$: 469.1635, found: 469.163.

(4ia) (*Z/E*)-5-chloro-2,2-dimethyl-9-((3-oxoisoindolin-1-ylidene)methyl)-9-phenyl-2,3-dihydro-1*H*,9*H*-pyrazolo[1,2-a]indazol-1-one

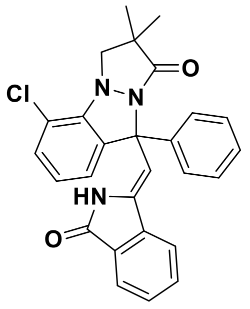

Following by general procedure for the synthesis of **4aa**. Yellow solid, 40 mg, yield: 45%. **^{1}H NMR (600 MHz, DMSO-*d*$_6$)** δ ^{1}H NMR (600 MHz, DMSO) δ 9.51 (s, 1H), 8.07 (d, *J* = 7.7 Hz, 1H), 7.75 (d, *J* = 7.5 Hz, 1H), 7.70 (t, *J* = 7.5 Hz, 1H), 7.60 (t, *J* = 7.5 Hz, 1H), 7.41 – 7.35 (m, 4H), 7.33 (t, *J* = 6.7 Hz, 1H), 7.03 (d, *J* = 9.9 Hz, 1H), 7.02 – 6.99 (m, 1H), 6.86 (t, *J* = 8.0 Hz, 1H), 6.16 (s, 1H), 3.99 (d, *J* = 8.7 Hz, 1H), 3.54 (d, *J* = 8.7 Hz, 1H), 1.25 (s,

3H), 1.23 (s, 3H). **$^{13}C\{^1H\}$ NMR (151 MHz, DMSO-d_6)** δ 169.9, 167.5, 163.5, 162.1, 147.3, 147.2, 139.0, 137.3, 136.4, 132.6, 131.7, 130.0, 129.7, 128.8, 128.7, 128.1, 127.5, 125.2, 125.2, 122.9, 121.2, 110.1, 110.0, 105.6, 98.5, 98.3, 68.4, 62.7, 45.6, 22.5, 22.1. **HRMS (ESI) m/z**: calculated for $C_{27}H_{22}FN_3NaO_2$ [M + Na]$^+$: 462.1588, found: 462.1591. HPLC purity: 93.2%; t_R = 3.055 min, MeOH-H_2O (80:20).

(4ja) (*Z/E*)-6-chloro-2,2-dimethyl-9-((3-oxoisoindolin-1-ylidene)methyl)-9-phenyl-2,3-dihydro-1*H*,9*H*-pyrazolo[1,2-*a*]indazol-1-one

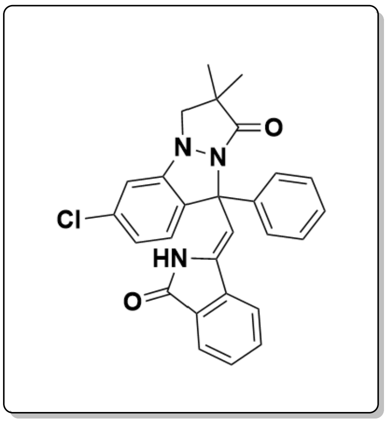

Following by general procedure for the synthesis of **4aa**. Yellow solid, 43 mg, yield: 47%. **^1H NMR (600 MHz, DMSO-d_6)** δ 9.52 (s, 1H), 8.07 (d, J = 7.7 Hz, 1H), 7.75 (d, J = 7.5 Hz, 1H), 7.70 (t, J = 7.5 Hz, 1H), 7.60 (t, J = 7.4 Hz, 1H), 7.40 – 7.35 (m, 4H), 7.33 (t, J = 7.4 Hz, 1H), 7.25 (d, J = 1.7 Hz, 1H), 7.08 (dd, J = 8.2, 1.8 Hz, 1H), 7.01 (d, J = 8.2 Hz, 1H), 6.15 (s, 1H), 4.01 (d, J = 8.7 Hz, 1H), 3.56 (d, J = 8.7 Hz, 1H), 1.24 (s, 3H), 1.23 (s, 3H). **$^{13}C\{^1H\}$ NMR (201 MHz, DMSO-d_6)** 169.3, 166.9, 146.4, 138.3, 136.6, 135.9, 134.0, 133.1, 132.0, 129.4, 128.1, 127.5, 126.9, 124.5, 122.5, 122.2, 120.6, 110.1, 104.5, 67.8, 62.0, 44.9, 21.9, 21.5. **HRMS (ESI) m/z**: calculated for $C_{27}H_{21}ClN_3O_2$ [M- H]$^-$: 454.1328, found:454.1328. HPLC purity: 93.4%; t_R = 3.054 min, MeOH-H_2O (80:20).

(4ka) (*Z/E*)-6-bromo-2,2-dimethyl-9-((3-oxoisoindolin-1-ylidene)methyl)-9-phenyl-2,3-dihydro-1*H*,9*H*-pyrazolo[1,2-*a*]indazol-1-one

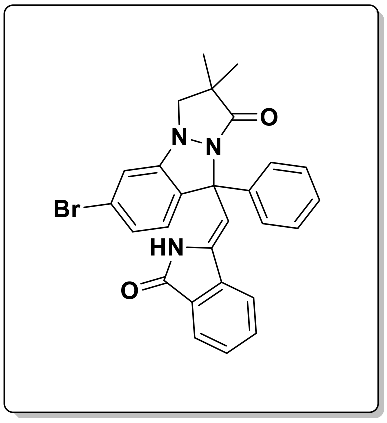

Following by general procedure for the synthesis of **4aa**. Yellow solid, 67 mg, yield: 68%. **^1H NMR (400 MHz, Chloroform-*d*)** δ 8.90 (s, 1H), 7.86 (dt, J = 7.4, 1.1 Hz, 1H), 7.70 (dt, J = 7.8, 1.0 Hz, 1H), 7.61 (td, J = 7.5, 1.2 Hz, 1H), 7.53 (td, J = 7.4, 1.1 Hz, 1H), 7.42 – 7.33 (m, 5H), 7.18 (dd, J = 8.1, 1.7 Hz, 1H), 7.04 (d, J = 1.7 Hz, 1H), 6.88 (d, J = 8.2 Hz, 1H), 5.85 (s, 1H),

3.76 (d, *J* = 8.3 Hz, 1H), 3.43 (d, *J* = 8.3 Hz, 1H), 1.39 (s, 3H), 1.33 (s, 3H). **^{13}C{^{1}H} NMR (126 MHz, Chloroform-*d*)** δ 169.3, 167.9, 146.9, 137.8, 137.1, 136.9, 134.7, 131.7, 129.3, 129.1, 128.5, 128.3, 127.2, 126.2, 124.6, 123.1, 122.5, 119.6, 113.1, 103.7, 68.6, 63.5, 45.6, 22.4, 22.2. **HRMS (ESI) m/z**: calculated for $C_{27}H_{21}BrN_3O_2$ [M- H]$^-$: 498.0823, found:498.0822.

(4ab) (*Z/E*)-9-(4-fluorophenyl)-2,2-dimethyl-9-((3-oxoisoindolin-1-ylidene)methyl)-2,3-dihydro-1*H*,9*H*-pyrazolo[1,2-*a*]indazol-1-one

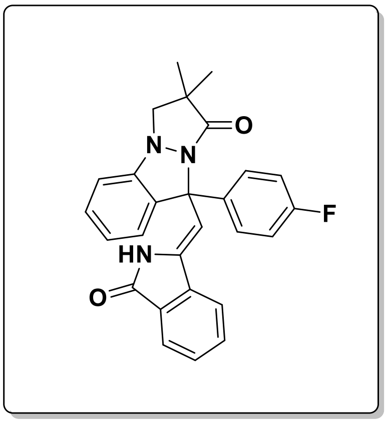

Following by general procedure for the synthesis of **4aa**. Yellow solid, 52 mg, yield: 60%. **^{1}H NMR (400 MHz, Chloroform-*d*)** δ 9.10 (s, 1H), 7.86 (dt, *J* = 7.4, 1.0 Hz, 1H), 7.69 (dd, *J* = 7.6, 1.0 Hz, 1H), 7.60 (td, *J* = 7.5, 1.2 Hz, 1H), 7.52 (td, *J* = 7.4, 1.0 Hz, 1H), 7.40 – 7.34 (m, 3H), 7.10 – 6.98 (m, 4H), 6.91 (d, *J* = 7.9 Hz, 1H), 5.90 (s, 1H), 3.83 (d, *J* = 8.3 Hz, 1H), 3.38 (d, *J* = 8.4 Hz, 1H), 1.40 (s, 3H), 1.31 (s, 3H). **^{13}C{^{1}H} NMR (126 MHz, Chloroform-*d*)** δ 169.4, 168.0, 162.1 (d, *J* = 248.5 Hz), 145.6, 137.1, 136.7, 135.3, 134.1, 134.1, 131.7, 129.4 (d, *J* = 8.5 Hz), 129.2 (d, *J* = 26.0 Hz), 123.5, 123.2, 123.1, 119.6, 115.4, 115.2, 109.9, 104.2, 68.1, 63.9, 45.6, 22.4, 22.1. **HRMS (ESI) m/z**: calculated for $C_{27}H_{22}FN_3NaO_2$ [M+ Na]$^+$: 462.1588, found:462.1588.

(4ac) (*Z/E*)-9-(4-chlorophenyl)-2,2-dimethyl-9-((3-oxoisoindolin-1-ylidene)methyl)-2,3-dihydro-1*H*,9*H*-pyrazolo[1,2-*a*]indazol-1-one

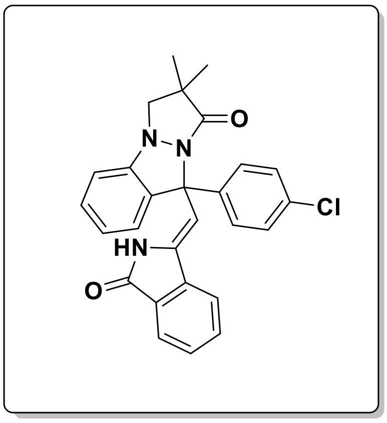

Following by general procedure for the synthesis of **4aa**. Yellow solid, 47 mg, yield: 52%. **^{1}H NMR (500 MHz, Chloroform-*d*)** δ 9.06 (s, 1H), 7.83 (d, *J* = 7.5 Hz, 1H), 7.66 (d, *J* = 7.7 Hz, 1H), 7.57 (t, *J* = 7.5 Hz, 1H), 7.50 (t, *J* = 7.4 Hz, 1H), 7.36 – 7.27 (m, 5H), 7.05 (t, *J* = 7.5 Hz, 1H), 6.96 (d, *J* = 7.7 Hz, 1H), 6.88 (d, *J* = 7.9 Hz, 1H), 5.85 (s, 1H), 3.80 (d, *J* = 8.3 Hz, 1H),

3.36 (d, *J* = 8.4 Hz, 1H), 1.37 (s, 3H), 1.28 (s, 3H). **^{13}C{^{1}H} NMR (126 MHz, Chloroform-*d*)** δ 169.4, 167.9, 145.5, 136.9, 135.1, 134.1, 131.7, 129.3, 129.1, 129.1, 128.8, 128.5, 123.5, 123.1, 119.6, 110.0, 103.8, 68.1, 63.9, 45.6, 22.4, 22.1. **HRMS (ESI) m/z**: calculated for $C_{27}H_{21}ClN_3O_2$ [M- H]$^-$: 454.1328, found:454.1329.

(4ad) (*Z/E*)-9-(4-bromophenyl)-2,2-dimethyl-9-((3-oxoisoindolin-1-ylidene)methyl)-2,3-dihydro-1*H*,9*H*-pyrazolo[1,2-*a*]indazol-1-one

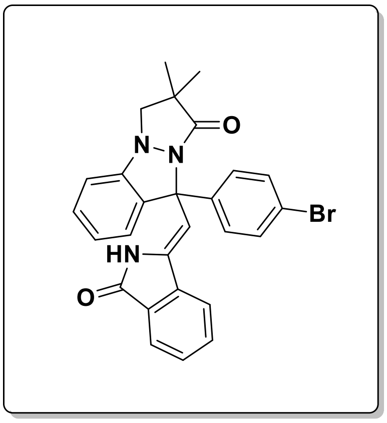

Following by general procedure for the synthesis of **4aa**. Yellow solid, 40 mg, yield: 40%. **^{1}H NMR (400 MHz, Chloroform-*d*)** δ 9.08 (s, 1H), 7.86 (dt, *J* = 7.5, 1.0 Hz, 1H), 7.69 (dt, *J* = 7.7, 1.0 Hz, 1H), 7.60 (td, *J* = 7.5, 1.2 Hz, 1H), 7.53 (td, *J* = 7.4, 1.1 Hz, 1H), 7.51 – 7.45 (m, 2H), 7.36 (td, *J* = 7.6, 1.2 Hz, 1H), 7.31 – 7.23 (m, 3H), 7.08 (td, *J* = 7.6, 1.0 Hz, 1H), 6.98 (dd, *J* = 7.8, 1.2 Hz, 1H), 6.91 (dt, *J* = 7.9, 0.8 Hz, 1H), 5.86 (s, 1H), 3.83 (d, *J* = 8.3 Hz, 1H), 3.38 (d, *J* = 8.3 Hz, 1H), 1.40 (s, 3H), 1.31 (s, 3H). **^{13}C{^{1}H} NMR (151 MHz, Chloroform-*d*)** δ 169.8, 168.4, 146.0, 137.9, 137.5, 137.4, 135.6, 132.1, 131.9, 129.8, 129.6, 129.6, 124.0, 123.6, 123.6, 122.9, 120.1, 110.4, 104.1, 68.5, 64.4, 46.1, 22.8, 22.6.**HRMS (ESI) m/z**: calculated for $C_{27}H_{21}BrN_3O_2$ [M-H]$^-$: 498.0823, found:498.0825.

(4ae) (*Z/E*)-2,2-dimethyl-9-((3-oxoisoindolin-1-ylidene)methyl)-9-(p-tolyl)-2,3-dihydro-1*H*,9*H*-pyrazolo[1,2-*a*]indazol-1-one

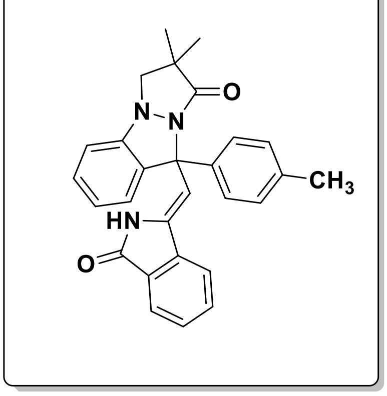

Following by general procedure for the synthesis of **4aa**. Yellow solid, 37 mg, yield: 43%. **^{1}H NMR (500 MHz, Chloroform-*d*)** δ 8.97 (s, 1H), 7.83 (d, *J* = 7.5 Hz, 1H), 7.67 (d, *J* = 7.6 Hz, 1H), 7.59 – 7.53 (m, 1H), 7.49 (t, *J* = 7.4 Hz, 1H), 7.31 (td, *J* = 7.6, 1.3 Hz, 1H), 7.25 (d, *J* = 8.3 Hz, 2H), 7.13 (d, *J* = 8.0 Hz, 2H), 7.07 – 6.97 (m, 2H), 6.87 (d, *J* = 7.9

Hz, 1H), 5.90 (s, 1H), 3.76 (d, J = 8.3 Hz, 1H), 3.39 (d, J = 8.2 Hz, 1H), 2.32 (s, 3H), 1.37 (s, 3H), 1.29 (s, 3H). **^{13}C{^{1}H} NMR (126 MHz, Chloroform-*d*)** δ 169.3, 167.9, 145.7, 137.9, 137.2, 136.4, 135.6, 135.4, 131.5, 129.1, 129.1, 128.8, 127.3, 123.3, 123.0, 119.6, 109.8, 104.8, 68.6, 63.9, 45.6, 22.4, 22.2, 20.7.**HRMS (ESI) m/z**: calculated for $C_{28}H_{25}N_3NaO_2$ [M+ Na]$^+$: 458.1839, found:458.1841.

(4ag) (*Z/E*)-2,2-dimethyl-9-((3-oxoisoindolin-1-ylidene)methyl)-9-(4-pentylphenyl)-2,3-dihydro-1*H*,9*H*-pyrazolo[1,2-*a*]indazol-1-one

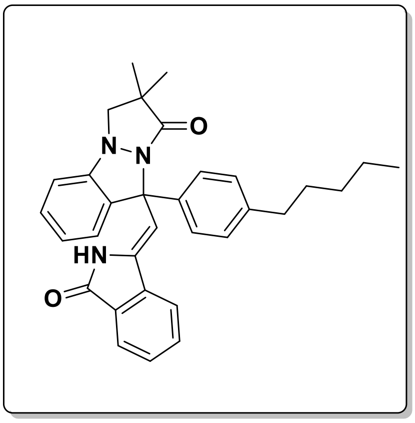

Following by general procedure for the synthesis of **4aa**. Yellow solid, 47 mg, yield: 48%. **^{1}H NMR (500 MHz, Chloroform-*d*)** δ 8.86 (s, 1H), 7.83 (dt, J = 7.6, 1.0 Hz, 1H), 7.69 – 7.65 (m, 1H), 7.56 (td, J = 7.5, 1.2 Hz, 1H), 7.49 (td, J = 7.5, 1.0 Hz, 1H), 7.34 – 7.27 (m, 2H), 7.30 – 7.24 (m, 3H), 7.16 – 7.12 (m, 2H), 7.07 – 7.00 (m, 2H), 6.87 (d, J = 7.9 Hz, 1H), 5.92 (s, 1H), 3.73 (d, J = 8.3 Hz, 1H), 3.41 (d, J = 8.3 Hz, 1H), 2.61 – 2.55 (m, 2H), 1.63 – 1.55 (m, 2H), 1.37 (s, 3H), 1.34 – 1.30 (m, 7H), 0.91 – 0.87 (m, 3H). **^{13}C{^{1}H} NMR (126 MHz, Chloroform-*d*)** δ 145.7, 142.8, 136.3, 135.6, 135.5, 131.5, 129.1, 129.1, 128.8, 128.3, 127.1, 123.4, 123.2, 123.0, 119.6, 109.8, 104.9, 68.6, 63.8, 45.6, 35.1, 31.2, 30.4, 22.4, 22.3, 22.1, 13.6.**HRMS (ESI) m/z**: calculated for $C_{32}H_{32}N_3O_2$ [M- H]$^-$: 490.2500, found:490.2503.

(4ai) (*Z/E*)-2,2-dimethyl-9-((3-oxoisoindolin-1-ylidene)methyl)-9-(m-tolyl)-2,3-dihydro-1*H*,9*H*-pyrazolo[1,2-*a*]indazol-1-one

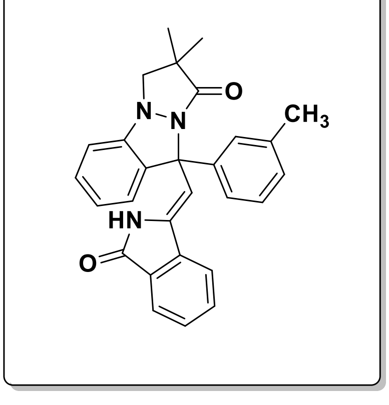

Following by general procedure for the synthesis of **4aa**. Yellow solid, 46 mg, yield: 54%. **^{1}H NMR (500 MHz, Chloroform-*d*)** δ 8.83 (s, 1H), 7.85 (d, J = 7.5 Hz, 1H), 7.71 (d, J = 7.7 Hz, 1H), 7.62 – 7.56 (m, 1H), 7.52 (t, J = 7.4 Hz, 1H), 7.37 – 7.31 (m, 1H), 7.26 (dd, J = 15.7, 8.0 Hz, 1H), 7.19 (d, J = 7.9 Hz, 2H), 7.14 (d, J = 7.5 Hz, 1H), 7.11 – 7.03 (m, 2H), 6.90 (d, J =

7.9 Hz, 1H), 5.95 (s, 1H), 3.75 (d, *J* = 8.3 Hz, 1H), 3.47 (d, *J* = 8.3 Hz, 1H), 2.34 (s, 3H), 1.39 (s, 3H), 1.34 (s, 3H). **$^{13}C\{^{1}H\}$ NMR (126 MHz, Chloroform-*d*)** δ 169.2, 167.8, 145.6, 138.5, 137.9, 137.2, 136.3, 135.3, 131.5, 129.1, 129.0, 128.9, 128.8, 128.2, 127.7, 124.4, 123.3, 123.2, 123.0, 119.6, 109.8, 104.6, 68.6, 63.8, 45.5, 22.3, 22.3, 21.2.**HRMS (ESI) m/z**: calculated for $C_{28}H_{25}N_3NaO_2$ $[M+ Na]^+$: 458.1839, found:458.1841.

(4aj) (*Z/E*)-9-(2-chlorophenyl)-2,2-dimethyl-9-((3-oxoisoindolin-1-ylidene)methyl)-2,3-dihydro-1*H*,9*H*-pyrazolo[1,2-*a*]indazol-1-one

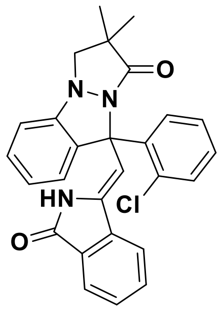

Following by general procedure for the synthesis of **4aa**. Yellow solid, 47 mg, yield: 52%. **^{1}H NMR (500 MHz, Chloroform-*d*)** δ 9.38 (s, 1H), 7.86 (dt, *J* = 7.5, 1.1 Hz, 1H), 7.78 – 7.73 (m, 1H), 7.68 – 7.64 (m, 1H), 7.57 (td, *J* = 7.5, 1.2 Hz, 1H), 7.53 – 7.48 (m, 1H), 7.36 – 7.26 (m, 4H), 7.02 (td, *J* = 7.6, 1.0 Hz, 1H), 6.86 (dd, *J* = 7.6, 1.2 Hz, 1H), 6.82 (d, *J* = 7.8 Hz, 1H), 5.71 (s, 1H), 3.85 (d, *J* = 8.1 Hz, 1H), 3.61 (d, *J* = 8.1 Hz, 1H), 1.40 (s, 3H), 1.34 (s, 3H). **$^{13}C\{^{1}H\}$ NMR (126 MHz, Chloroform-*d*)** δ 169.5, 168.0, 146.6, 137.1, 136.9, 134.3, 133.2, 132.0, 131.9, 131.6, 130.7, 130.1, 129.4, 129.3, 129.0, 126.7, 123.2, 122.8, 121.5, 119.7, 109.0, 104.6, 67.7, 62.4, 45.5, 23.5, 22.1. **HRMS (ESI) m/z**: calculated for $C_{27}H_{22}ClN_3NaO_2$ $[M+ Na]^+$: 478.1293, found:478.1295.

(4ak) (*Z/E*)-9-(3-chlorophenyl)-2,2-dimethyl-9-((3-oxoisoindolin-1-ylidene)methyl)-2,3-dihydro-1*H*,9*H*-pyrazolo[1,2-*a*]indazol-1-one

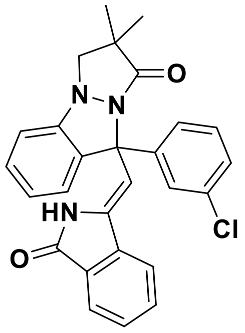

Following by general procedure for the synthesis of **4aa**. Yellow solid, 42 mg, yield: 46%. **^{1}H NMR (600 MHz, DMSO-d_6)** δ ^{1}H NMR (600 MHz, DMSO) δ 9.51 (s, 1H), 8.08 (d, *J* = 7.7 Hz, 1H), 7.75 (d, *J* = 7.4 Hz, 1H), 7.71 (t, *J* = 7.4 Hz, 1H), 7.61 (t, *J* = 7.4 Hz, 1H), 7.41 (s, 2H), 7.37 (t, *J* = 6.4 Hz, 1H), 7.34 (s, 2H), 7.13 – 7.02 (m, 3H), 6.17 (s, 1H), 4.00 (d, *J* =

8.6 Hz, 1H), 3.52 (d, J = 8.6 Hz, 1H), 1.26 (s, 3H), 1.23 (s, 3H). **$^{13}C\{^1H\}$ NMR (201 MHz, DMSO-d_6)** δ 169.4, 166.9, 145.0, 140.9, 136.6, 135.9, 134.5, 132.6, 132.0, 129.5, 128.9, 128.1, 127.6, 126.6, 126.0, 123.0, 123.0, 122.2, 120.7, 110.0, 104.4, 67.4, 62.4, 45.0, 21.8, 21.4. **HRMS (ESI) m/z**: calculated for $C_{27}H_{22}ClN_3NaO_2$ [M+ Na]$^+$: 478.1293, found:478.1294.

(4al) (*Z/E*)-2,2-dimethyl-9-((3-oxoisoindolin-1-ylidene)methyl)-9-(thiophen-3-yl)-2,3-dihydro-1*H*,9*H*-pyrazolo[1,2-*a*]indazol-1-one

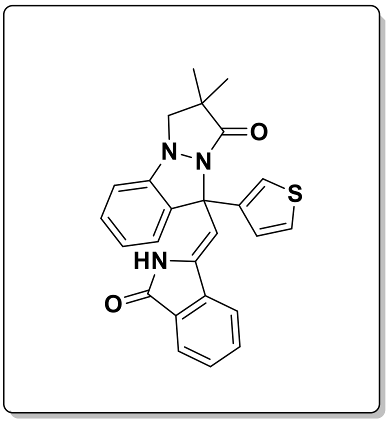

Following by general procedure for the synthesis of **4aa**. Yellow solid, 17 mg, yield: 21%. **^1H NMR (400 MHz, Chloroform-*d*)** δ 9.28 (s, 1H), 7.86 (dt, J = 7.4, 1.1 Hz, 1H), 7.67 (dt, J = 7.8, 1.0 Hz, 1H), 7.59 (td, J = 7.5, 1.3 Hz, 1H), 7.52 (td, J = 7.4, 1.0 Hz, 1H), 7.38 – 7.30 (m, 3H), 7.12 – 7.05 (m, 2H), 6.90 (dd, J = 7.9, 0.8 Hz, 1H), 6.87 – 6.83 (m, 1H), 5.94 (s, 1H), 3.82 (d, J = 8.3 Hz, 1H), 3.33 (d, J = 8.3 Hz, 1H), 1.40 (s, 3H), 1.31 (s, 3H). **$^{13}C\{^1H\}$ NMR (151 MHz, Chloroform-*d*)** δ 170.0, 168.4, 146.1, 140.0, 137.6, 137.0, 135.3, 132.0, 129.7, 129.5, 127.1, 127.1, 124.7, 123.8, 123.5, 123.5, 120.0, 110.4, 105.4, 66.0, 64.6, 46.1, 22.9, 22.7.**HRMS (ESI) m/z**: calculated for $C_{25}H_{20}N_3O_2S$[M- H]$^-$: 426.1282, found:426.1284. HPLC purity: 98.6%; t_R = 2.443 min, MeOH-H_2O (80:20).

(5aa) 2-(1-(3-amino-2,2-dimethyl-3-oxopropyl)-2-phenyl-1*H*-indole-3-carbonyl)benzamide

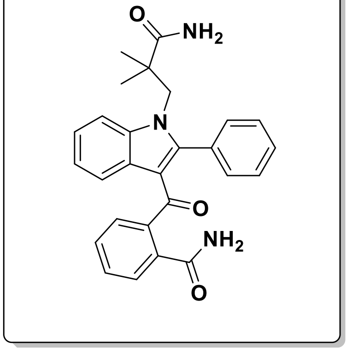

Following by general procedure for the synthesis of **5aa**. White solid, 72 mg, yield: 80%. **^1H NMR (400 MHz, DMSO-d_6)** δ 8.02 (d, J = 7.2 Hz, 1H), 7.75 (d, J = 8.2 Hz, 1H), 7.72 (s, 1H), 7.43 – 7.33 (m, 3H), 7.28 – 7.24 (m, 1H), 7.22 – 7.09 (m, 7H), 7.02 – 6.98 (m, 1H), 6.92 (s, 1H), 6.88 (dd, J = 7.7, 1.2 Hz, 1H), 4.42 (s, 2H), 0.68

(s, 6H). **$^{13}C\{^{1}H\}$ NMR (126 MHz, DMSO-*d*6)** δ 192.8, 178.2, 169.7, 147.8, 142.4, 137.6, 135.6, 130.9, 129.3, 128.8, 128.6, 128.2, 127.8, 127.5, 127.3, 123.1, 122.4, 121.8, 115.2, 112.7, 50.5, 44.0, 24.7. **HRMS (ESI) m/z**: calculated for $C_{27}H_{24}N_3O_3$ $[M - H]^-$: 438.1823, found: 438.1824.

(5ba) 2-(1-(3-amino-2,2-dimethyl-3-oxopropyl)-5-fluoro-2-phenyl-1*H*-indole-3-carbonyl)benzamide

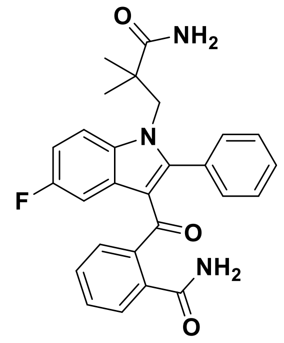

Following by general procedure for the synthesis of **5aa**. White solid, 76 mg, yield: 85%. **^{1}H NMR (500 MHz, DMSO-*d*6)** δ 7.78 (dd, *J* = 9.1, 4.5 Hz, 1H), 7.75 (s, 1H), 7.70 (dd, *J* = 10.1, 2.7 Hz, 1H), 7.40 (dd, *J* = 7.7, 1.2 Hz, 1H), 7.37 – 7.32 (m, 2H), 7.25 – 7.10 (m, 7H), 7.01 (td, *J* = 7.5, 1.2 Hz, 1H), 6.94 (s, 1H), 6.91 – 6.86 (m, 1H), 4.41 (s, 2H), 0.68 (s, 6H). **$^{13}C\{^{1}H\}$ NMR (126 MHz, DMSO-*d*6)** δ 192.7, 178.2, 169.6, 159.1 (d, *J* = 234.6 Hz), 149.2, 142.1, 135.5, 134.2, 131.9, 130.6, 129.4, 129.0, 128.7, 128.1, 128.0 (d, *J* = 11.0 Hz), 127.9, 127.5, 115.2, 114.1 (d, *J* = 9.7 Hz), 111.2 (d, *J* = 25.9 Hz), 106.5 (d, *J* = 24.8 Hz), 50.9, 43.9, 24.6. **HRMS (ESI) m/z**: calculated for $C_{27}H_{23}FN_3O_3$ $[M - H]^-$: 456.1729, found: 456.1732.

(5ca) 2-(1-(3-amino-2,2-dimethyl-3-oxopropyl)-5-chloro-2-phenyl-1*H*-indole-3-carbonyl)benzamide

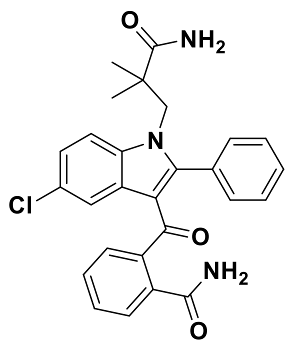

Following by general procedure for the synthesis of **5aa**. White solid, 86 mg, yield: 91%. **^{1}H NMR (400 MHz, DMSO-*d*6)** δ 8.05 (d, *J* = 2.2 Hz, 1H), 7.79 (d, *J* = 8.9 Hz, 1H), 7.75 (s, 1H), 7.40 (dd, *J* = 7.7, 1.1 Hz, 1H), 7.36 – 7.31 (m, 2H), 7.28 (dd, *J* = 8.9, 2.2 Hz, 1H), 7.23 (s, 1H), 7.20 – 7.07 (m, 5H), 7.02 (td, *J* = 7.5, 1.2 Hz, 1H), 6.96 – 6.83 (m, 2H), 4.40 (s, 2H),

0.67 (s, 6H). **^{13}C{^{1}H} NMR** (126 MHz, DMSO-d_6) δ 177.6, 169.0, 148.4, 141.5, 135.6, 134.9, 129.8, 128.9, 128.5, 128.2, 128.0, 127.6, 127.4, 127.0, 126.7, 122.6, 120.3, 114.4, 113.9, 50.4, 43.4, 24.1. **HRMS (ESI) m/z**: calculated for $C_{27}H_{23}ClN_3O_3$ [M - H]$^-$: 472.1433, found: 472.1434.

(5fa) 2-(1-(3-amino-2,2-dimethyl-3-oxopropyl)-5-methoxy-2-phenyl-1*H*-indole-3-carbonyl)benzamide

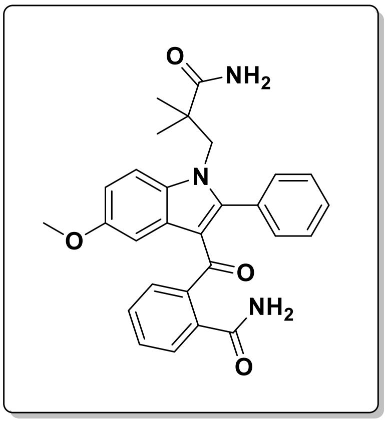

Following by general procedure for the synthesis of **5aa**. White solid, 76 mg, yield: 81%. **^{1}H NMR (400 MHz, DMSO-d_6)** δ 7.72 (s, 1H), 7.64 (d, J = 9.0 Hz, 1H), 7.57 (d, J = 2.6 Hz, 1H), 7.42 – 7.31 (m, 3H), 7.21 (s, 1H), 7.18 – 7.07 (m, 5H), 7.00 – 6.95 (m, 1H), 6.91 (s, 1H), 6.88 (dd, J = 9.0, 2.6 Hz, 1H), 6.84 (d, J = 7.5 Hz, 1H), 4.38 (s, 2H), 3.76 (s, 3H), 0.66 (s, 6H). **^{13}C{^{1}H} NMR (126 MHz, DMSO-d_6)** δ 178.2, 169.8, 155.9, 148.1, 142.4, 135.6, 132.6, 131.1, 129.2, 128.7, 128.5, 128.2, 128.1, 127.8, 127.5, 114.9, 113.5, 112.8, 103.5, 55.7, 50.7, 44.0, 24.6. **HRMS (ESI) m/z**: calculated for $C_{28}H_{26}N_3O_4$ [M - H]$^-$: 468.1929, found: 468.1928.

(5ab) 2-(1-(3-amino-2,2-dimethyl-3-oxopropyl)-2-(4-fluorophenyl)-1*H*-indole-3-carbonyl)benzamide

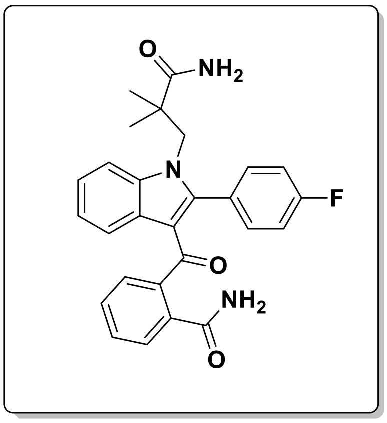

Following by general procedure for the synthesis of **5aa**. White solid, 65 mg, yield: 72%. **^{1}H NMR (500 MHz, DMSO-d_6)** δ 8.03 (d, J = 2.3 Hz, 1H), 7.76 (d, J = 8.8 Hz, 1H), 7.73 (s, 1H), 7.38 (d, J = 7.7 Hz, 1H), 7.33 – 7.30 (m, 2H), 7.26 (dd, J = 8.8, 2.3 Hz, 1H), 7.21 (s, 1H), 7.18 – 7.11 (m, 3H), 7.12 – 7.06 (m, 3H), 6.99 (t, J = 7.5 Hz, 1H), 6.91 (s, 1H), 6.86 (d, J = 7.5 Hz, 1H), 4.38 (s, 2H), 0.65 (s, 6H). **^{13}C{^{1}H} NMR**

(126 MHz, DMSO-d_6) δ 192.7, 178.1, 169.7, 162.3 (d, J = 246.3 Hz), 146.7, 142.4, 137.7, 135.6, 129.4, 128.5, 128.2, 127.6, 127.2, 123.3, 122.5, 121.8, 115.4, 114.9, 114.7, 112.7, 50.6, 44.1, 24.7. **HRMS (ESI) m/z**: calculated for $C_{27}H_{23}FN_3O_3$ $[M - H]^-$: 456.1729, found: 456.1733.

(5af) 2-(1-(3-amino-2,2-dimethyl-3-oxopropyl)-2-(4-methoxyphenyl)-1*H*-indole-3-carbonyl)benzamide

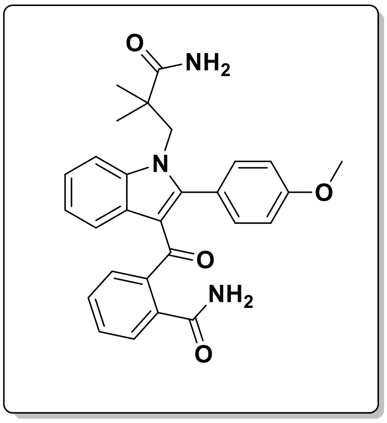

Following by general procedure for the synthesis of **5aa**. White solid, 68 mg, yield: 72%. **^{1}H NMR (500 MHz, DMSO-d_6)** δ 8.02 (d, J = 7.9 Hz, 1H), 7.71 (d, J = 8.9 Hz, 2H), 7.39 (d, J = 7.7 Hz, 1H), 7.23 (t, J = 6.3 Hz, 3H), 7.16 (dd, J = 14.1, 7.4 Hz, 3H), 7.08 (s, 1H), 6.99 (t, J = 7.8 Hz, 1H), 6.90 (s, 1H), 6.81 (d, J = 7.6 Hz, 1H), 6.62 (d, J = 8.3 Hz, 2H), 4.40 (s, 2H), 3.67 (s, 3H), 0.68 (s, 6H). **^{13}C{^{1}H} NMR (126 MHz, DMSO-d_6)** δ 192.4, 177.8, 169.3, 159.1, 147.6, 142.0, 137.1, 135.1, 128.8, 127.9, 127.7, 127.1, 127.0, 122.6, 122.5, 121.9, 121.2, 112.9, 112.1, 55.1, 50.0, 43.6, 24.2. **HRMS (ESI) m/z**: calculated for $C_{28}H_{26}N_3O_4$ $[M – H]^-$: 468.1929, found: 468.1927. HPLC purity: 95.4%, MeOH-H_2O (80:20).

(5ai) 2-(1-(3-amino-2,2-dimethyl-3-oxopropyl)-2-(3-methylphenyl)-1*H*-indole-3-carbonyl)benzamide

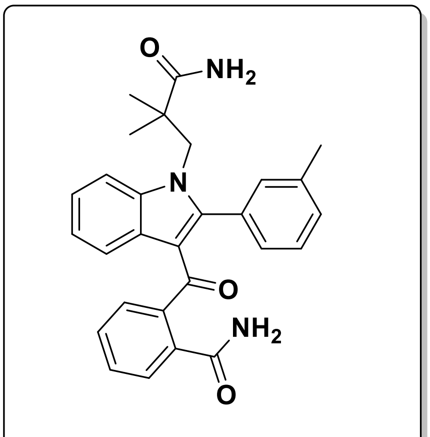

Following by general procedure for the synthesis of **5aa**. White solid, 56 mg, yield: 62%. **^{1}H NMR (600 MHz, DMSO-d_6)** δ 8.12 (d, J = 7.7 Hz, 1H), 7.74 (d, J = 8.2 Hz, 1H), 7.70 (s, 1H), 7.42 (d, J = 7.6 Hz, 1H), 7.26 (t, J = 7.4 Hz, 1H), 7.23 – 7.19 (m, 2H), 7.18 (s, 1H), 7.14 (d, J = 7.5 Hz, 1H), 7.13 – 7.09 (m, 2H), 7.05 (t, J = 7.4 Hz, 1H), 6.98 (t, J = 7.2 Hz, 1H), 6.96 – 6.91 (m, 2H), 6.83 (d, J = 7.2

Hz, 1H), 4.44 (s, 2H), 2.08 (s, 3H), 0.69 (s, 6H). **$^{13}C\{^1H\}$ NMR (151 MHz, DMSO-d_6)** δ 192.9, 178.3, 169.7, 148.1, 142.4, 137.6, 136.9, 135.4, 130.7, 129.4, 129.2, 128.4, 127.7, 127.4, 127.3, 123.1, 122.4, 121.8, 115.1, 112.6, 50.6, 44.0, 21.1. **HRMS (ESI) m/z**: calculated for $C_{28}H_{28}N_3O_3$ [M + H]$^-$: 454.2125, found: 454.2129. HPLC purity: 95.2%, MeOH-H_2O (80:20).

(5ak) 2-(1-(3-amino-2,2-dimethyl-3-oxopropyl)-2-(3-chlorophenyl)-1*H*-indole-3-carbonyl)benzamide

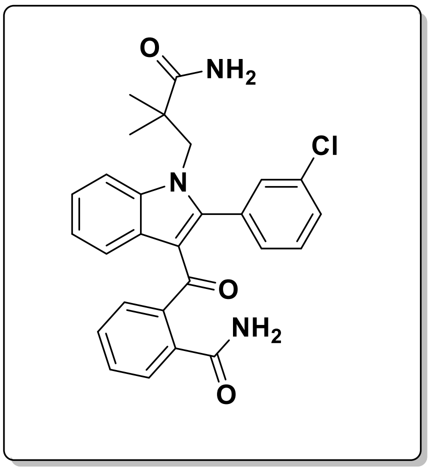

Following by general procedure for the synthesis of **5aa**. White solid, 67 mg, yield: 71%. **^{1}H NMR (600 MHz, DMSO-d_6)** δ ^{1}H NMR (600 MHz, DMSO) δ 8.06 (d, *J* = 7.9 Hz, 1H), 7.77 (d, *J* = 8.4 Hz, 2H), 7.49 (s, 1H), 7.47 (d, *J* = 7.7 Hz, 1H), 7.35 – 7.31 (m, 1H), 7.28 (t, *J* = 7.6 Hz, 1H), 7.24 (s, 1H), 7.23 – 7.18 (m, 4H), 7.11 (s, 1H), 7.04 (t, *J* = 7.4 Hz, 1H), 6.91 (s, 1H), 6.88 (d, *J* = 7.3 Hz, 1H), 4.41 (d, *J* = 72.9 Hz, 2H), 0.87 – 0.85 (m, 3H), 0.71 (s, 6H). **$^{13}C\{^1H\}$ NMR (151 MHz, DMSO-d_6)** δ 194.6, 179.9, 171.5, 147.8, 144.2, 139.6, 137.3, 134.8, 134.6, 131.5, 131.3, 130.7, 130.5, 129.5, 129.0, 125.3, 124.5, 123.8, 117.3, 114.7, 114.5, 52.6, 46.0. **HRMS (ESI) m/z**: calculated for $C_{28}H_{25}ClN_3O_3$ [M + H]$^-$: 474.1579, found: 474.1579. HPLC purity: 99.4%, MeOH-H_2O (80:20).

(5al) 2-(1-(3-amino-2,2-dimethyl-3-oxopropyl)-2-(thiophen-3-yl)-1*H*-indole-3-carbonyl)benzamide

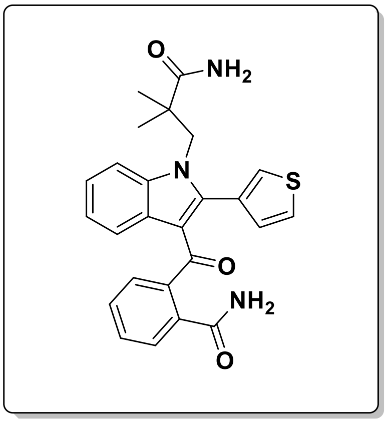

Following by general procedure for the synthesis of **5aa**. White solid, 66 mg, yield: 75%. **^{1}H NMR (400 MHz, DMSO-*d*$_6$)** δ 8.08 (d, *J* = 7.5 Hz, 1H), 7.80 (s, 1H), 7.74 (d, *J* = 8.3 Hz, 1H), 7.62 (dd, *J* = 2.9, 1.2 Hz, 1H), 7.43 (dd, *J* = 7.7, 1.2 Hz, 1H), 7.32 – 7.18 (m, 5H), 7.15 – 7.03 (m, 3H), 6.94 (s, 1H), 6.88 (dd, *J* = 7.6, 1.2 Hz, 1H), 4.47 (s, 2H), 0.71 (s, 6H). **^{13}C{^{1}H} NMR (126 MHz, DMSO-*d*$_6$)** δ 192.2, 177.7, 169.7, 142.7, 141.7, 137.3, 135.5, 130.5, 129.7, 128.7, 128.2, 127.4, 127.2, 126.9, 125.4, 122.8, 122.0, 121.2, 114.9, 112.0, 50.3, 43.6, 24.0. **HRMS (ESI) m/z**: calculated for $C_{25}H_{22}N_3O_3S$ [M - H]$^-$: 444.1387, found: 444.1389.、

(5am) 2-(1-(3-amino-2,2-dimethyl-3-oxopropyl)-5,6-dichloro-2-phenyl-1*H*-indole-3-carbonyl)benzamide

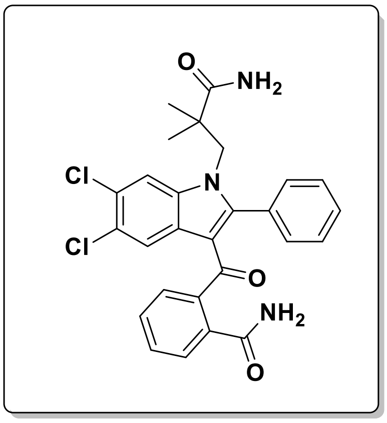

Following by general procedure for the synthesis of **5aa**. White solid, 62 mg, yield: 61%. **^{1}H NMR (500 MHz, DMSO-*d*$_6$)** δ 8.24 (d, *J* = 7.1 Hz, 1H), 7.90 (s, 1H), 7.75 (d, *J* = 7.7 Hz, 1H), 7.54 (s, 1H), 7.42 (s, 1H), 7.34 – 7.25 (m, 4H), 7.21 – 7.12 (m, 3H), 7.09 (s, 1H), 6.98 (s, 1H), 6.91 (s, 1H), 4.39 (s, 2H), 0.68 (s, 6H). **^{13}C{^{1}H} NMR (126 MHz, DMSO-*d*$_6$)** δ 177.7, 167.0, 148.1, 141.5, 137.3, 135.2, 131.6, 131.4, 130.5, 130.3, 129.9, 129.0, 128.3, 127.4, 126.8, 123.1, 122.4, 121.4, 114.5, 112.3, 50.3, 43.5, 24.2. **HRMS (ESI) m/z**: calculated for $C_{27}H_{22}Cl_2N_3O_3$ [M - H]$^-$: 506.1044, found: 506.1048.

VIII. X-ray Crystallographic Data

Single Crystal X-ray Diffraction (SCXRD): X-ray diffractions of all single crystals were carried out at 100(2) K on a Bruker D8 VENTURE diffractometer using Mo-Kα radiation (λ = 0.71073 Å). Integration and scaling of intensity data was performed using the SAINT program. Data were corrected for the effects of absorption using SADABS. The structures were solved by direct method and refined with full-matrix least-squares technique using SHELX-2014 software. Non-hydrogen atoms were refined with anisotropic displacement parameters, and hydrogen atoms were placed in calculated positions and refined with a riding model.

(1) The Single Crystal Structure of 3aa (Deposition Data: CCDC 2410303)

Crystal Data for $C_{54}H_{52}N_6O_5$ (M = 865.01 g/mol): monoclinic, space group $P2_1/c$ (no. 14), a = 10.9888(2) Å, b =18.4115(5) Å, c =12.1884(3) Å, α =90°, β = 109.3740(10)°, γ = 90°, V = 2326.32(10) Å^3, Z = 2, T = 170(2) K, μ(CuKα) = 0.640 mm^{-1}, Dcalc = 1.235 g/cm^3, 22292 reflections measured (8.53° ≤ 2Θ ≤ 149.236°), 4691 unique (R_{int} = 0.0599, R_{sigma} = 0.0412) which were used in all calculations. The final R_1 was 0.1018 (I > 2σ(I)) and wR_2 was 0.2367 (all data).

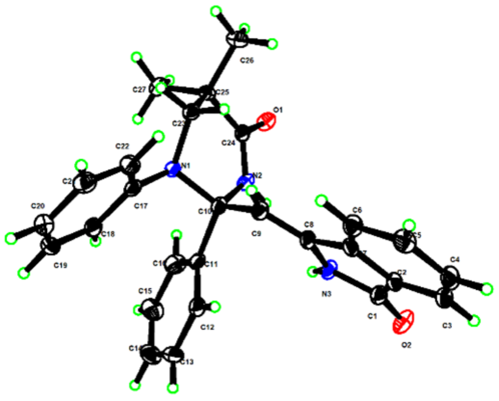

(2) The Single Crystal Structure of 4aa (Deposition Data: CCDC 2410625)

Crystal Data for $C_{55}H_{48}Cl_2N_6O_4$ (M = 927.89 g/mol): monoclinic, space group Pn (no. 7), a = 14.0724(3) Å, b =41.3599(10) Å, c =16.7154(4) Å, α = 90°, β = 102.8130(10)°, γ = 90°, V = 9486.7(4) Å^3, Z = 8, T = 170(2) K, μ(CuKα) = 1.662 mm^{-1}, Dcalc = 1.299 g/cm^3, 150635 reflections measured (4.272° ≤ 2Θ ≤ 149.836°), 36461 unique (R_{int} =

0.0557, R_{sigma} = 0.0449) which were used in all calculations. The final R_1 was 0.0526 (I > 2σ(I)) and wR_2 was 0.1531 (all data).

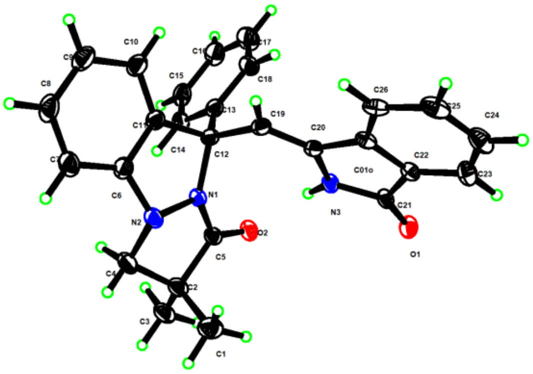

(3) The Single Crystal Structure of 5aa (Deposition Data: CCDC 2410305)

Crystal Data for $C_{27}H_{25}N_3O_3$ (M = 439.50 g/mol): orthorhombic, space group Pbca (no. 61), a = 19.2491(12) Å, b = 9.9787(4) Å, c =22.4268(14) Å, α = 90°, β = 90°, γ = 90°, V = 4307.8(4) $Å^3$, Z = 8, T = 170.00 K, μ(MoKα) = 0.720 mm^{-1}, Dcalc = 1.355 g/cm^3, 18845 reflections measured (7.884° ≤ 2Θ ≤ 127.572°), 3488 unique (R_{int} = 0.0995, R_{sigma} = 0.0666) which were used in all calculations. The final R_1 was 0.0590 (I > 2σ(I)) and wR_2 was 0.1477 (all data).

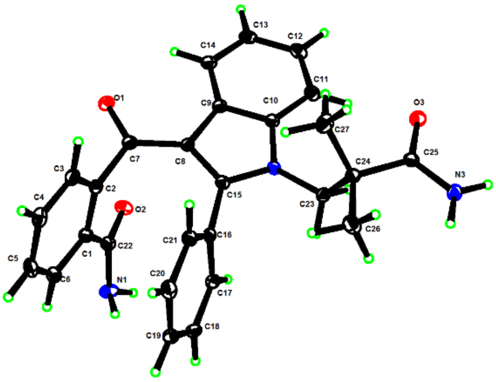

IX. NMR Spectra of Products

(*Z/E*)-3-((5,5-dimethyl-4-oxo-1,2-diphenylhexahydropyrimidin-2-yl)methylene)isoindolin-1-one (3aa)

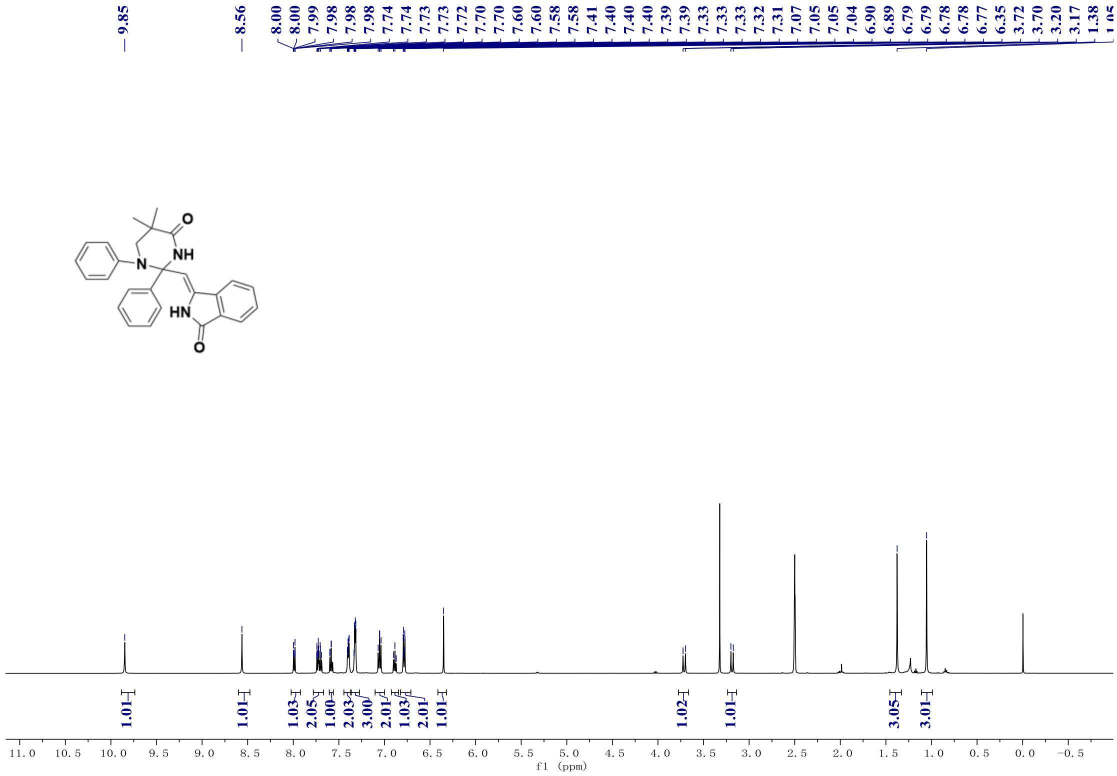

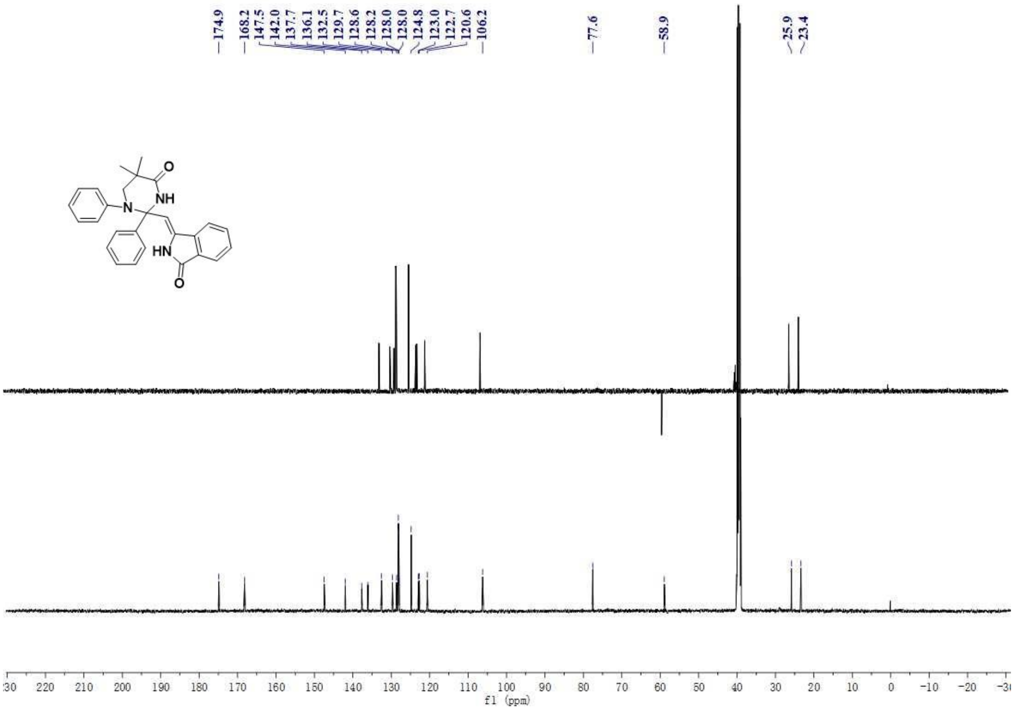

(*Z/E*)-3-((1-(4-fluorophenyl)-5,5-dimethyl-4-oxo-2-phenylhexahydropyrimidin-2-yl) methylene)isoindolin-1-one (3ba)

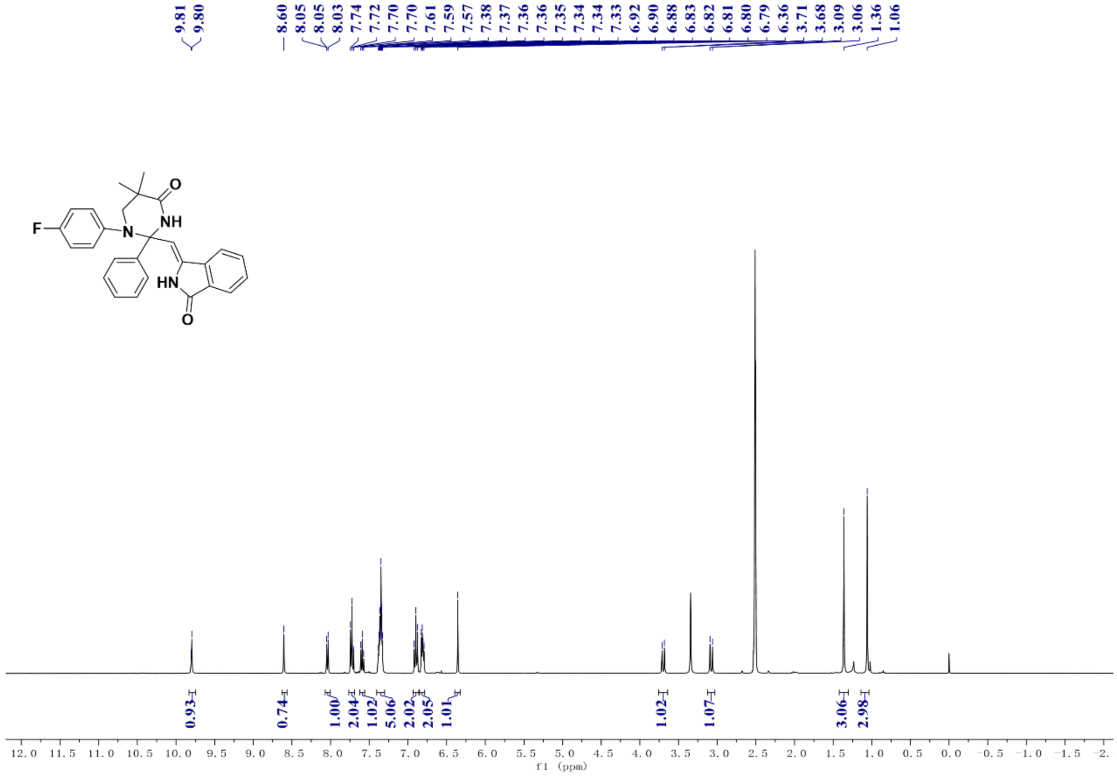

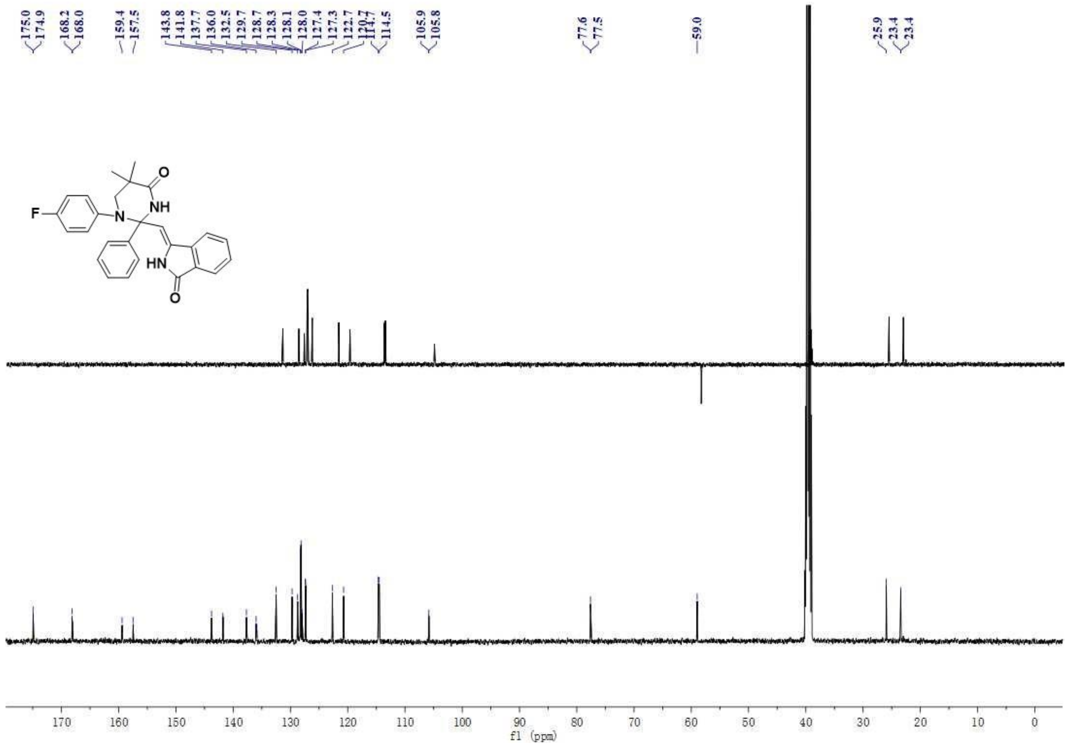

(*Z/E*)-3-((1-(4-chlorophenyl)-5,5-dimethyl-4-oxo-2-phenylhexahydropyrimidin-2-yl)methylene)isoindolin-1-one (3ca)

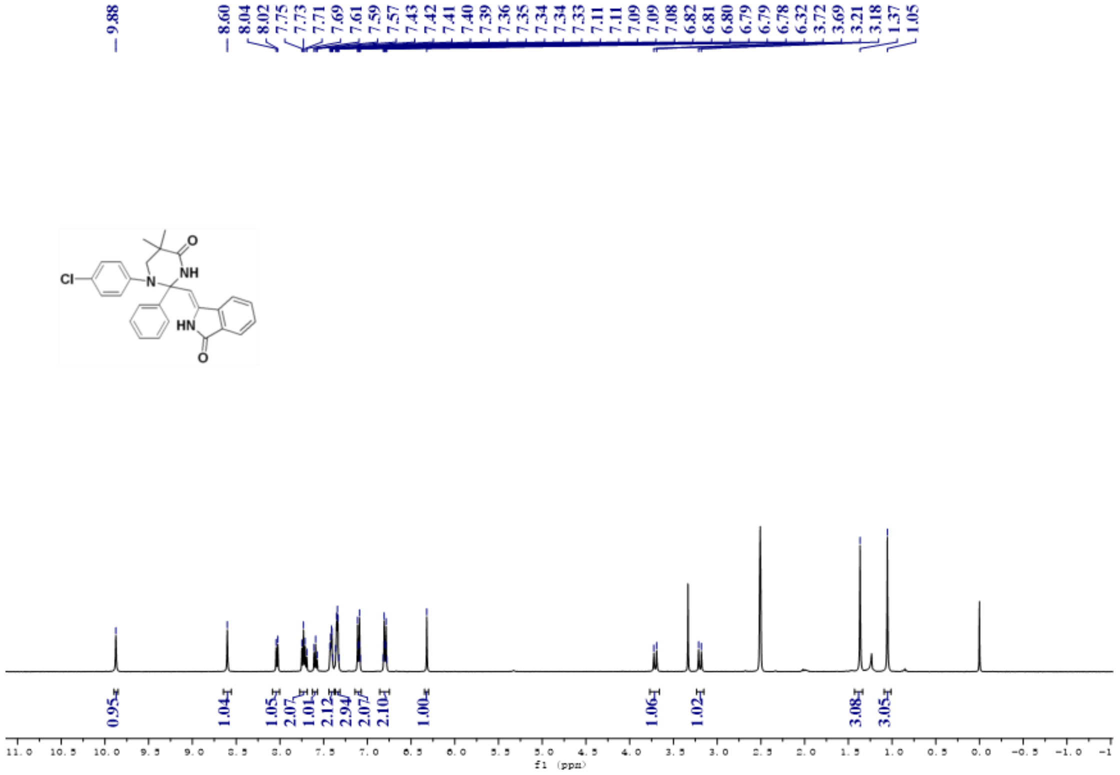

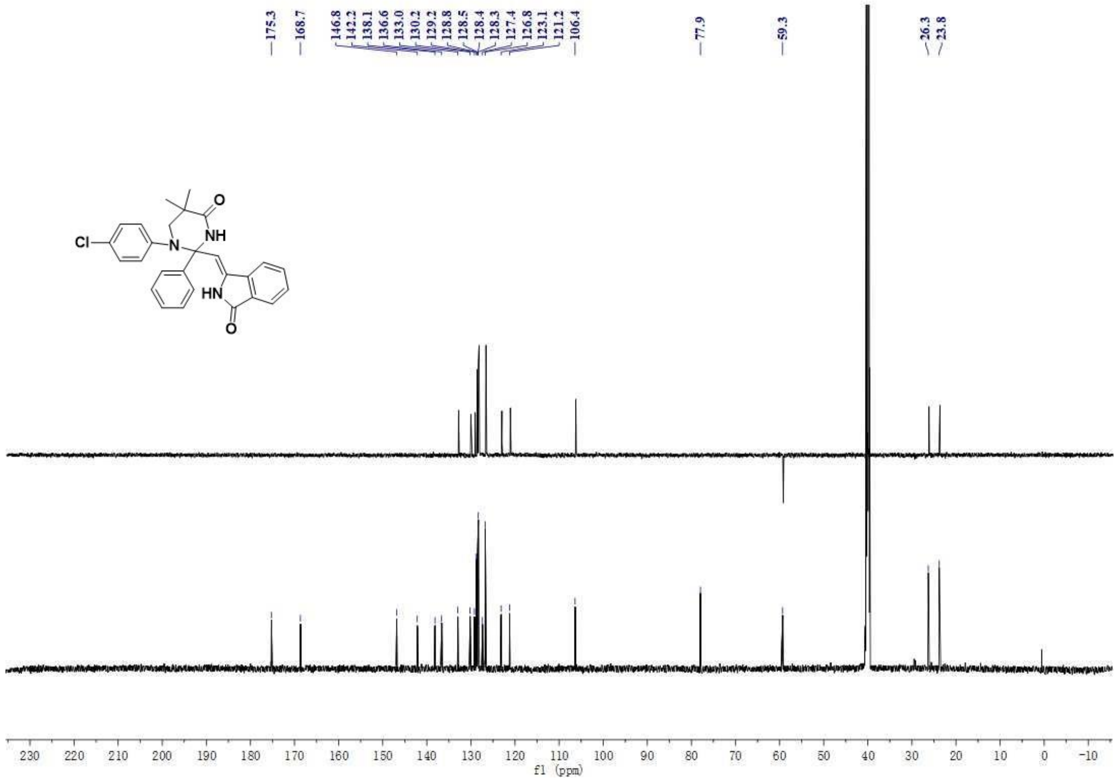

(*Z/E*)-3-((1-(4-bromophenyl)-5,5-dimethyl-4-oxo-2-phenylhexahydropyrimidin-2-yl)methylene)isoindolin-1-one (3da)

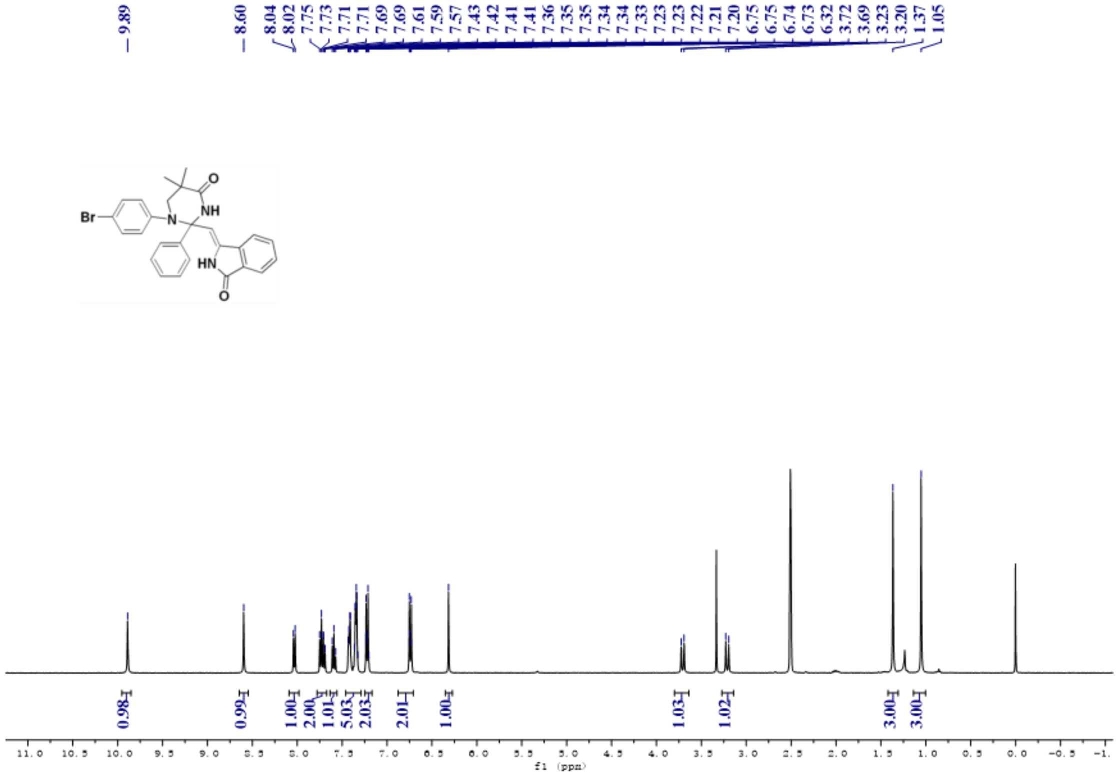

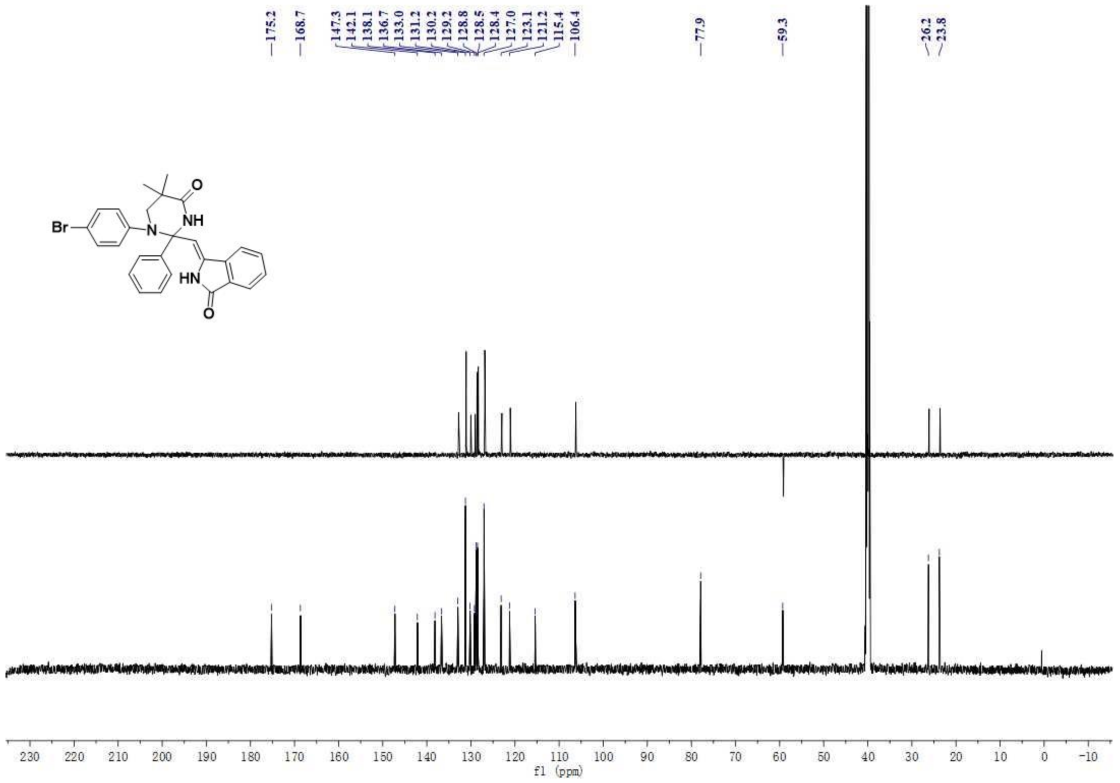

(*Z/E*)-3-((5,5-dimethyl-4-oxo-2-phenyl-1-(*p*-tolyl)hexahydropyrimidin-2-yl)methylene)isoindolin-1-one (3ea)

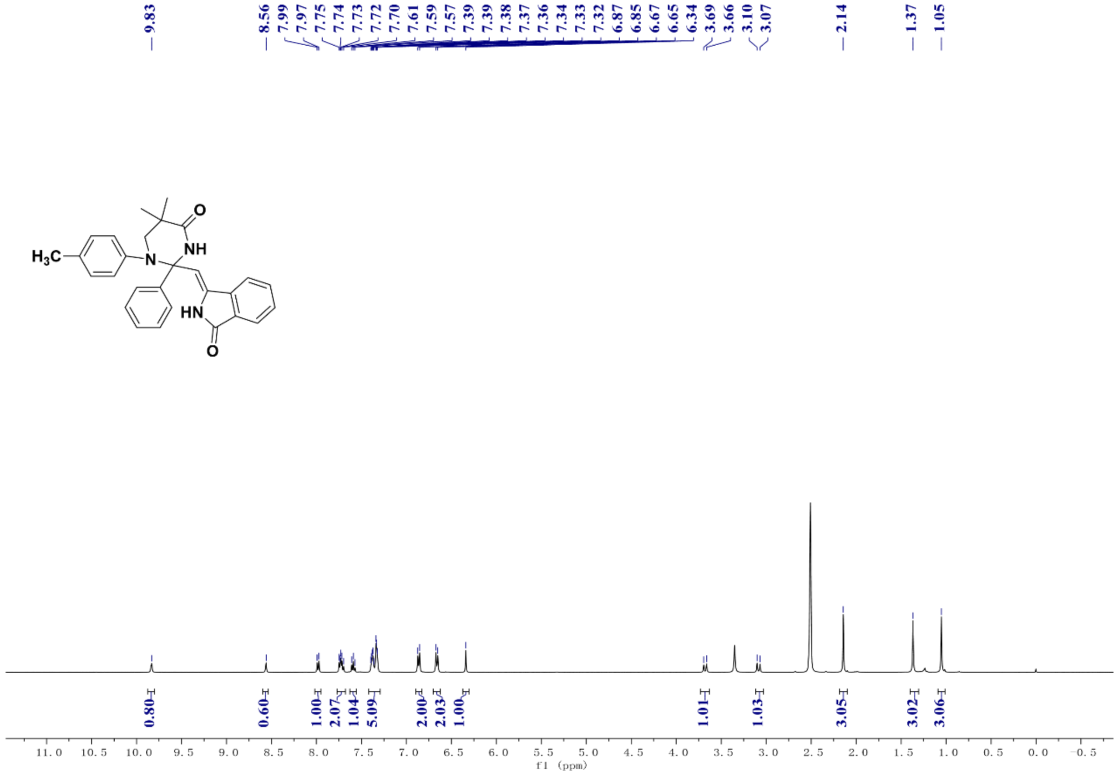

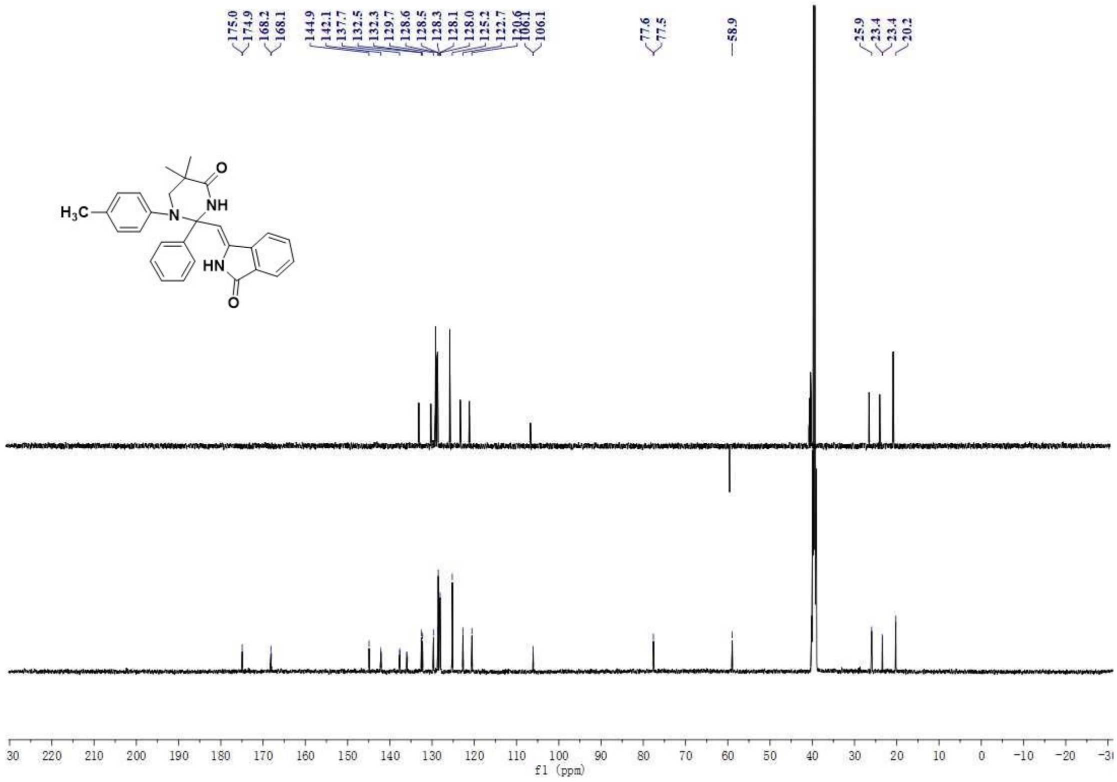

(*Z/E*)-3-((1-(4-methoxyphenyl)-5,5-dimethyl-4-oxo-2-phenylhexahydropyrimidin-2-yl)methylene)isoindolin-1-one (3fa)

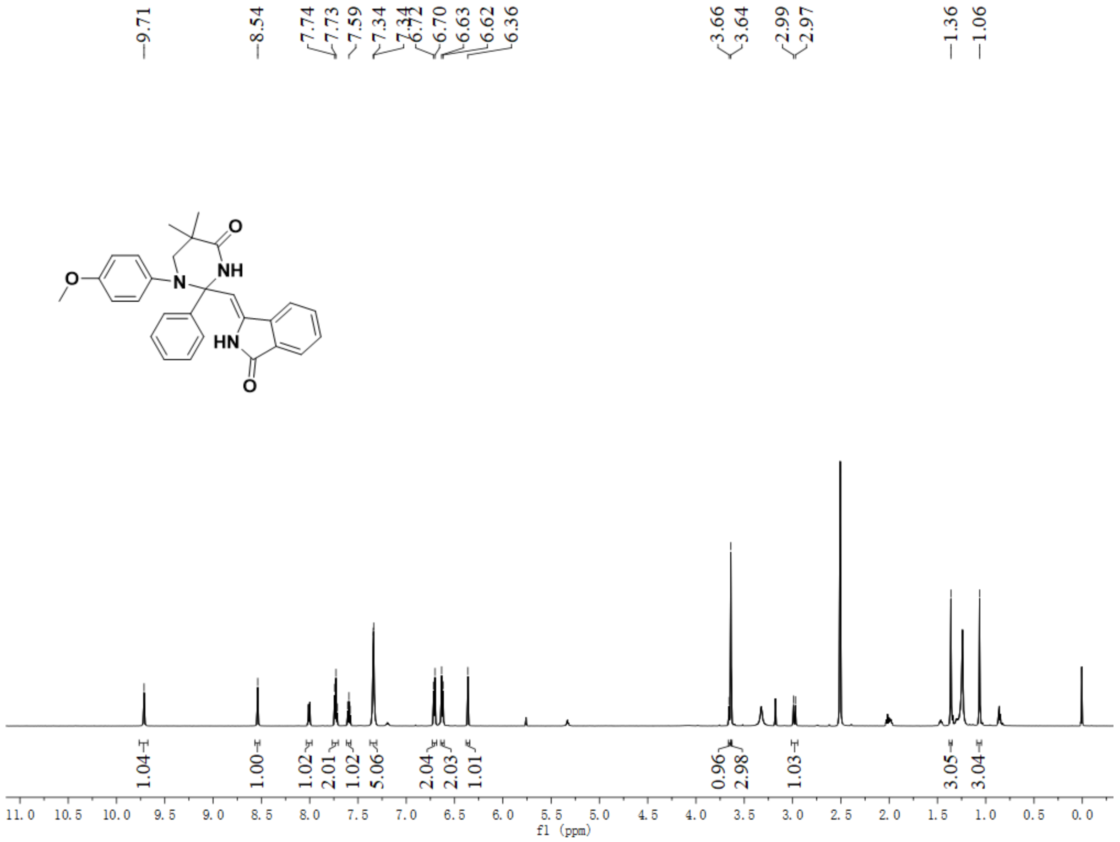

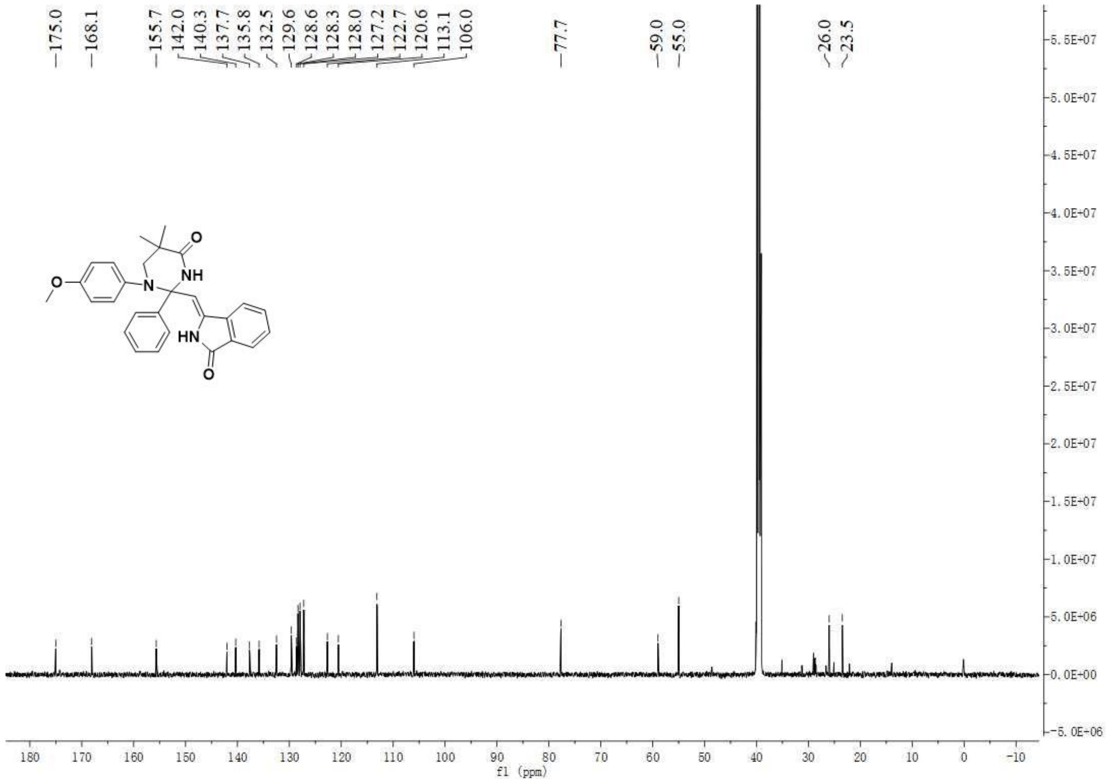

(*Z/E*)-3-((1-(3-fluorophenyl)-5,5-dimethyl-4-oxo-2-phenylhexahydropyrimidin-2-yl)methylene)isoindolin-1-one (3ia)

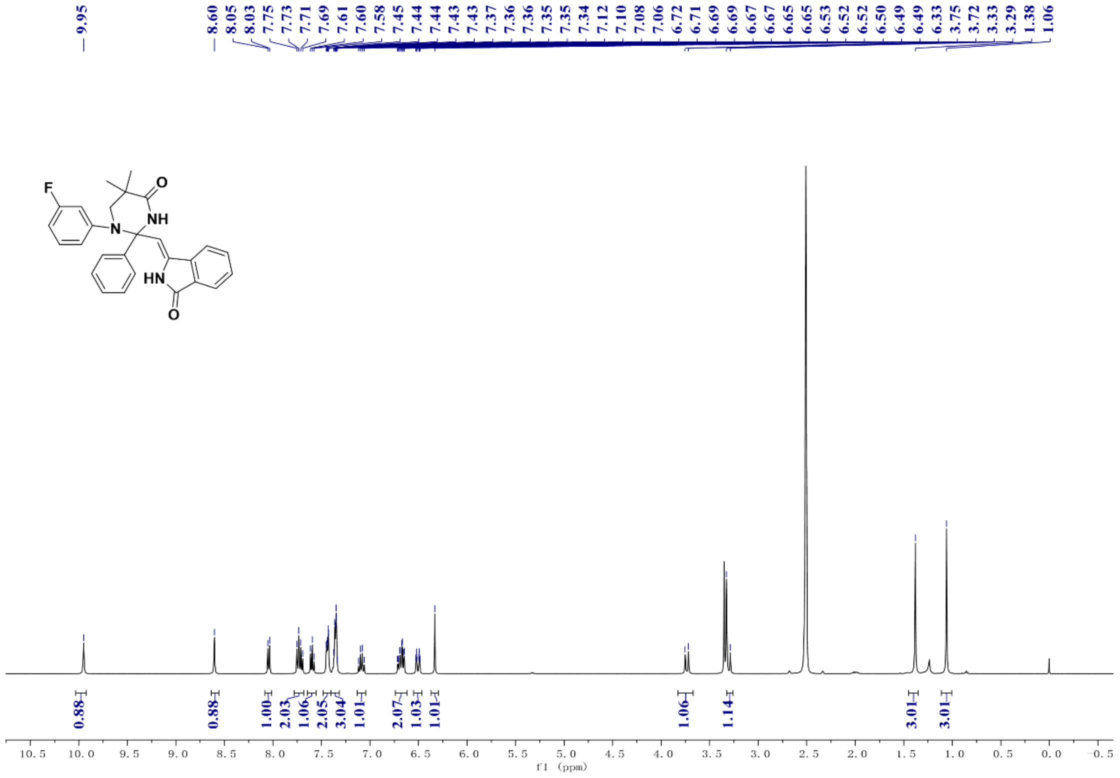

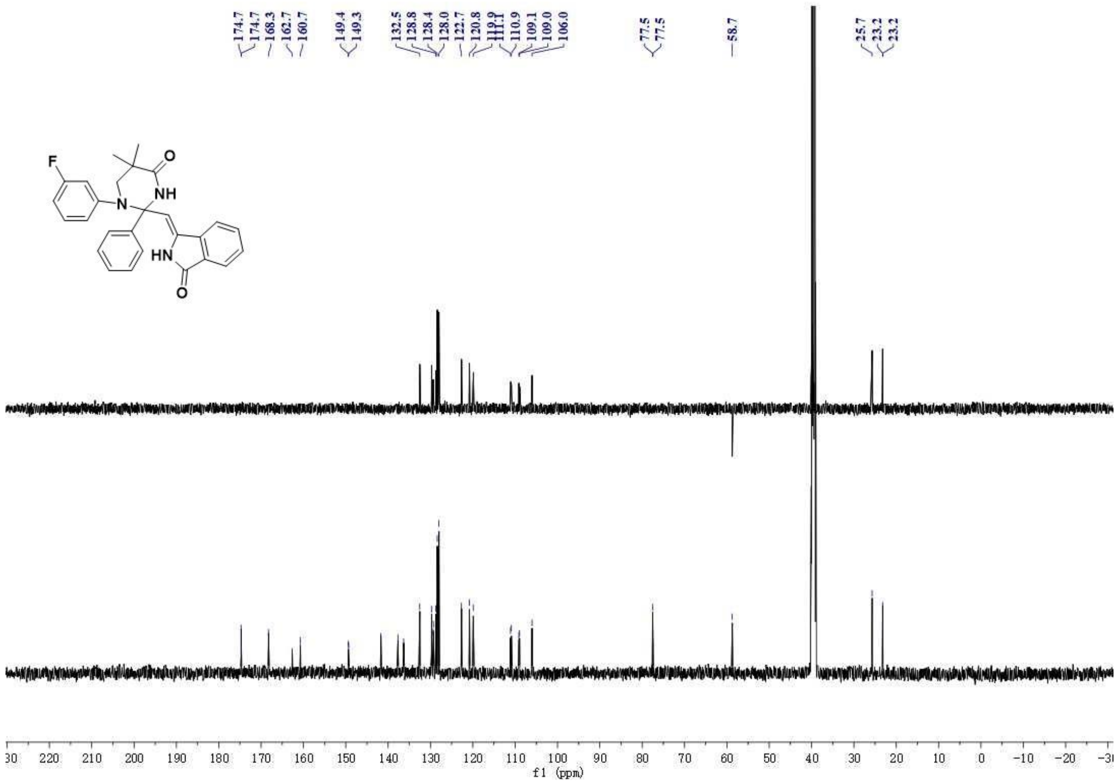

(*Z/E*)-3-((2-(4-fluorophenyl)-5,5-dimethyl-4-oxo-1-phenylhexahydropyrimidin-2-yl)methylene)isoindolin-1-one (3ab)

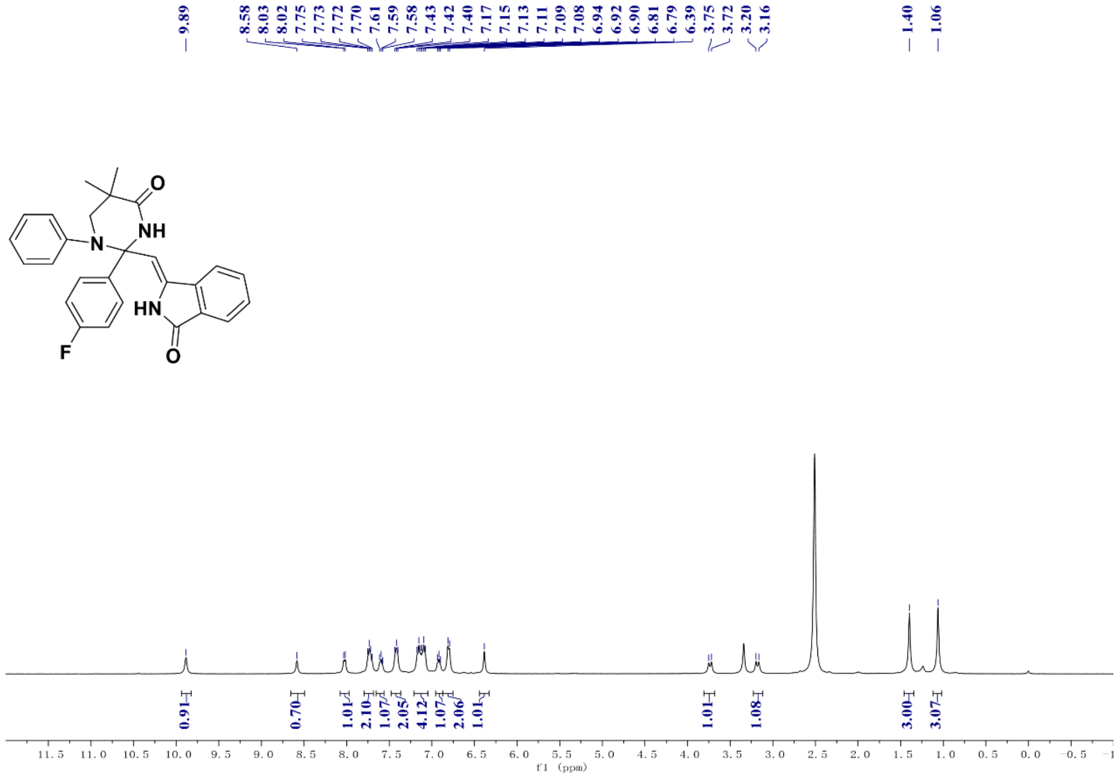

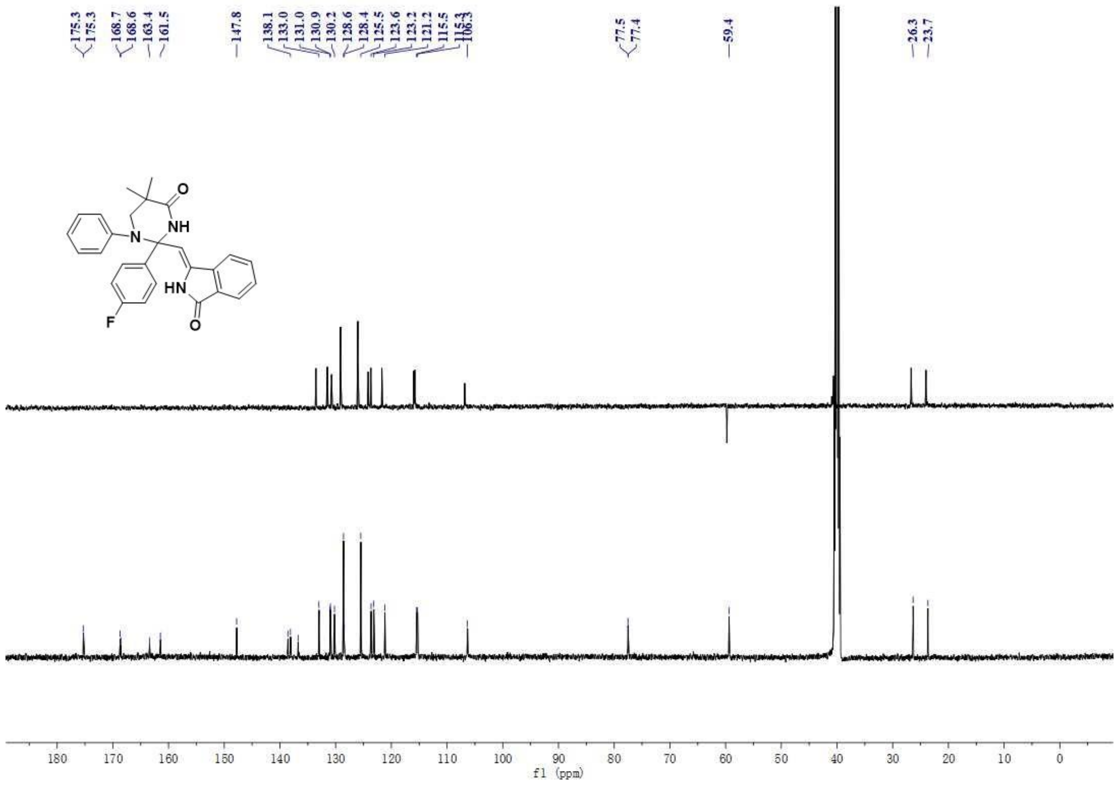

(*Z/E*)-3-((2-(4-chlorophenyl)-5,5-dimethyl-4-oxo-1-phenylhexahydropyrimidin-2-yl)methylene)isoindolin-1-one (3ac)

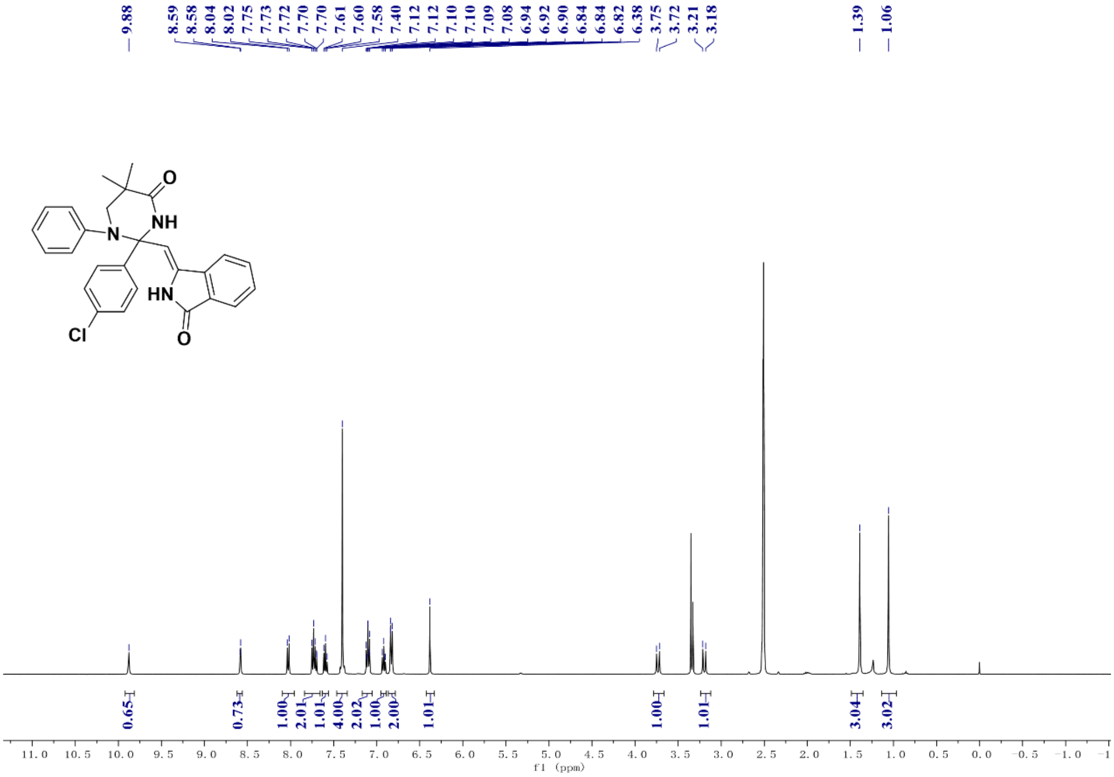

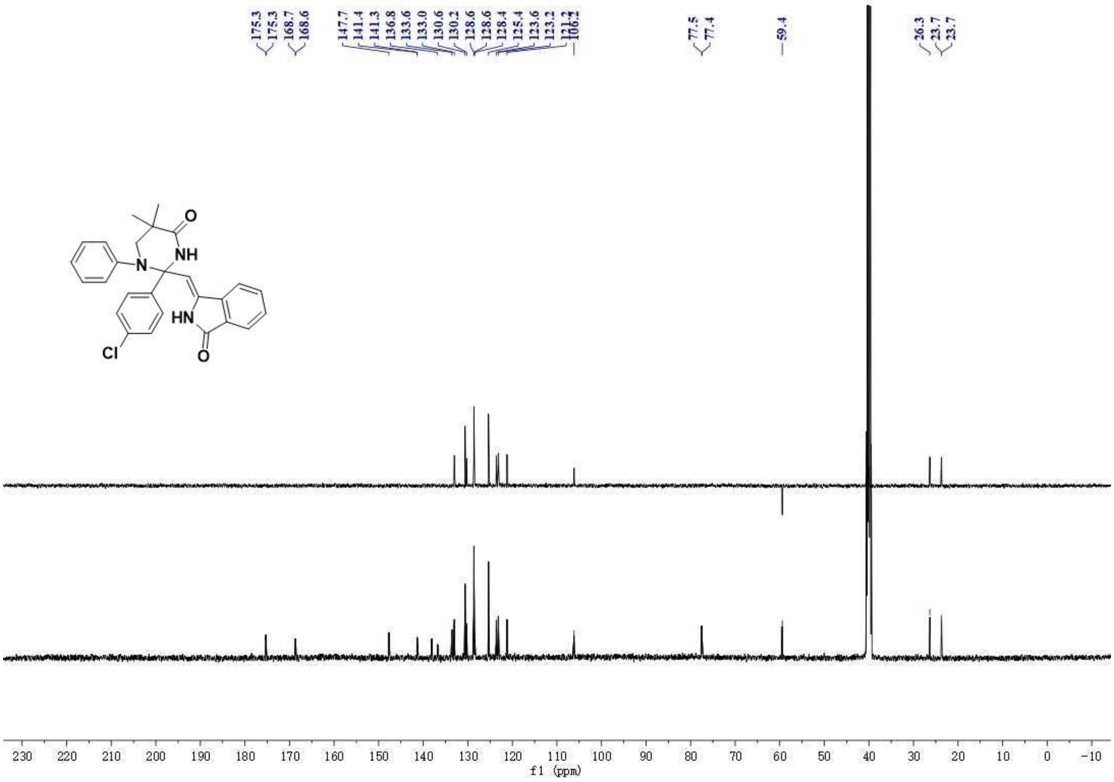

(*Z/E*)-3-((2-(4-bromophenyl)-5,5-dimethyl-4-oxo-1-phenylhexahydropyrimidin-2-yl)methylene)isoindolin-1-one (3ad)

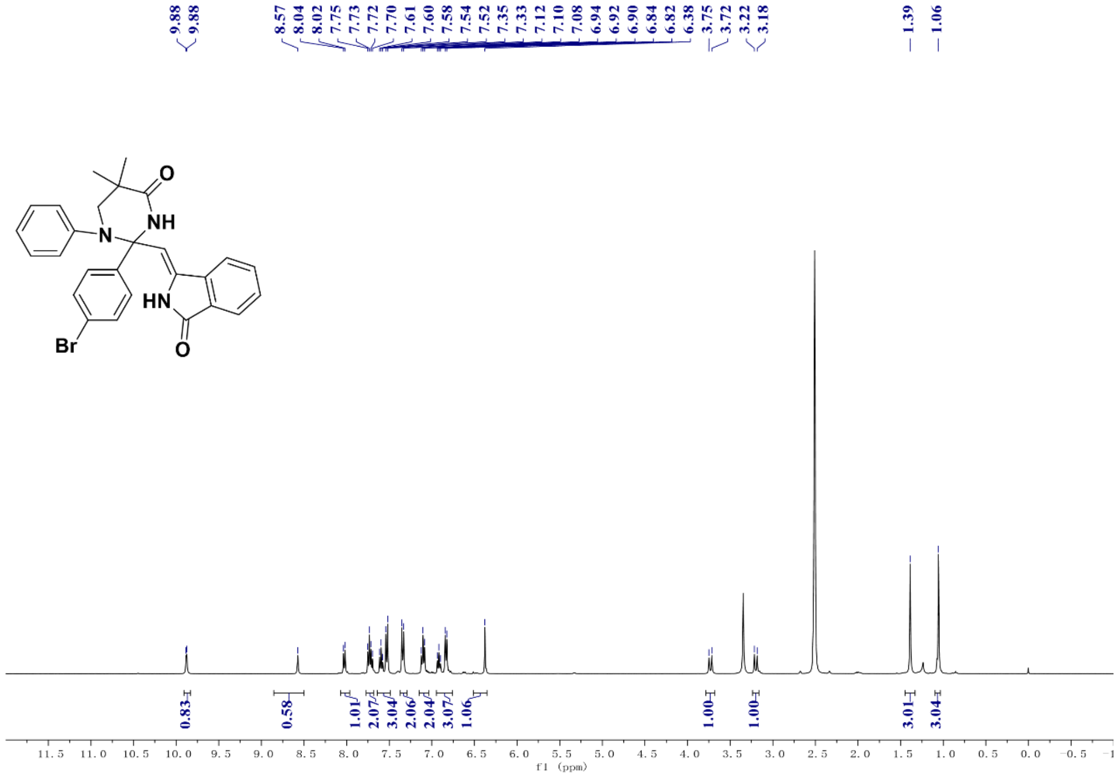

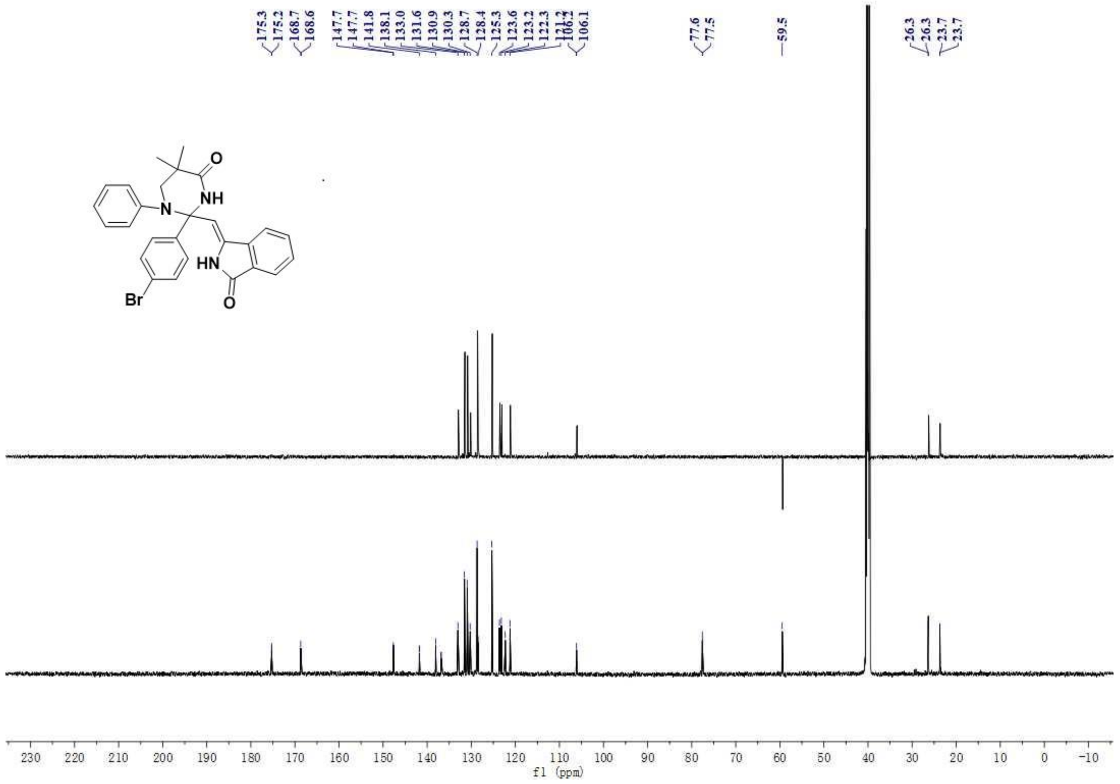

(*Z/E*)-3-((5,5-dimethyl-4-oxo-1-phenyl-2-(p-tolyl)hexahydropyrimidin-2-yl)methylene)isoindolin-1-one (3ae)

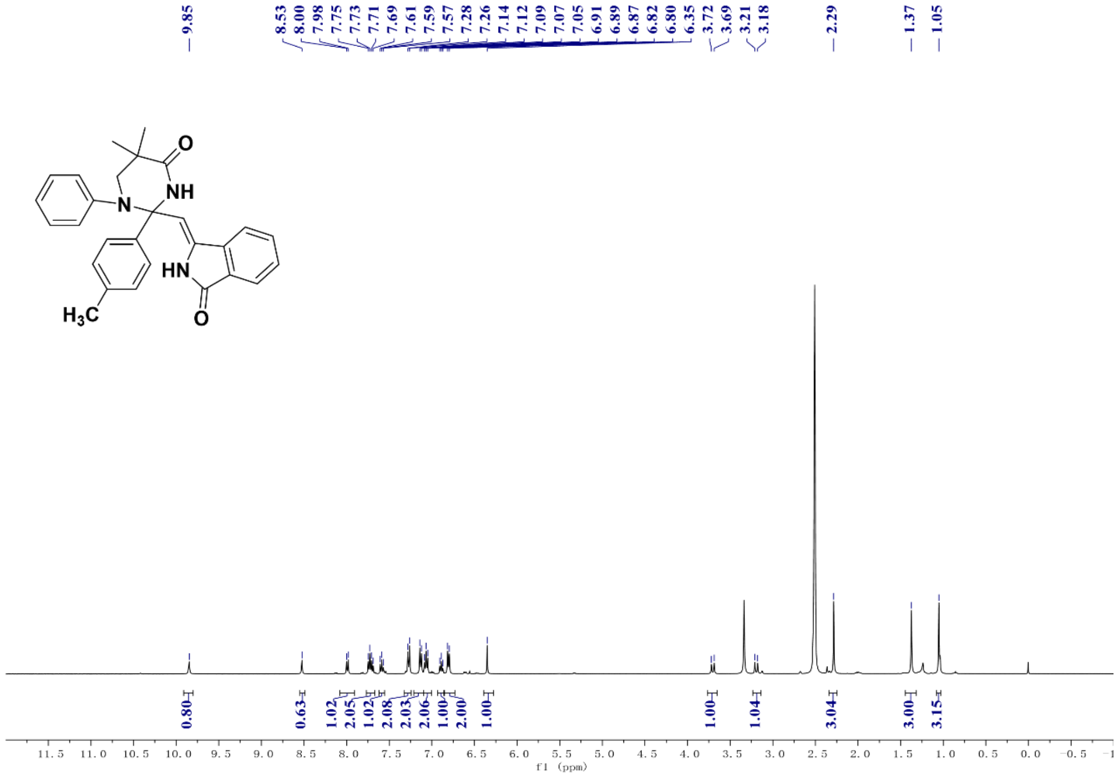

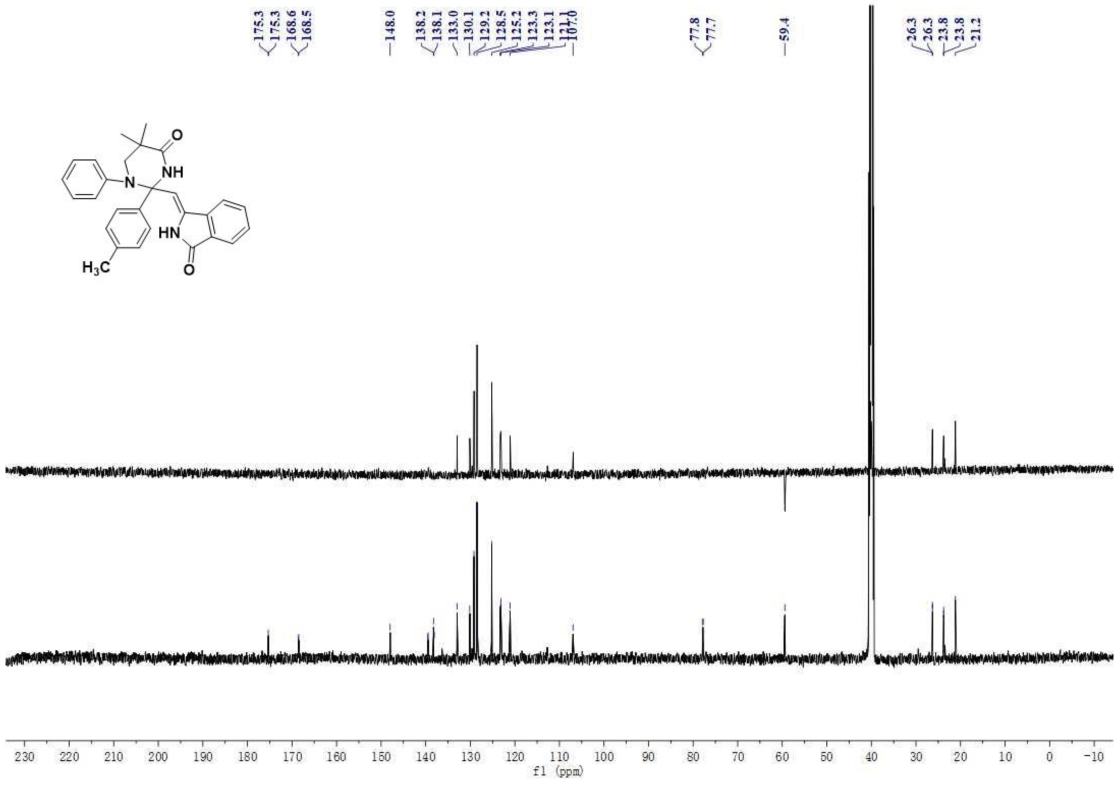

(*Z/E*)-3-((2-(4-methoxyphenyl)-5,5-dimethyl-4-oxo-1-phenylhexahydropyrimidin-2-yl)methylene)isoindolin-1-one (3af)

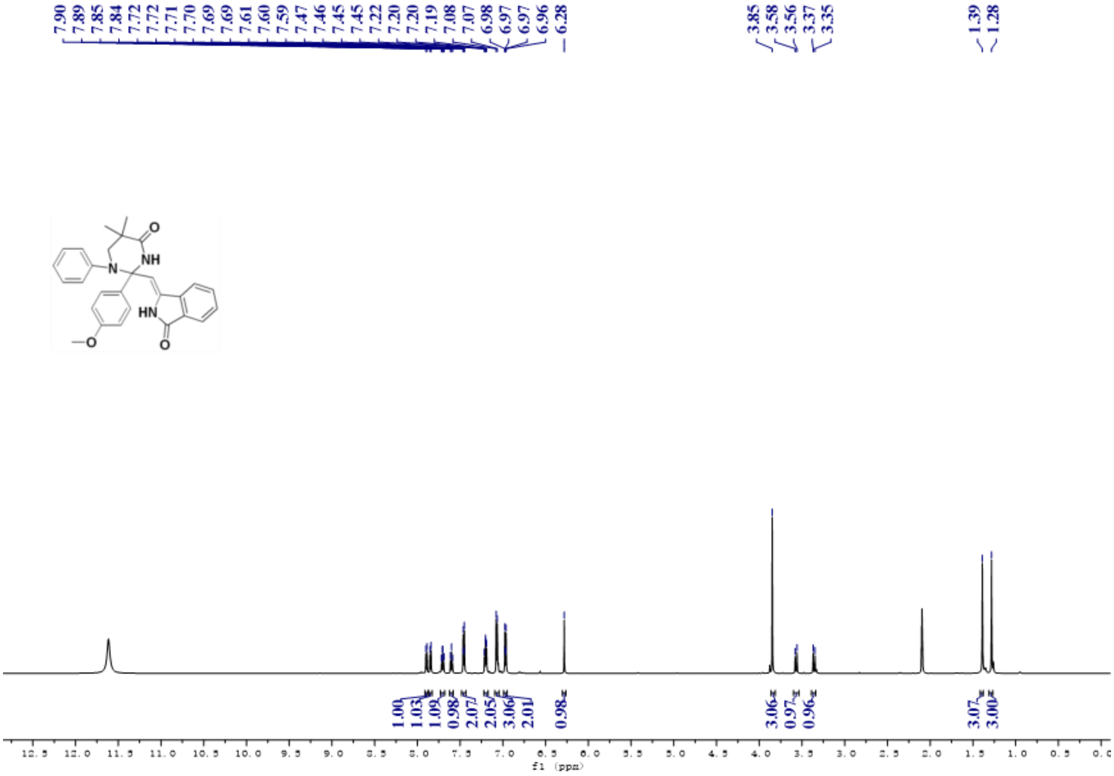

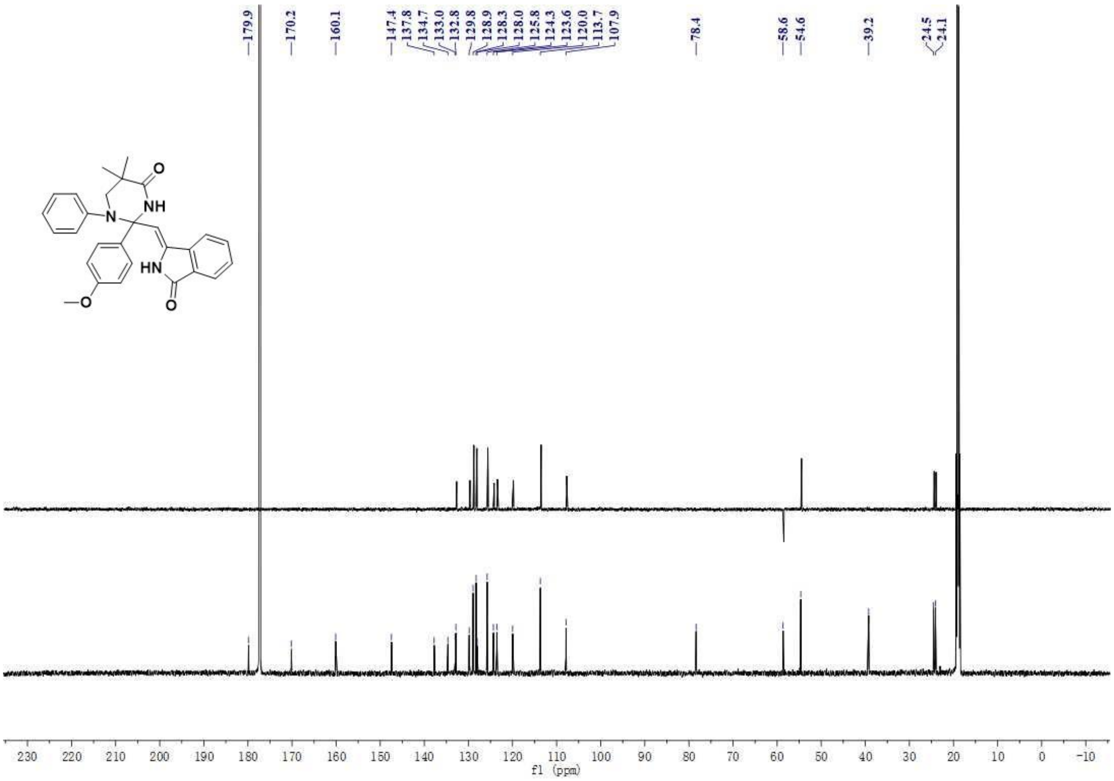

(*Z/E*)-3-((5,5-dimethyl-4-oxo-2-(4-pentylphenyl)-1-phenylhexahydropyrimidin-2-yl)methylene)isoindolin-1-one (3ag)

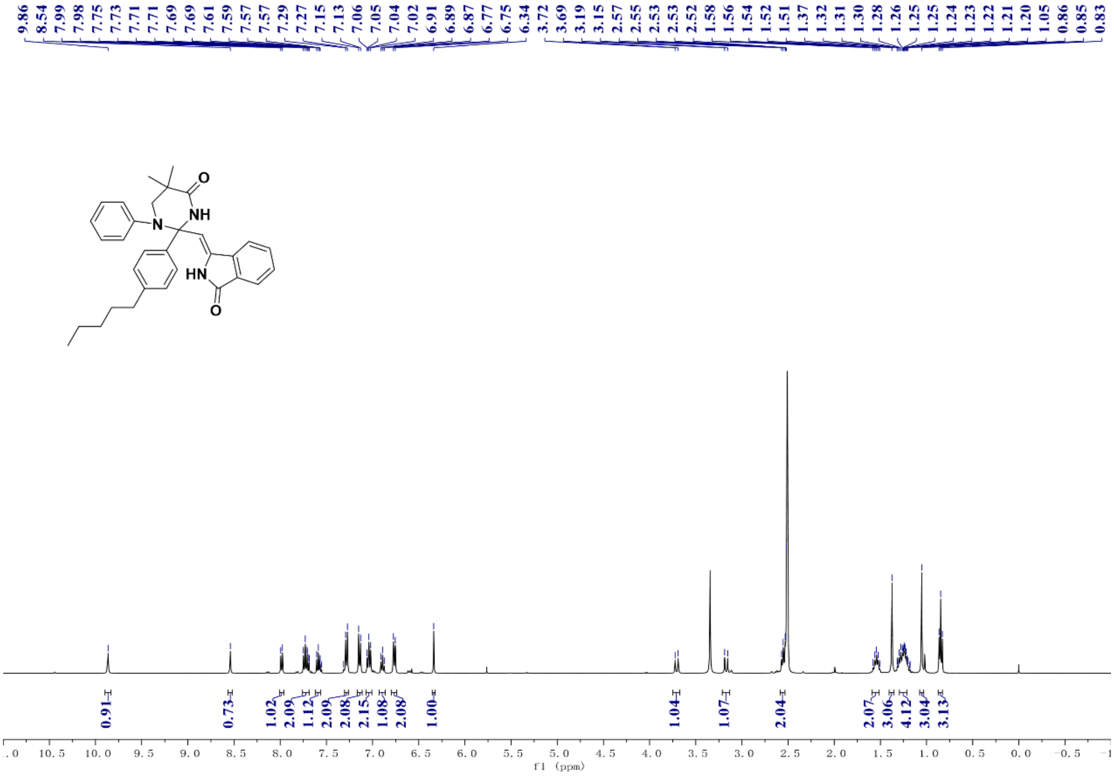

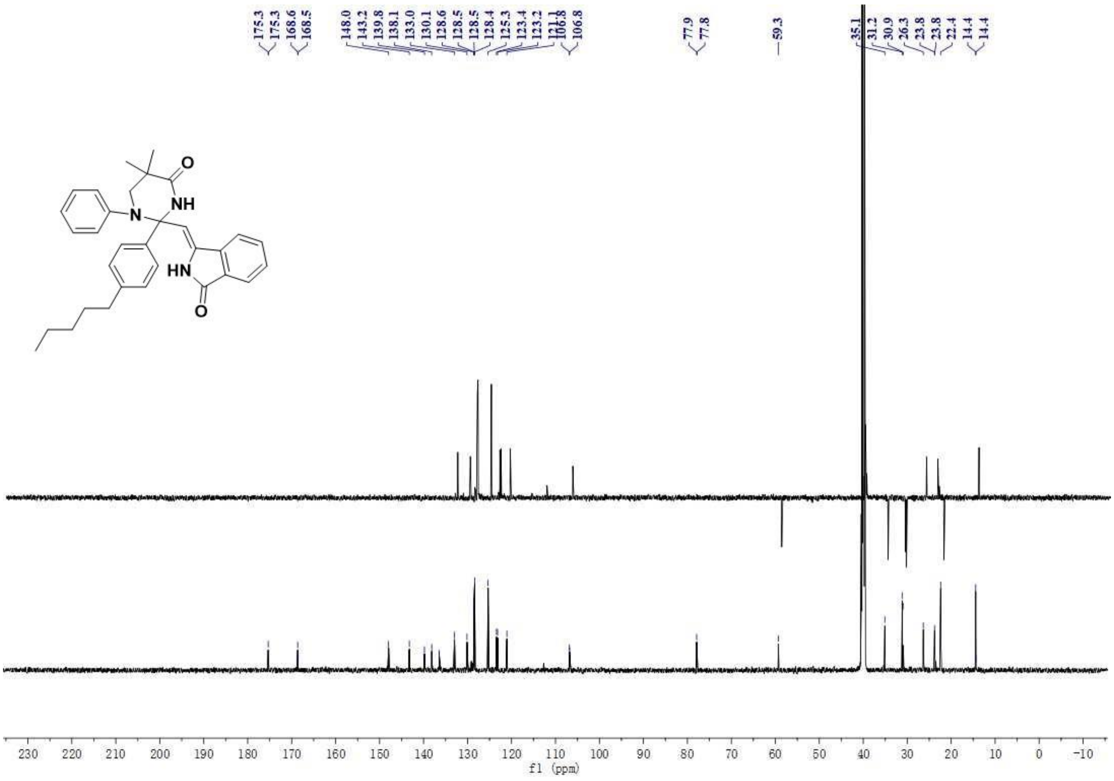

(*Z/E*)-3-((5,5-dimethyl-4-oxo-1-phenyl-2-(m-tolyl)hexahydropyrimidin-2-yl)methylene)isoindolin-1-one (3ai)

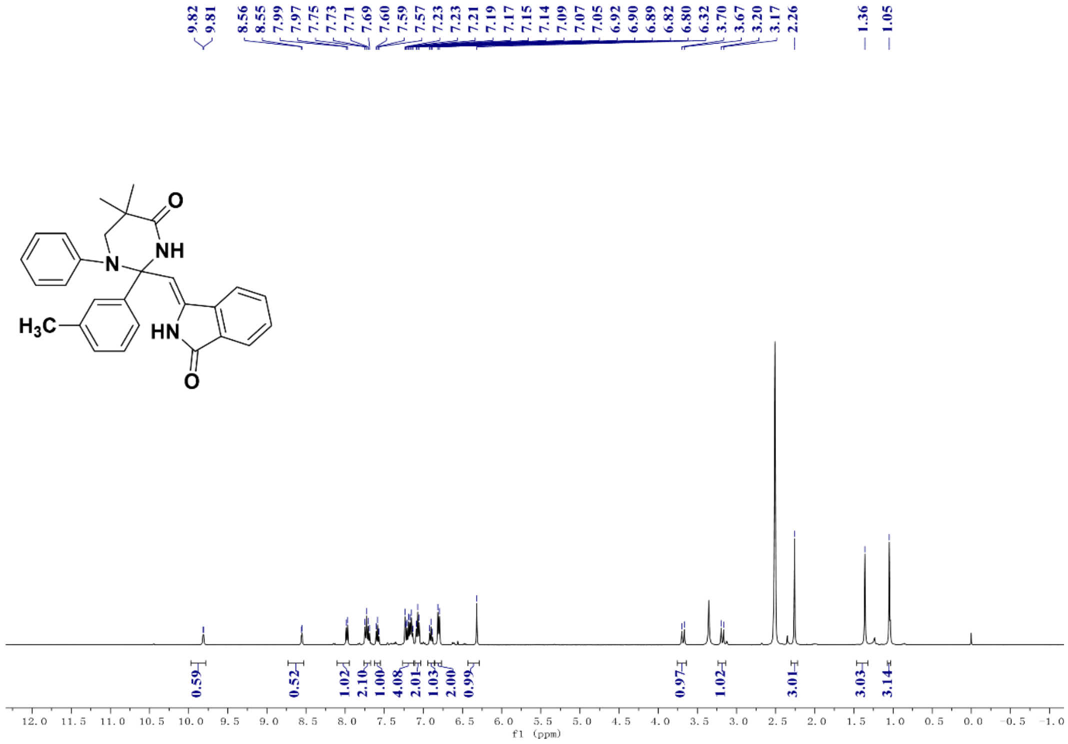

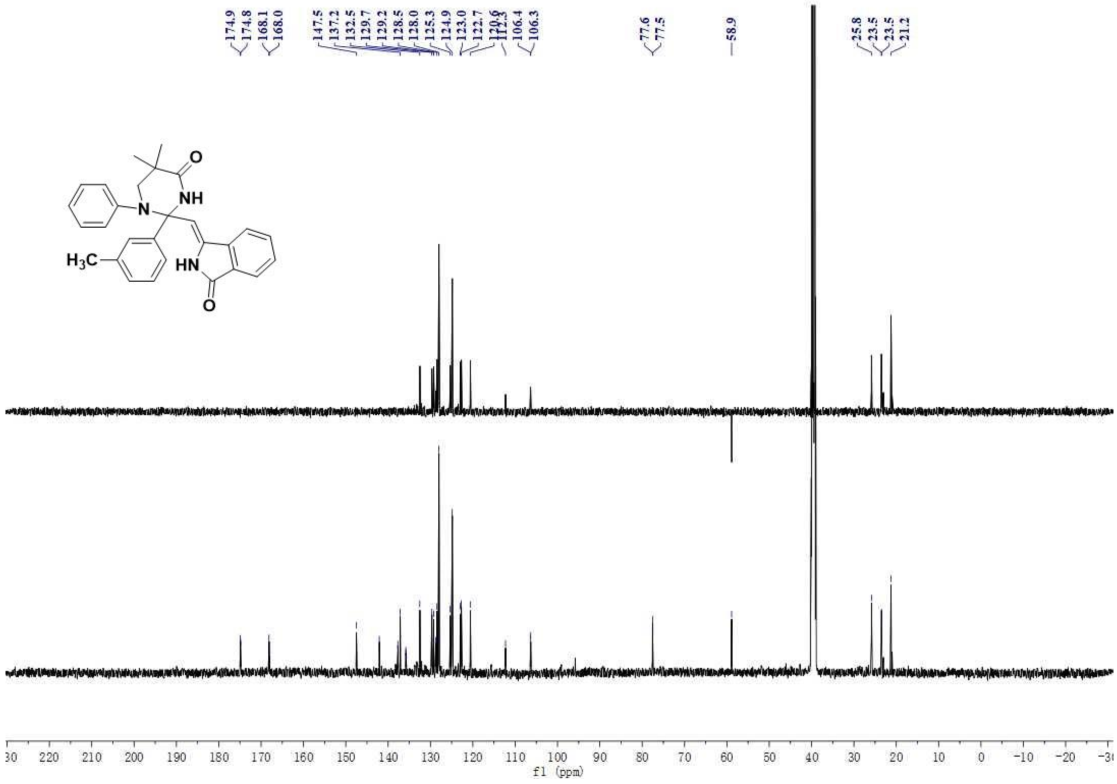

(*Z/E*)-3-((2-(3-chlorophenyl)-5,5-dimethyl-4-oxo-1-phenylhexahydropyrimidin-2-yl)methylene)isoindolin-1-one (3ak)

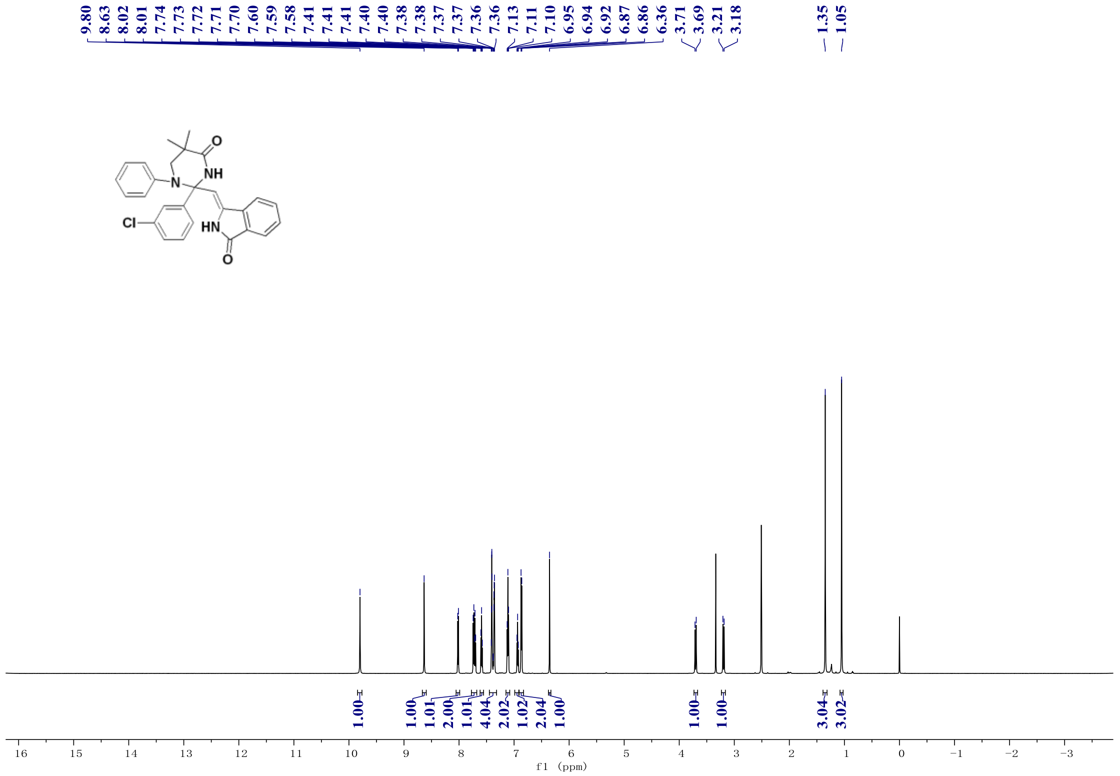

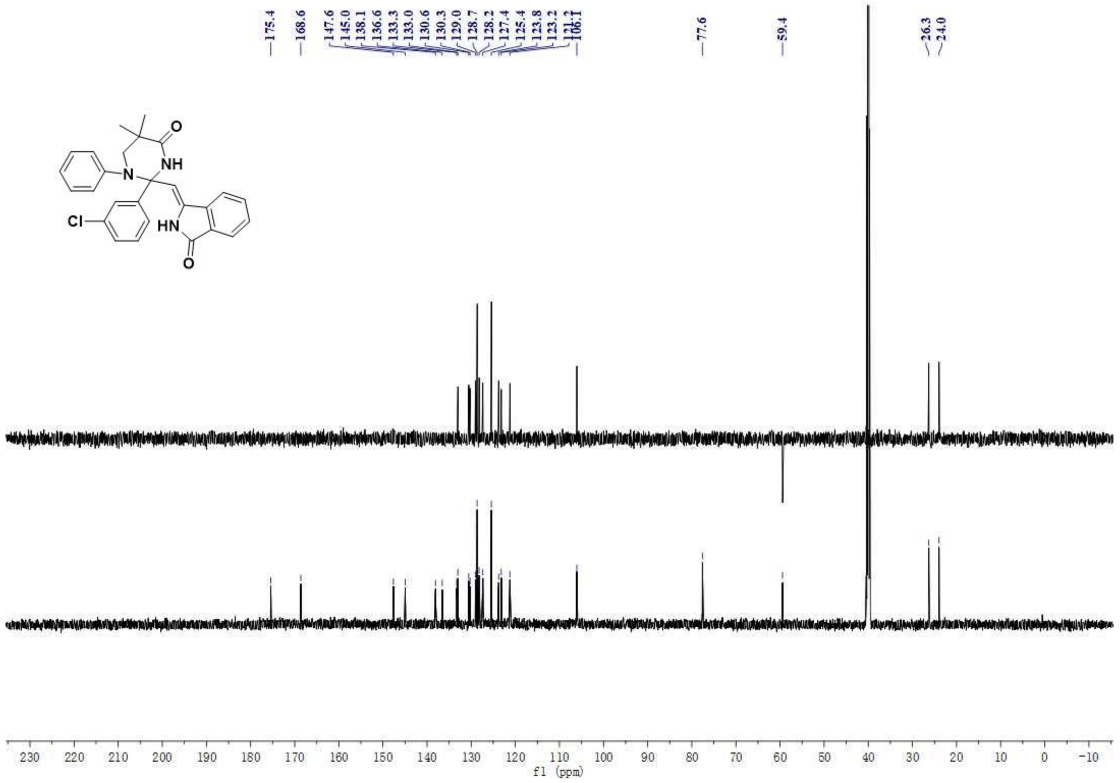

(*Z/E*)-3-((5,5-dimethyl-4-oxo-1-phenyl-2-(thiophen-3-yl)hexahydropyrimidin-2-yl)methylene)isoindolin-1-one (3al)

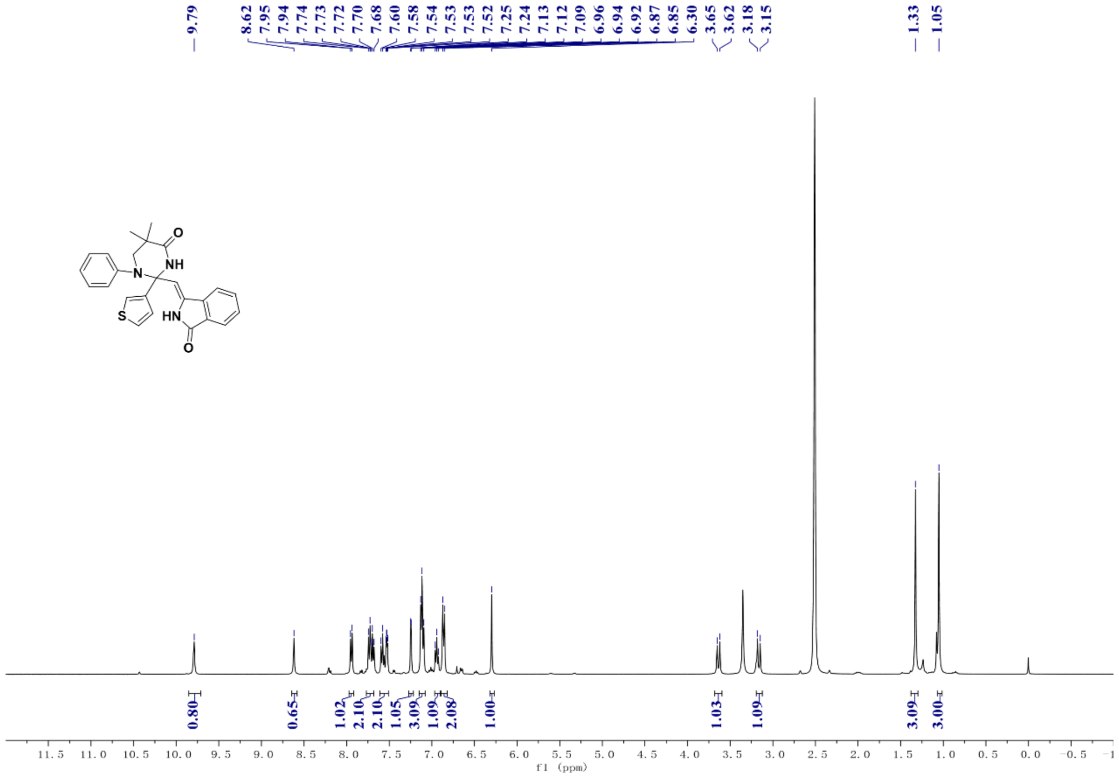

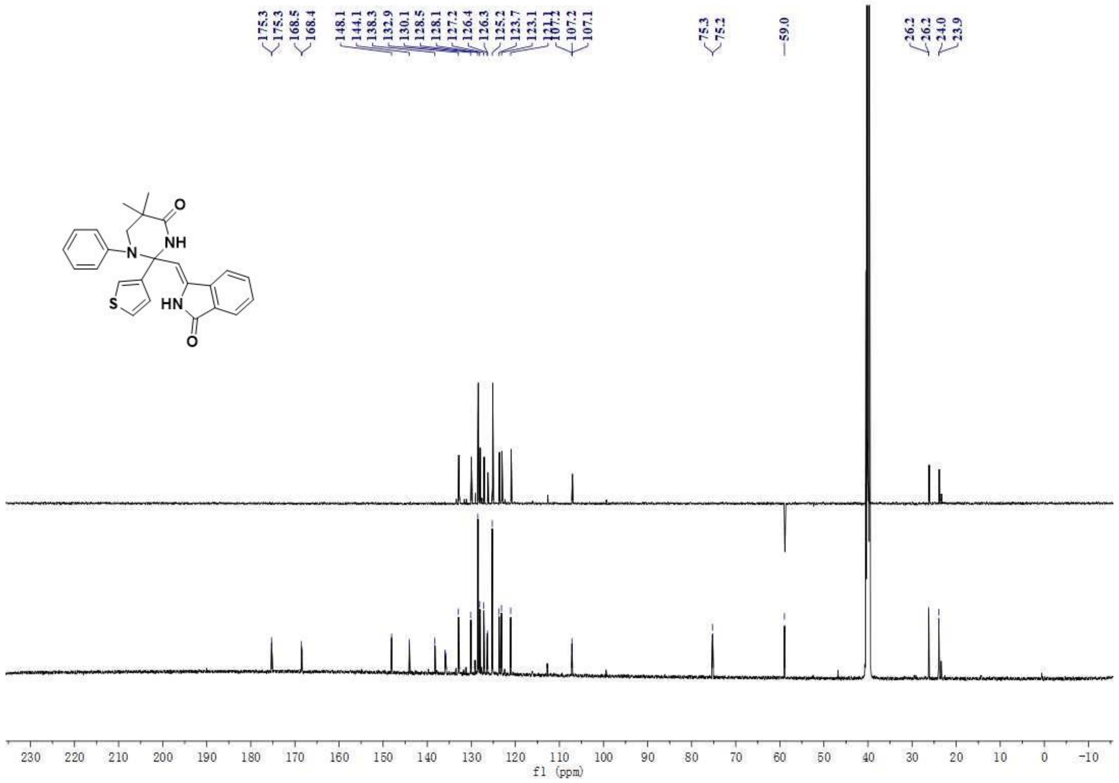

(*Z/E*)-2,2-dimethyl-9-((3-oxoisoindolin-1-ylidene)methyl)-9-phenyl-2,3-dihydro-1*H*,9*H*-

pyrazolo[1,2-*a*]indazol-1-one (4aa)

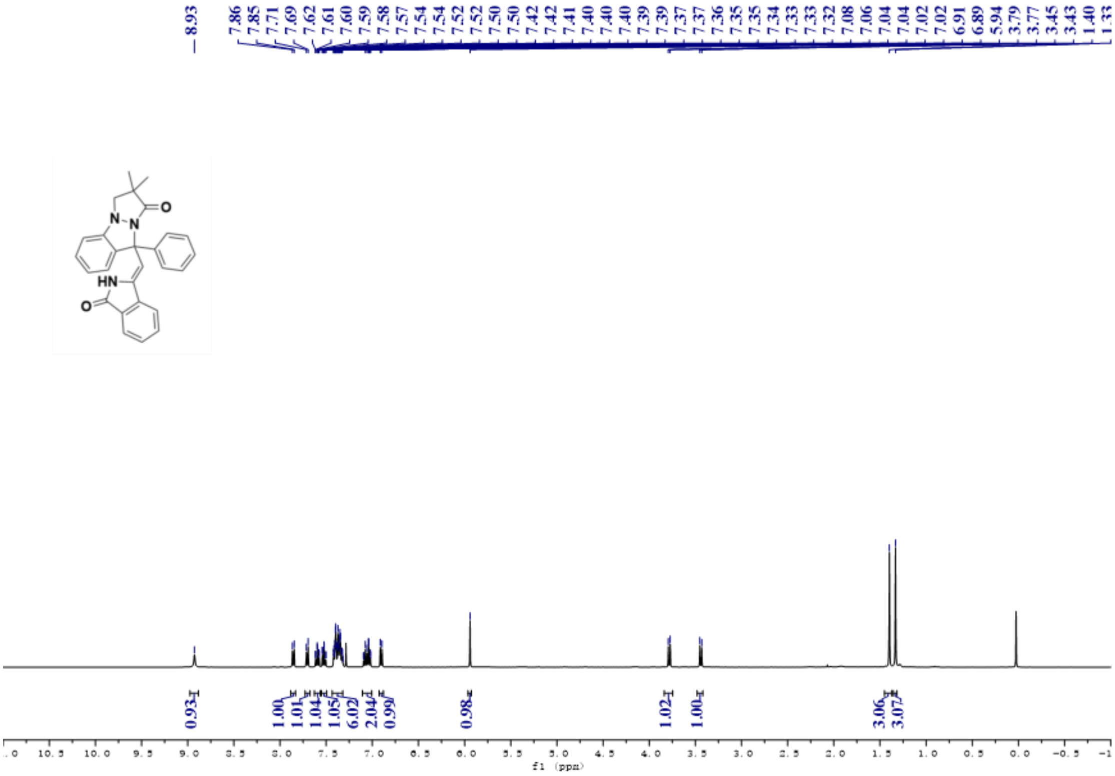

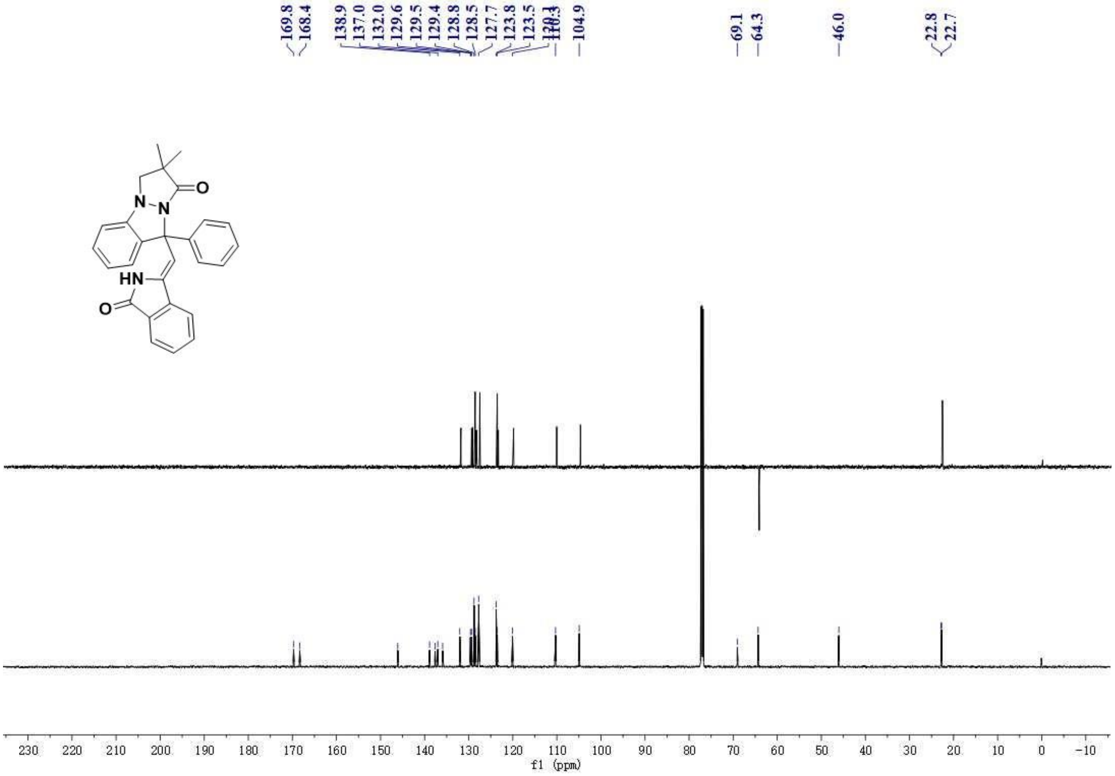

(*Z/E*)-7-fluoro-2,2-dimethyl-9-((3-oxoisoindolin-1-ylidene)methyl)-9-phenyl-2,3-dihydro-

1*H*,9*H*-pyrazolo[1,2-*a*]indazol-1-one (4ba)

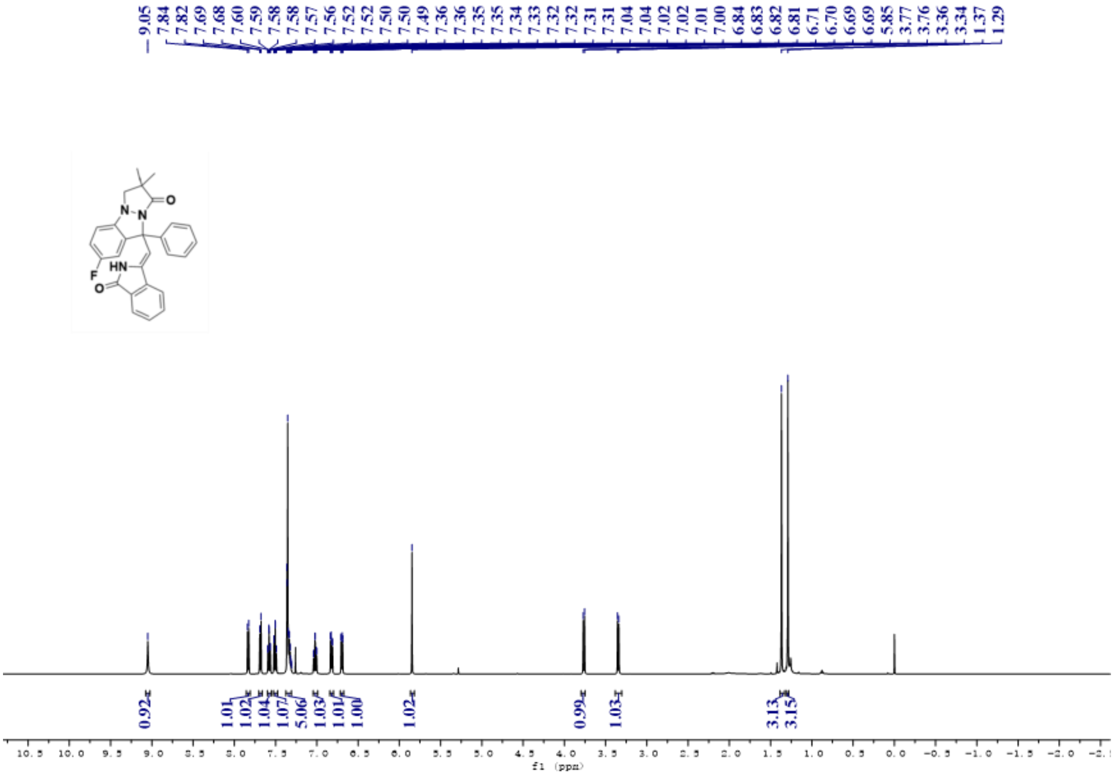

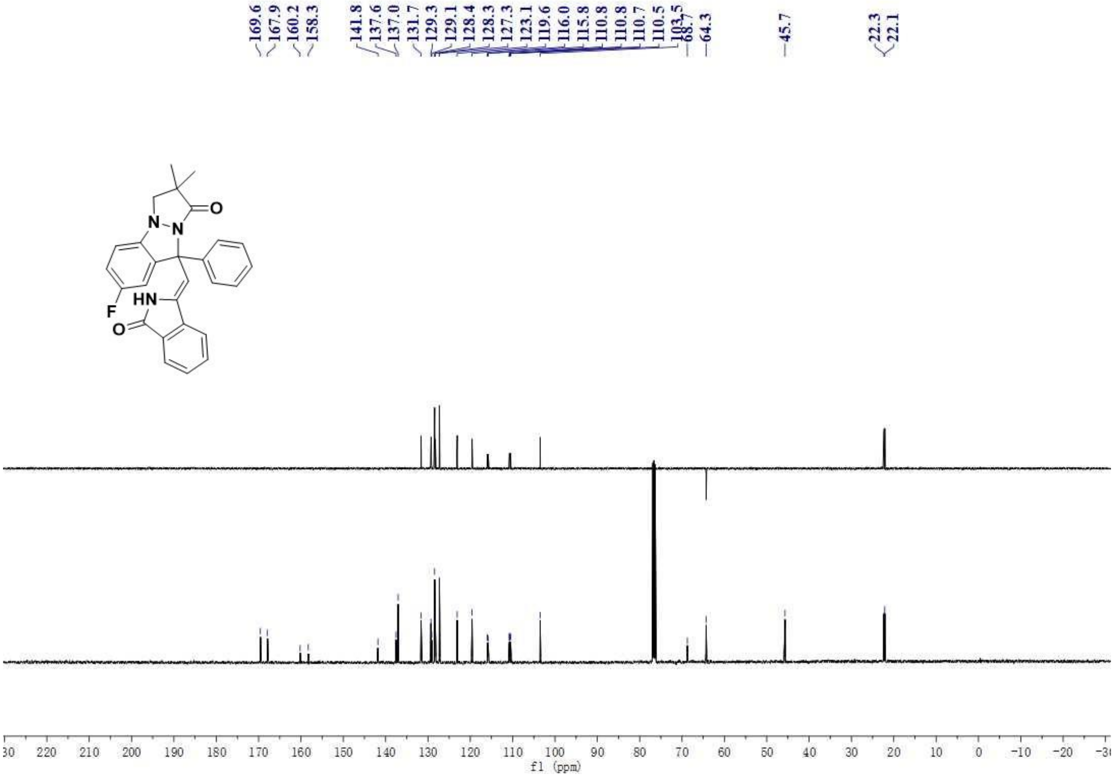

(*Z/E*)-7-chloro-2,2-dimethyl-9-((3-oxoisoindolin-1-ylidene)methyl)-9-phenyl-2,3-

dihydro-1*H*,9*H*-pyrazolo[1,2-*a*]indazol-1-one (4ca)

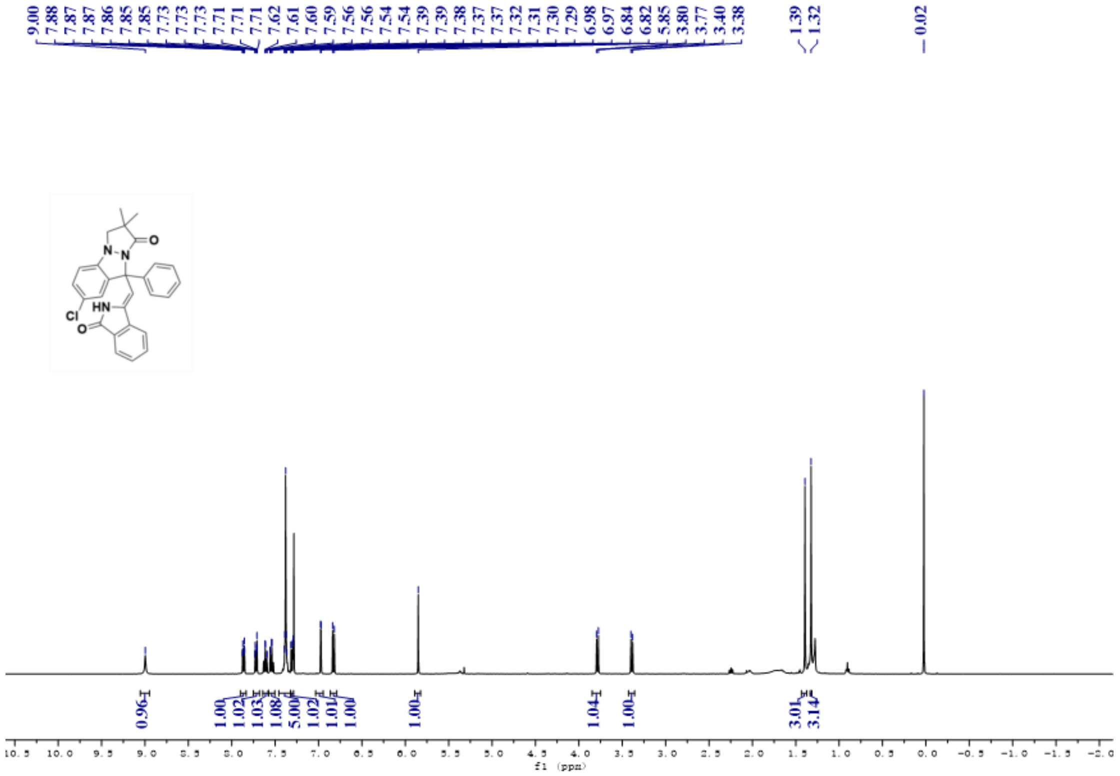

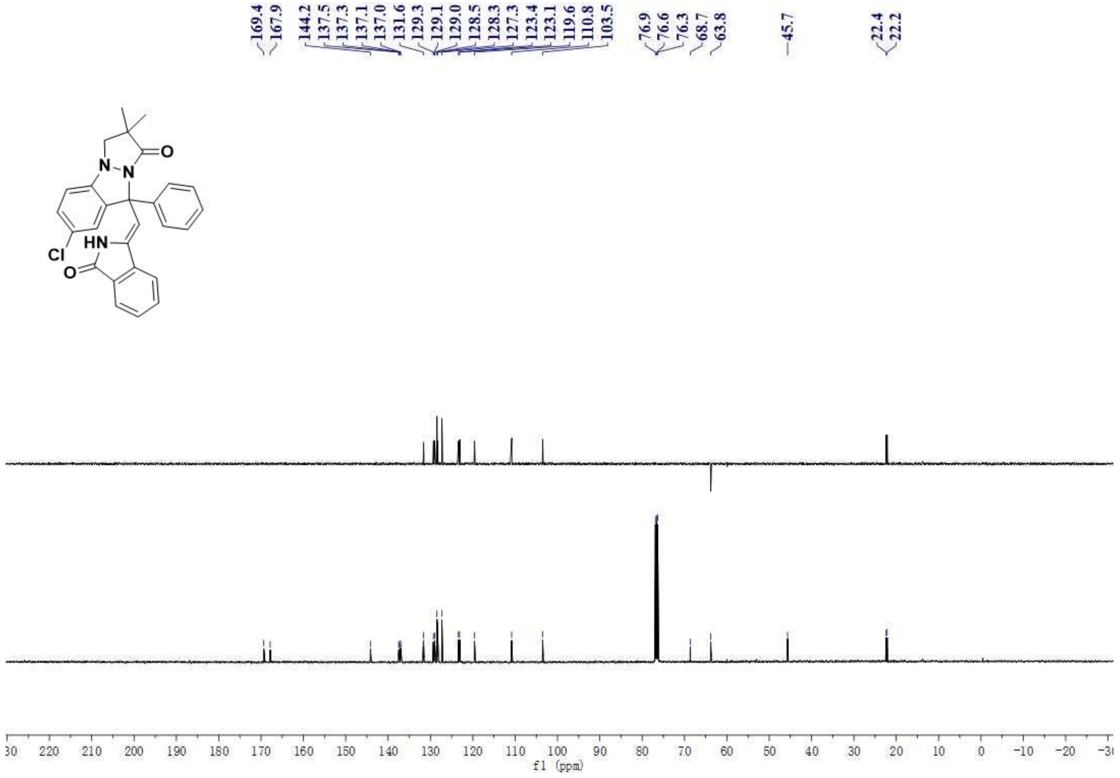

(*Z/E*)-7-bromo-2,2-dimethyl-9-((3-oxoisoindolin-1-ylidene)methyl)-9-phenyl-2,3-dihydro-1*H*,9*H*-pyrazolo[1,2-*a*]indazol-1-one (4da)

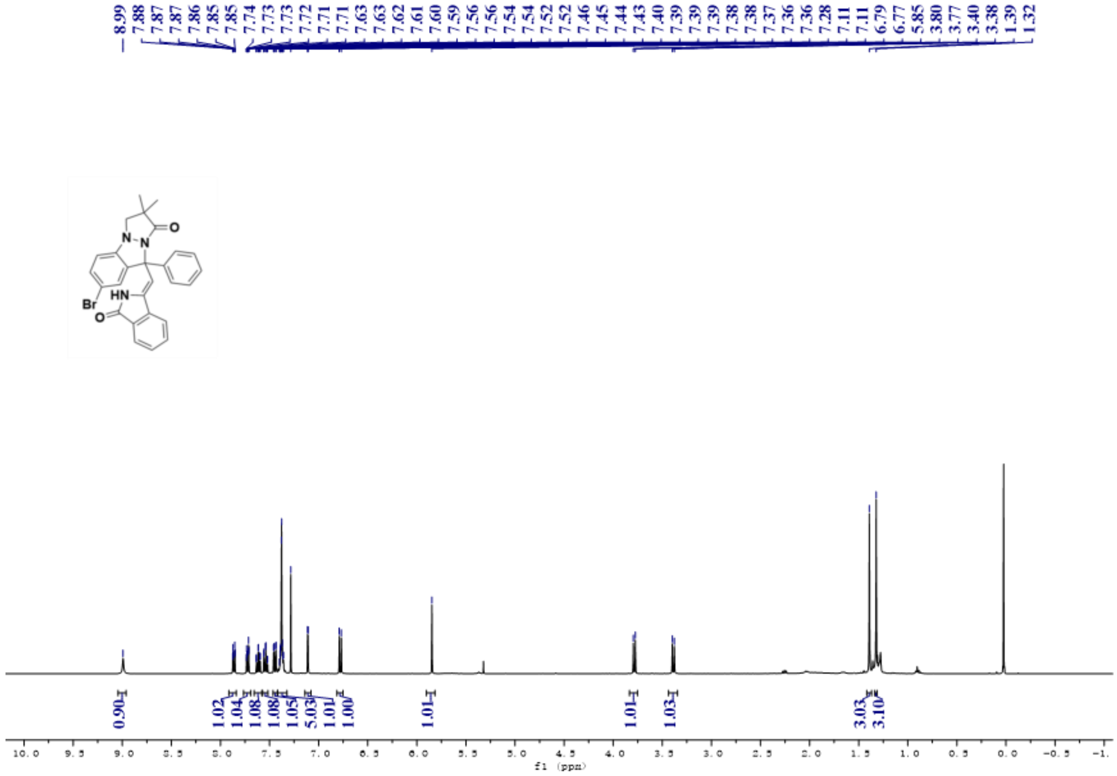

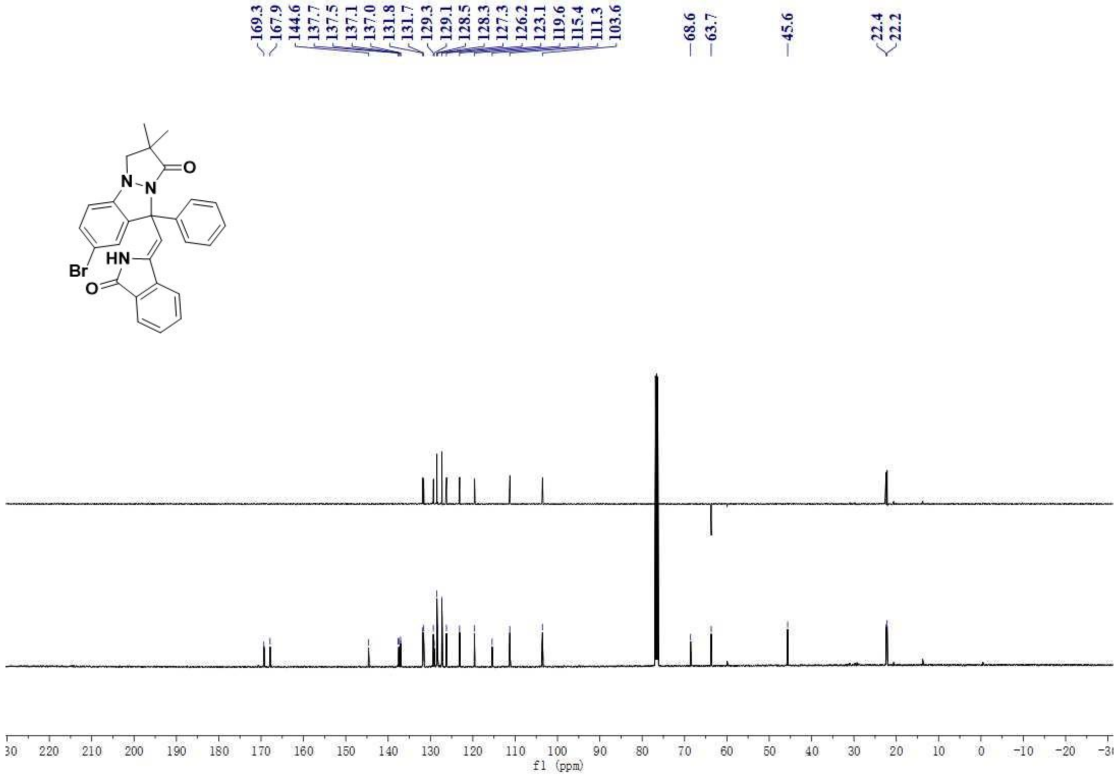

(*Z/E*)-2,2,7-trimethyl-9-((3-oxoisoindolin-1-ylidene)methyl)-9-phenyl-2,3-dihydro-1*H*,9*H*-pyrazolo[1,2-*a*]indazol-1-one (4ea)

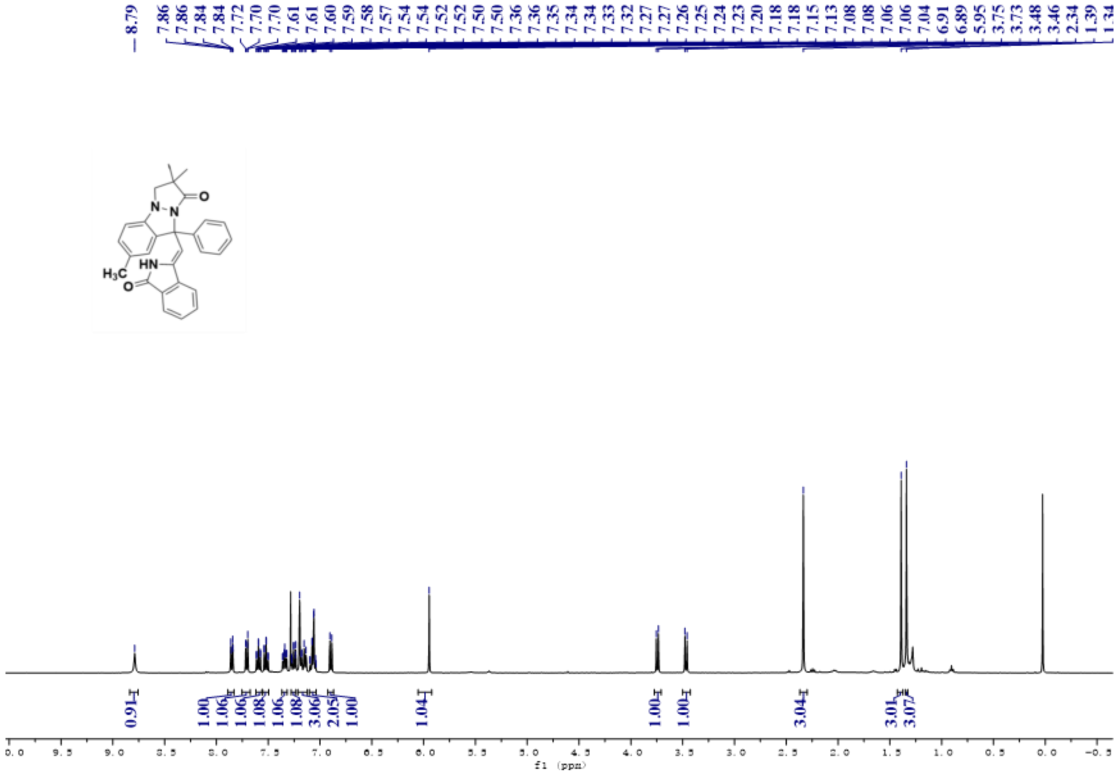

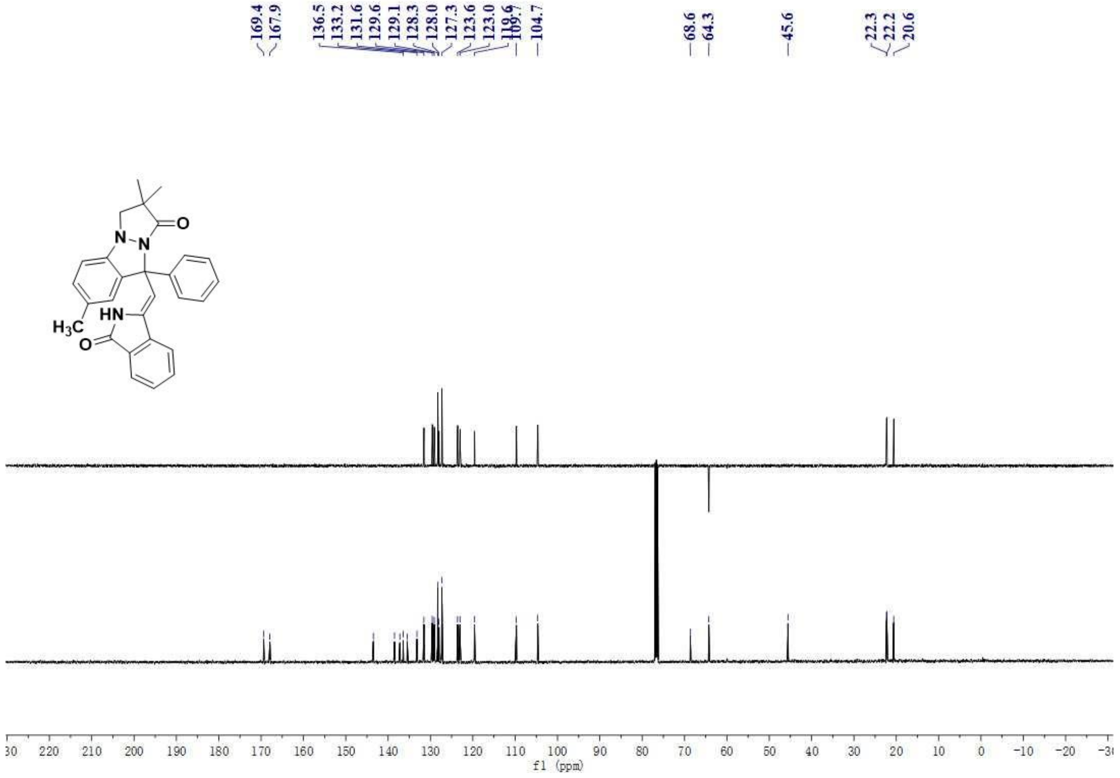

(*Z/E*)-7-methoxy-2,2-dimethyl-9-((3-oxoisoindolin-1-ylidene)methyl)-9-phenyl-2,3-dihydro-1*H*,9*H*-pyrazolo[1,2-*a*]indazol-1-one (4fa)

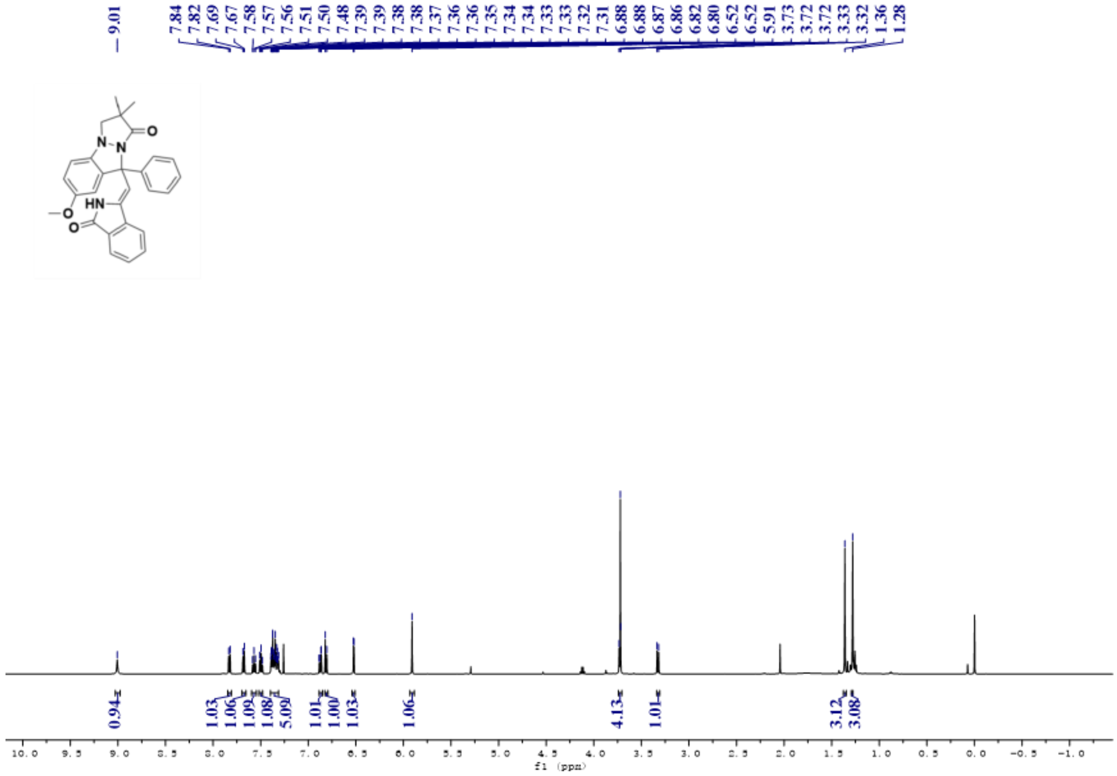

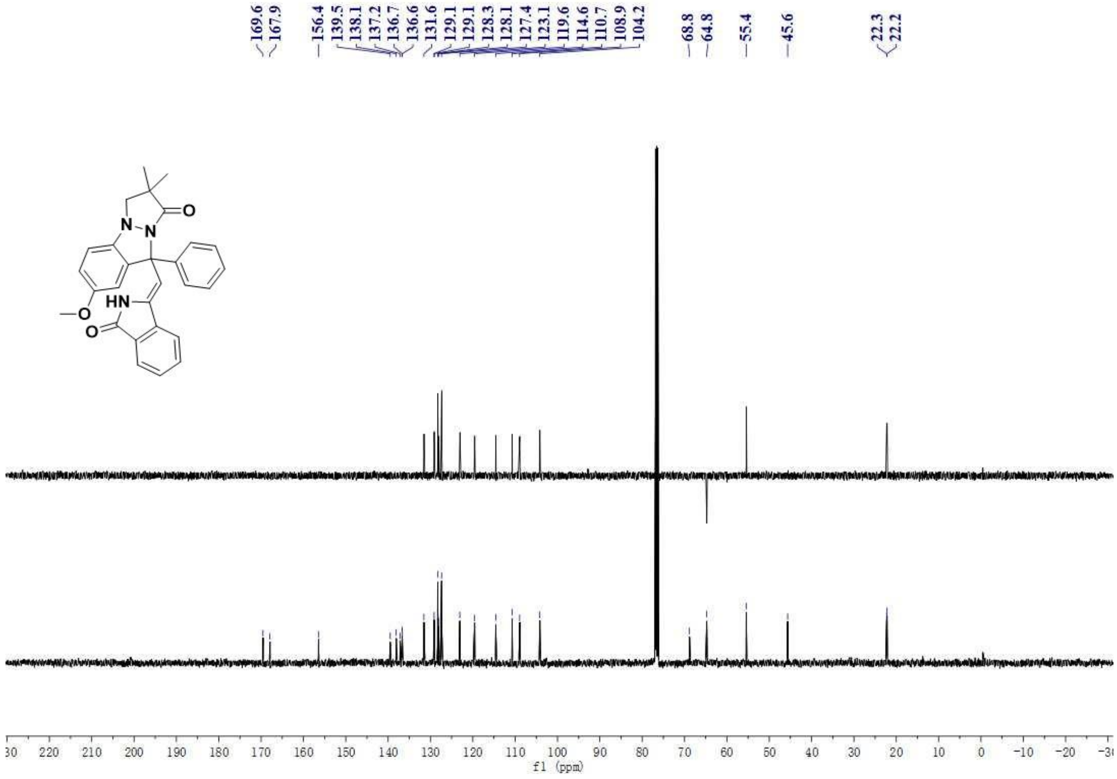

(*Z/E*)-2,2-dimethyl-9-((3-oxoisoindolin-1-ylidene)methyl)-9-phenyl-7-(trifluoromethyl)-2,3-dihydro-1*H*,9*H*-pyrazolo[1,2-*a*]indazol-1-one (4ga)

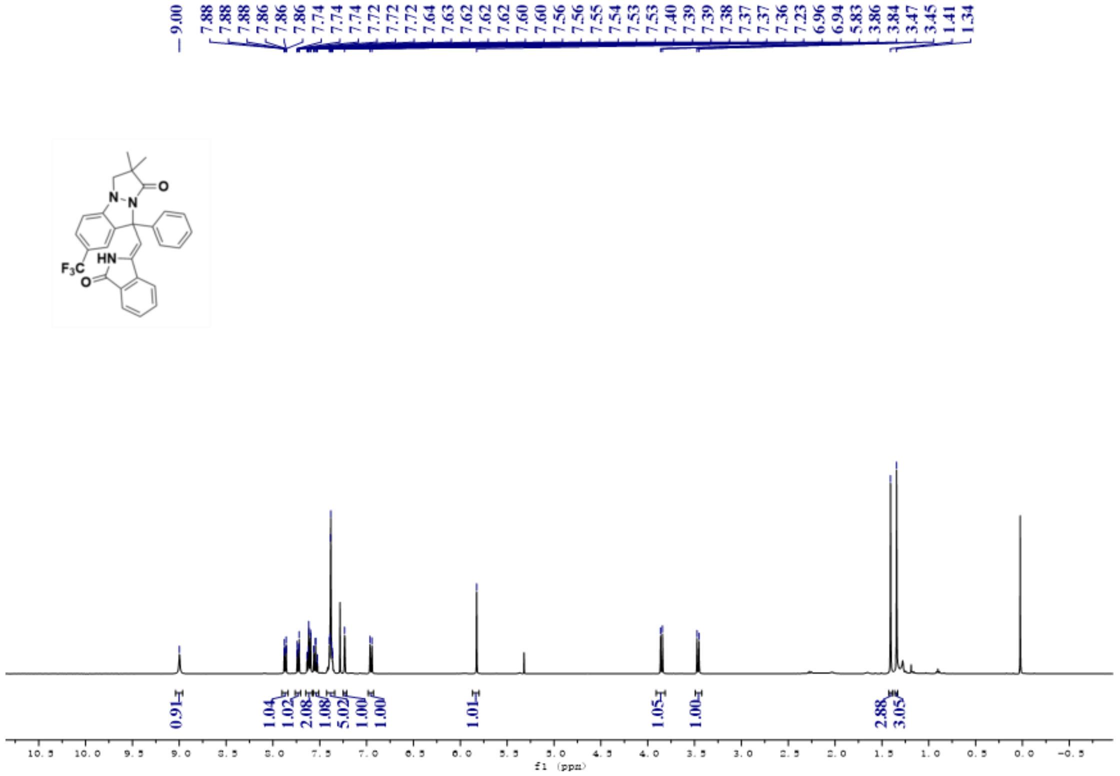

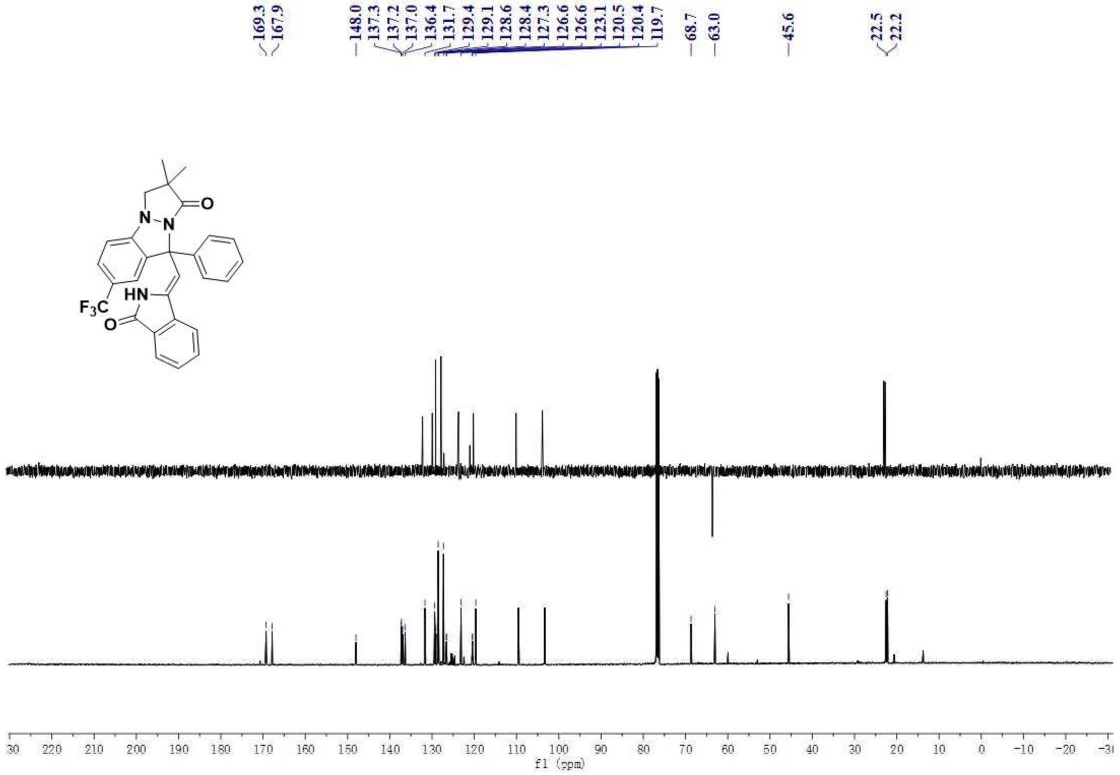

(*Z/E*)-2,2-dimethyl-1-oxo-9-((3-oxoisoindolin-1-ylidene)methyl)-9-phenyl-2,3-dihydro-1*H*,9*H*-pyrazolo[1,2-*a*]indazole-7-carbonitrile (4ha)

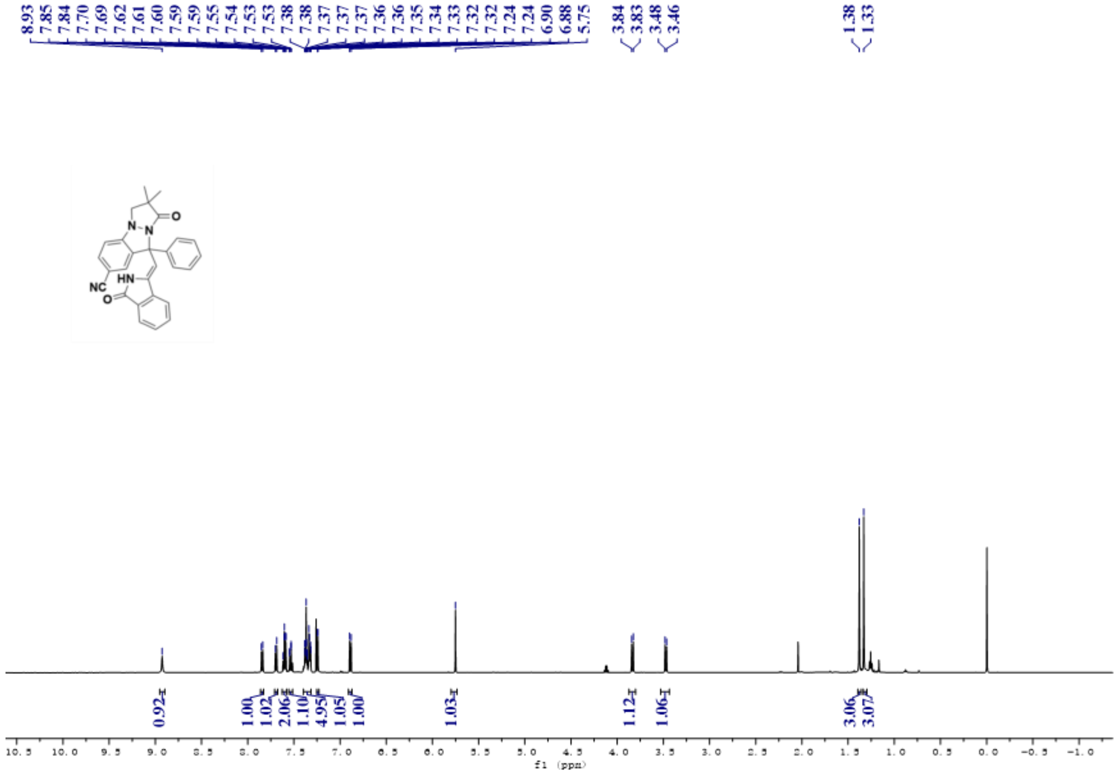

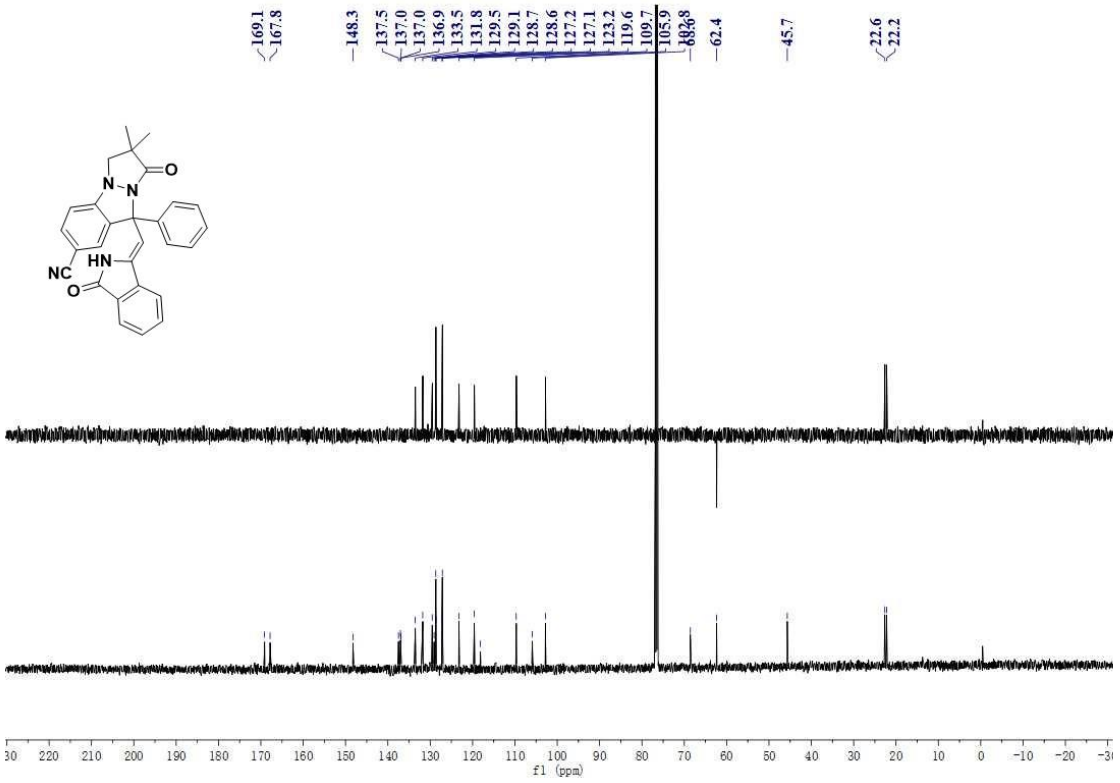

(*Z/E*)-6-fluoro-2,2-dimethyl-9-((3-oxoisoindolin-1-ylidene)methyl)-9-phenyl-2,3-dihydro-1*H*,9H-pyrazolo[1,2-a]indazol-1-one (4ia)

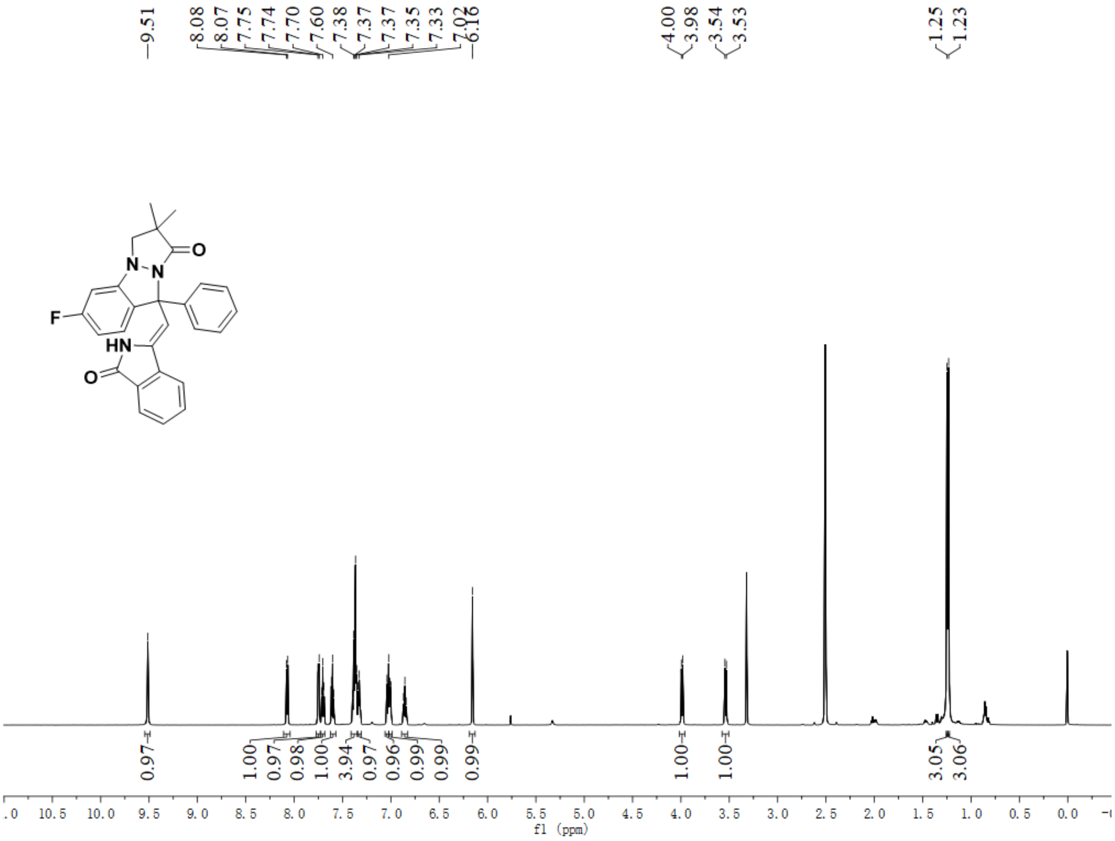

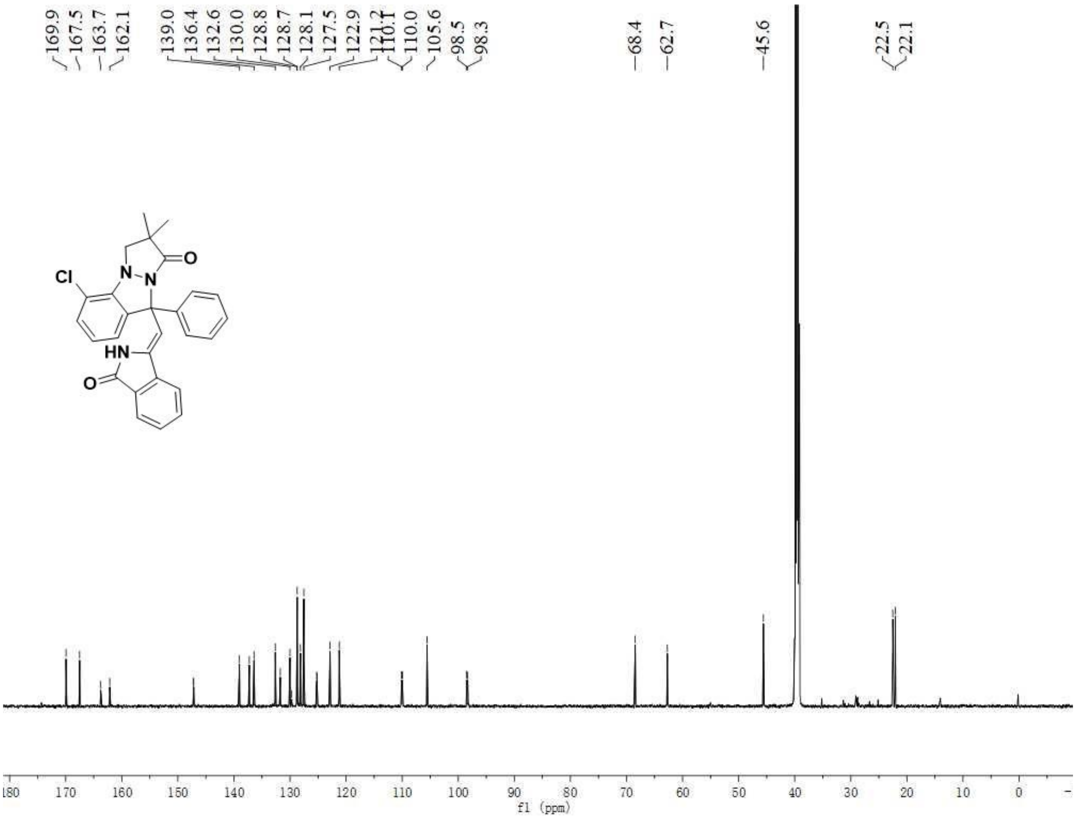

(*Z/E*)-6-chloro-2,2-dimethyl-9-((3-oxoisoindolin-1-ylidene)methyl)-9-phenyl-2,3-dihydro-1*H*,9*H*-pyrazolo[1,2-*a*]indazol-1-one (4ja)

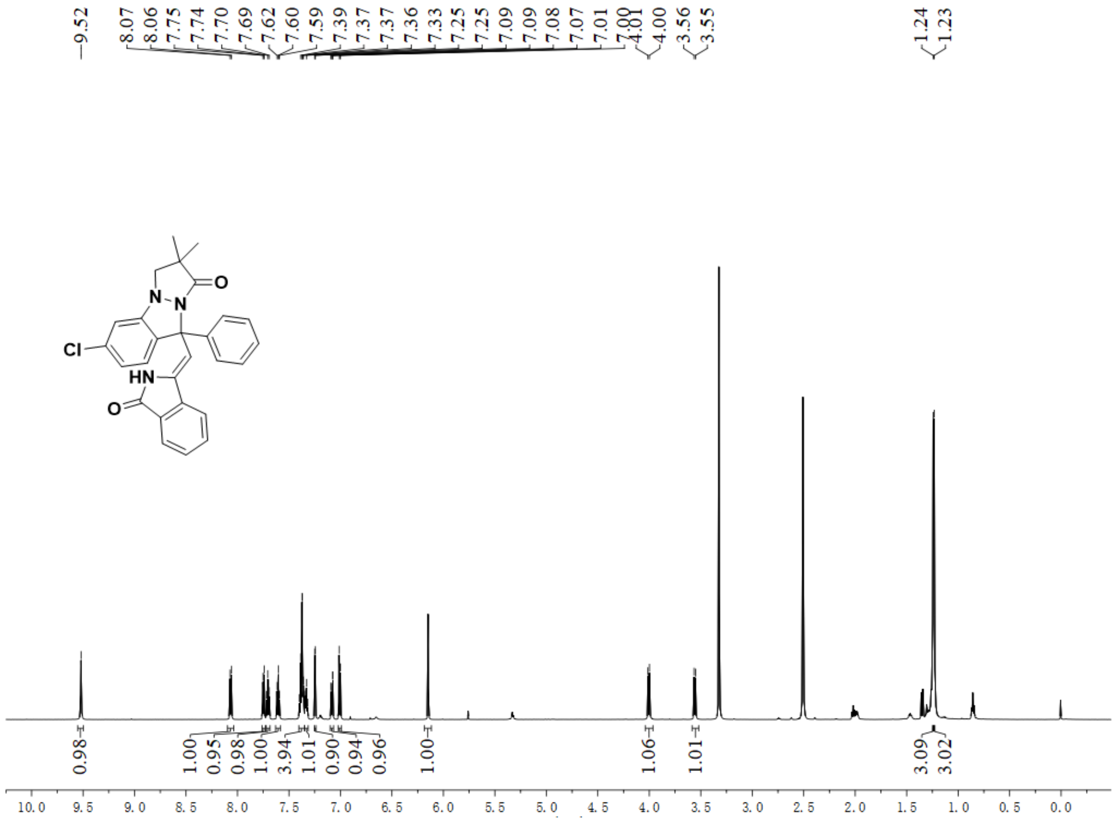

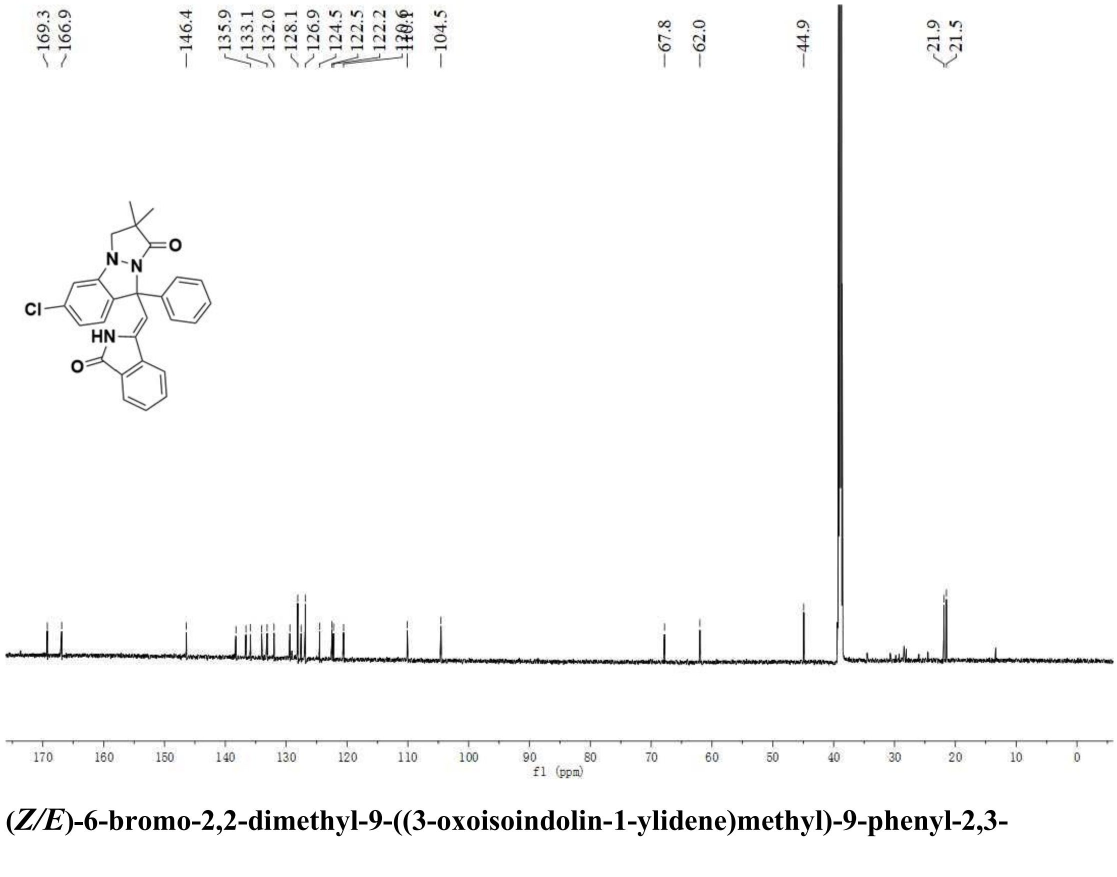

(*Z/E*)-6-bromo-2,2-dimethyl-9-((3-oxoisoindolin-1-ylidene)methyl)-9-phenyl-2,3-

dihydro-1*H*,9*H*-pyrazolo[1,2-*a*]indazol-1-one (4ka)

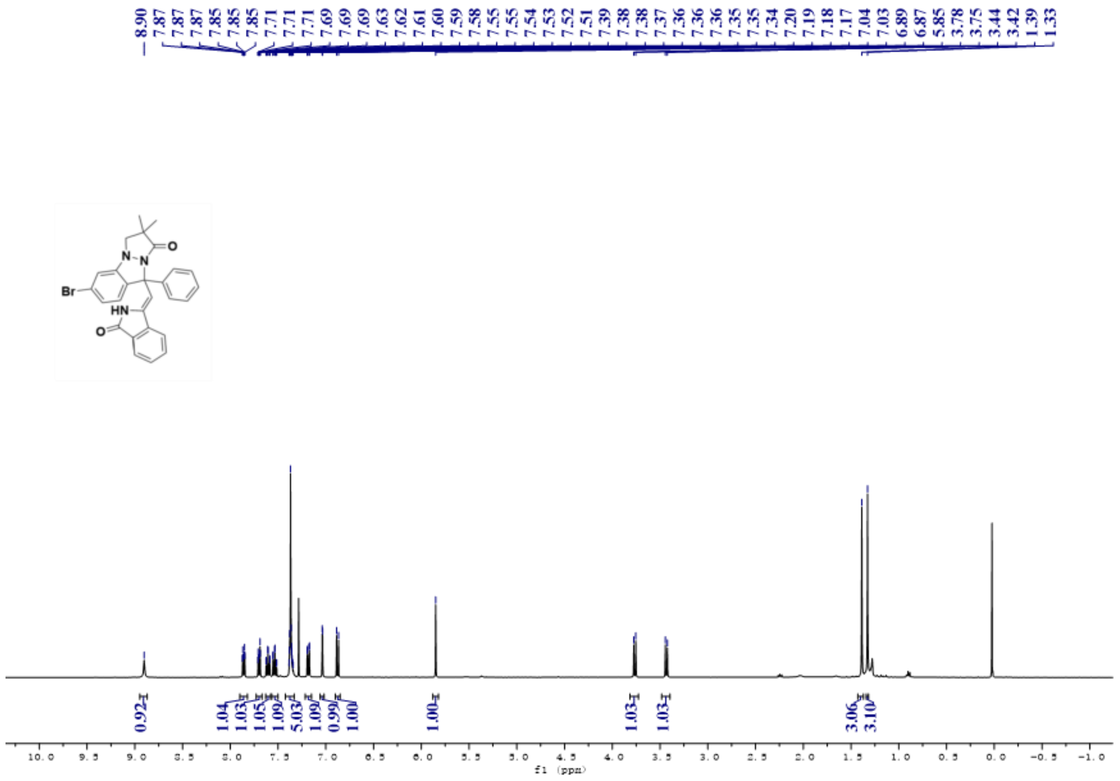

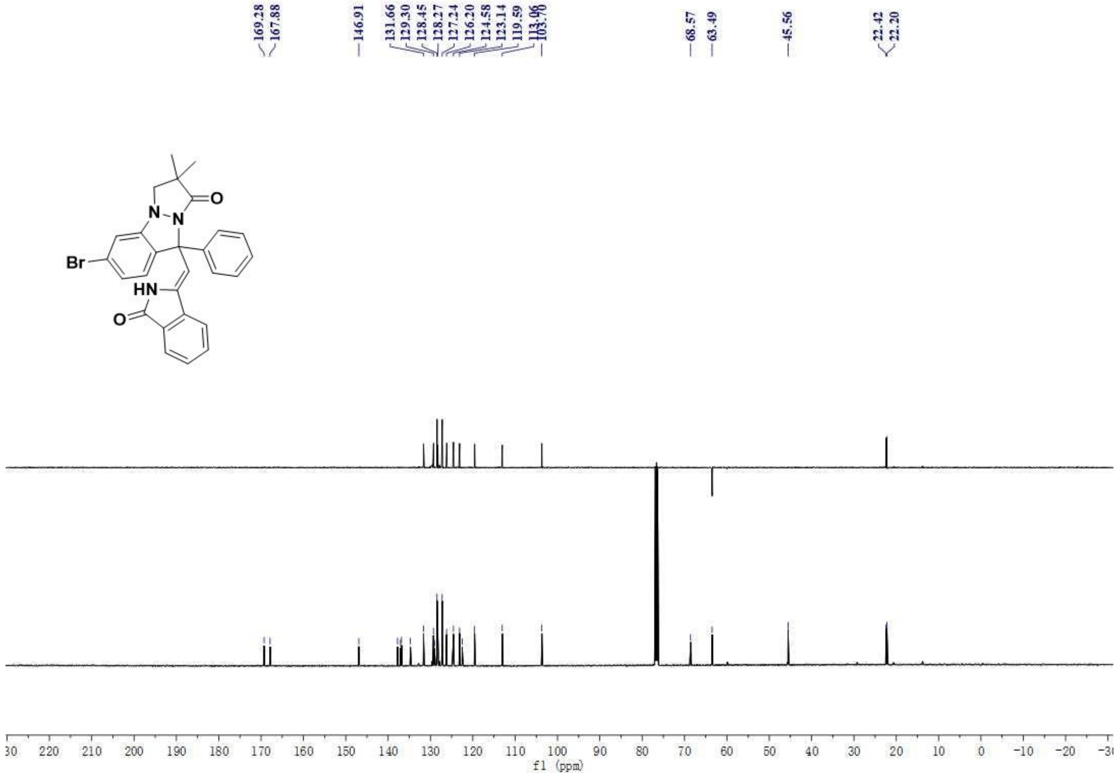

(*Z/E*)-6-bromo-2,2-dimethyl-9-((3-oxoisoindolin-1-ylidene)methyl)-9-phenyl-2,3-dihydro-1*H*,9*H*-pyrazolo[1,2-*a*]indazol-1-one (4ab)

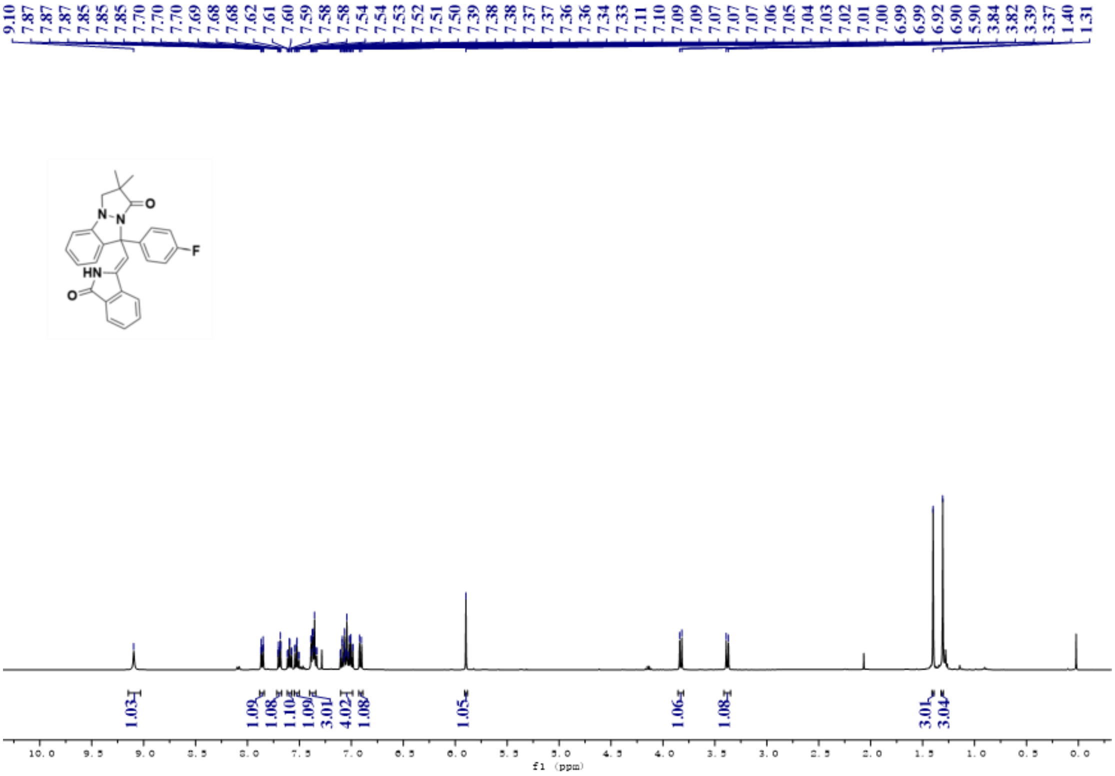

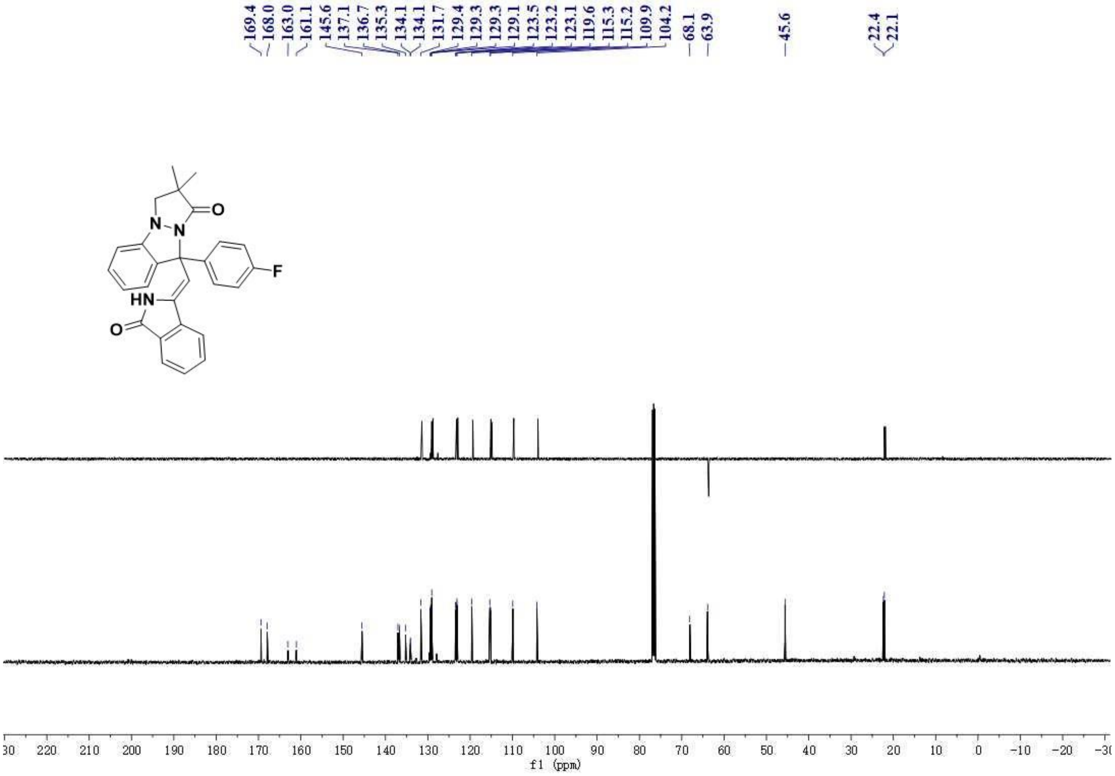

(*Z/E*)-9-(4-chlorophenyl)-2,2-dimethyl-9-((3-oxoisoindolin-1-ylidene)methyl)-2,3-dihydro-1*H*,9*H*-pyrazolo[1,2-*a*]indazol-1-one (4ac)

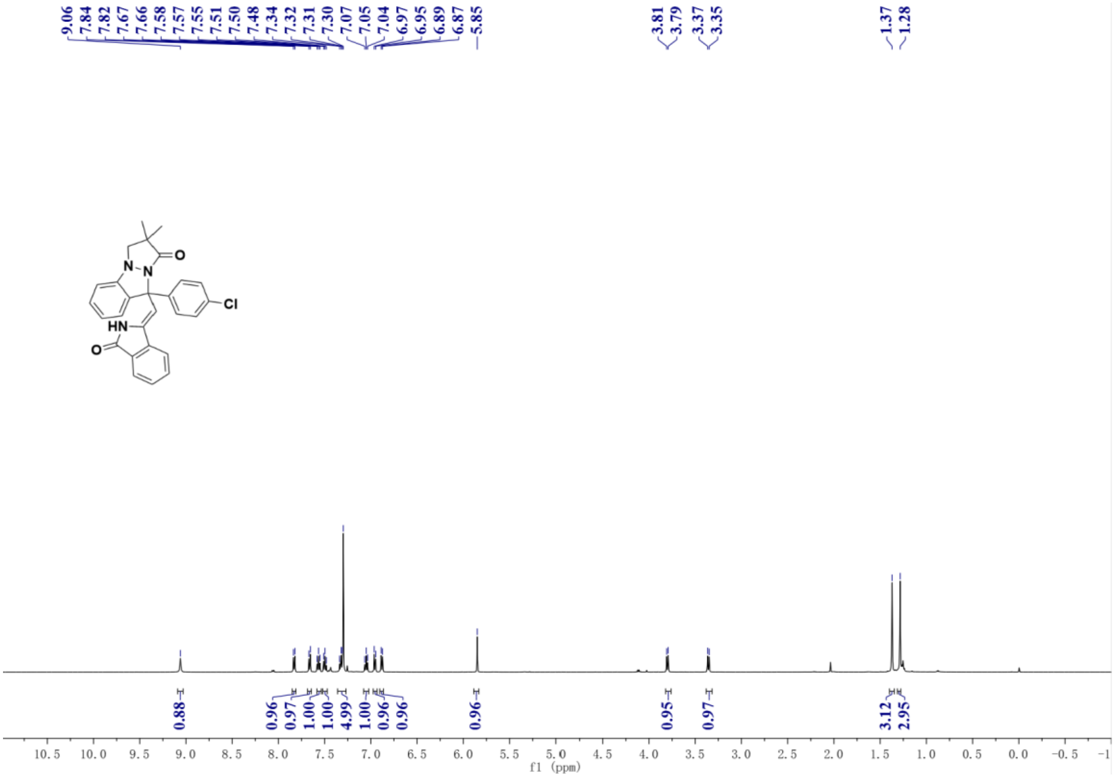

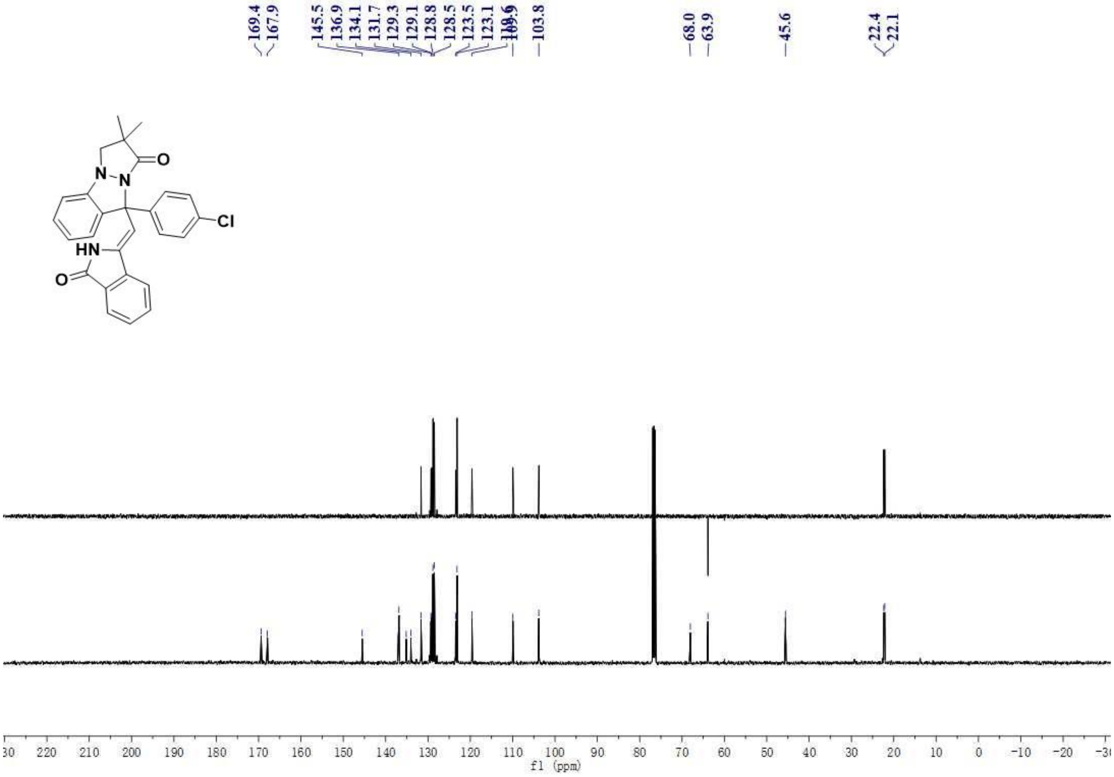

(*Z/E*)-9-(4-bromophenyl)-2,2-dimethyl-9-((3-oxoisoindolin-1-ylidene)methyl)-2,3-dihydro-1*H*,9*H*-pyrazolo[1,2-*a*]indazol-1-one (4ad)

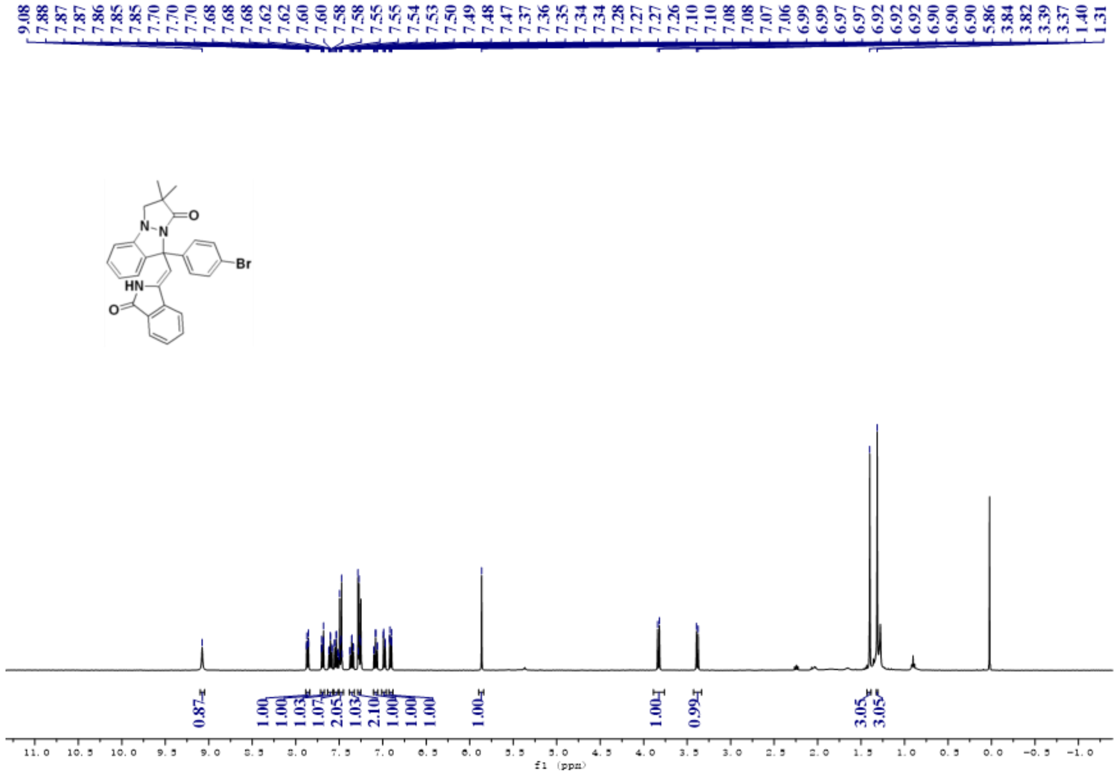

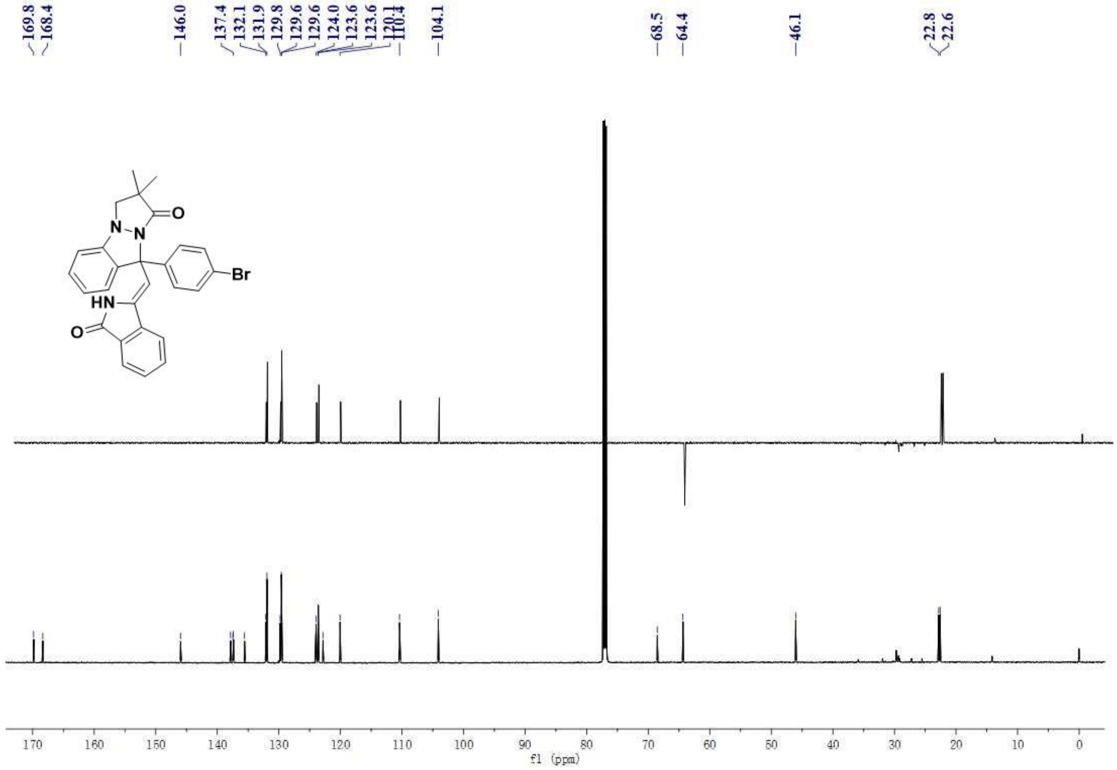

(*Z/E*)-2,2-dimethyl-9-((3-oxoisoindolin-1-ylidene)methyl)-9-(p-tolyl)-2,3-dihydro-1*H*,9*H*-pyrazolo[1,2-*a*]indazol-1-one (4ae)

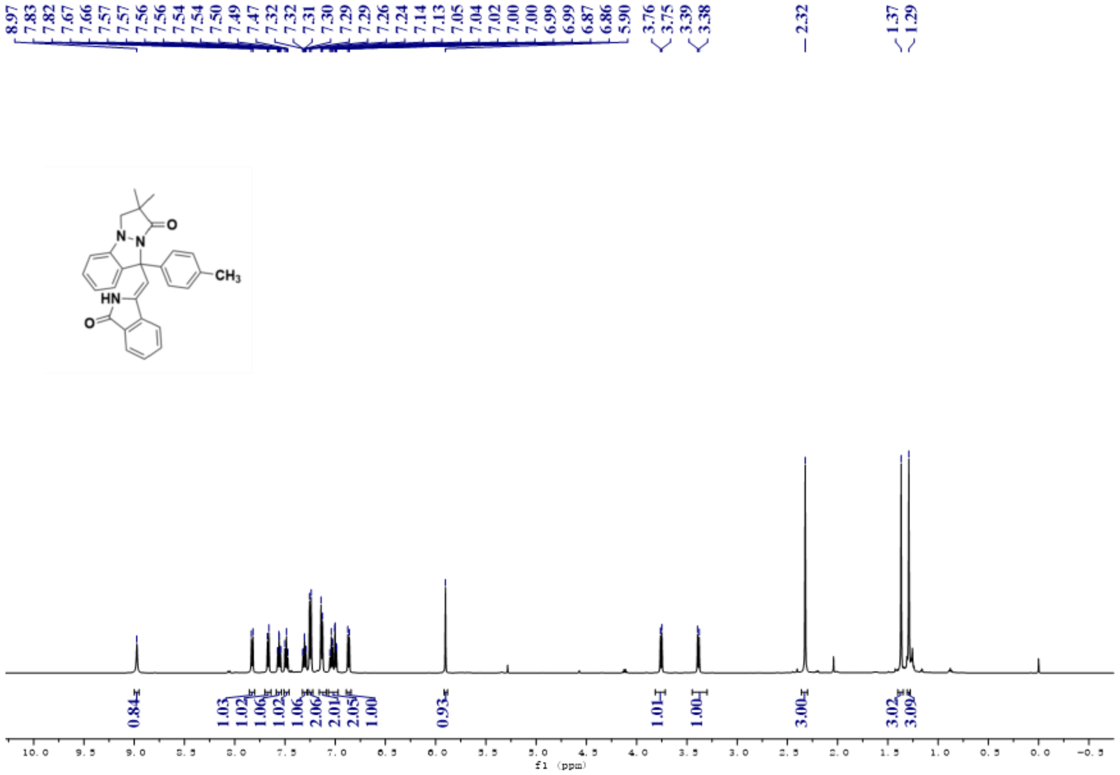

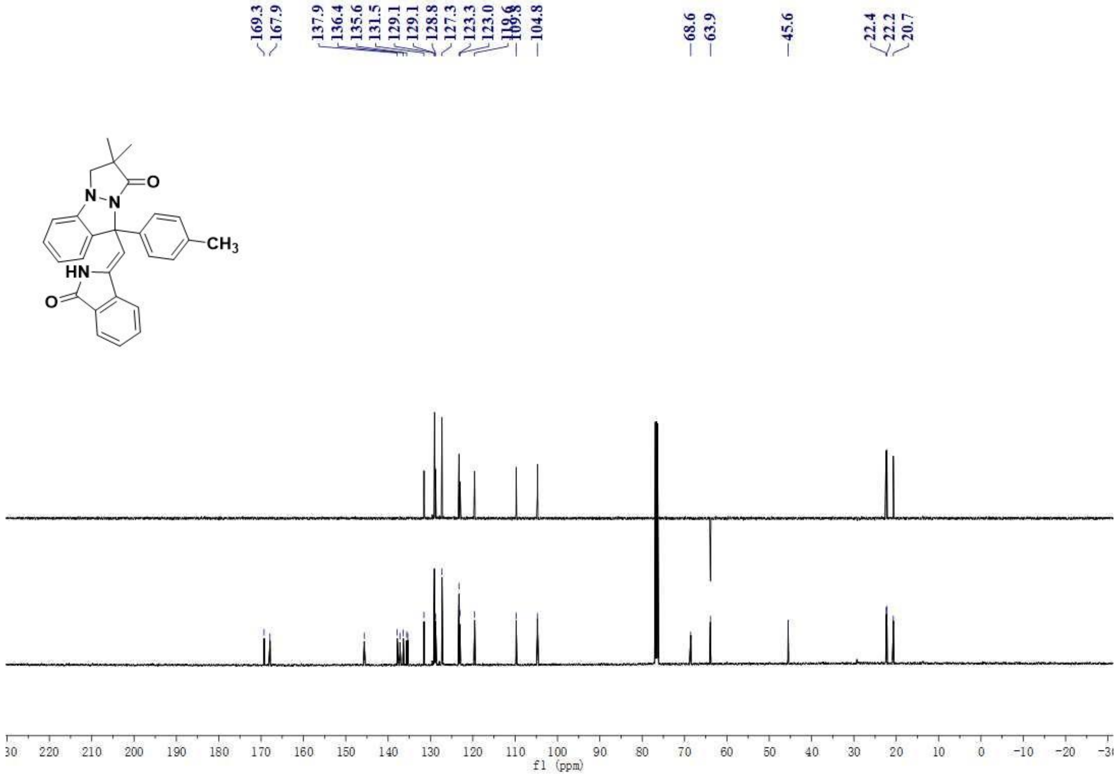

(*Z/E*)-2,2-dimethyl-9-((3-oxoisoindolin-1-ylidene)methyl)-9-(4-pentylphenyl)-2,3-dihydro-1*H*,9*H*-pyrazolo[1,2-*a*]indazol-1-one (4ag)

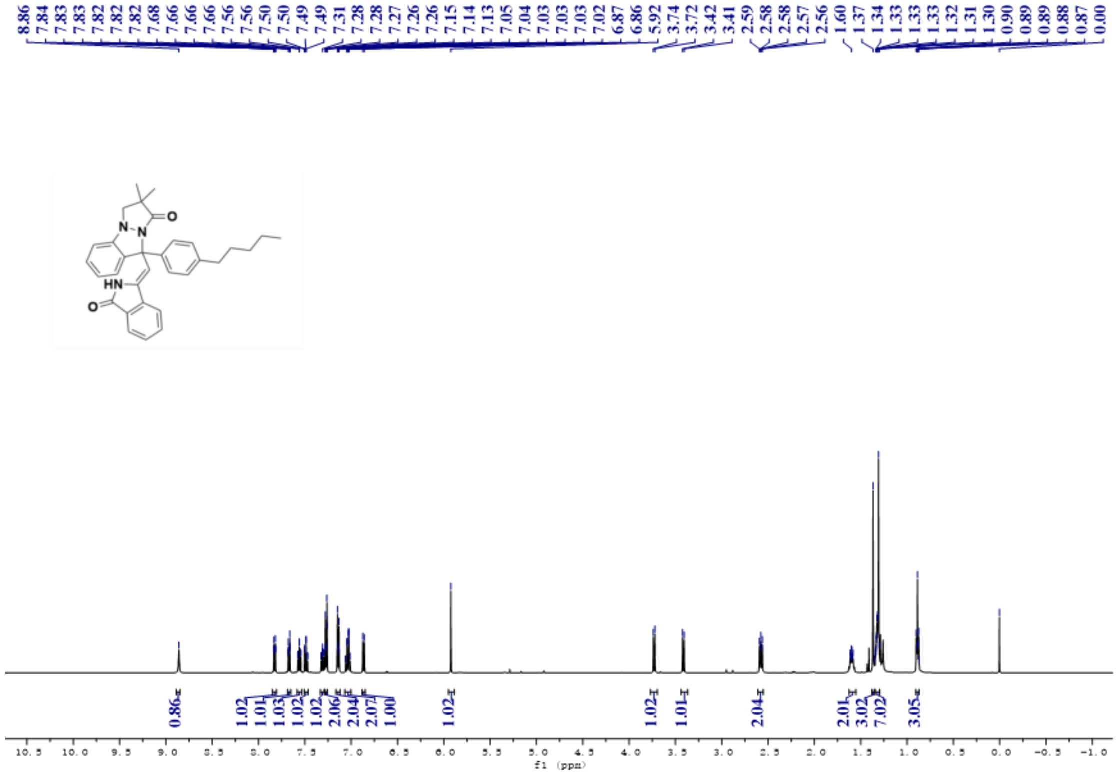

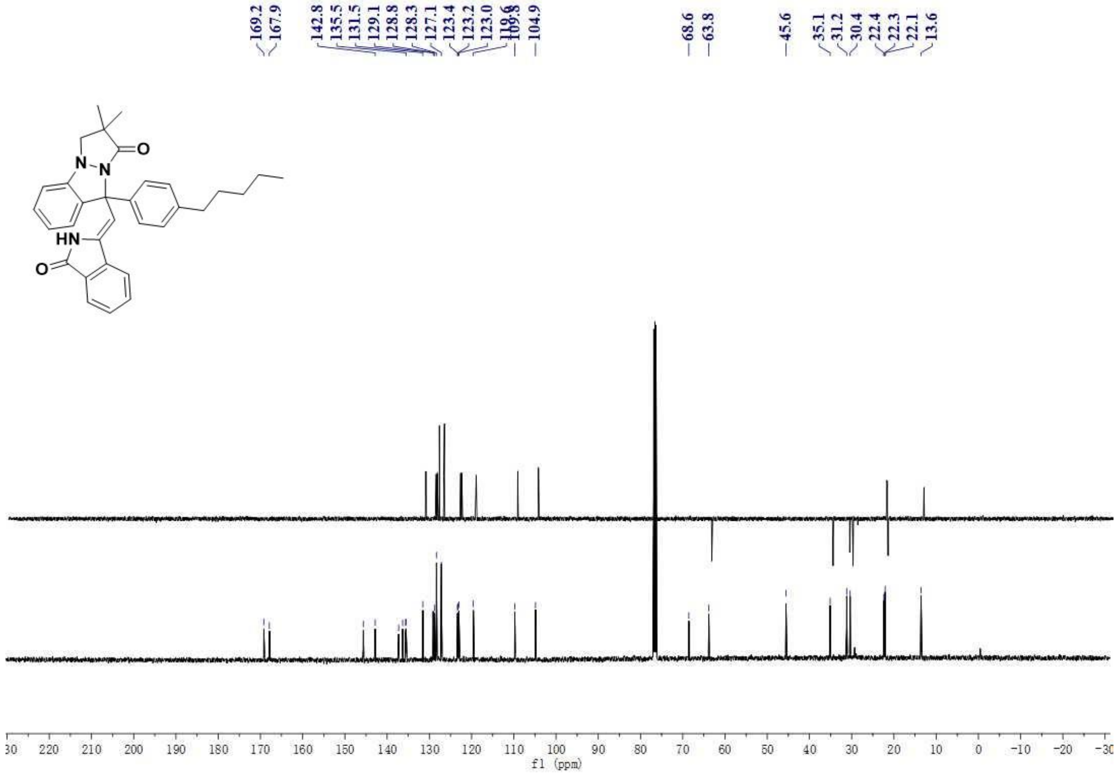

(*Z/E*)-2,2-dimethyl-9-((3-oxoisoindolin-1-ylidene)methyl)-9-(m-tolyl)-2,3-dihydro-1*H*,9*H*-pyrazolo[1,2-*a*]indazol-1-one (4ai)

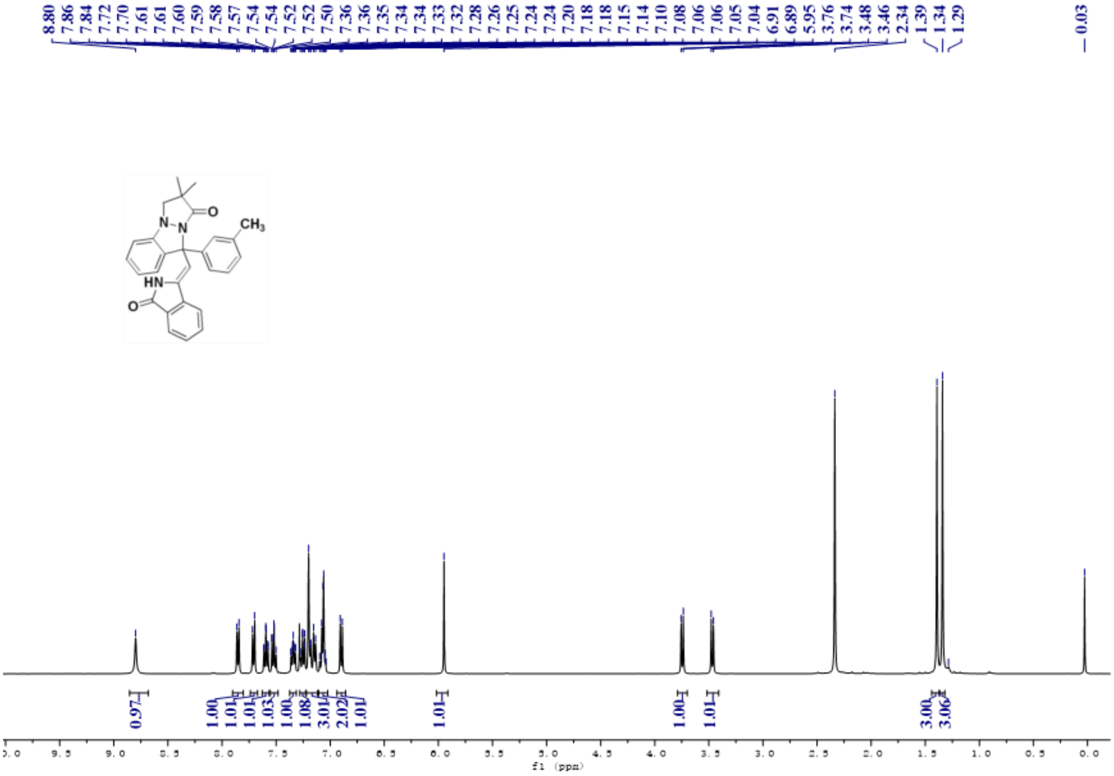

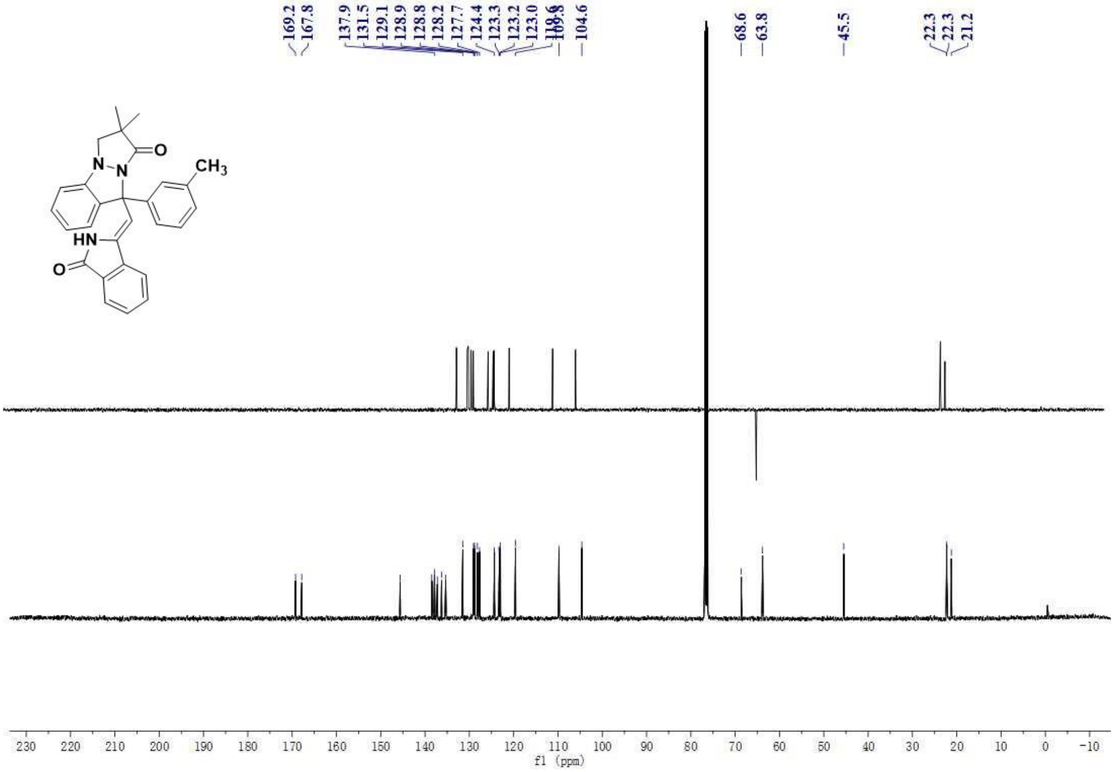

(*Z/E*)-9-(2-chlorophenyl)-2,2-dimethyl-9-((3-oxoisoindolin-1-ylidene)methyl)-2,3-dihydro-1*H*,9*H*-pyrazolo[1,2-*a*]indazol-1-one (4aj)

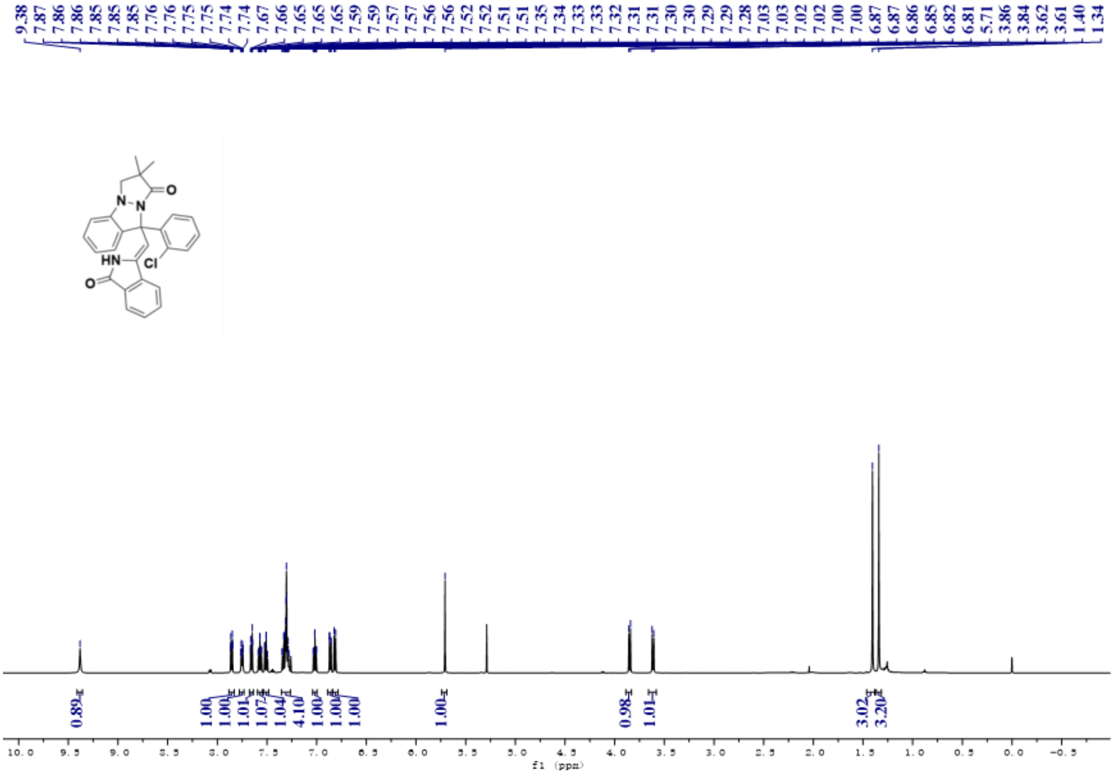

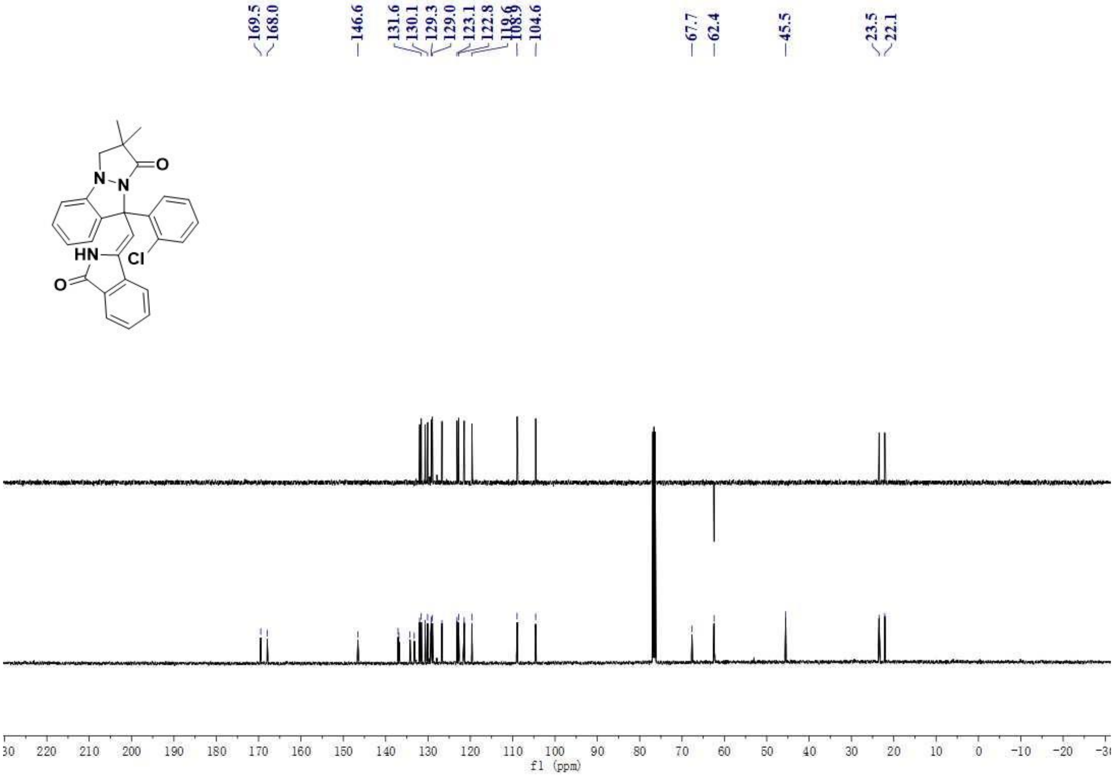

(*Z/E*)-9-(3-chlorophenyl)-2,2-dimethyl-9-((3-oxoisoindolin-1-ylidene)methyl)-2,3-dihydro-1*H*,9*H*-pyrazolo[1,2-*a*]indazol-1-one (4ak)

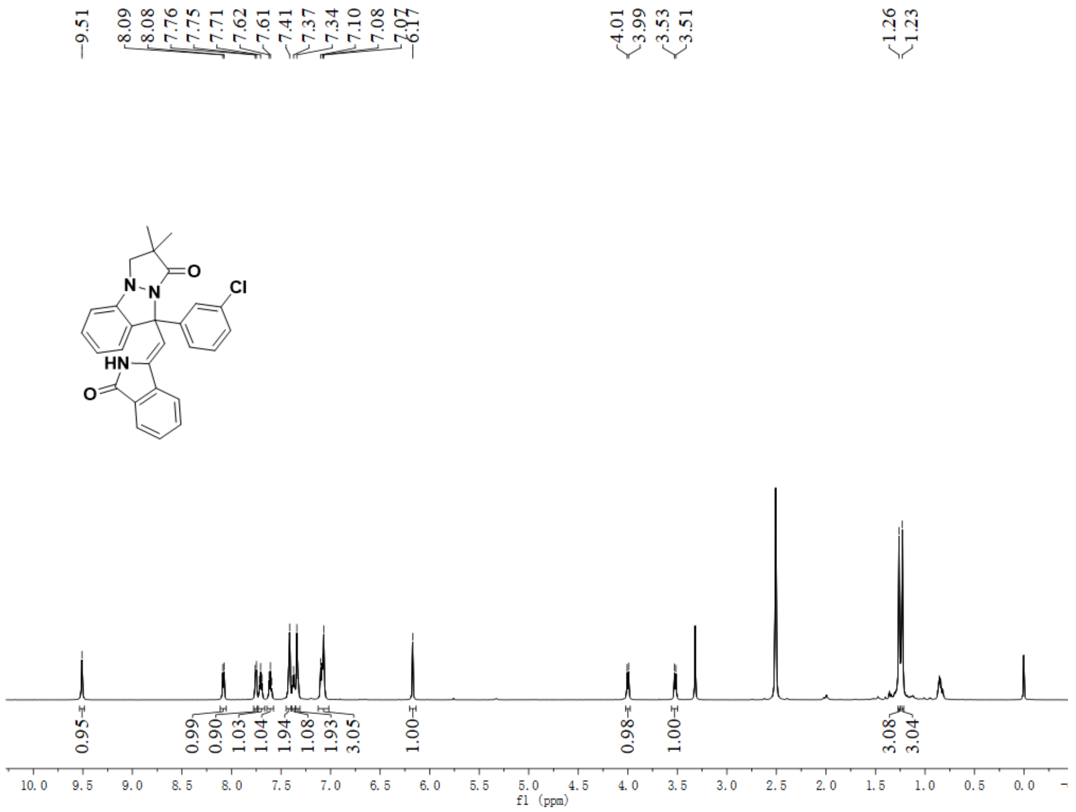

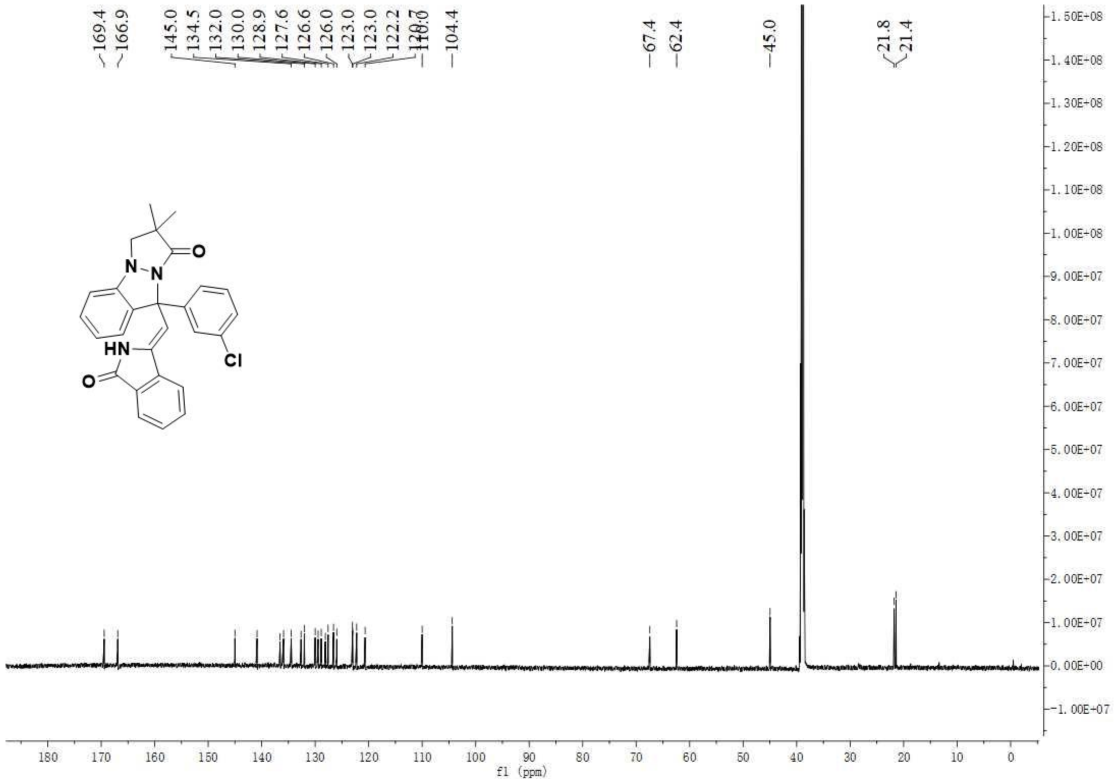

(*Z/E*)-2,2-dimethyl-9-((3-oxoisoindolin-1-ylidene)methyl)-9-(thiophen-3-yl)-2,3-dihydro-1*H*,9*H*-pyrazolo[1,2-*a*]indazol-1-one (4al)

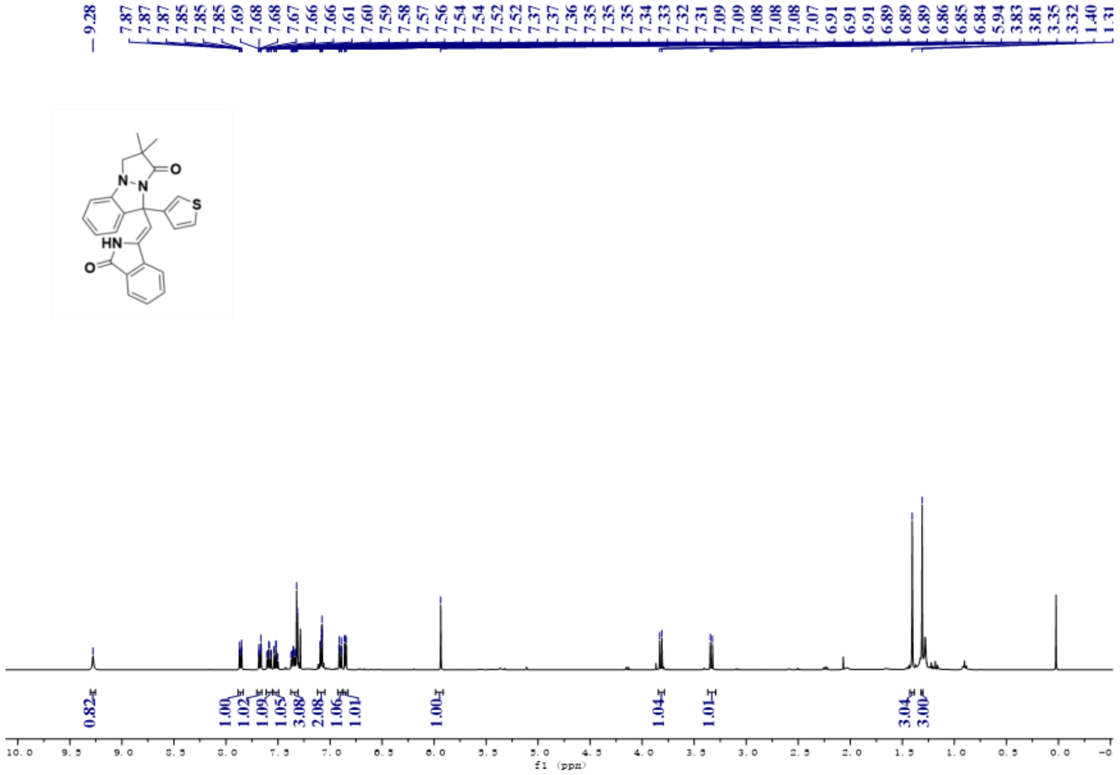

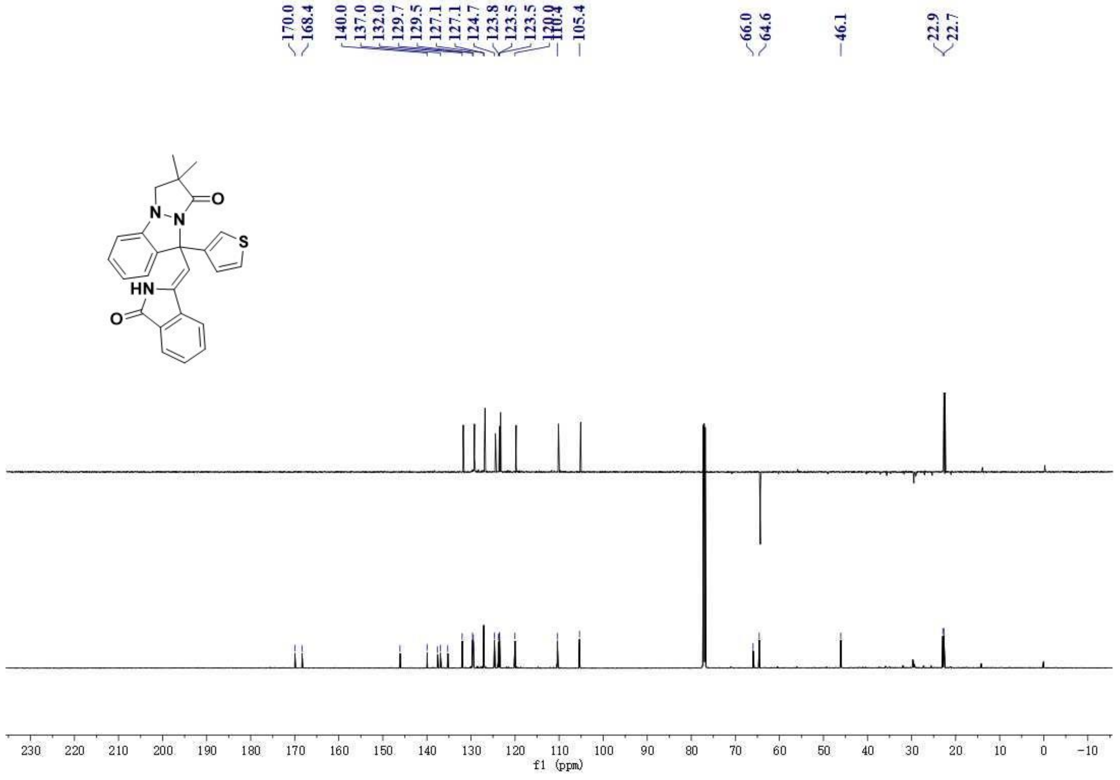

2-(1-(3-amino-2,2-dimethyl-3-oxopropyl)-2-phenyl-1*H*-indole-3-carbonyl)benzamide

(5aa)

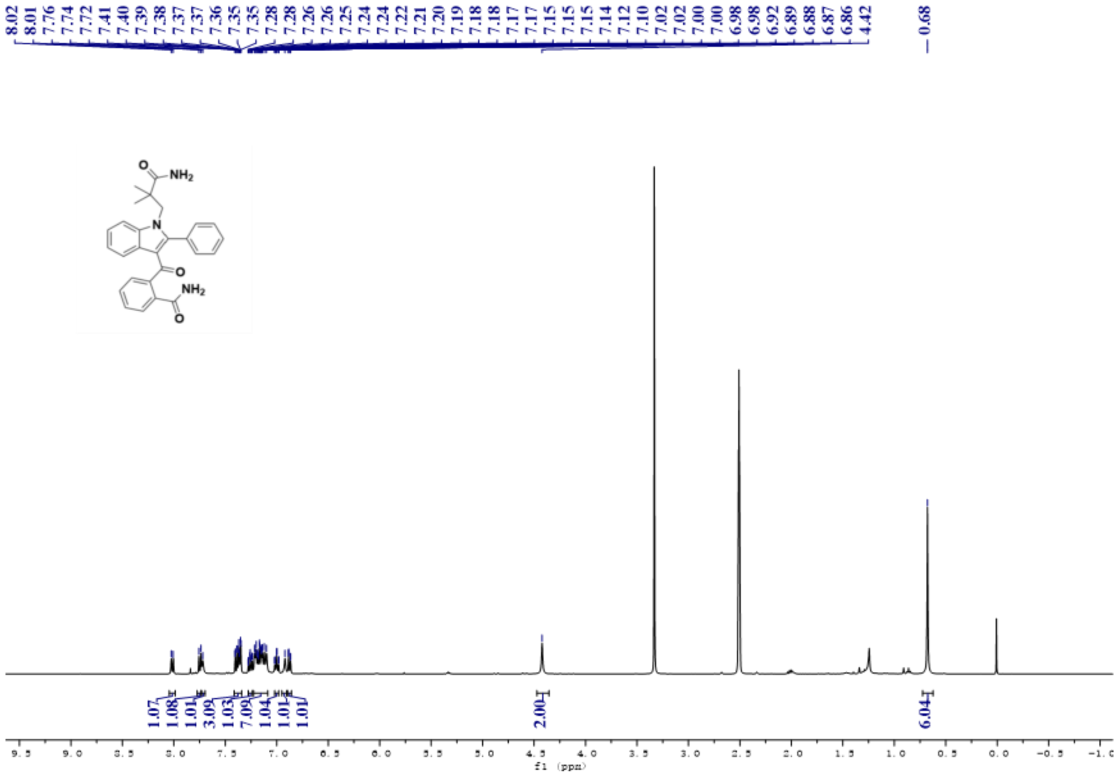

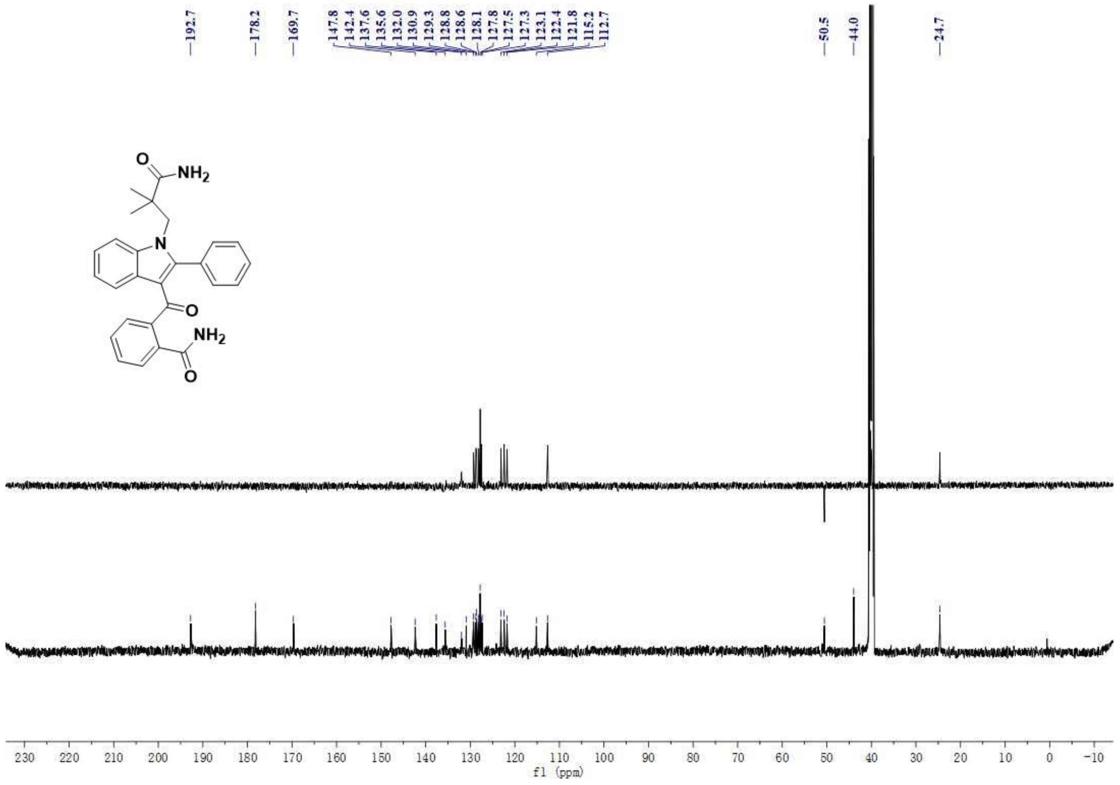

2-(1-(3-amino-2,2-dimethyl-3-oxopropyl)-5-fluoro-2-phenyl-1*H*-indole-3-

carbonyl)benzamide (5ba)

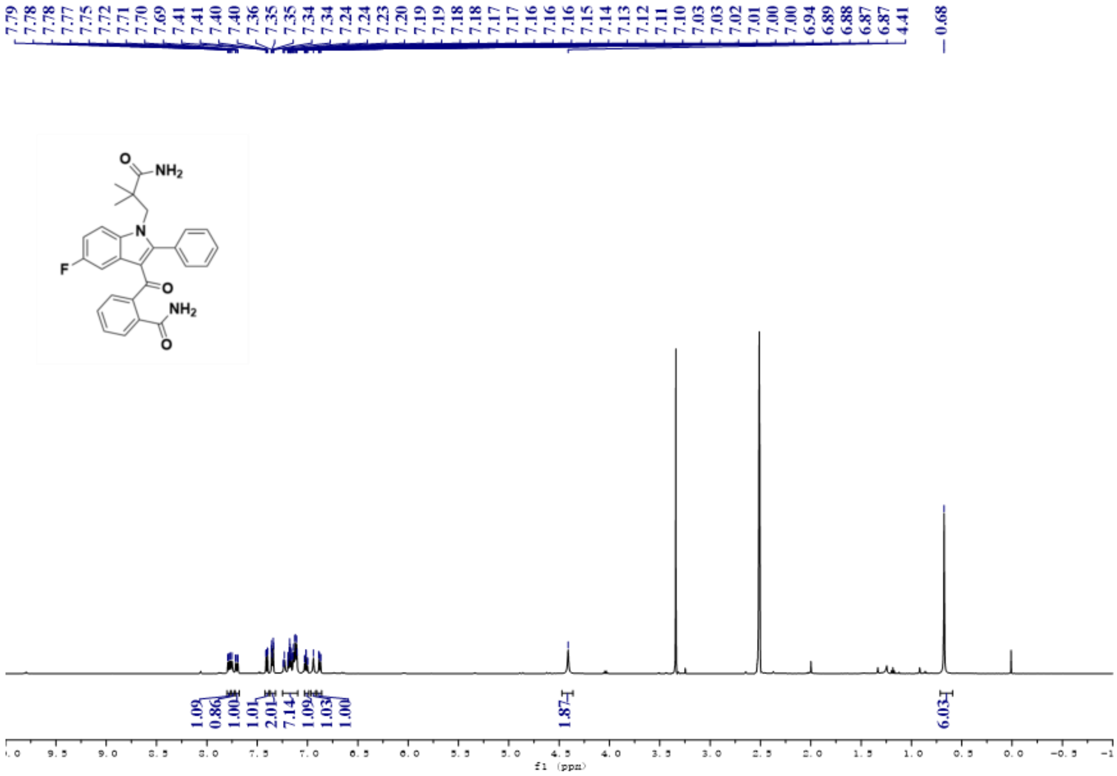

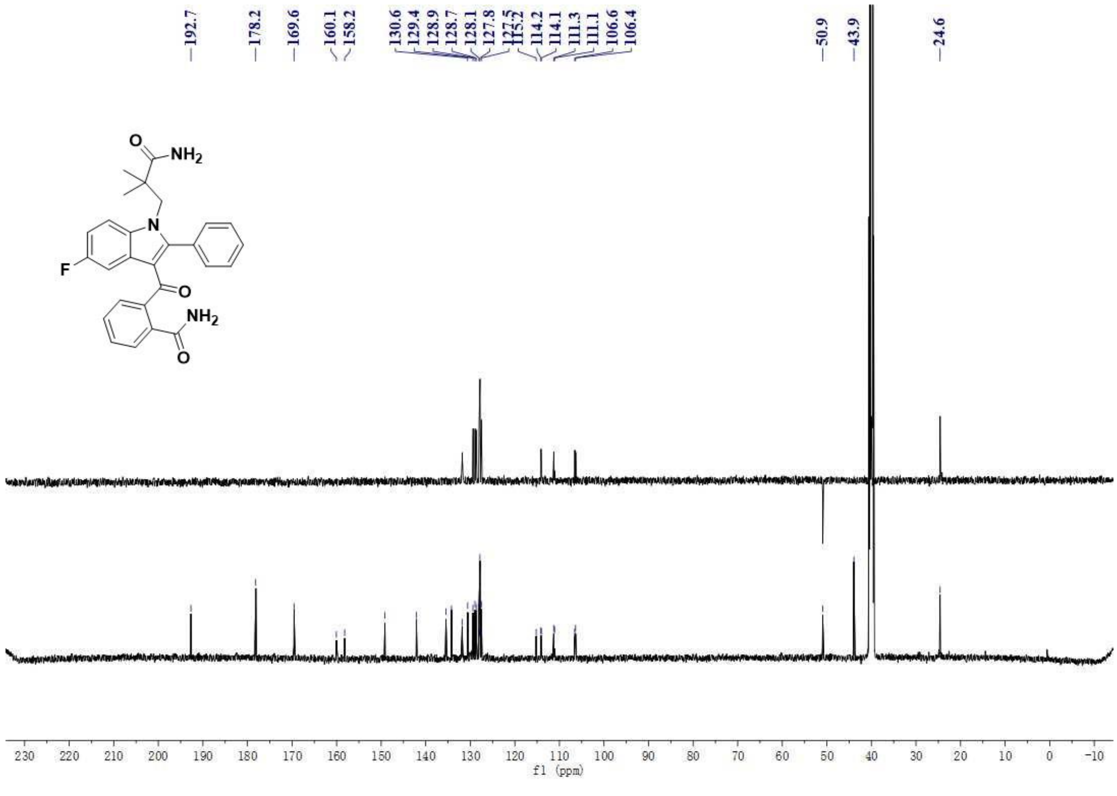

2-(1-(3-amino-2,2-dimethyl-3-oxopropyl)-5-chloro-2-phenyl-1*H*-indole-3-carbonyl)benzamide (5ca)

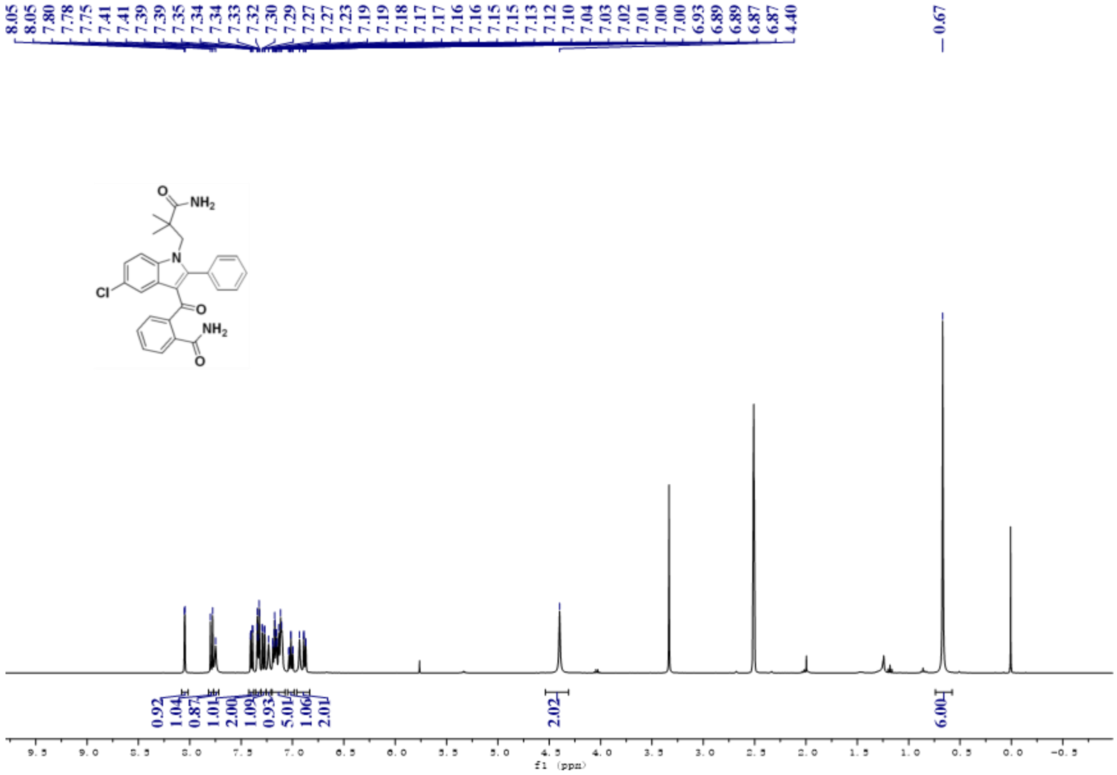

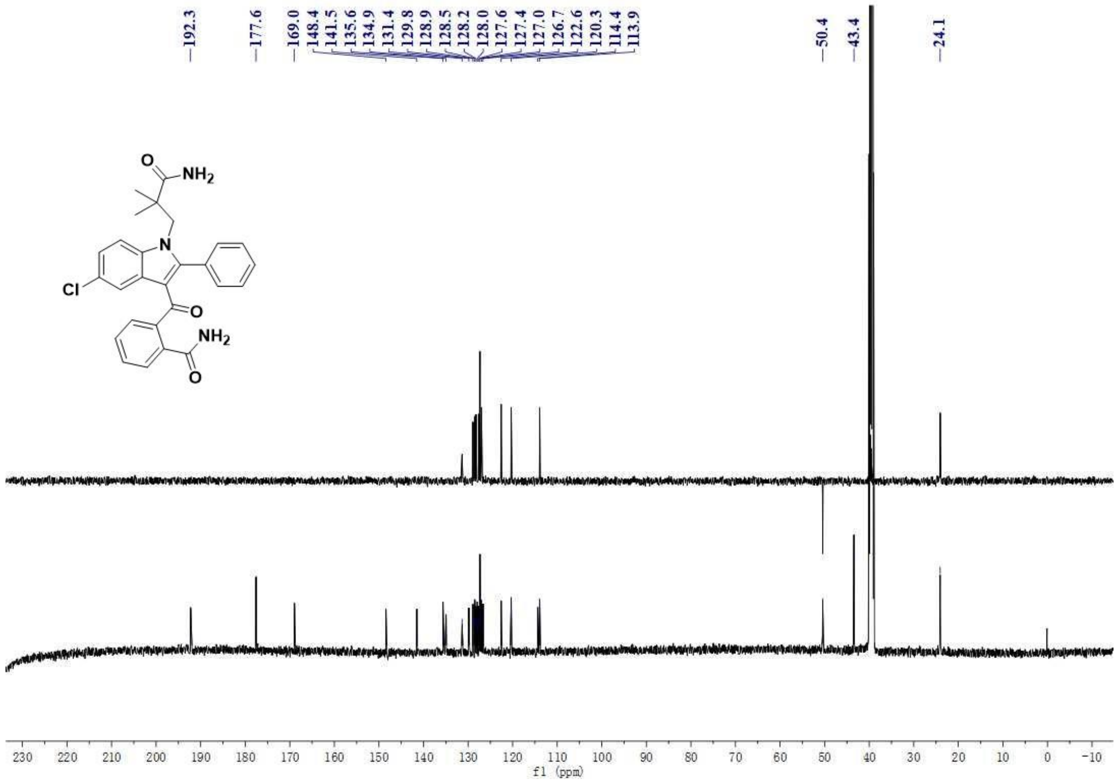

2-(1-(3-amino-2,2-dimethyl-3-oxopropyl)-5-methoxy-2-phenyl-1*H*-indole-3-carbonyl)benzamide (5fa)

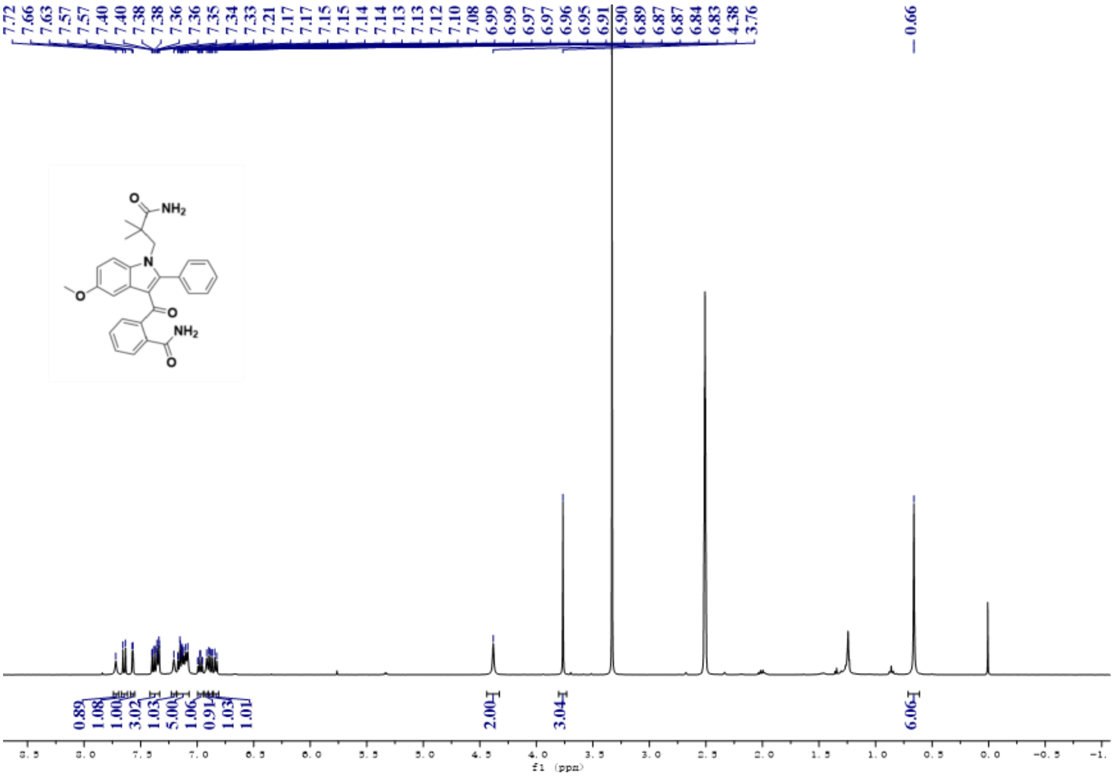

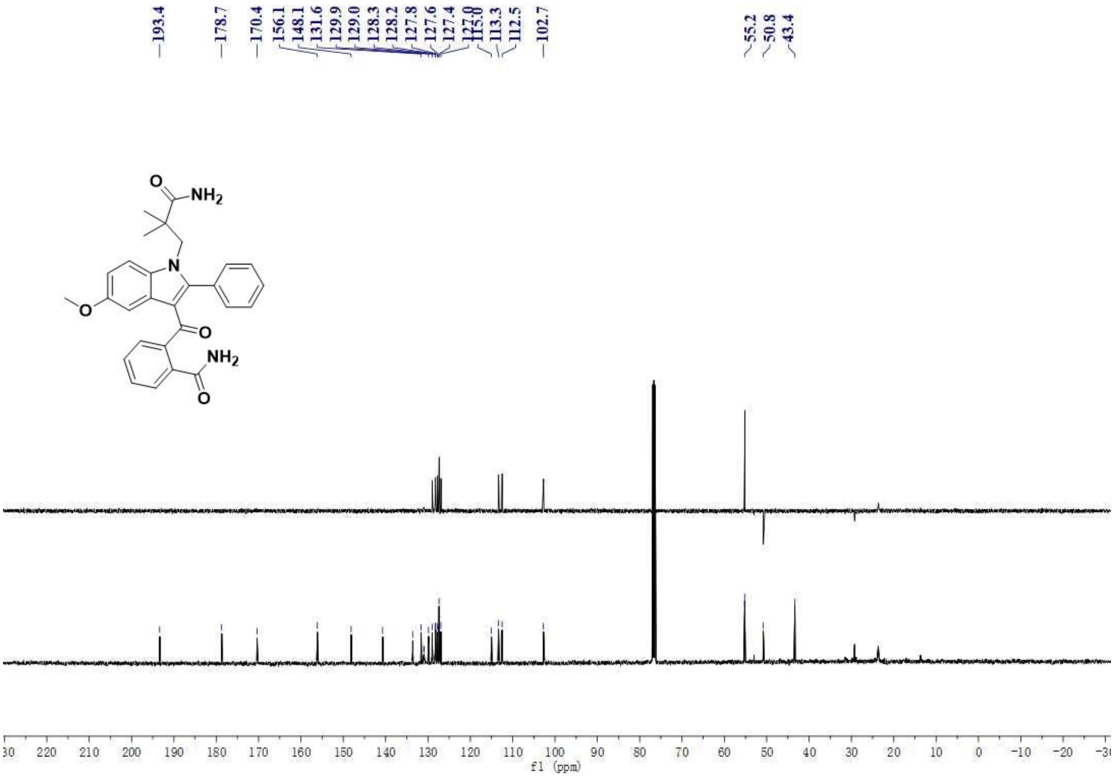

2-(1-(3-amino-2,2-dimethyl-3-oxopropyl)-2-(4-fluorophenyl)-1*H*-indole-3-carbonyl)benzamide (5ab)

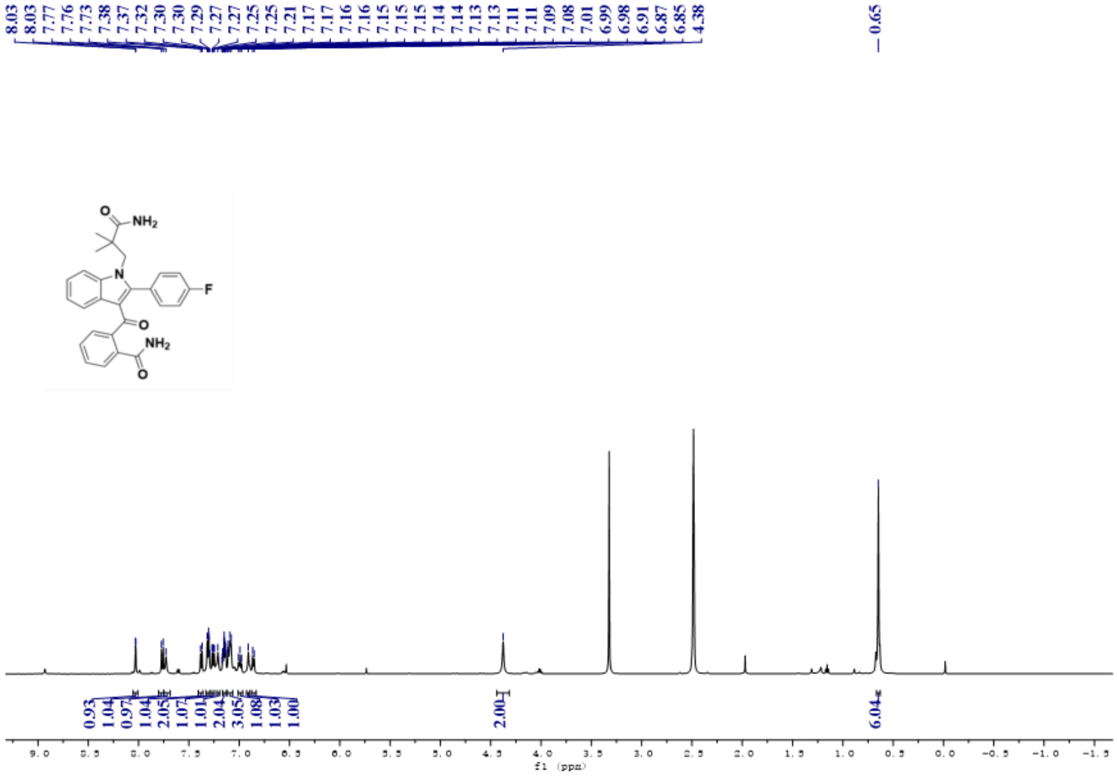

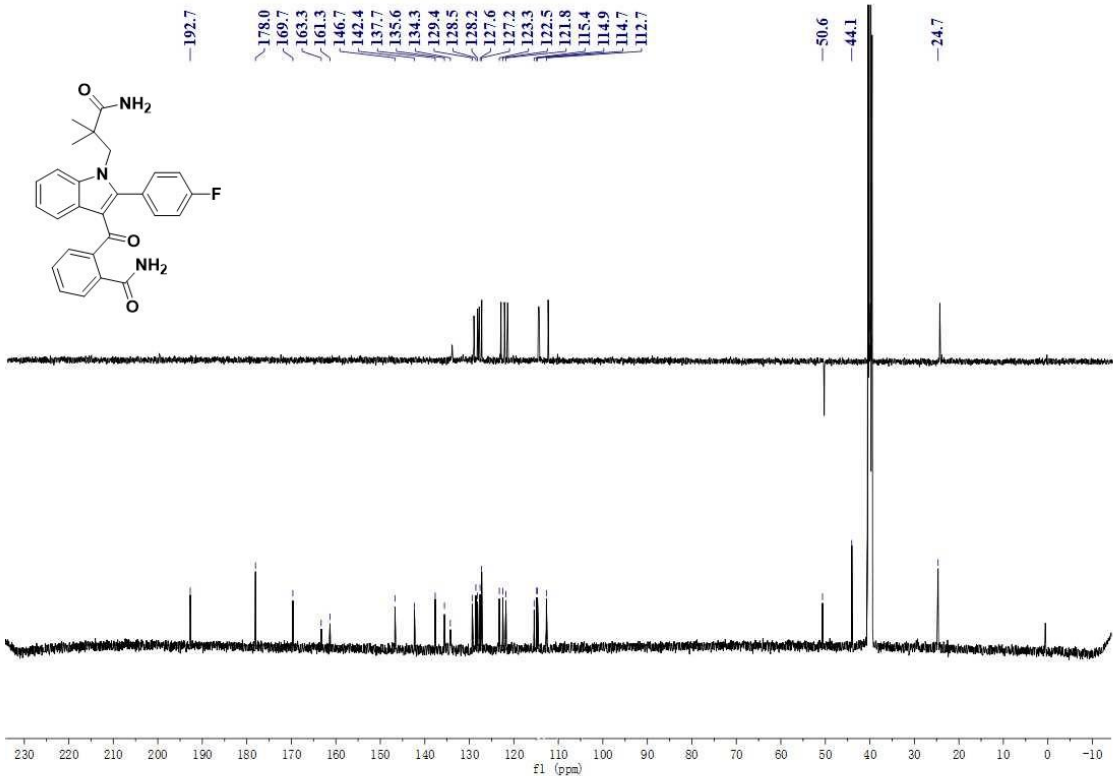

2-(1-(3-amino-2,2-dimethyl-3-oxopropyl)-2-(4-methoxyphenyl)-1*H*-indole-3-carbonyl)benzamide (5af)

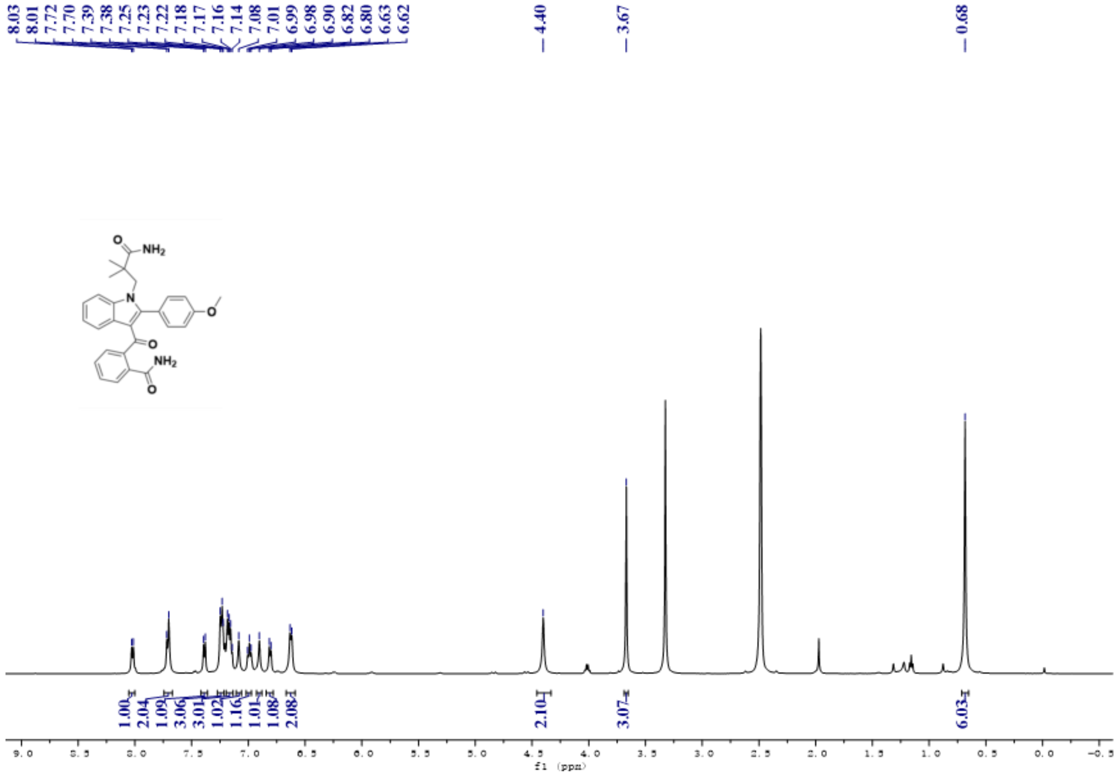

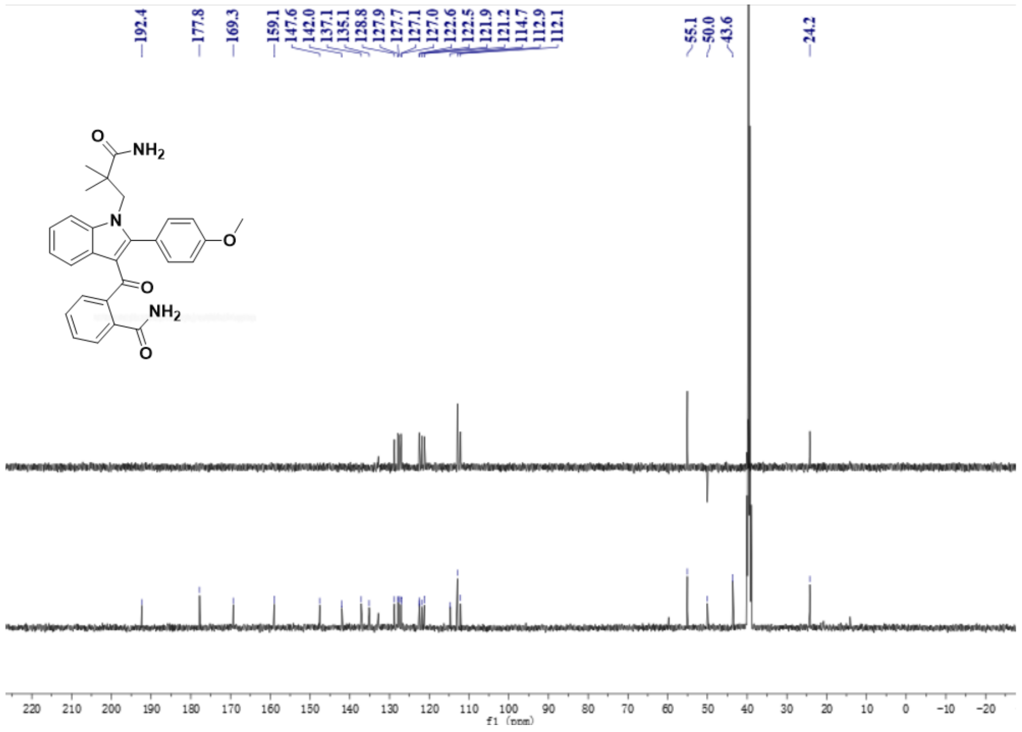

2-(1-(3-amino-2,2-dimethyl-3-oxopropyl)-2-(3-methylphenyl)-1*H*-indole-3-carbonyl)benzamide (5ai)

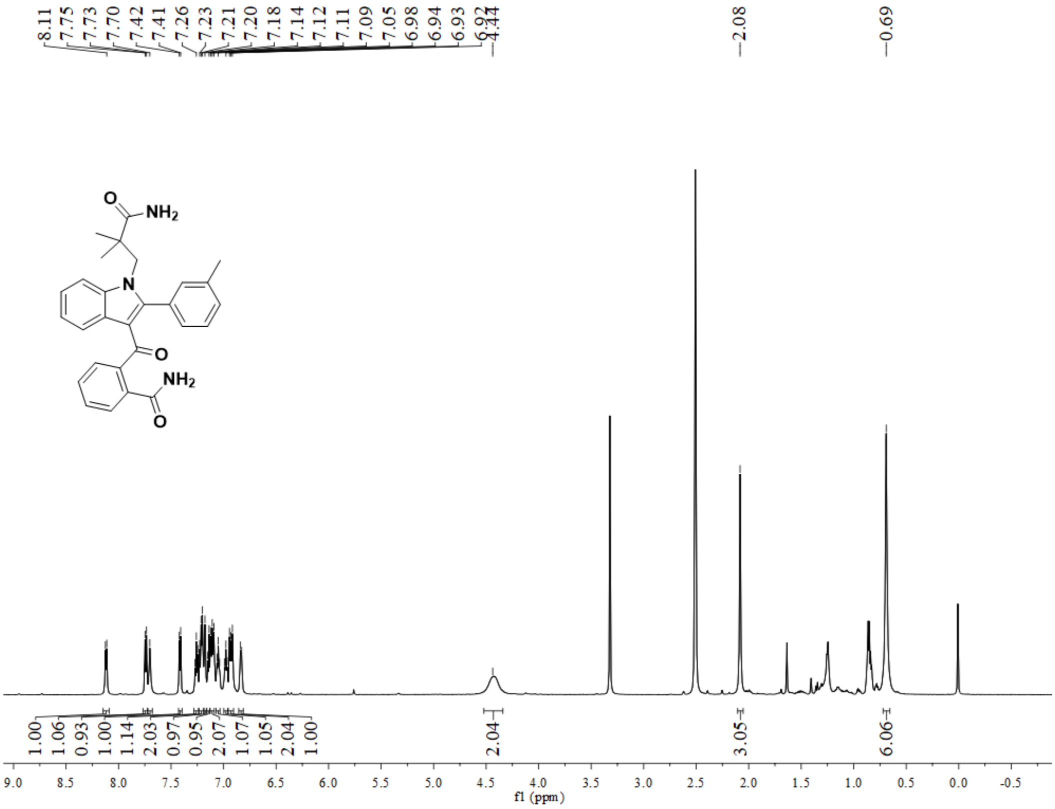

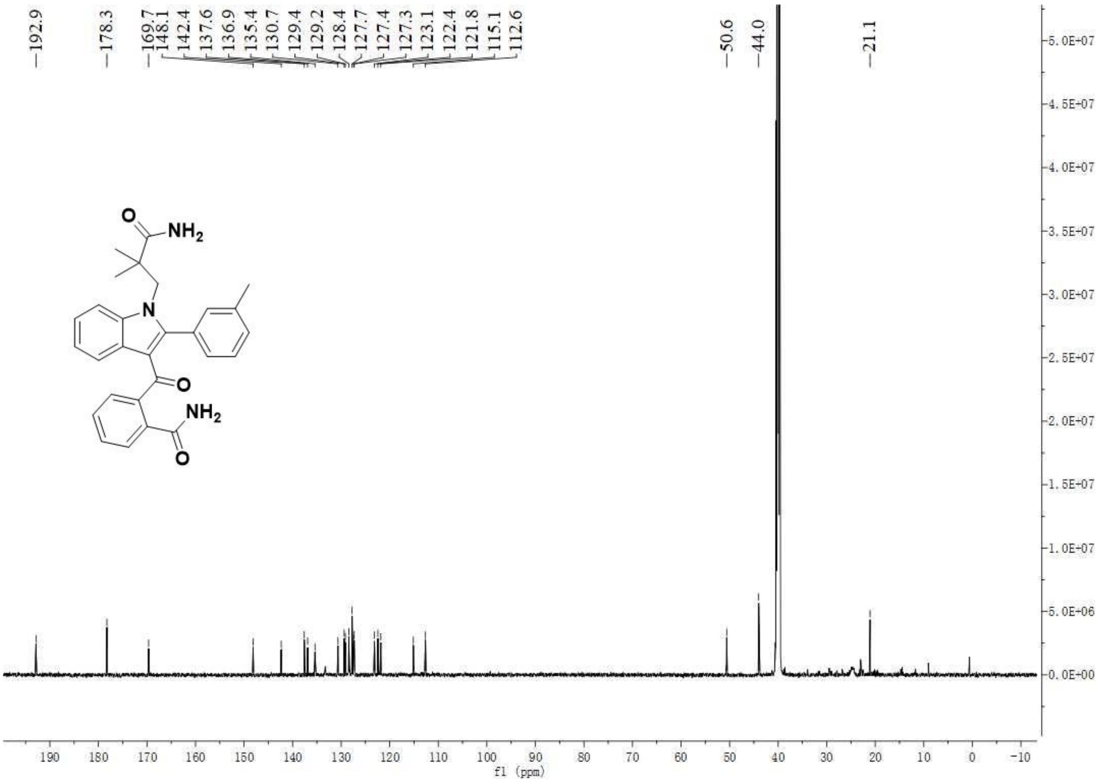

2-(1-(3-amino-2,2-dimethyl-3-oxopropyl)-2-(3-chlorophenyl)-1*H*-indole-3-carbonyl)benzamide (5ak)

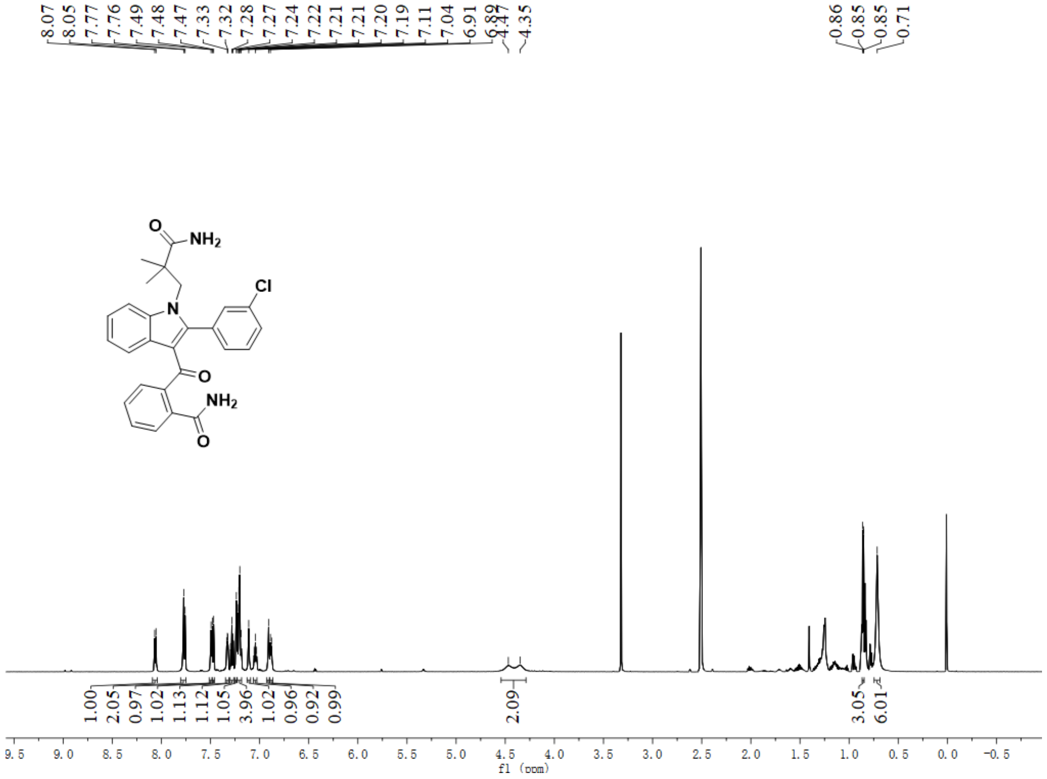

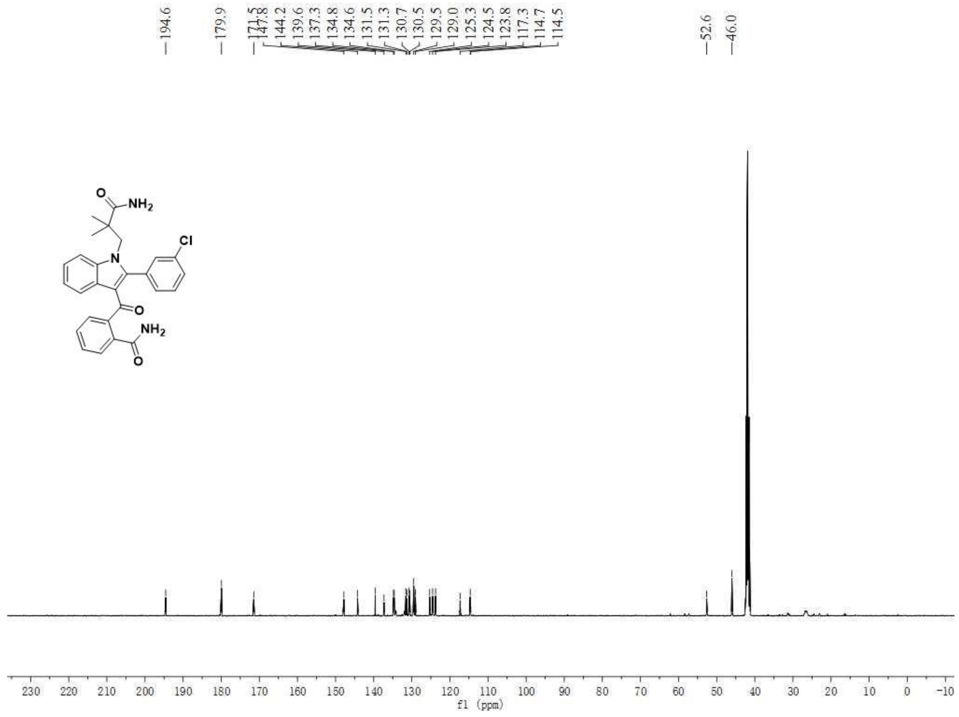

2-(1-(3-amino-2,2-dimethyl-3-oxopropyl)-2-(thiophen-3-yl)-1*H*-indole-3-carbonyl)benzamide (5al)

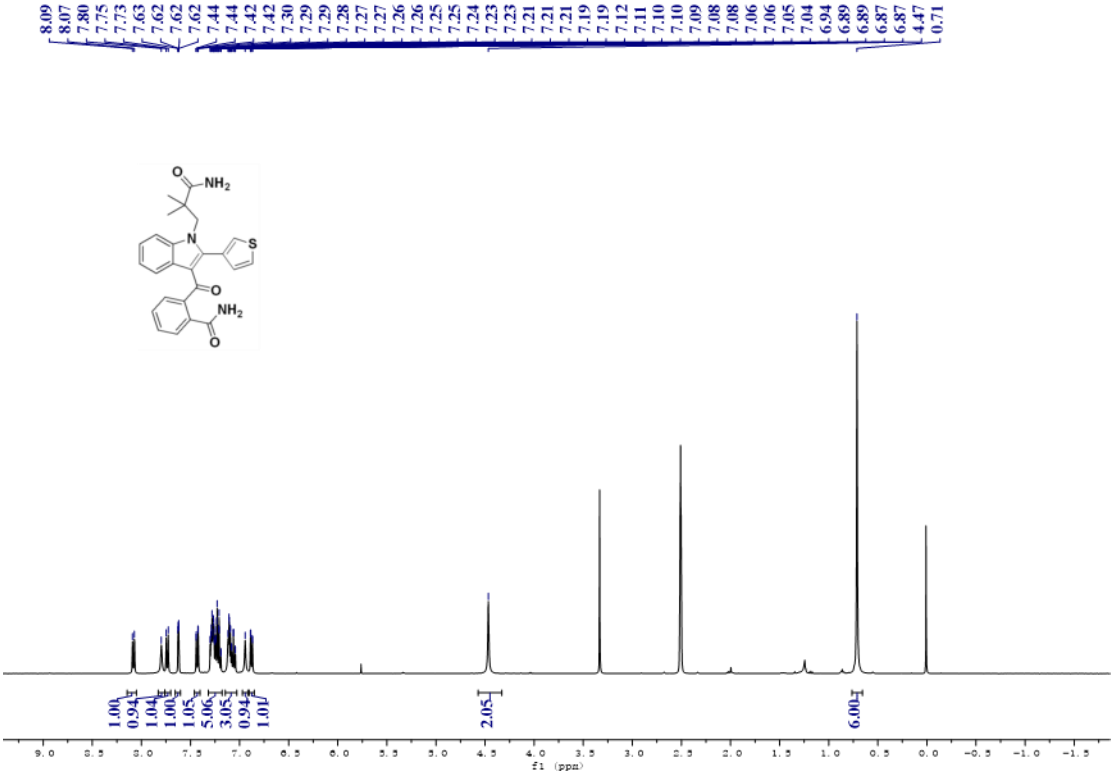

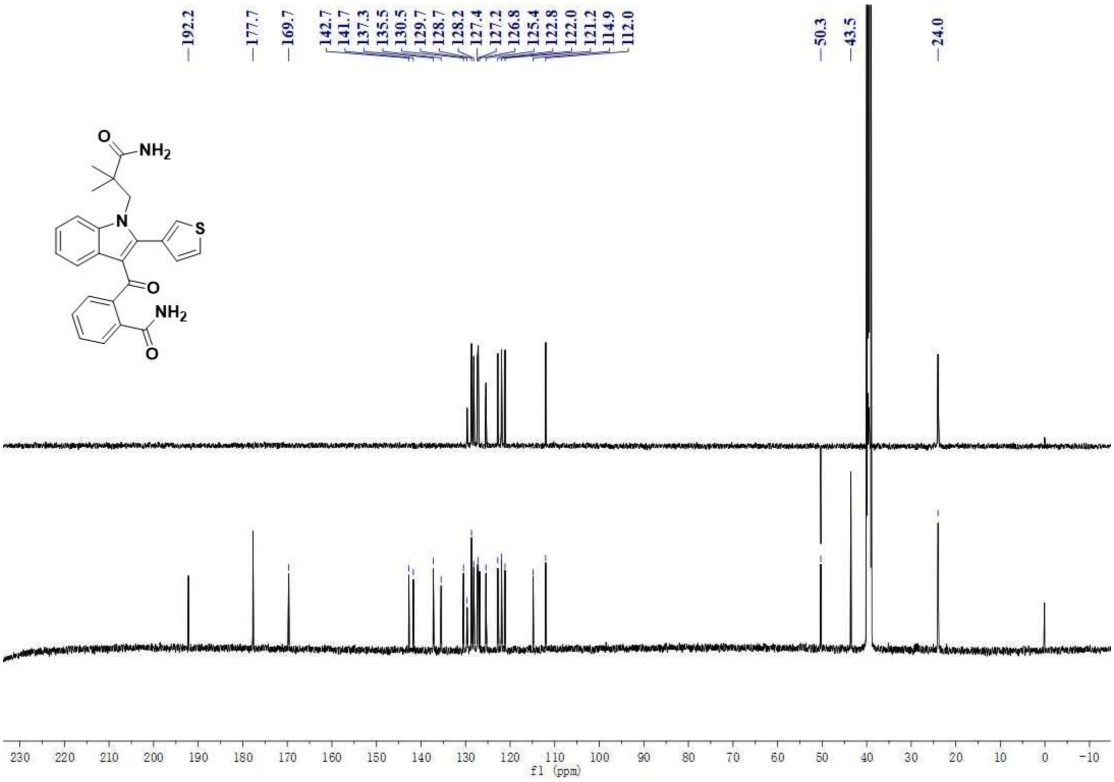

2-(1-(3-amino-2,2-dimethyl-3-oxopropyl)-5,6-dichloro-2-phenyl-1H-indole-3-carbonyl)benzamide (5am)

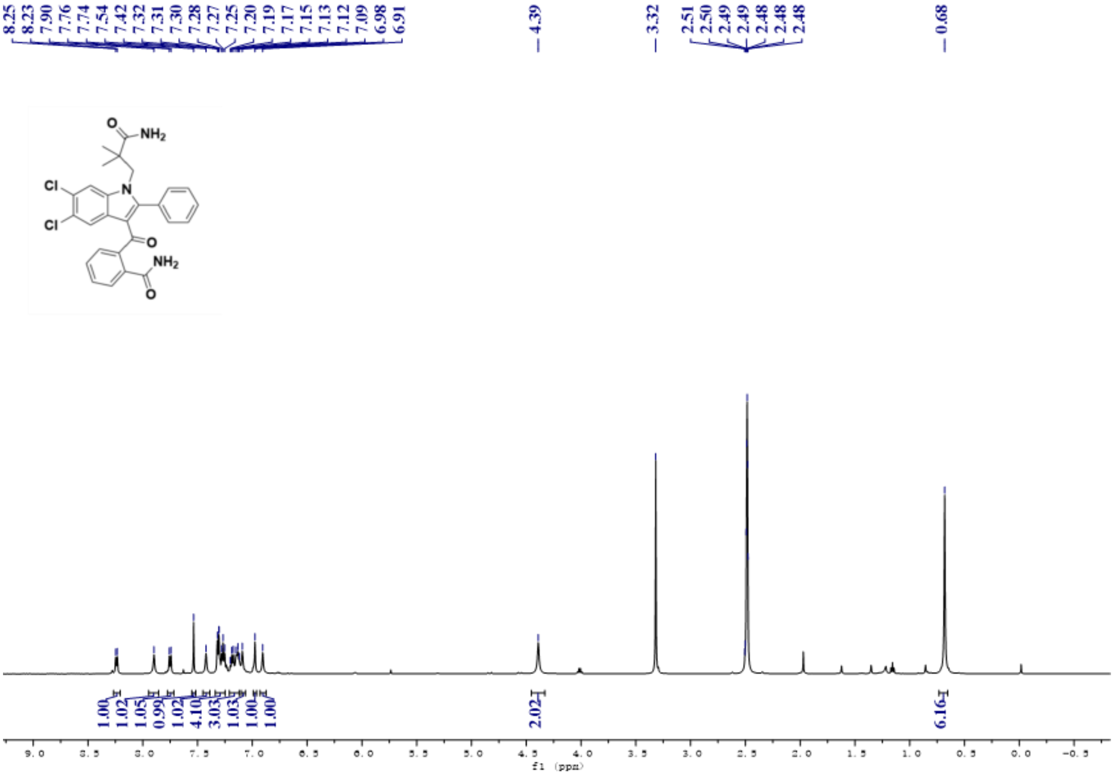

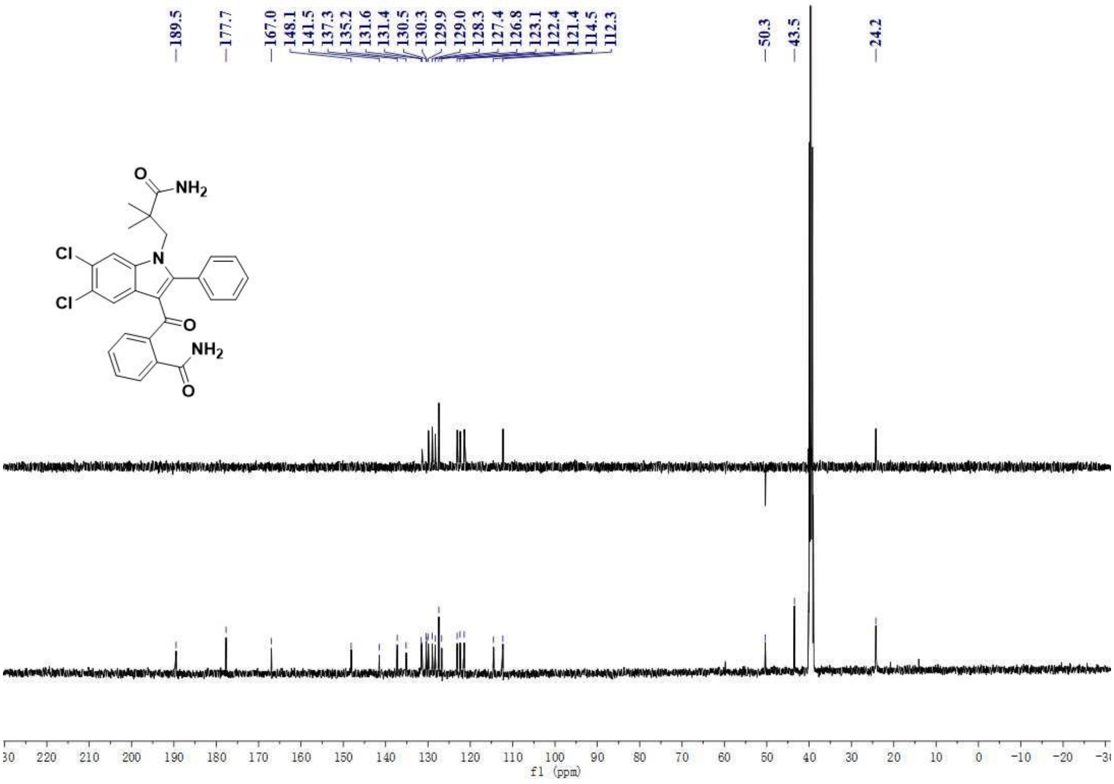

2,2-dimethyl-9-((3-oxoisoindolin-1-yl)methyl)-9-phenyl-2,3-dihydro-1*H*,9*H*-pyrazolo[1,2-a]indazol-1-one (6)

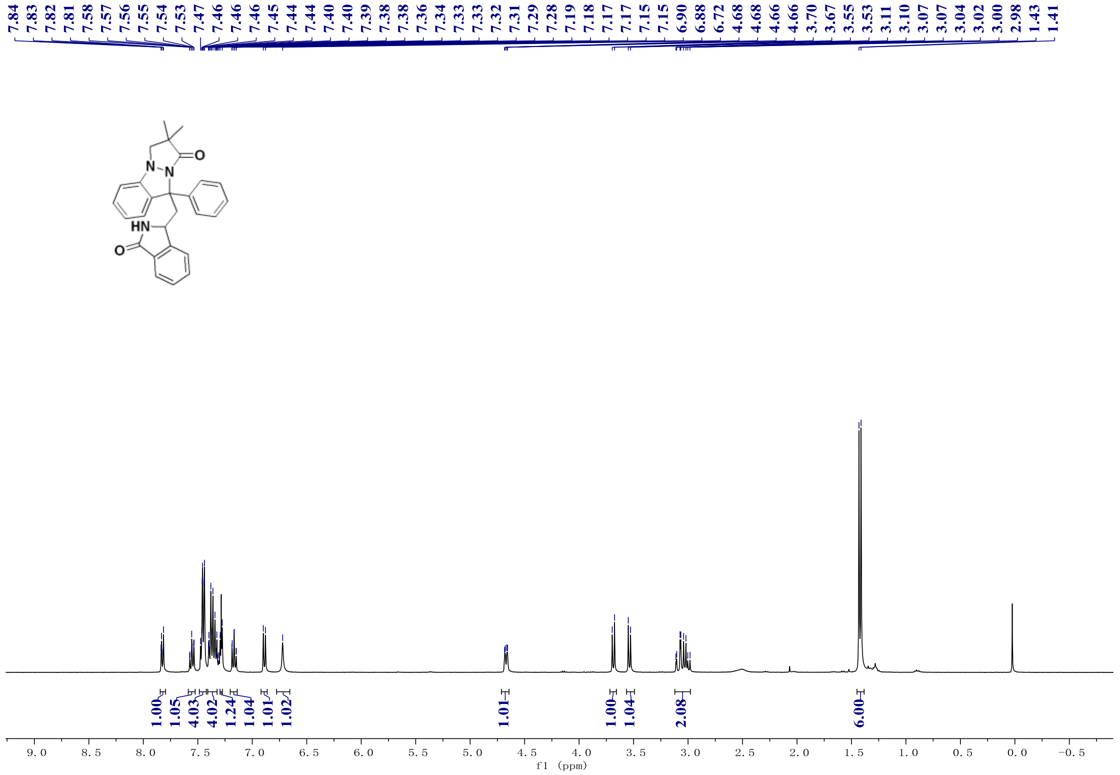

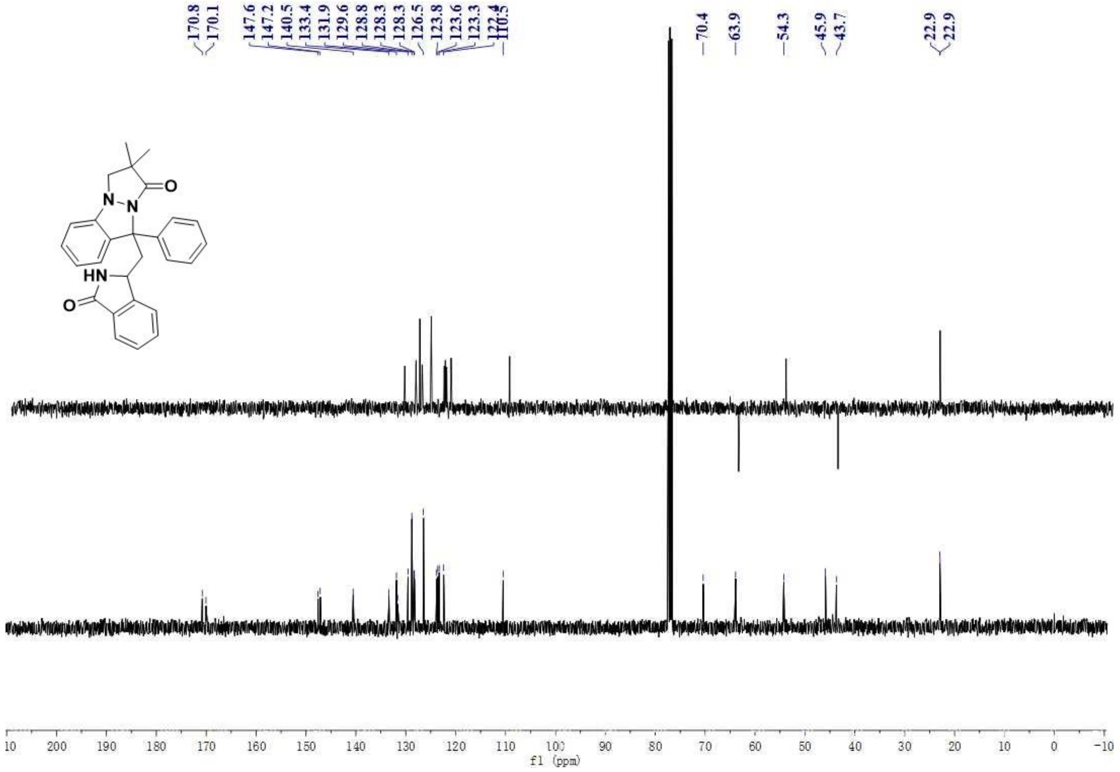

2-(1-(2-cyano-2-methylpropyl)-2-phenyl-1*H*-indole-3-carbonyl)benzonitrile (7)

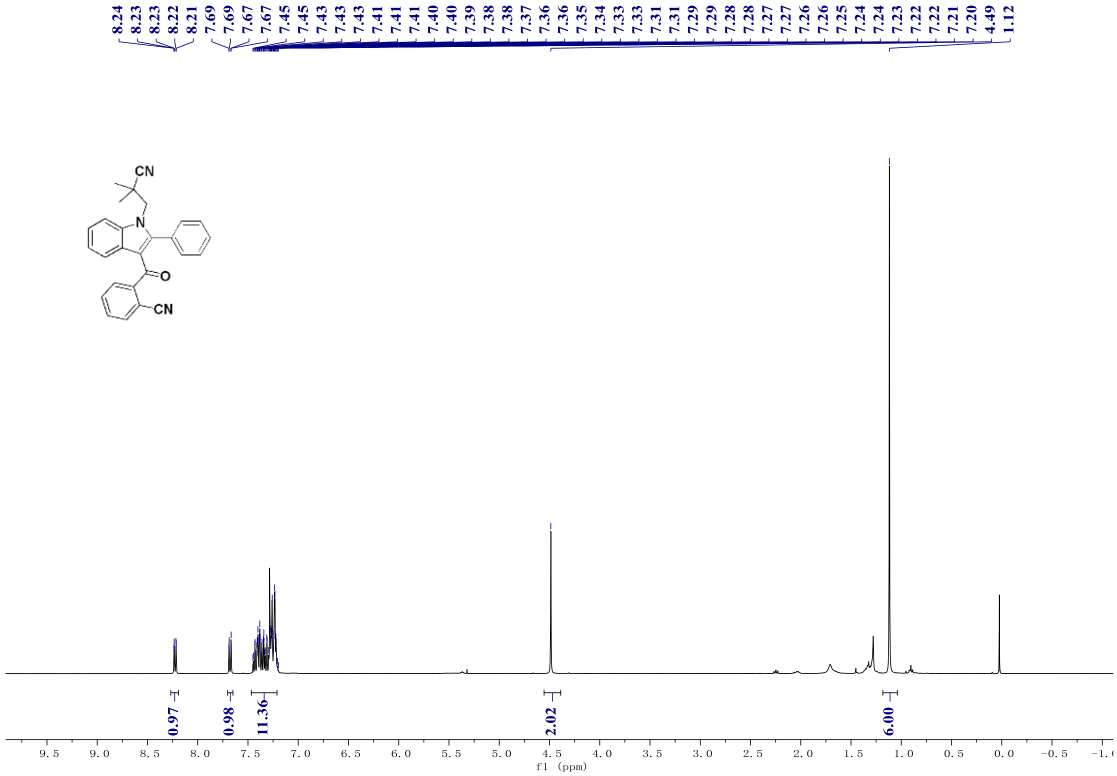

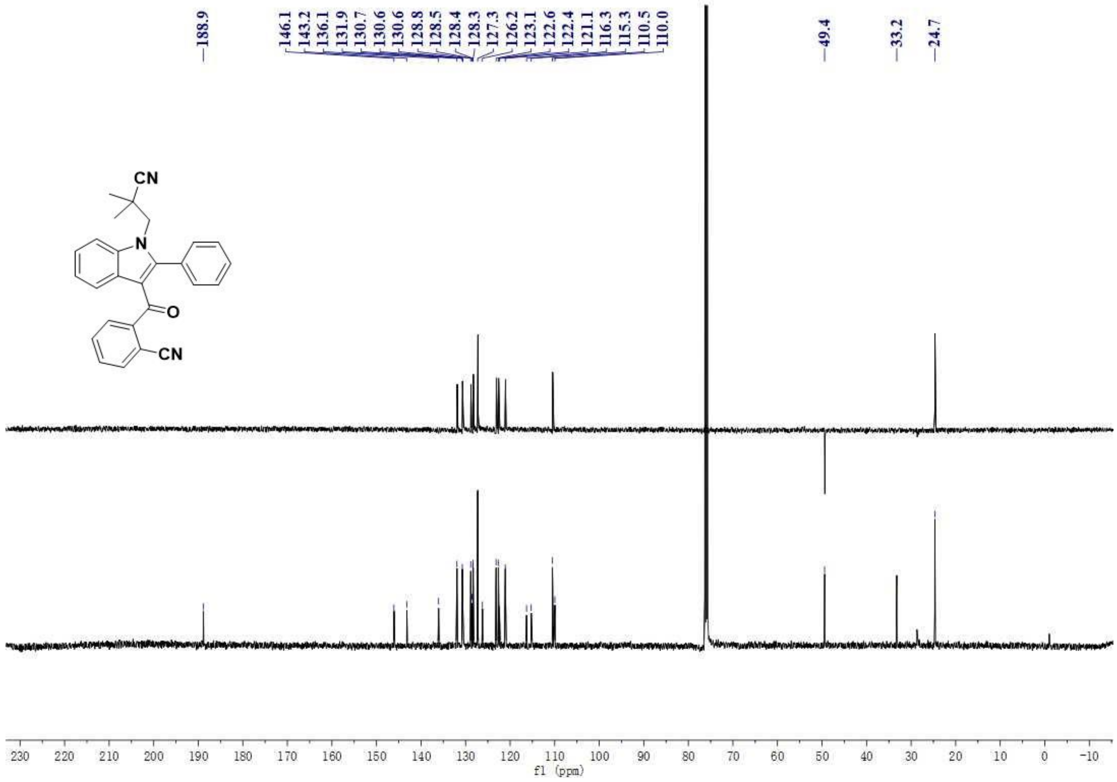

2,2-dimethyl-3-(3-(3-oxoisoindolin-1-yl)-2-phenyl-1*H*-indol-1yl)propenamide (8)

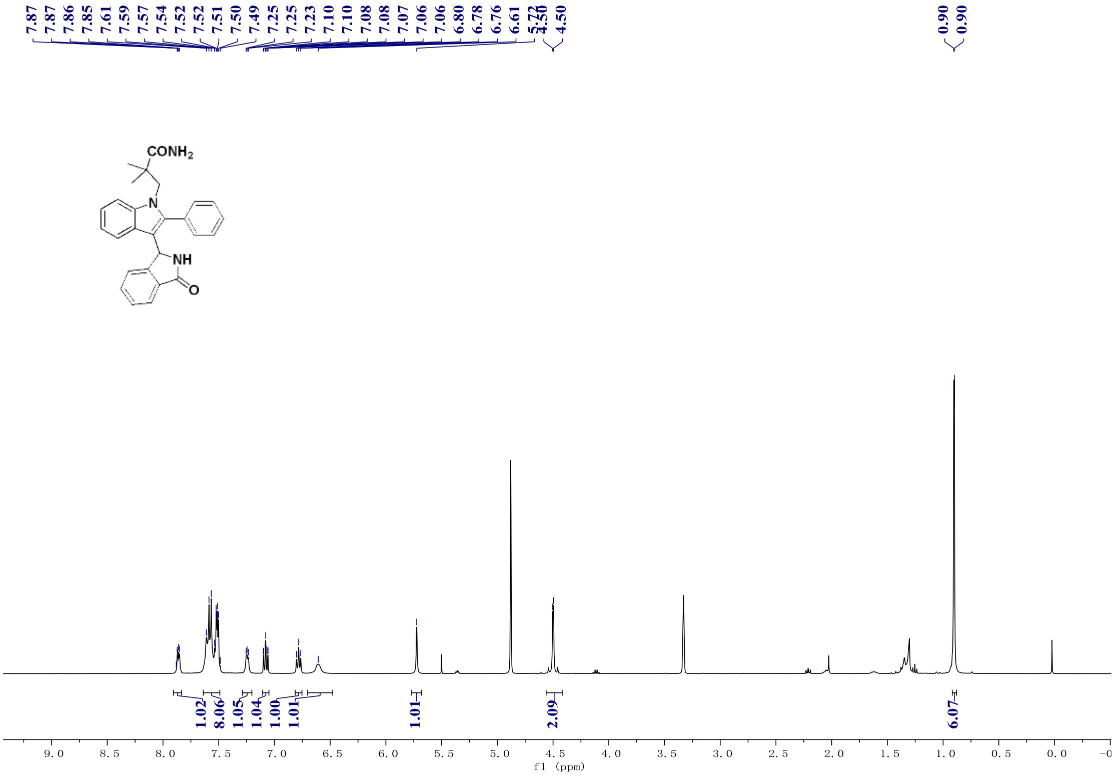

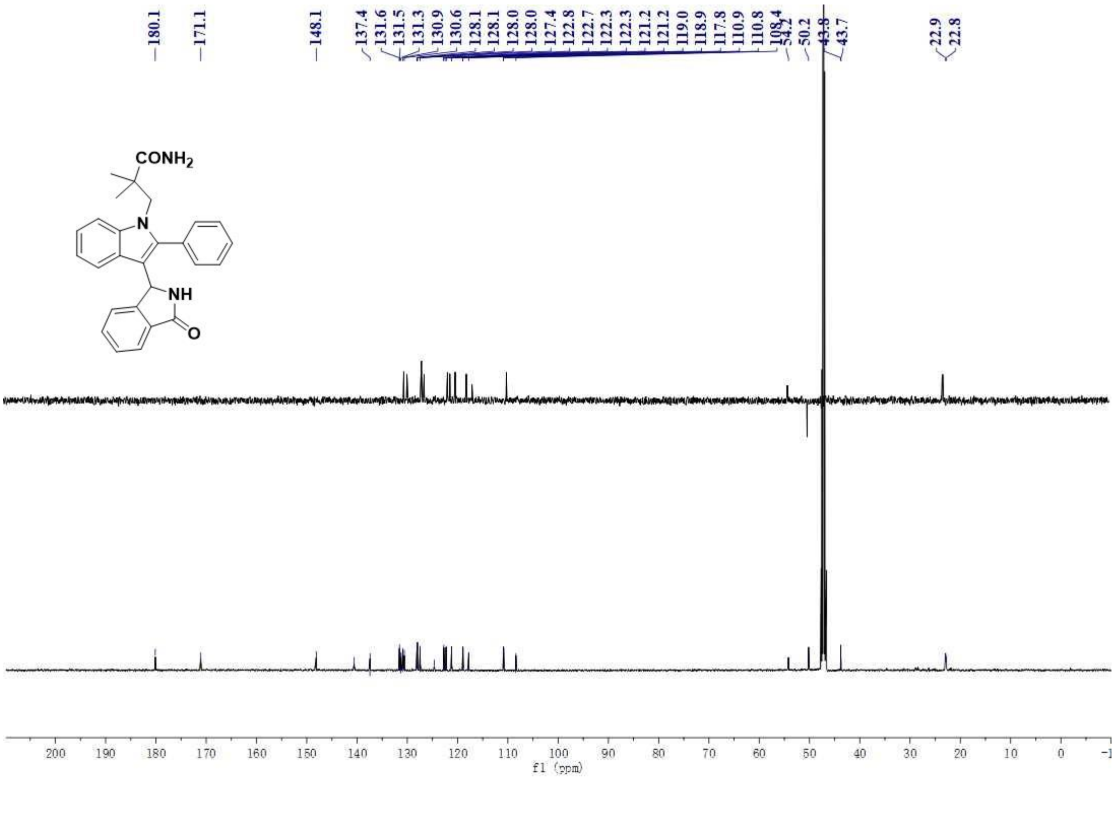

X. HPLC Data of Products

(*Z/E*)-3-((1-(4-methoxyphenyl)-5,5-dimethyl-4-oxo-2-phenylhexahydropyrimidin-2-yl)methylene)isoindolin-1-one (3fa)

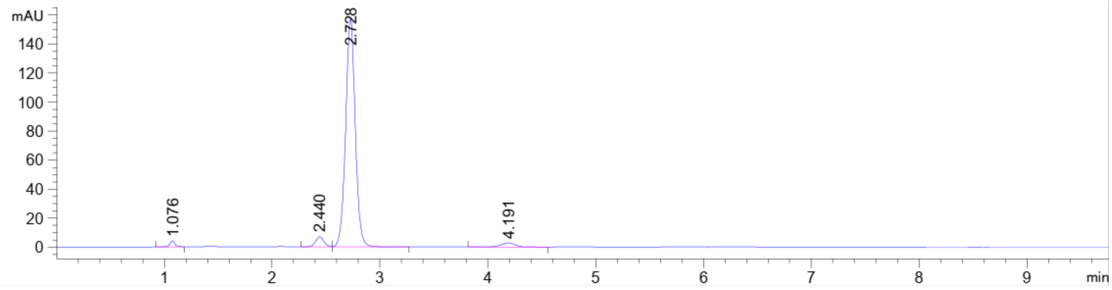

峰 #	保留时间 [min]	类型	峰宽 [min]	峰面积 [mAU*s]	峰高 [mAU]	峰面积 %
1	1.076	BB	0.0543	14.04102	4.06391	1.3356
2	2.440	BV	0.0840	38.33609	6.98852	3.6466
3	2.728	VB	0.0938	971.08032	157.76369	92.3719
4	4.191	BB	0.1447	27.81449	2.87882	2.6458

(*Z/E*)-3-((5,5-dimethyl-4-oxo-1-phenyl-2-(m-tolyl)hexahydropyrimidin-2-yl)methylene)isoindolin-1-one (3ai)

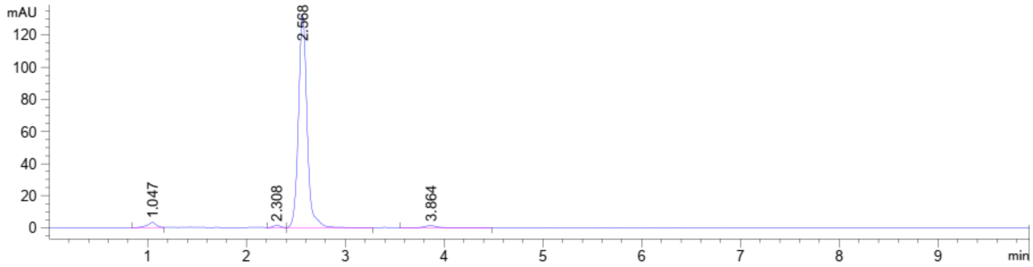

峰 #	保留时间 [min]	类型	峰宽 [min]	峰面积 [mAU*s]	峰高 [mAU]	峰面积 %
1	1.047	BB	0.0873	19.97430	3.18078	2.3687
2	2.308	BV	0.0747	7.43303	1.52927	0.8815
3	2.568	VB	0.0927	802.23846	132.39668	95.1352
4	3.864	BB	0.1411	13.61569	1.40390	1.6146

(*Z/E*)-3-((5,5-dimethyl-4-oxo-1-phenyl-2-(thiophen-3-yl)hexahydropyrimidin-2-yl)methylene)isoindolin-1-one (3al)

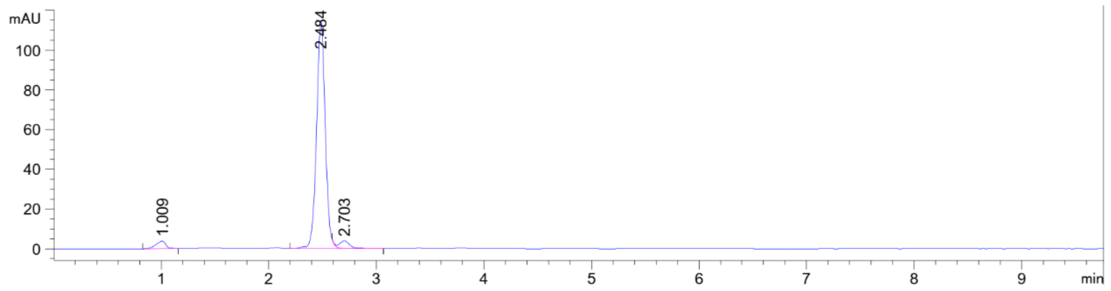

峰 #	保留时间 [min]	类型	峰宽 [min]	峰面积 [mAU*s]	峰高 [mAU]	峰面积 %
1	1.009	BB	0.0908	24.35287	3.79697	3.4850
2	2.484	BV R	0.0861	648.51910	114.44514	92.8065
3	2.703	VB E	0.0984	25.91402	3.85796	3.7084

(*Z/E*)-6-fluoro-2,2-dimethyl-9-((3-oxoisoindolin-1-ylidene)methyl)-9-phenyl-2,3-dihydro-1*H*,9H-pyrazolo[1,2-a]indazol-1-one (4ia)

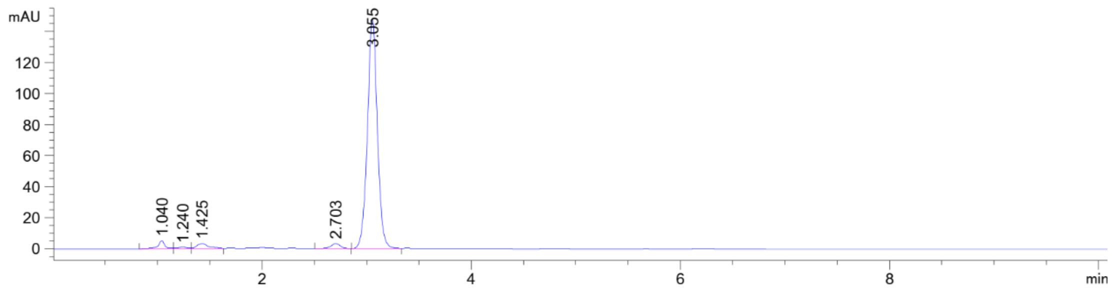

峰 #	保留时间 [min]	类型	峰宽 [min]	峰面积 [mAU*s]	峰高 [mAU]	峰面积 %
1	1.040	BV	0.0634	22.78896	5.17395	2.1785
2	1.240	VV	0.0759	5.87707	1.07313	0.5618
3	1.425	VB	0.1164	22.80117	3.00785	2.1796
4	2.703	BB	0.0927	19.26088	3.17848	1.8412
5	3.055	BB	0.1010	975.37708	147.83421	93.2389

(*Z/E*)-6-chloro-2,2-dimethyl-9-((3-oxoisoindolin-1-ylidene)methyl)-9-phenyl-2,3-dihydro-1*H*,9*H*-pyrazolo[1,2-*a*]indazol-1-one (4ja)

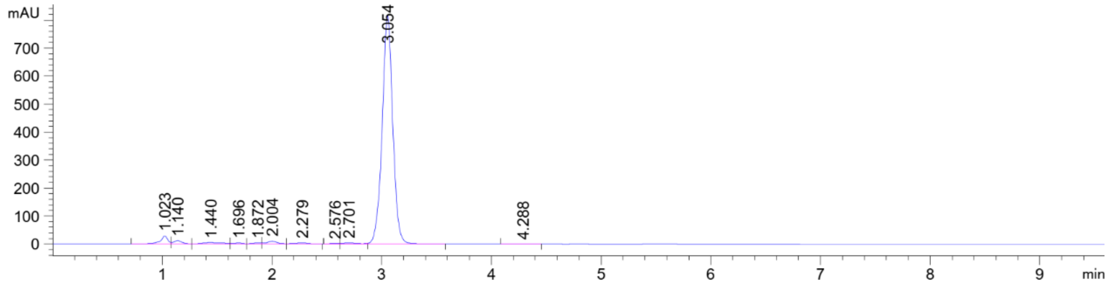

峰 #	保留时间 [min]	类型	峰宽 [min]	峰面积 [mAU*s]	峰高 [mAU]	峰面积 %
1	1.023	BV	0.0665	132.13000	28.26178	2.2374
2	1.140	VB	0.0751	57.38038	11.31824	0.9716
3	1.440	BV	0.1365	46.54298	4.43126	0.7881
4	1.696	VB	0.0671	12.11019	2.76519	0.2051
5	1.872	BV	0.0746	12.96139	2.66935	0.2195
6	2.004	VB	0.0832	50.64160	8.79382	0.8575
7	2.279	BB	0.0945	23.67161	3.61219	0.4008
8	2.576	BV E	0.0807	9.51313	1.82884	0.1611
9	2.701	VV E	0.1082	32.24899	4.36544	0.5461
10	3.054	VB R	0.1029	5517.39697	815.78876	93.4267
11	4.288	BB	0.1353	10.99170	1.24193	0.1861

(*Z/E*)-2,2-dimethyl-9-((3-oxoisoindolin-1-ylidene)methyl)-9-(thiophen-3-yl)-2,3-dihydro-1*H*,9*H*-pyrazolo[1,2-*a*]indazol-1-one (4al)

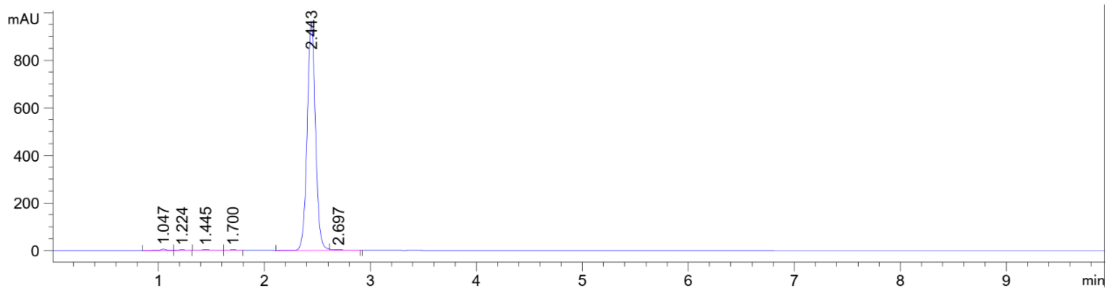

峰 #	保留时间 [min]	类型	峰宽 [min]	峰面积 [mAU*s]	峰高 [mAU]	峰面积 %
1	1.047	BV	0.0610	21.83553	5.19918	0.4130
2	1.224	VB	0.0526	12.57133	3.61460	0.2378
3	1.445	BB	0.0983	18.15282	2.63877	0.3434
4	1.700	BV	0.0918	9.08126	1.61106	0.1718
5	2.443	BV R	0.0812	5210.88867	961.96759	98.5612
6	2.697	VB E	0.0905	14.42750	2.32060	0.2729

2-(1-(3-amino-2,2-dimethyl-3-oxopropyl)-2-(4-methoxyphenyl)-1*H*-indole-3-carbonyl)benzamide (5af)

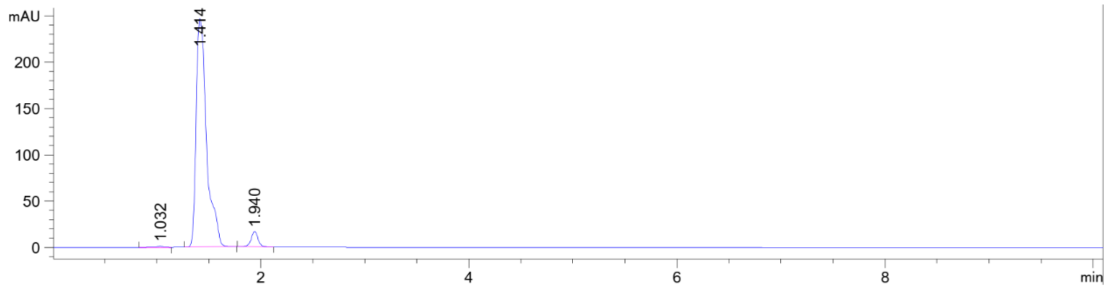

峰 #	保留时间 [min]	类型	峰宽 [min]	峰面积 [mAU*s]	峰高 [mAU]	峰面积 %
1	1.032	BB	0.0648	5.48077	1.21180	0.3027
2	1.414	BB	0.1096	1726.49805	246.97122	95.3599
3	1.940	BB	0.0707	78.52799	16.76131	4.3373

2-(1-(3-amino-2,2-dimethyl-3-oxopropyl)-2-(3-methylphenyl)-1*H*-indole-3-carbonyl)benzamide (5ai)

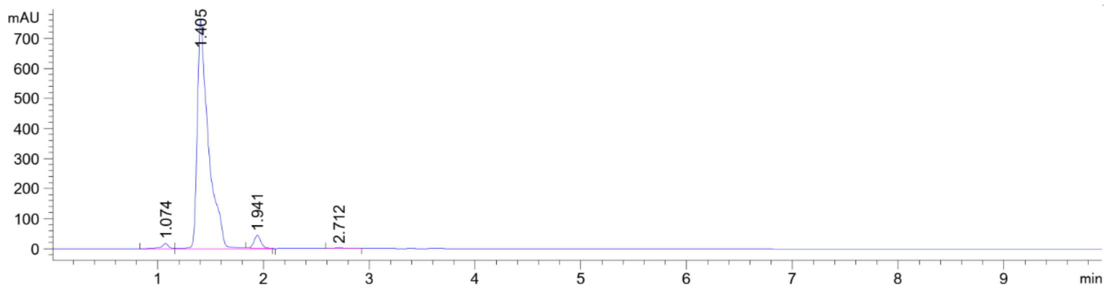

峰 #	保留时间 [min]	类型	峰宽 [min]	峰面积 [mAU*s]	峰高 [mAU]	峰面积 %
1	1.074	BV	0.0651	77.66707	17.72319	1.3090
2	1.405	VV R	0.1027	5650.14258	759.90240	95.2278
3	1.941	VB E	0.0688	192.32324	42.52042	3.2414
4	2.712	BB	0.0985	13.15908	2.00787	0.2218

2-(1-(3-amino-2,2-dimethyl-3-oxopropyl)-2-(3-chlorophenyl)-1*H*-indole-3-carbonyl)benzamide (5ak)

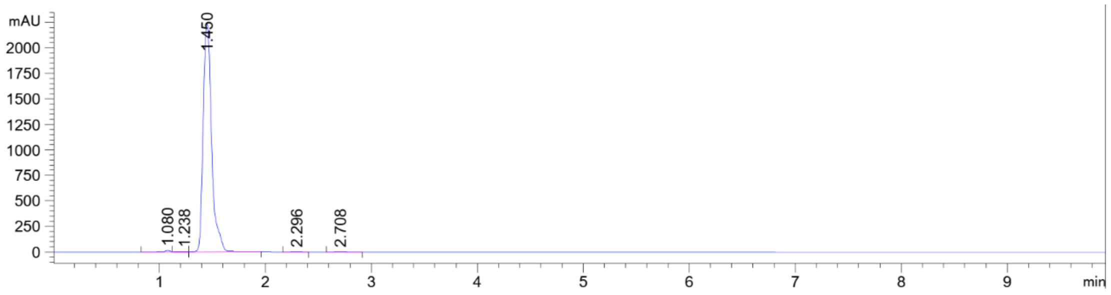

峰 #	保留时间 [min]	类型	峰宽 [min]	峰面积 [mAU*s]	峰高 [mAU]	峰面积 %
1	1.080	BV R	0.0554	45.99453	12.35875	0.3714
2	1.238	VV E	0.0829	12.61239	2.19988	0.1018
3	1.450	VB	0.0862	1.23102e4	2238.36646	99.4051
4	2.296	BB	0.0757	6.71095	1.35656	0.0542
5	2.708	BB	0.0967	8.34928	1.30432	0.0674
