# Supplementary material for: Catalytic system-controlled divergent reactions of pyrazolidinones with 3-alkynyl-3-hydroxyisoindolinones to construct diversified nitrogen-containing heterocyclic scaffolds
Source: RSC Adv. 2025 Jun 26;15(27):21872–8. doi: 10.1039/d5ra01992c (PMC12198952; doi:10.1039/d5ra01992c)
Supplement: RA-015-D5RA01992C-s002 [file RA-015-D5RA01992C-s002.pdf]

No syntax errors found.
Please wait while processing ....

CIF dictionary
Interpreting this report

Datablock: cu_20221176_0m

Bond precision: C-C = 0.0073 A Wavelength=1.54178

Cell: a=10.9888(2) b=18.4115(5) c=12.1884(3)
alpha=90 beta=109.374(1) gamma=90

Temperature: 170 K

	Calculated	Reported
Volume	2326.32(10)	2326.32(10)
Space group	P 21/c	P 1 21/c 1
Hall group	-P 2ybc	-P 2ybc
Moiety formula	2(C27 H25 N3 O2), H2 O	2(C27 H25 N3 O2), H2 O
Sum formula	C54 H52 N6 O5	C54 H52 N6 O5
Mr	865.02	865.01
Dx,g cm-3	1.235	1.235
Z	2	2
Mu (mm-1)	0.640	0.640
F000	916.0	916.0
F000'	918.66	
h,k,lmax	13,23,15	13,23,15
Nref	4763	4691
Tmin,Tmax	0.940,0.969	0.736,0.929
Tmin'	0.926	

Correction method= # Reported T Limits: Tmin=0.736 Tmax=0.929 AbsCorr = MULTI-SCAN

Data completeness= 0.985 Theta(max)= 74.618

R(reflections)= 0.1021(4218) wR2(reflections)= 0.2371(4691)

S = 1.100 Npar= 304

The following ALERTS were generated. Each ALERT has the format
test-name_ALERT_alert-type_alert-level.
Click on the hyperlinks for more details of the test.

Alert level B

PLAT420_ALERT_2_B D-H Bond Without Acceptor O3 --H3C . Please Check

Alert level C

PLAT340_ALERT_3_C Low Bond Precision on C-C Bonds .... 0.00733 Ang.

Alert level G

PLAT007_ALERT_5_G Number of Unrefined Donor-H Atoms .... 4 Report
H2 H3 H3B H3C
PLAT012_ALERT_1_G No _shelx_res_checksum Found in CIF ...... Please Check
PLAT083_ALERT_2_G SHELXL Second Parameter in WGHT Unusually Large 13.05 Why ?
PLAT299_ALERT_4_G Atom Site Occupancy Constrained at .... 0.5 Check
O3 H3B H3C
PLAT302_ALERT_4_G Anion/Solvent/Minor-Residue Disorder (Resd 2) 100% Note
PLAT304_ALERT_4_G Non-Integer Number of Atoms in ..... (Resd 2) 1.50 Check
PLAT793_ALERT_4_G Model has Chirality at C10 (Centro SpGr) S Verify
PLAT860_ALERT_3_G Number of Least-Squares Restraints .... 3 Note
PLAT933_ALERT_2_G Number of HKL-OMIT Records in Embedded .res File 12 Note
-1 1 12, -1 2 10, 1 2 6, 1 3 7, 1 8 8, 2 4 4,
2 5 6, 3 2 5, 5 8 5, 7 6 4, 9 1 6, 10 1 2,
PLAT941_ALERT_3_G Average HKL Measurement Multiplicity .... 4.8 Low

0 **ALERT level A** = Most likely a serious problem - resolve or explain
1 **ALERT level B** = A potentially serious problem, consider carefully
1 **ALERT level C** = Check. Ensure it is not caused by an omission or oversight
10 **ALERT level G** = General information/check it is not something unexpected

1 ALERT type 1 CIF construction/syntax error, inconsistent or missing data
3 ALERT type 2 Indicator that the structure model may be wrong or deficient
3 ALERT type 3 Indicator that the structure quality may be low
4 ALERT type 4 Improvement, methodology, query or suggestion
1 ALERT type 5 Informative message, check

It is advisable to attempt to resolve as many as possible of the alerts in all categories. Often the minor alerts point to easily fixed oversights, errors and omissions in your CIF or refinement strategy, so attention to these fine details can be worthwhile. In order to resolve some of the more serious problems it may be necessary to carry out additional measurements or structure refinements. However, the purpose of your study may justify the reported deviations and the more serious of these should normally be commented upon in the discussion or experimental section of a paper or in the "special_details" fields of the CIF. checkCIF was carefully designed to identify outliers and unusual parameters, but every test has its limitations and alerts that are not important in a particular case may appear. Conversely, the absence of alerts does not guarantee there are no aspects of the results needing attention. It is up to the individual to critically assess their own results and, if necessary, seek expert advice.

Publication of your CIF in IUCr journals

A basic structural check has been run on your CIF. These basic checks will be run on all CIFs submitted for publication in IUCr journals (*Acta Crystallographica*, *Journal of Applied Crystallography*, *Journal of Synchrotron Radiation*); however, if you intend to submit to *Acta Crystallographica Section C* or *E* or *IUCrData*, you should make sure that full publication checks are run on the final version of your CIF prior to submission.

Publication of your CIF in other journals

Please refer to the *Notes for Authors* of the relevant journal for any special instructions relating to CIF submission.

PLATON version of 02/02/2025; check.def file version of 02/02/2025

Datablock cu_20221176_0m - ellipsoid plot

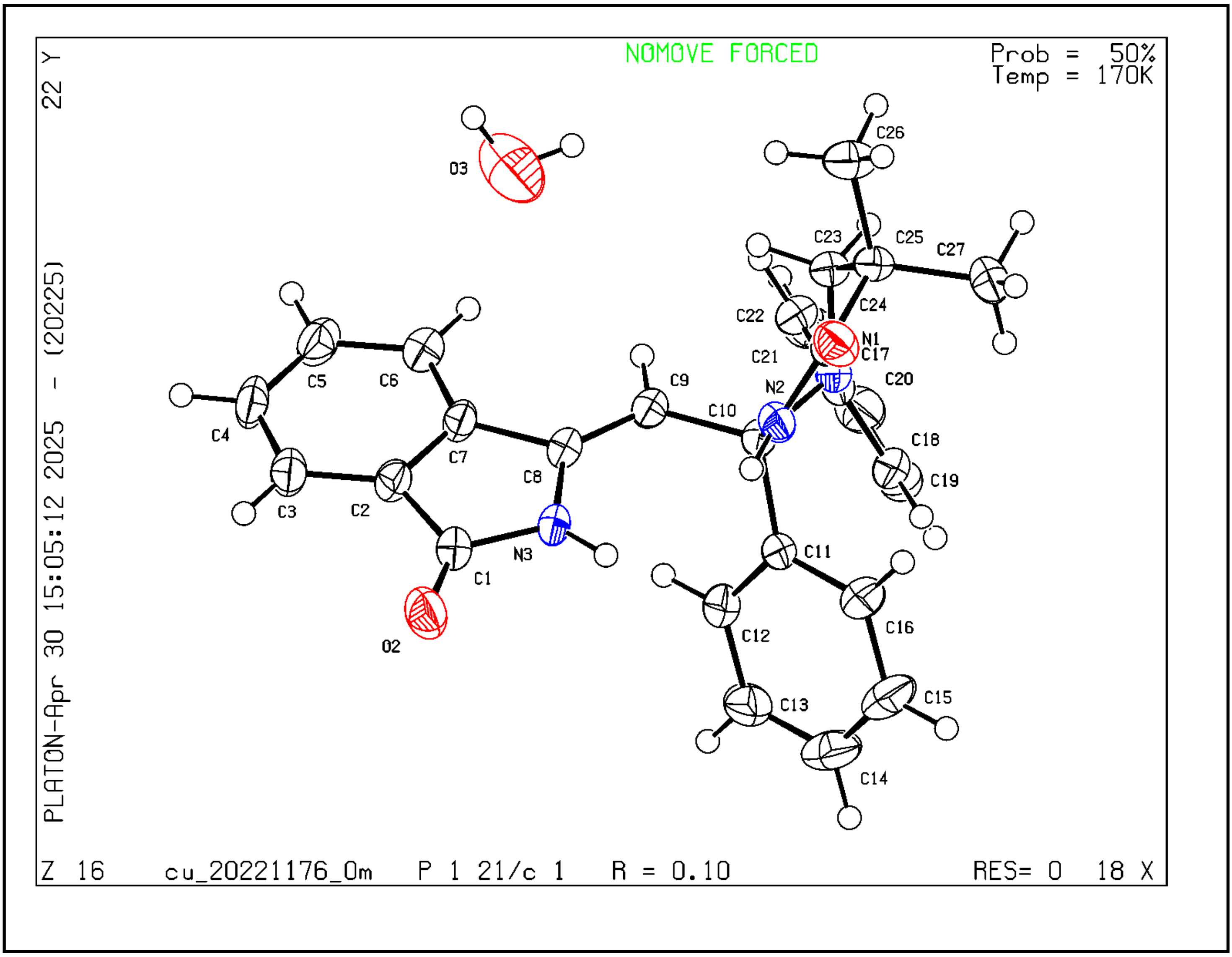

Download CIF editor (publCIF) from the IUCr
Download CIF editor (enCIFer) from the CCDC
Test a new CIF entry
