# Supplementary material for: Catalytic system-controlled divergent reactions of pyrazolidinones with 3-alkynyl-3-hydroxyisoindolinones to construct diversified nitrogen-containing heterocyclic scaffolds
Source: RSC Adv. 2025 Jun 26;15(27):21872–8. doi: 10.1039/d5ra01992c (PMC12198952; doi:10.1039/d5ra01992c)

checkCIF/PLATON report

Structure factors have been supplied for datablock(s) cu_20221175_0m

THIS REPORT IS FOR GUIDANCE ONLY. IF USED AS PART OF A REVIEW PROCEDURE FOR PUBLICATION, IT SHOULD NOT REPLACE THE EXPERTISE OF AN EXPERIENCED CRYSTALLOGRAPHIC REFEREE.

No syntax errors found. CIF dictionary Interpreting this report

Datablock: cu_20221175_0m

Bond precision:	C-C = 0.0059 A		Wavelength=1.54178
Cell:	a=14.0724(3)	b=41.3599(10)	c=16.7154(4)
	alpha=90	beta=102.813(1)	gamma=90
Temperature:	170 K		

	Calculated	Reported
Volume	9486.7(4)	9486.7(4)
Space group	P n	P 1 n 1
Hall group	P -2yac	P -2yac
Moiety formula	2(C27 H23 N3 O2), C H2 Cl2	C H2 Cl2, 2(C27 H23 N3 O2)
Sum formula	C55 H48 Cl2 N6 O4	C55 H48 Cl2 N6 O4
Mr	927.89	927.89
Dx,g cm-3	1.299	1.299
Z	8	8
Mu (mm-1)	1.662	1.662
F000	3888.0	3888.0
F000'	3904.25	
h,k,lmax	17,51,20	17,51,20
Nref	39043[19547]	36461
Tmin,Tmax	0.870,0.936	0.562,0.754
Tmin'	0.779	

Correction method= # Reported T Limits: Tmin=0.562 Tmax=0.754
AbsCorr = MULTI-SCAN

Data completeness= 1.87/0.93 Theta(max)= 74.918

R(reflections)= 0.0526(28963) wR2(reflections)= 0.1531(36461)

S = 1.024 Npar= 2430

The following ALERTS were generated. Each ALERT has the format
test-name_ALERT_alert-type_alert-level.
Click on the hyperlinks for more details of the test.

Alert level C

```
PLAT042_ALERT_1_C Calc. and Reported MoietyFormula Strings  Differ     Please Check
              Calc: 2(C27 H23 N3 O2), C H2 Cl2
              Rep.: C H2 Cl2, 2(C27 H23 N3 O2)
PLAT244_ALERT_4_C Low    'Solvent' Ueq as Compared to Neighbors of       C127 Check
PLAT244_ALERT_4_C Low    'Solvent' Ueq as Compared to Neighbors of       C073 Check
PLAT244_ALERT_4_C Low    'Solvent' Ueq as Compared to Neighbors of       C124 Check
PLAT244_ALERT_4_C Low    'Solvent' Ueq as Compared to Neighbors of       C290 Check
PLAT340_ALERT_3_C Low Bond Precision on  C-C Bonds ...............    0.00592 Ang.
PLAT911_ALERT_3_C Missing FCF Refl Between Thmin & STh/L=    0.600        72 Report
                3  2  0,   4  2  0,   3  4  0,   7 12  0,  -1  0  1,  -2  0  2,
               -3  0  3,  -4  1  5,  -4  0  6,  -4  1  6,  -5  0  7,  -4  0  8,
               -4  2  9,  -4  3  9,  -8 15  9, -10  0 12,  -9  0 13,  -8  4 13,
               -9  1 14,  -7  1 14,  -9  3 14, -11  5 14, -10  5 14, -11  7 14,
              -10  1 15, -10  2 15,  -9  2 15, -12  0 16, -10  0 16, -12  1 16,
              -11  1 16,  -9  1 16, -12  2 16, -10  2 16,  -9  2 16,  -7  2 16,
               -7  4 16, -11  0 17,  -9  0 17, -11  1 17, -10  1 17,  -9  1 17,
              -11  2 17, -10  2 17, -11  3 17, -10  3 17,  -8  3 17, -11  4 17,
              -10  4 17,  -8  4 17, -11  5 17, -10  5 17, -11  6 17, -10  6 17,
              -11  7 17, -11  8 17, -10  0 18, -10  1 18,  -9  1 18, -10  2 18,
               -9  2 18, -10  3 18,  -9  3 18, -10  4 18,  -9  4 18, -10  5 18,
              -10  6 18, -10  7 18, -10  8 18,  -8  1 19,  -8  2 19,  -8  3 19,
PLAT992_ALERT_5_C Repd & Actual _reflns_number_gt Values Differ by         18 Check
```

Alert level G

```
PLAT007_ALERT_5_G Number of Unrefined Donor-H Atoms ..............          8 Report
              H3    H60   H115  H159  H164  H218  H230  H283
PLAT012_ALERT_1_G No       _shelx_res_checksum Found in CIF ......     Please Check
PLAT112_ALERT_2_G ADDSYM Detects New (Pseudo) Symm. Elem       163         98 %Fit
PLAT112_ALERT_2_G ADDSYM Detects New (Pseudo) Symm. Elem       sub         89 %Fit
PLAT720_ALERT_4_G Number of Unusual/Non-Standard Labels ..........          4 Note
              C01O    C073    H07A    H07B
PLAT792_ALERT_1_G Model has Chirality at C12          (Polar  SpGr)         S Verify
PLAT792_ALERT_1_G Model has Chirality at C71          (Polar  SpGr)         R Verify
PLAT792_ALERT_1_G Model has Chirality at C98          (Polar  SpGr)         S Verify
PLAT792_ALERT_1_G Model has Chirality at C135         (Polar  SpGr)         R Verify
PLAT792_ALERT_1_G Model has Chirality at C173         (Polar  SpGr)         R Verify
PLAT792_ALERT_1_G Model has Chirality at C198         (Polar  SpGr)         S Verify
PLAT792_ALERT_1_G Model has Chirality at C237         (Polar  SpGr)         S Verify
PLAT792_ALERT_1_G Model has Chirality at C265         (Polar  SpGr)         S Verify
PLAT910_ALERT_3_G Missing # of FCF Reflection(s) Below Theta(Min).          1 Note
                0  1  0,
PLAT912_ALERT_4_G Missing # of FCF Reflections Above STh/L=  0.600        185 Note
PLAT913_ALERT_3_G Missing # of Very Strong Reflections in FCF ....          3 Note
                3  2  0,   4  2  0,   3  4  0,
PLAT933_ALERT_2_G Number of HKL-OMIT Records in Embedded .res File          3 Note
              -10  2 16,  -9  2 16,   0  9-10,
PLAT969_ALERT_5_G The 'Henn et al.' R-Factor-gap value ...........      2.541 Note
              Predicted wR2: Based on SigI**2  6.03 or SHELX Weight 14.95
PLAT978_ALERT_2_G Number C-C Bonds with Positive Residual Density.          1 Info
```

```
 0 ALERT level A = Most likely a serious problem - resolve or explain
 0 ALERT level B = A potentially serious problem, consider carefully
 8 ALERT level C = Check. Ensure it is not caused by an omission or oversight
19 ALERT level G = General information/check it is not something unexpected

10 ALERT type 1 CIF construction/syntax error, inconsistent or missing data
 4 ALERT type 2 Indicator that the structure model may be wrong or deficient
 4 ALERT type 3 Indicator that the structure quality may be low
 6 ALERT type 4 Improvement, methodology, query or suggestion
 3 ALERT type 5 Informative message, check
```

It is advisable to attempt to resolve as many as possible of the alerts in all categories. Often the minor alerts point to easily fixed oversights, errors and omissions in your CIF or refinement strategy, so attention to these fine details can be worthwhile. In order to resolve some of the more serious problems it may be necessary to carry out additional measurements or structure refinements. However, the purpose of your study may justify the reported deviations and the more serious of these should normally be commented upon in the discussion or experimental section of a paper or in the "special_details" fields of the CIF. checkCIF was carefully designed to identify outliers and unusual parameters, but every test has its limitations and alerts that are not important in a particular case may appear. Conversely, the absence of alerts does not guarantee there are no aspects of the results needing attention. It is up to the individual to critically assess their own results and, if necessary, seek expert advice.

Publication of your CIF in IUCr journals

A basic structural check has been run on your CIF. These basic checks will be run on all CIFs submitted for publication in IUCr journals (*Acta Crystallographica*, *Journal of Applied Crystallography*, *Journal of Synchrotron Radiation*); however, if you intend to submit to *Acta Crystallographica Section C* or *E* or *IUCrData*, you should make sure that full publication checks are run on the final version of your CIF prior to submission.

Publication of your CIF in other journals

Please refer to the *Notes for Authors* of the relevant journal for any special instructions relating to CIF submission.

PLATON version of 02/02/2025; check.def file version of 02/02/2025

Datablock cu_20221175_0m - ellipsoid plot

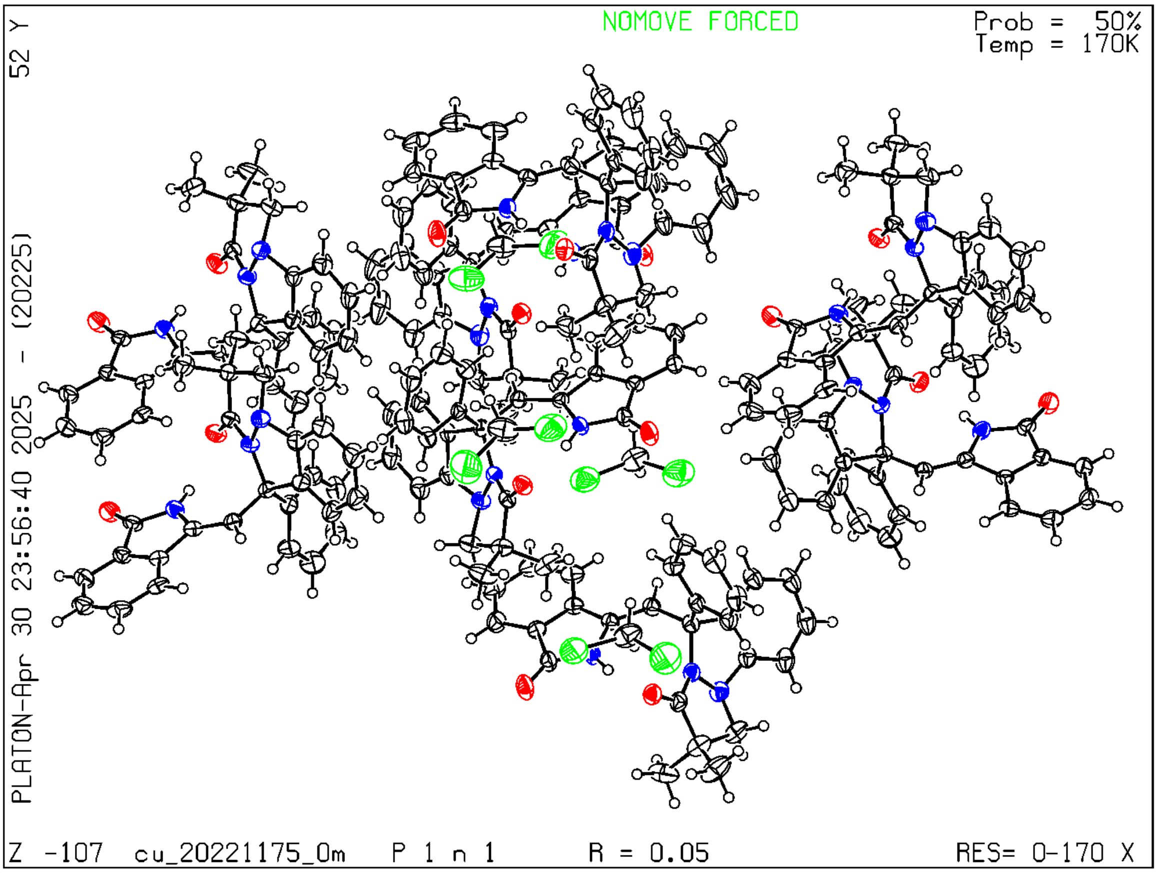

Supplement: RA-015-D5RA01992C-s003 [file RA-015-D5RA01992C-s003.pdf]
